# Geographical, landscape and host associations of *Trypanosoma cruzi* DTUs and lineages

**DOI:** 10.1186/s13071-016-1918-2

**Published:** 2016-12-07

**Authors:** Amaia Izeta-Alberdi, Carlos N. Ibarra-Cerdeña, David A. Moo-Llanes, Janine M. Ramsey

**Affiliations:** 1 El Colegio de la Frontera Sur (ECOSUR), Tapachula, Chiapas Mexico; 2 Departamento de Ecología Humana, Centro de Investigación y de Estudios Avanzados del IPN (Cinvestav) Unidad Mérida, Mérida, Yucatán Mexico; 3 Centro Regional de Investigación en Salud Pública (CRISP), Instituto Nacional de Salud Pública (INSP), Tapachula, Chiapas Mexico

**Keywords:** *Trypanosoma cruzi*, Discrete Type Unit, Host specificity, Niche identity, Ecological niche modeling, Chagas disease

## Abstract

**Background:**

The evolutionary history and ecological associations of *Trypanosoma cruzi*, the need to identify genetic markers that can distinguish parasite subpopulations, and understanding the parasite’s evolutionary and selective processes have been the subject of a significant number of publications since 1998, the year when the first DNA sequence analysis for the species was published.

**Methods:**

The current analysis systematizes and re-analyzes this original research, focusing on critical methodological and analytical variables and results that have given rise to interpretations of putative patterns of genetic diversity and diversification of *T. cruzi* lineages, discrete typing units (DTUs), and populations, and their associations with hosts, vectors, and geographical distribution that have been interpreted as evidence for parasite subpopulation specificities.

**Results:**

Few studies use hypothesis-driven or quantitative analysis for *T. cruzi* phylogeny (16/58 studies) or phylogeography (10/13). Among these, only one phylogenetic and five phylogeographic studies analyzed molecular markers directly from tissues (i.e. not from isolates). Analysis of *T. cruzi* DTU or lineage niche and its geographical projection demonstrate extensive sympatry among all clades across the continent and no significant niche differences among DTUs. DTU beta-diversity was high, indicating diverse host assemblages across regions, while host dissimilarity was principally due to host species turnover and to a much lesser degree to nestedness. DTU-host order specificities appear related to trophic or microenvironmental interactions.

**Conclusions:**

More rigorous study designs and analyses will be required to discern evolutionary processes and the impact of landscape modification on population dynamics and risk for *T. cruzi* transmission to humans.

**Electronic supplementary material:**

The online version of this article (doi:10.1186/s13071-016-1918-2) contains supplementary material, which is available to authorized users.

## Background

*Trypanosoma cruzi* is the etiological agent of Chagas disease (CD), considered one of the most important parasitic infections in Latin America. Between 25 and 90 million humans are at infection risk via at least one of multiple infection mechanisms [1]. Under natural conditions, the principal transmission modes are transplacental or via one of more than 140 hematophagous triatomine bugs (Reduviidae: Triatominae). Triatomines acquire the parasite from mammal reservoirs due to their obligate blood-feeding (albeit triatomines can also feed on non-reservoir vertebrates such as birds and reptiles) [2–5]. The disease burden for CD in the Latin America and Caribbean region, based on disability-adjusted life-years (DALYs), is at least five times greater than that of malaria, and is approximately one-fifth that of HIV/AIDS [6]. In recent decades, CD has extended to other continents outside natural reservoir or vector distributions due to human migration, with a minimum estimated 10 million individuals infected worldwide [6].

A significant number of studies have been published since 1998 to analyze *T. cruzi*’s evolutionary history using subpopulation-informative genetic markers (the year when the first DNA sequence analysis for *T. cruzi* was published). *Trypanosoma cruzi* is a diploid organism having clonal structure [7–9], strong linkage disequilibrium, and an absence of segregated and recombining genotypes [10]. Only isolated events of recombination or genetic exchange have been documented [8, 11, 12]. Recent technological developments and novel genetic markers are providing new evidence regarding the parasite’s genetic variability [13, 14], prevalence in different hosts, and geographical distribution. Multi-locus enzyme electrophoresis (MLEE), random amplified polymorphic DNA (RAPD), and other methods all demonstrate a broad genetic diversity [15, 16]. Two major lineages of the parasite, *T. cruzi* I and *T. cruzi* II, were defined originally using isoenzymes and other molecular markers such as 24S rDNA, and the mini-exon genes [16, 17]. These two clades can be further subdivided into six subclades named discrete typing units (DTU), while subsequent studies using alternative gene sequences have proposed an alternative phylogeny with three principal lineages [11, 17, 18]. The first major lineage proposal [14] designates Lineage I to include only DTUI which has been further subdivided into subgroups a, b, c, d and e based on the microsatellite region of the SL-IR and mini-exon genes [13, 19, 20]. Lineage II from the first scheme includes all other DTUs, originally designated IIa, IIb, IIc, IId and IIe. These are now assigned DTU classification IV, II, III, V and VI, respectively [14]. The newest proposal for three primary lineages is based on a multilocus phylogenetic analysis [18], in which Lineage I continues to include only DTUI, while Lineage II only includes DTUII, and Lineage III includes both DTUs III and IV [18]. The remaining hybrid DTUs, V and VI, are not assigned to any lineage, although they are closest to DTUII.

Understanding *T. cruzi* population genetics is fundamental to discern parasite’s flow within landscapes, particularly related to fragmentation and land use change, where there are native host community structures and non-native hosts (i.e. livestock, companion animals and humans). Its population dynamics is mediated by the dispersal capacity and interactions of hosts and vector species within landscapes, key components of transmission risk to humans. Since knowledge of parasite population dynamics is fundamental to design effective barriers to prevent human-vector contact and to guide effective patient treatment and clinical care [11, 21], what evidence currently exists regarding the phylogenetic patterns (i.e. speciation events) or phylogeography (i.e. geographical variation of genetic diversity) of *T. cruzi*? Is there a significant association between lineage, DTU, or subtype, with particular host taxa or vector species, ecotopes (sylvatic, transformed, domestic/peridomestic), landscape change (conserved, matrix, urbanized), biome, or latitudinal gradient? What associations exist between these variables and genetic patterns, or phylogeography of the parasite? Despite previous reviews of evidence regarding genetic diversity and phylogeography of *T. cruzi*, most have focused on gathering concluding information from publications without systematically analyzing coherence among study aims, design, sampling methods, and criteria used to reach conclusions regarding clonal evolution, presence of DTUs, eco-epidemiology, or clinical associations of parasite populations [9, 11, 13, 22]. Former proposed patterns can be re-evaluated by new evidence or as the result of incomplete experimental designs, or biased field data collections [23, 24]. An additional and more pressing problem for analyzing *T.cruzi*’s genetic diversity, variation, or structuring from a landscape perspective is the fact that, in general, most data are generated from culture or laboratory animal selected populations (parasite isolates). These difficulties to analyze parasite populations in all hosts have created voids in knowledge of intra-host and metapopulation dynamics, in addition to limitations from study designs and sampling methods, including ex-host selection (in vitro or in vivo) documented previously [25–27].

A complete review of gene sequences used in molecular diagnosis and genotyping summarizes evidence regarding their specificity and sensitivity from certain hosts [28]. MLEE [29], RAPD [30] and kDNA amplification [31], were the first techniques used for *T. cruzi* molecular identification and characterization. Methods for phylogenetic and phylogeographic studies have used the mini-exon [32], kinetoplast DNA (kDNA), ribosomal DNA (rDNA) [17], *GPI* [33] and *cytb* genes [34]. kDNA and satellite DNA (DNAsat) are the principal sequences amplified for human parasite diagnosis, the latter being more specific than kDNA in humans [28, 35]. There are different pairs of primers used for kDNA PCR (s35/36, s34/67, 32f/148r and 121/122), each with different sensitivities, and all produce false positive bands (amplification of homologous host or symbiont DNA). Hence, a combination of both kDNA and DNAsat markers have been considered an optimum solution for patient diagnosis and other aims, if sequencing is not procedural [28]. It is important to note that the sensitivity and specificity of most markers and primers have been analyzed using culture or animal model selected “isolates”, which may indicate a bias for homogeneous populations having non-polymorphic sequences, and may not be specific or sensitive additionally for those populations not tolerant to in vitro methods. Genotyping and classification techniques for *T. cruzi* have evolved from the use of RAPDs and isoenzymes, to the use of microsatellites and single nucleotide polymorphisms (SNPs). New strategies using both conventional and real time (rt) PCR techniques improve specific amplification for diagnosis in blood by using lower DNA template quantity without the high sequencing costs [28]. However, these methods have not been validated with original samples from a broad range of hosts. Several of these typing methods combined with other markers are also used for DTU classification [36–38].

The term DTU was defined for a group of isolates genetically more similar than each clade to the others, using several molecular markers [39]. Six DTUs (I-VI) were defined as noted above [14, 30], and more recently Tcbat was proposed, and the most frequently used technique to classify DTUs is a multi-primer combined PCR-RFLP analysis that discriminates between the six. Diversity within the various DTUs has required additional sub-classifications, based on additional genetic markers [19, 20, 40]. The multiplex rtPCR is a more recent quantitative method which attempts to standardize parasite DTU typing along with parasite detection, which again will depend on sensitivity to amplify a broad range of haplotypes [11, 28].

From an ecological perspective, host diversity of a parasite represents one of its ecological niche breadth components, since it reflects the diversity of resources used [41]. From an evolutionary perspective, host diversity is not merely a function of how many host species can be exploited, but also which of these are exploited, and how closely-related are they to each other [42]. A parasite’s host spectrum is a result of both location and breadth of its multidimensional ecological niche which can be measured using theoretical methods developed for community ecology [43]. Since all *T. cruzi* DTUs have been identified across a wide geographical range in the American continent, analyzing host diversity implies understanding host specificity patterns across the continent. A parasite can be highly host specific at local scales, and opportunistic at global scales, or vice versa. In the former case, the parasite uses few hosts that may be substituted across areas (host species turnover), while in the latter case, a parasite may exploit optimally a consecutive subset of host species that are regionally restricted (nested host communities). These two facets of host diversity are additive and contrasting and can be used to evaluate turnover and nestedness of *T. cruzi* (DTU or lineage) using host niches across regions [44, 45].

The current re-analysis first reviews diagnostic methods and their ability to identify subpopulations from all hosts, their DTUs and lineages, since they are fundamental tools to analyze the evolutionary history and ecological relationships of *T. cruzi*. It systematizes and weighs original research on *T. cruzi* population genetics, based on critical methodological and analytical variables. Current genetic and geographical evidence from appropriate studies have then been submitted to a re-analysis of associations with specific host taxa and geographical areas. Particular questions guiding this re-analysis were whether: (i) parasite populations typed as DTUs or lineage schemes including that proposing TcI and TcII (L1) [14], or an alternative lineage scheme proposing TcI, TcII, and TcIII (L2) [18] are broadly distributed and sympatric (or not); (ii) the suggested relationship between marsupials and armadillos and TcI and TcII of lineage scheme L1, respectively, are sustained when contrasted with representative mammal community analyses from other landscapes, and (iii) greater DTU or lineage diversity is found in any particular ecotope or host. The re-analysis evaluates whether in fact these questions could be answered and quantitatively analyzed based on currently published evidence.

## Methods

### Evidence for *T. cruzi* population genetics

A systematic search of published evidence was carried out using PubMed and Google Scholar, using the following terms: “molecular diagnosis AND *Trypanosoma cruzi*”, “discrete typing units AND *Trypanosoma cruzi*”, “population genetics AND *Trypanosoma cruzi*”, “genetic diversity AND *Trypanosoma cruzi* “, “population structure AND *Trypanosoma cruzi* “,“landscape genetics AND *Trypanosoma cruzi*”, “phylogeography AND *Trypanosoma cruzi*” and “phylogenetics AND *Trypanosoma cruzi*”, “host association AND *Trypanosoma cruzi* “, and “geographic distance and *Trypanosoma cruzi*”. An additional search was carried out, using some of the most frequent authors of the first search: “Tibayrenc”, “Barnabé”, “Brisse”, “Miles MA”, “Breniere” “Zingales”, “Oliveira RP”, “Machado”,”Guhl”, “Schijmann”, “Llewellyn”, etc., adding “AND *Trypanosoma cruzi*”. The criteria for inclusion of data from publications were that (i) they were original research on molecular diagnosis, genotyping, phylogeny, or phylogeography of *T. cruzi* as related to associations with reservoirs, vectors, geographical range, or disease associations; (ii) that they were published after 1998, the year when reproducible techniques were established for genotyping; (iii) they include DNA sequence analysis, which may also include use of restriction fragment length polymorphisms (RFLP) or the low stringency single specific primer (LSSP); and (iv) that they were published in peer reviewed indexed journals. The only exclusion criterion for studies used in re-analysis was the lack of use of statistical analytical methods for phylogeny or phylogeography. Publications were reviewed based on three principal categories: (i) study approach: aim of the study, formulation of hypotheses, and level of analysis; (ii) criteria for sample selection: origin of the population analyzed (direct from tissue, culture isolates, etc.), taxon of origin of the parasite population, and number of samples per taxon; and (iii) use of one of the following molecular markers or methods: DTU classification, mitochondrial or nuclear gene markers, size of sequences, use and number of microsatellites, and use and type of outgroups. A database was created with the above information along with information from each study results and conclusions.

All studies were classified and categorized according to four main criteria based not only on each study conclusions, but rather based on study aim and methods: molecular analytical methods, evidence generated regarding phylogenetics, evidence generated regarding phylogeography or landscape genetics, and evidence of association between parasite populations and illness. Most studies, both for phylogeny or phylogeography, did not use parasite populations directly from the host, but rather used “isolates” (58/59 for phylogeny and 11/14 for phylogeography). Sample size, geographical scale, and analytical methods were compared separately for studies using isolates *vs* original parasite populations.

### Biotic niches of *T. cruzi* DTUs and lineages

We assessed biotic (i.e. mammal and triatomine hosts) and abiotic (i.e. bioclimatic/topographic conditions) niche dimensions and divergence among current *T. cruzi* populations. Biotic niche divergence was evaluated complementarily by testing if each *T. cruzi* DTU or lineage was singularly more associated to any vector genus or mammal according to a proportional distribution of frequencies (i.e. no singular association), using a Chi-square independence test. Since there are nil or less than 5 samples per DTU or taxa in certain categories, a secondary analysis using confidence intervals to analyze for the null hypothesis that all DTUs had the same probability (17%) to be found in all taxa. We used three different classification schemes to analyze the prevalence of *T. cruzi* across reservoir orders and vector genera: (i) individual DTUs I, II, III, IV, V and VI; (ii) the L1 [14] major Lineages I and II; and (iii) the L2 [18] lineages I, II and III classifications (see Additional file 1: Table S1). A contingency table was generated with the observed frequencies reported from the literature database for *Triatoma*, *Rhodnius* and *Panstrongylus*, and separately for mammal orders, with no consideration of spatial location.

Given increasing suggestion of sorted host usage by different parasite clades, we analyzed specificity for vector and mammal community assemblages as opposed to single host orders, a measure of host specificity that reflects spatial variation in host species composition or “host spectrum” [45]. This method uses the turnover component of beta-diversity (free from the effect of nestedness) as a new measure of host specificity ("beta-specificity"), since it reflects the "pure" ability of a parasite to shift hosts from one region to another independently of any non-random and/or any nested patterns. Nestedness tends to inflate beta-diversity (and thus beta-specificity) but does not inform whether a parasite is able to shift host composition across scales, only that the parasite population infests subsets of hosts that are nested within the broader host spectrum, in a specific location. A beta-specificity index was calculated to reflect the total beta-diversity (β_SOR_) based on the Sorensen dissimilarity, by adding the dissimilarity due to "pure" host species turnover (β_SIM_, a measure of multi-site spatial turnover free from the influence of species richness based on the Simpson dissimilarity index) and β_SNE_ dissimilarity due to nestedness. The mathematical properties of these indices have been tested using different ecological circumstances and have been proven to be highly robust [46–48]. Beta specificity was calculated using the package *beta-multi.R* [49] for the R software environment (R version 3.0.2). Beta-specificity for each DTU or lineage scheme was analyzed to determine if *T. cruzi* has consistent host niche patterns (i.e. if lineages/DTUs behave differently in their host spectrum thus reflecting a difference in generalization). Presence-absence matrices were constructed for each DTU or lineage scheme by assigning regions to rows (composed of 50 × 50 km units), and host species that harbored specific lineage in a specific geographic pixel to columns. A matrix reflecting the variation in species composition and host spectrum was constructed for each DTU or lineage, and matrices were grouped by vectors, mammal hosts, or both combined.

### Abiotic niche divergence

We constructed bioclimatic niches and assessed abiotic niche divergence by conducting a two-step framework. First, ecological niche models were constructed for each DTU or lineage scheme (L1 and L2), and projected to the American continent for distribution range maps. Subsequently spatial overlap among DTUs and lineages was analyzed using a statistical framework based on null models, to determine if niche was more similar than expected randomly. The same dataset used for biotic niche evaluations (see Additional file 1: Table S1) was used to develop ecological niche models (ENM) for all DTUs, for which collection sites were reported. A total of 234 (out of 270 reported) unique data points were used for *T. cruzi* DTUI, 8 (out of 14) for DTUII, 27 (out of 38) for DTUIII, 36 (out of 53) for DTUIV, 13 (out of 13) for DTUV, and 49 data-points for DTUVI. These data points were grouped for Lineage I/L1 and L2 (234 data points), Lineage II/L1 (108 data points), Lineage II/L2 (8 data points), or Lineage III/L2 (63 data points). A total of 1997 data points for more than one DTU or lineage or for which georeference resolution was not reported, were not used for model construction. The American continent was divided into 2,632,469 pixels at a resolution of 2.5 min (0.0416667° ≈ 5 km) for latitude and longitude. Nine bioclimatic data layers (BIO1, BIO4, BIO5, BIO6, BIO7, BIO12, BIO13, BIO14 and BIO15) were obtained from Worldclim (www.worldclim.org) at a resolution of 2.5 min [50]. These bioclimatic variables were selected from a total of 19 by choosing the more meaningful variables hypothesized to limit species distributions at coarse-grain scales, after analysis of multi-collinearity in a correlation matrix [51]. The final dataset layer included variables with relatively low correlation (*r* < 0.75). Additionally, four topographic layers (aspect, slope, topographic index and elevation) were used from the Hydro 1 k data set (http://lta.cr.usgs.gov/GTOPO30). ENM’s based on occurrence data, bioclimatic, and topographic layers were constructed using the Genetic Algorithm for Rule-set Prediction (GARP) [52]. For GARP, we used a convergence criterion of 0.01 and 1000 maximum iterations; a consensus of replicate model was achieved via a 20% relative omission threshold, retaining the central 50% of the distribution of proportional areas predicted as suitable. The software randomly divides occurrence points into calibration data for model building (70%) and evaluation data for model testing (30%) [53, 54]. Each ENM was evaluated using partial ROC (receiver operating curve) using software developed by [55, 56]. The AUC (area under the ROC) was calculated using the values of “sensitivity” in the y-axis and the commission error in the x-axis, measuring the maximum inflection point where both errors are minimized. AUC values are significantly different than random when above 1.0 [51]. The 10 best subsets for each model were projected along with the data points. We used a minimum presence threshold criterion of 95% (E = 5%) in order to generate a binary map (presence/absence) of each projection from the 0–100 range of model output.

To compare similarity between niche models among DTUs or the major lineages (schemes L1 and L2), we used an identity test with the 13 climate and topographic variables for the entire continent. Niche models for identity tests were developed using MaxENT since this tool is not available for GARP [57], with 75% random test, bootstrap replicated runs, 500 maximum iterations, and minimum training presence using ENMtools v.13.2 (http://enmtools.com/). The Hellinger’s and Schoener’s indices for niche identity were calculated for all pairwise combinations of DTUs, or lineages from L1 and L2. The empirical measure of niche similarity between DTUs or lineages was compared to a null distribution for significant difference from that generated for niche models constructed with data points extracted randomly from the distribution range. The hypothesis of niche identity is rejected when the empirically observed value for the two distance indices falls to the left side of the similarity axis, outside of the frequency distribution of the random models. Observed similarities falling inside the frequency distribution or to the right of the axis are considered as insufficient evidence for lack of niche identity [58].

## Results

The dataset for re-analysis of *T. cruzi* DTUs or lineages included 59 studies on *T. cruzi* phylogeny (Tables 1 and 2) and 14 studies on its phylogeography (Tables 3 and 4). Characteristics of studies excluded from analyses are summarized in Additional file 2: Table S2 and Additional file 3: Table S3. Study design, sample selection, and criteria for molecular marker analysis of *T. cruzi* phylogeny are summarized in Table 1. Only two studies contained sample size and type, and analytical design based on a hypothesis testing approach (use of original parasite samples and outgroups). The number of samples used in each study was highly variable and almost all, except for four studies, analyzed isolates selected from previous studies (either using culture and/or laboratory animal selection). Two studies analyzed both cultured isolates and original parasite samples, although differential results for both are not presented. Complete or partial classification of DTUs was carried out in 45 of 59 studies, some of these used samples that had been classified in previous studies (27/59). Only a minor proportion of the phylogenetic studies used quantitative methods to validate results (appropriate outgroup, probabilities, and confidence intervals) (17/59) (Table 2).

**Table 1:** Classification of phylogenetic studies based on review categories

Review category	Classification	No. of studies	Reference
Spatial level	Continental	29	[18, 33, 40, 88–113]
	National	17	[19, 20, 27, 73, 114–126]
	Regional	13	[127–139]
Aims	Phylogenetic relationship and/or genetic diversity	42	[19, 20, 33, 40, 73, 96, 100–113, 120–125, 132–139]
	Population structure and/or genetic diversity	9	[88–91, 93, 94, 114, 117, 126]
	Genetic diversity and/or host association	3	[27, 116, 127]
	Genetic diversity and genome or proteome association	5	[18, 92, 95, 115, 128]
Hypothesis	Study and sample design based on a hypothesis	2	[133, 136]
	Study and sample design not based on a hypothesis	57	[18–20, 27, 33, 40, 73, 88–132, 134, 135, 137–139]
Sample source	Humans, other mammals, and triatomines	43	[18–20, 33, 40, 73, 88, 90, 92–99, 102–120, 122–124, 126–128, 131, 139]
	Triatomines and humans	6	[27, 89, 100, 121, 130, 134]
	Triatomines and other mammals	2	[125, 137]
	Triatomines	4	[129, 133, 135, 136]
	Other mammals	1	[91]
	Humans	3	[131, 132, 138]
Parasite population analyzed	Isolates selected using in vitro or in vivo methods	53	[18–20, 33, 73, 88–128, 130–132, 134, 135, 137, 139]
	Original sample	4	[27, 129, 133, 136]
	Isolates + original	2	[40, 138]
Molecular marker used for parasite diagnosis and genotyping	DTU	9	[89, 90, 94, 97, 99, 104, 112, 122, 127]
	MLEE +/or RAPD + DTUs	9	[18, 33, 95, 105–109, 111]
	Mini-exon + 24S rRNA	9	[91, 96, 113, 116, 130–132, 135, 138]
	MLEE +/or RAPD	6	[100, 103, 114, 118, 137, 139]
	Mini-exon + 24S rRNA + DTU	2	[73, 110]
	Mini-exon + MLEE+/or RAPD	2	[88, 102]
	GPI	2	[117, 128]
	Cyt b	1	[125]
	24SrRNA	6	[92, 93, 98, 115, 127, 133]
	Mini-exon	10	[19, 20, 40, 119–121, 123, 124, 134, 136]
	kDNA (121/122)	1	[126]
	kDNA (S35/S36)	2	[27, 129]
DTU	Classified	19	[19, 20, 27, 40, 73, 98, 116, 119–121, 123, 125, 128, 130, 133–137]
	Previously classified	26	[18, 33, 89–91, 94, 95, 97, 100, 102, 104, 106–112, 114, 117, 118, 122, 124, 126, 127, 131]
	Not classified	14	[16, 88, 92, 93, 96, 99, 103, 105, 113, 115, 129, 132, 138, 139]
Population genetic analyses	Nuclear	23	[19, 20, 33, 40, 89, 96–99, 101, 105, 106, 110–113, 117, 120, 127, 133, 135–137]
	Mitochondrial	3	[108, 116, 125]
	Nuclear + mitocondrial	7	[18, 73, 100, 103, 107, 118, 122]
	Nuclear + mitochondrial + microsatelite	4	[88, 90, 104, 131]
	Nuclear + microsatellite	1	[121]
	Mitochondrial + microsatellite	2	[94, 128]
	Microsatelite	10	[27, 91–93, 102, 109, 114, 115, 130, 132]
	LSSP or RFLP	9	[95, 119, 123, 124, 126, 129, 134, 138, 139]
Outgroups	*T. c. marinkelli*	9	[95, 106–108, 116, 125, 127, 135, 136]
	*T. c. marinkelli + T. dionisii*	1	[73]
	*T. c. marinkelli + T. rangeli*	2	[33, 101]
	*T. c. marinkelli + T. brucei*	1	[122]
	*T. c. marinkelli + Leishmania major + L. donovani*	1	[96]
	*T. c. marinkelli + T. vespertilionis*	3	[18, 100, 103]
	*T. c. marinkellei + T. rangeli + T. brucei*	1	[90]
	*T. rangeli + T. dionisii*	1	[119]
	*T. rangeli*	5	[113, 123, 124, 126, 133]
	*TcII* and/ or T*cIV*	4	[20, 117, 120, 128]
	*Leishmania chagasi*	1	[134]
	*B. caudatus + T. borreli + T. rangeli*	1	[98]
	Not used	29	[19, 27, 40, 88, 89, 91–94, 97, 99, 102, 104, 105, 109–112, 114, 115, 118, 121, 129–132, 137–139]

**Table 2:** Phylogenetic studies which use quantitative analytical methods

Article	Hypothesis/Aim	Parasite population	Sample size	Geographical scale	Temporal scale (yrs)	Population genetic analysis	Outgroup	Statistical analytical method
Flores-Lopez & Machado [18]	Reconstruction of the evolutionary history of Tc	Isolates	7	Tc stocks	md	Nucleotide sequences from 32 loci	*T. c. marinkellei, T. vespertilionis*	Test of selection, divergence time estimates
Venegas et al. [27]	Specific host-parasite association in Chilean populations of Tc	Original	117	Chile	2	Microsatellite loci	none	Phylogram tree (NJ)/Genetic differentiation
Barnabe et al. [114]	Subsample analyses of MLMT structuring among reference stocks belonging to known DTUs	Isolates	94	Bolivia and Peru	32	Microsatellite loci	none	NJ trees/Fixation indices F_IS_ and F_ST_/Genetic diversity Hs/ANOVA
Freitas et al. [88]	Dissect the multilocus genotypes into their constituent haploid genome blocks to understand Tc evolutionary history	Isolates	75	Brazil	md	Microsatellite loci, 24SrRNA, *cox*2 sequencing/ RFLP	none	Distance matrices, multidimensional scaling and NJ tree/Haplotype inference and network construction
Ienne et al. [89]	Test hybridization hypothesis	Isolates	9	Tc stocks	md	195 SAT	none	Phylogenetic inference (NJ)/Network
Lauthier et al. [127]	Stability of multilocus genotypes as the required condition for any molecular epidemiology approach (strain typing)	Isolates	32	Argentina	md	Multilocus sequence typing (10 targets)	*T. c. marinkellei*	Phylogenetic tree/Genotype network
Lewis et al. [90]	Origins and evolution of Tc at several overlapping levels	Isolates	35	South America	md	GPI, *cox*2-*nad*1, microsatellite loci	none	Bayesian Inference, microsatellite analysis
Llewellyn et al. [91]	Within-host diversity in TcI	Isolates	211	Bolivia, Venezuela and Brazil	5	Microsatellite loci	none	Genetic distance (DAS), F_IS_ (FSTAT), AMOVA and index of association
Macedo et al. [92]	Usefulness of microsatellite typing in population genetic studies of Tc	Isolates	53	Tc stocks	md	Microsatellite loci	none	Wagner network
Oliveira et al. [115]	Population structure of the parasite	Isolates	30	Brazil and Colombia	md	8 microsatellites	none	Wagner network
Oliveira et al. [93]	Population structure of Tc	Isolates	54	md	md	8 microsatellites	none	Tests for Hardy-Weinberg and linkage disequilibrium/Wagner network
Pena et al. [94]	Population structure of TcI	Isolates	75	Tc stocks	md	Microsatellite and mitochondrial sequences	none	NJ/ Network
Ramirez et al. [116]	Genetic variability within TcI clones and concordance with the established genotypes	Isolates	70	Colombia	md	*cytb* sequencing	*T. c. marinkellei*	Phylogenetic tree/Genotype network
Ramirez et al. [128]	Contemporary cryptic sexuality in Tc	Isolates	369	Colombia	10	Microsatellite and mitochondrial sequences	DTUII and DTUIV	Genetic diversity/NJ/ML/BEAST
Ramirez et al. [117]	Nuclear MLST markers to unravel the genetic structure of TcI in Colombia	Isolates	50	Colombia	11	Nuclear multilocus sequence typing	DTUII and DTUIV	Genetic diversity and diploid sequence types (DSTs)
Telleria et al. [95]	Association between Tc subspecific phylogenetic diversity and levels of protein expression	Isolates	26	Tc stocks	md	Proteomics data	*T. c. marinkellei*	MLEE genetic distances and proteomic Euclidian distances
Tomazi et al. [96]	Hybrids are of polyphyletic origin, evolving independently from various hybridization events	Isolates	26	South America	md	Sequences of SSU rDNA, EF-1α, actin, DHFR-TS and TR genes	none	Phylogeny inference and network geneologies

*Abbreviations*: *md* missing data, *Tc Trypanosoma cruzi*

**Table 3:** Classification of phylogeographic studies based on review categories

Review category	Classification	No. of studies	Reference
Spatial scale	Continental	5	[67, 68, 140–142]
	National	4	[77, 78, 143, 144]
	Regional	5	[64, 80, 81, 84, 145]
Aims	Phylogenetic relationships	4	[64, 142, 144, 145]
	Genetic diversity and phylogenetic relationships	2	[78, 141]
	Genetic diversity and spatial associations	4	[67, 68, 81, 143]
	Population structure and spatial associations	2	[77, 84]
	Spatial association and haplotype-host association	2	[80, 140]
Hypothesis	Study and sample design based on a hypothesis	2	[80, 84]
	Study and sample design not based on a hypothesis	12	[64, 67, 68, 77, 78, 81, 140–145]
Sample source	Humans, other mammals and triatomines	8	[64, 68, 80, 84, 140–142, 144]
	Triatomines and other mammals	5	[67, 77, 78, 81, 145]
	Triatomines	1	[143]
Parasite population analyzed	Isolates selected using in vitro or in vivo methods	11	[64, 67, 68, 77, 78, 81, 140–142, 144, 145]
	Original sample	3	[80, 84, 143]
Molecular marker used for diagnosis and genotyping	Mini-exon	3	[80, 140, 144]
	24S rRNA	1	[67]
	24S rRNA + mini-exon	3	[64, 68, 142]
	S35/S36 kDNA	2	[81, 143]
	S35/S36 + TcZ1/TcZ2 + mini-exon + 24S rRNA + 18S rRNA	1	[145]
	MLEE +/or RAPD+ miniexon	1	[84]
	LSU rDNA + HSP60 + GPI	1	[77]
	GPI	2	[78, 141]
DTU classification	Classified in study	13	[64, 67, 68, 77, 78, 80, 81, 84, 141–145]
	Previously classified	1	[140]
Population genetic analysis	Nuclear sequences	3	[80, 140, 145]
	Nuclear + mitochondrial sequences	2	[64, 142]
	Microsatellites	3	[68, 81, 143]
	Nuclear sequence and microsatellite	1	[67]
	Mitochondrial sequence and microsatellite	3	[77, 78, 141]
	RFLP or LSSP-PCR	2	[84, 144]
Outgroups	*T. c. marinkelli*	1	[67]
	*T. c. marinkelli + T. dionisii*	1	[142]
	DTUI	1	[77]
	DTUII	2	[84, 144]
	DTUIII + DTUIV	1	[141]
	DTUIV	1	[78]
	none	7	[64, 68, 80, 81, 140, 143, 145]

**Table 4:** Phylogeographic studies using quantitative analytical methods

Article	Hypothesis/Aim	Parasite population	Sample size	Geographical scale	Temporal scale (yrs)	Population genetic analysis	Outgroup	Statistical analytical method
Llewellyn et al. [67]	Population genetics of sylvatic TcIIc from South America (diversity, spatial structure and climatic associations)	Isolates	53	Colombia, Brazil, Venezuela, Bolivia and Paraguay	25	GPI sequencing/ microsatellite	*T. c. marinkellei*	NJ/Genetic diversity (Ar/He/Ho/HD)/Mantel test
Llewellyn et al. [68]	Population genetics of sylvatic TcI/diversity associated with epidemiological relevance	Isolates	135	Americas	22	Microsatellite	none	Genetic diversity (Ar/He/Ho/HD)/Mantel test
Messenger et al. [77]	Population structure, hybridization and role for humans in parasite dispersal	Isolates	199	Bolivia	6	Microsatellite and maxicircle	DTUI	Population genetic parameters/NJ/Fst/ Mantel test/ML
Lima et al. [78]	Genetic diversity, genetic exchange and impact of ecological disturbance	Isolates	107	Brazil	md	Microsatellite and maxicircle	DTUIV	Genetic diversity parameters/ML
López-Cancino et al. [80]	Relationships between parasite diversity, host metacommunities, and vectors in a human-disturbed gradient	Original	81	Yucatán, México	5	Mini-exon sequencing	none	Network analysis/Phylogenetic (ML)
Ocaña-Mayorga et al. [81]	Genetic subdivision by transmission cycle, and anthropogenic dispersal between communities and panmixia among strains	Isolates	81	Loja Province, Ecuador	md	Microsatellite	none	Genetic diversity (Ar/PA/FIS/FST)/ AMOVA/DAS/Mantel test
Rodriguez et al. [84]	Transmission dynamics of Tc genotypes	Original	121	Colombia	md	LSSP-PCR, Southern Blot	DTUII	Nei's distance and NJ/AMOVA
Herrera et al.[140]	Sequence variability of the SL-IR	Isolates	244	11 Latin American countries	md	Mini-exon sequencing	none	Network/PCA
Herrera et al. [145]	Genotype diversity of Tc in Louisiana	Isolates	15	New Orleans, Louisiana (USA)	4	Miniexon sequencing	none	Phylogenetic (ML)
Venegas et al. [143]	Geographical structure and genetic differences among or within lineages in Chilean Tc populations	Original	64	Chile	2	Microsatellite	none	Fisher's exact test, AMOVA, F_ST_, Mantel test
Zumaya-Estrada et al. [141]	Dispersal among domestic transmission cycles of Tcdom in northern South America, sister group of North American strains	Isolates	72	Americas	md	Microsatellite	DTUIII, DTUIV	Genetic diversity/Bayesian topology/NJ/MYA geographic calibration point

*Abbreviations*: *md* missing data, *Tc Trypanosoma cruzi*, *Tcdom*, *Trypanosoma cruzi* domestic genotype

Fourteen studies analyzed an association between *T. cruzi* genetic and geographical distance (Table 3), although only one analyzed genetic distance at the landscape level. Two studies analyzed population structure and spatial associations, and another two studies analyzed spatial and haplotype-host associations. Only two studies had compatible specific aim and sample or analytical design, while only three studies analyzed original samples as opposed to isolates. DTUs were classified in 13 of 14 studies, even though some of these did not complete DTU typing. Most of these studies (11/14) used quantitative analytical methods to validate results (Table 4).

A total of 1402 vector specimen records from 56 species across 6 genera were included in the present analysis, although sufficient data points for quantitative analysis with DTUs or lineages was available only for the three major genera: *Triatoma*, *Rhodnius* and *Panstrongylus* (Table 5). A total of 975 mammal specimens infected with any *T. cruzi* DTU or lineage were recorded from 95 mammal species, categorized by order (Table 5). The summary of *T. cruzi* DTUs recorded from mammals and used in this re-analysis is summarized by order, including Carnivora (with and without pets) and Primates without humans, respectively (see Additional file 4: Table S4). Current data do not report any presence of certain DTUs in specific hosts, or there are less than 5 reports per taxa-DTU. An analysis of confidence intervals indicates that the probability of true negative was below 67% for DTUII, DTUIII, DTUIV and DTUV only in Artiodactyla, while in all other taxa and for all DTUs, the probability was greater than 98%.

**Table 5:** *Trypanosoma cruzi* DTUs and lineages reported from vector and mammal species and samples in literature

	No. of species of vectors	Vector samples (*n*)	No. of species of mammals	Mammal samples (*n*)
DTUI	29	1204	44	640
DTUII	5	14	10	30
DTUIII	7	52	10	87
DTUIV	10	36	21	103
DTUV	2	41	3	41
DTUVI	3	55	7	74
L1-I	29	1204	44	640
L1-II	27	198	51	335
L2-I	29	1204	44	640
L2-II	5	14	10	30
L2-III	17	88	31	190

### *Trypanosoma cruzi* DTU associations with specific vectors, hosts and geographical range

**Fig 1:**
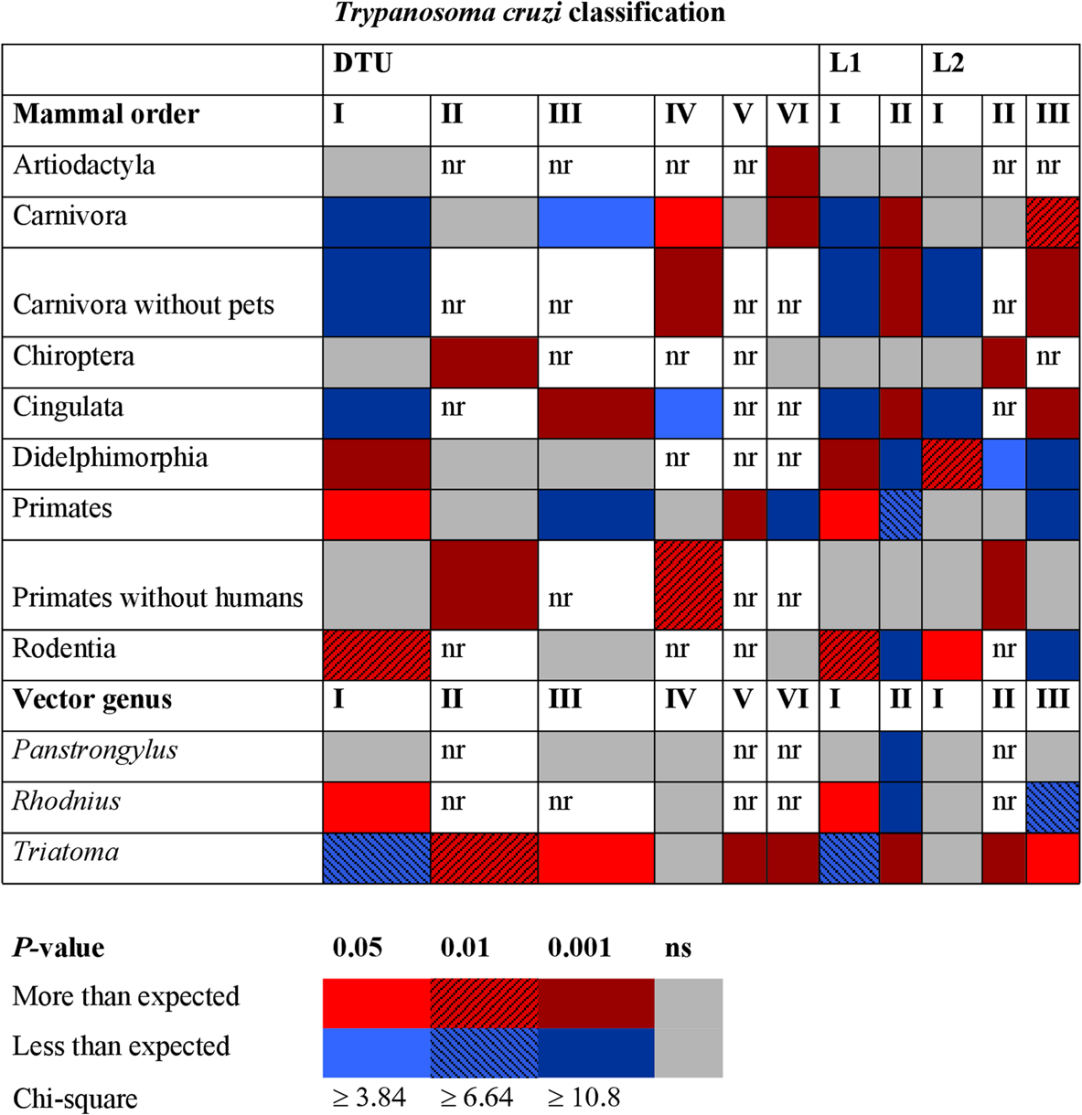
Frequency significance for *Trypanosoma cruzi* DTUs and lineages in mammal orders and three primary vector genera. *Abbreviations*: L1, Lineage L1 [14]; L2, Lineage L2 [18]. *Abbreviations*: nr, not reported; ns, not significant

DTU frequenciess across reservoir taxa was not independent of DTU class, indicating a significant difference from expected (*χ* ^2^ = 1,334.084, *df* = 40, *P* < 0.0001). This was particularly the case for Carnivora, Didelphimorphia, Primates (with and without humans), and Rodentia. Only DTUI and VI have been reported in Artiodactyla, with a significant 10-fold increase from expected for DTUVI (*P* < 0.001) (Fig. 1). All DTUs were reported in Carnivora, although significantly more DTUIV (*P* < 0.05) and VI (*P* < 0.001), while significantly less than expected DTUI (*P* < 0.001) and III (*P* < 0.05). If pets were excluded from Carnivora, only an increase of DTUIV (*P* < 0.001) and a decrease of DTUI (*P* < 0.001) were significant, since DTUVI and DTUIII were only recorded in pets. DTUII was reported significantly more than expected in Chiroptera (*P* < 0.001); both DTUVI and DTUI were also reported from this latter order, but their frequencies were not significantly different from expected. Statistical inferences for the three previous groups must be considered preliminary, given the low sample size currently genotyped.

Only DTUI, III and IV have been reported from the Cingulata, with significantly greater than expected DTUIII (*P* < 0.001), although significantly less DTUI (*P* < 0.001) and DTUIV (*P* < 0.05) in the order. DTUI, II and III have been reported from Didelphimorphia, with significantly more than expected DTUI (*P* < 0.001) and no significant difference from expected frequencies of the other two. Only DTUs I, III and VI have been reported in Rodentia, although only DTUI was reported significantly more than expected (*P* < 0.01).

All DTUs were recorded from Primates, although only DTUs I, II and IV have been reported in non-human Primates (Fig. 1). If non-human Primates are analyzed alone, both DTUII (*P* < 0.001) and IV (*P* < 0.01) were recorded significantly more than expected; neither was significant if humans were included. Primates including humans had significantly greater frequencies of DTUI (*P* < 0.05) and DTUV (*P* < 0.001); the significance is inferred only for *Homo sapiens* since the frequencies were not significant if humans were not included. DTUIII and VI (along with DTUV) were recorded less than expected (*P* < 0.001) only if humans were included in the Primate group.

When DTUs were grouped into one of the two major lineage schemes, there were differences in recorded frequencies across reservoir taxa (*P* < 0.001, Fig. 1). With the exception of Artiodactyla, Chiroptera and non-human Primates, all frequencies for individual mammal orders were significant for Lineage II/L1, similar to DTUI frequencies. The Lineage I/L2 frequency for Primates was no longer significant due to modified DTU frequencies. The Lineage II/L2, similar to DTUII, was more frequently reported from Chiroptera and Primates without humans, although significantly less than expected in Didelphimorphia. Lineage II/L1 was recorded significantly less than expected in Didelphimorphia, all Primates, and Rodentia, while more than expected in Cingulata and Carnivora, with or without pets, similar for Lineage III/L2.

Similar to that observed for reservoir taxa, there were significant differences of DTU frequencies in the three principal vector genera *Triatoma*, *Panstrongylus* and *Rhodnius* (*P* < 0.001, Fig. 1). Interestingly, there were no significant frequencies for any of the vector genera with DTUIV, and no significant frequencies of any DTU (I, III, or IV) in *Panstrongylus*, the latter potentially due to few records. DTUs II, V and VI have only been occasionally reported from other than the genus *Triatoma* in which they are recorded significantly more than expected (*P* < 0.01, *P* < 0.001 and *P* < 0.001, respectively). DTUIII was only recorded significantly in *Triatoma* (*P* < 0.05). Lineage II/L1 was recorded significantly less than expected in *Rhodnius* and *Panstrongylus* (*P* < 0.001), while more than expected in *Triatoma* (*P* < 0.001). Lineage III/L2 was recorded also significantly less in *Rhodnius* (*P* < 0.01). The frequency of DTUI in *Rhodnius* was significantly more than expected (*P* < 0.05), while that in *Triatoma* was significantly less (*P* < 0.01). The lineage I/L1 frequency was also significantly greater in *Rhodnius* (*P* < 0.05), while not significant for Lineage I/L2 in this genus. Lower or nil significance of DTUI in *Triatoma* was also observed for Lineage I/L1 and Lineage I/L2.

### Beta-specificity

**Fig. 2:**
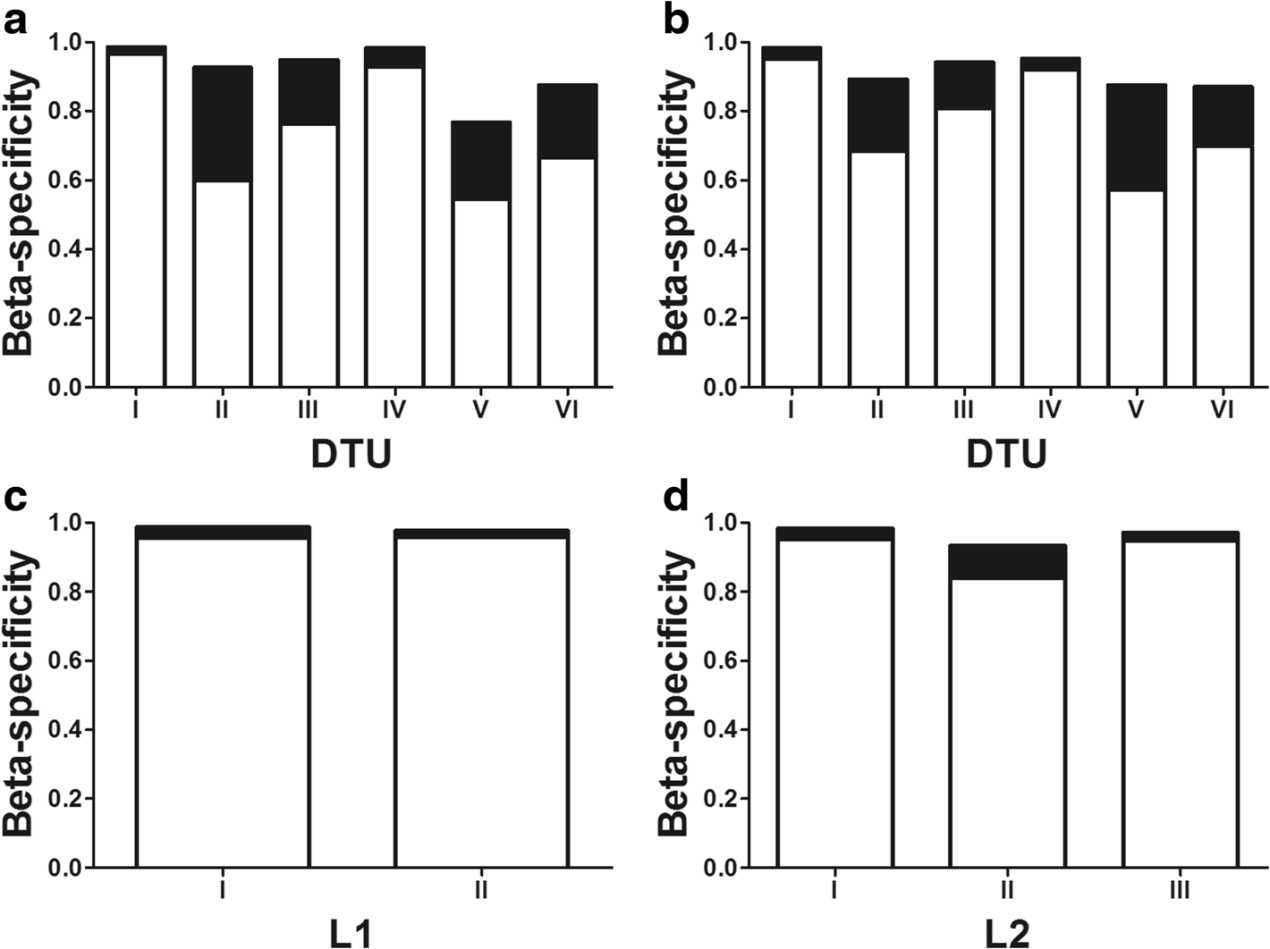
Host beta-specificity for *Trypanosoma cruzi* DTUs (**a**) for the three primary vector genera and (**b**) for mammal orders. Beta specificity for mammals according to major lineage schemes (**c**) L1 and (**d**) L2. Bars in *white* are beta dissimilarity due to host species turnover and in *black* due to nestedness. The range is between 0 (absence of species interchange across multiple regions) and 1 (complete species interchange across regions)

Overall beta-specificity (β_SOR_) approached 1 for all DTUs, reflecting a high rate of species turnover when all hosts (mammals and vectors) were considered (Fig. 2). In general, *T. cruzi* is opportunistic across geographical scales since beta-specificity free from species turnover (β_SIM_) was highest and uniform in contrast to nestedness (β_SNE_), for all DTUs and for all lineages of the L1 and L2 schemes (data shown only for mammals, although similar for vectors) (Fig. 2c, d). Beta specificity free from species turnover was lowest for DTUs II, V and VI, with ${\overline{\upbeta}}_{\mathrm{SNE}}$ ranging from 0.71–0.78 in both vectors and mammal hosts (Fig. 2a, b). Beta specificity free from species turnover averaged ${\overline{\upbeta}}_{\mathrm{SIM}} = 0.97$ for L1 and ${\overline{\upbeta}}_{\mathrm{SIM}} = 0.96$ for L2 (Fig. 2c, d), while it was slightly lower ${\overline{\upbeta}}_{\mathrm{SIM}} = 0.84$ for all DTUs. DTUs I and IV had lowest specificity due to nestedness in both mammals and vectors.

### Ecological niche models of *T. cruzi*

**Fig. 3:**
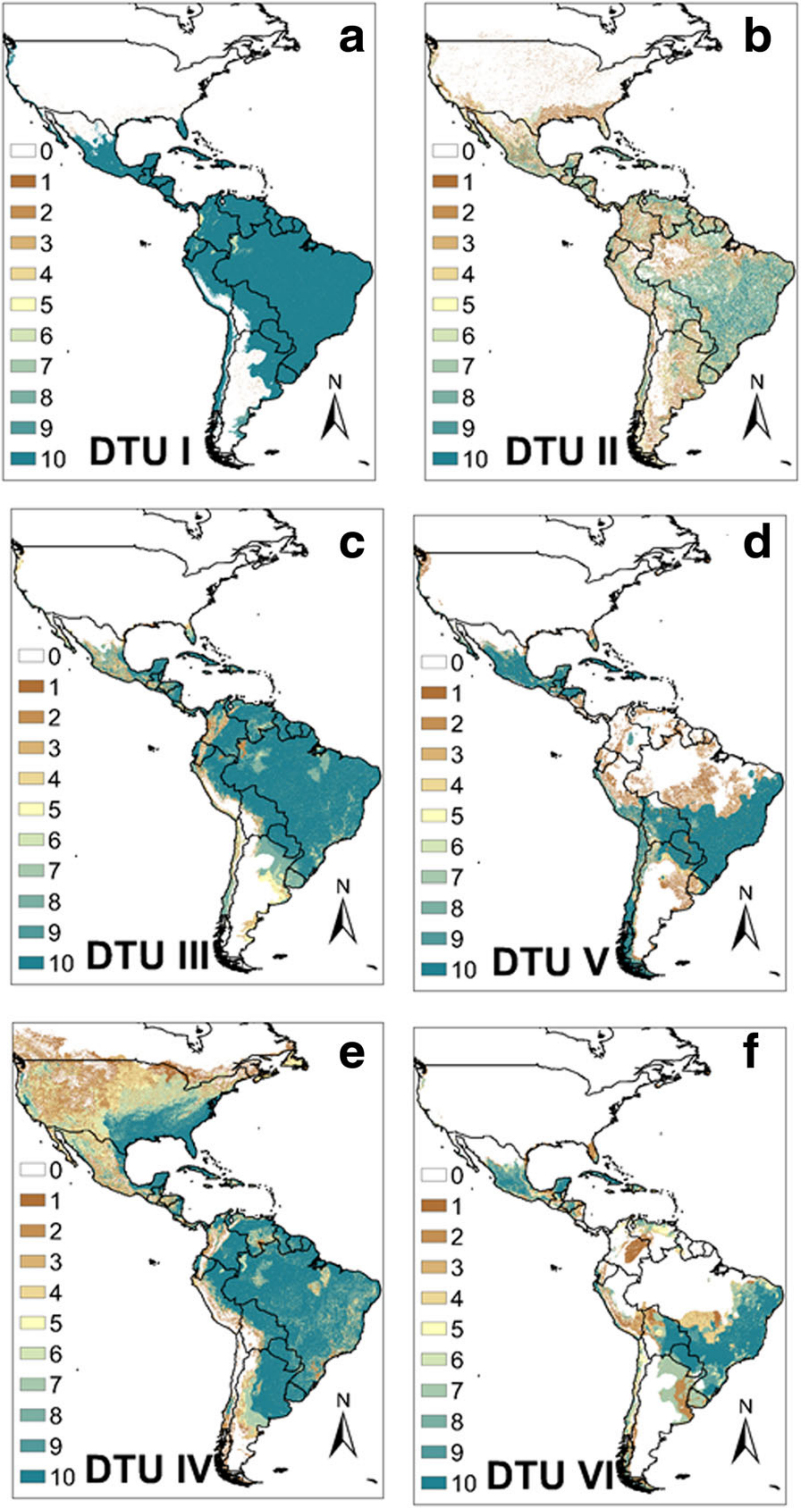
Ecological niche models for all *Trypanosoma cruzi* DTUs with classification of best (10) to worse (1) subsets.  **a** DTUI. **b** DTUII. **c** DTUIII. **d** DTUV. **e** DTUIV. **f** DTUVI

**Fig. 4:**
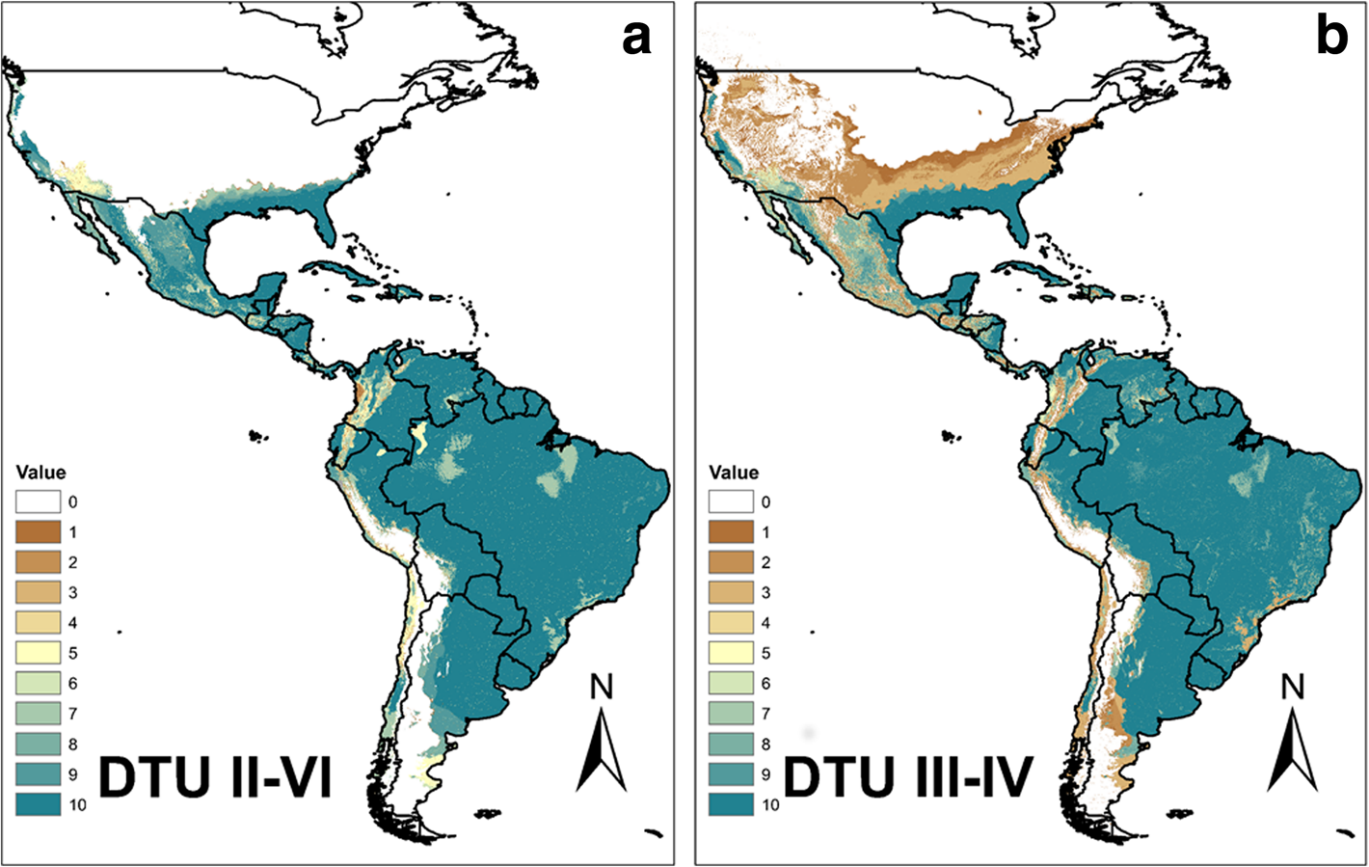
Ecological niche models for lineages from both principal schemes. Ecological niche models for *Trypanosoma cruzi* lineage I of L1 (**a**) and lineage III of L2 (**b**) with classification of best (10) to worse (1) subsets

Ecological niche models were constructed separately for all DTUs (Fig. 3) and both lineage schemes (Fig. 4). All niche models were highly accurate well above random expectation, the AUC values ranging from 1.2–1.9; the lowest AUC ratio was for Lineage II/L1 (see Additional file 5: Figure S1). The DTUIV ENM spans from the Nearctic region of Mexico, across the southeast USA to Argentina (Fig. 3e). DTUIII projects to a more reduced range, from southern Mexico to Argentina, with less coverage on the dryer Pacific coast (Fig. 3c). The ENM of DTUI (and Lineage I) projects throughout the Neotropical region from mid-Mexico to northeastern Argentina (Fig. 3a). The ENM for Lineage III/L2 includes projection similar to that of DTU III and IV, extending from the southeast and southwest USA to southern Argentina (Fig. 4b). In contrast to the geographical range of the former three clades, DTUII (and Lineage II/L2) has far more sparse geographical projection, which is primarily across Central America (CA), and the non-Amazon regions of Venezuela and southern Brazil (Fig. 3b). The ENM of DTUV and VI are more dense, although focused to the non-equatorial Neotropical regions of the continent (Mexico, Central America, southern Brazil, Bolivia) (Fig. 3d, f). The ENM for Lineage II/L1 (DTUs II–VI) includes areas from all individual constituent DTUs (Fig. 4a). The null hypothesis for lack of niche identity was rejected, i.e. niche dissimilarity was only significant for comparison of DTUIV and DTUVI (*P* <0.01). Distributions for all other comparisons were no different from random (Table 6).

**Table 6:** Hellinger’s Index and Schoener’s D Index and corresponding 95% confidence intervals (CI) for niche dissimilarity calculated for all pairwise combinations of DTUs or lineages from L1 and L2 schemes

Group	Comparison	*n*_*a*_ */n*_*b*_	Hellinger’s Index	95% CI	Schoener’sIndex	95% CI
	I *vs* II	234/8	0.93	0.35–0.95	0.73	0.15–0.95
	I *vs* III	234/27	0.91	0.55–0.90	0.70	0.35–0.90
	I *vs* IV	234/36	0.69	0.50–0.95	0.45	0.25–0.90
	I *vs* V	234/13	0.85	0.55–0.95	0.64	0.30–0.90
	I *vs* VI	234/49	0.80	0.70–0.95	0.53	0.50–0.90
	II *vs* III	8/27	0.96	0.20–0.85	0.79	0.10–0.80
DTU	II *vs* IV	8/36	0.67	0.30–0.80	0.42	0.10–0.80
	II *vs* V	8/13	0.83	0.35–0.90	0.67	0.25–0.90
	II *vs* VI	8/49	0.79	0.15–0.80	0.52	0.00–0.80
	III *vs* IV	27/49	0.71	0.55–0.90	0.48	0.40–0.85
	III *vs* V	27/13	0.82	0.40–0.95	0.63	0.25–0.90
	III *vs* VI	27/49	0.77	0.45–0.90	0.50	0.20–0.90
	IV *vs* V	36/13	0.70	0.30–0.80	0.43	0.20–0.70
	IV *vs* VI	36/49	0.38*	0.50–0.85	0.18*	0.30–0.85
	V *vs* VI	13/49	0.69	0.30–0.75	0.42	0.15–0.70
Lineage (L1)	I *vs* II	234/133	0.91	0.80–0.95	0.73	0.65–0.95
	I *vs* II	234/8	0.93	0.25–0.90	0.73	0.00–0.90
Lineage (L2)	I *vs* III	234/63	0.86	0.70–0.95	0.65	0.55–0.95
	II *vs* III	8/63	0.84	0.25–0.85	0.63	0.00–0.85

*Abbreviation*: *n*_*a*_ */n*_*b*_, number of samples used for each comparison

*P < 0.01

## Discussion

Continuous reports of natural infections with *Trypanosoma cruzi* in many phylogenetically distant mammal orders provide evidence that it is a generalist parasite. Nonetheless, some attempts have been made to statistically test hypotheses for certain DTU specificities with host genera or orders. While this is a valid question, the broad extension of the *T. cruzi* clade across the American continent would require an ambitious sampling design and coverage of all mammal orders in study sites, which would require important funding, currently unlikely given reduction in science and technology budgets. The present study analyzes existing data from both single and multisite studies, by following a systematic and critical review, key associations between parasite clades and vector or mammal host taxa [59, 60]. Very few studies have included appropriate host diversity analyses, thus limiting assessment of potential DTU specificity. This would provide evidence for overlapping patterns of all DTU ranges (suggesting true generalization), or of a composite range of individual DTU ranges (indicating some degree of ecological or spatial restrictions that can be interpreted as specialization). To our knowledge, this is the first study that systematically and directly analyzes the published literature that intentionally or not reports the interaction between *Trypanosoma cruzi* and its hosts, after having been filtered based on methodological criteria for DTU detection, if the specimens in fact harbored the parasite. Two methodological assumptions regarding DTU specificity have been made, the first that the gene markers analyzed and the primers used can identify all DTUs (no DTU primer specificity), and the second is that host tissues used as a source of DTU DNA, can harbor any DTU with equal probability (no DTU tissue specificity). These two assumptions have been rarely assessed and when they have, are generally limited, and hence we encourage efforts to test the validity of these assumptions. Since the data analyzed herein rely on published information, and there is a paucity of reports from certain taxa and certain DTUs, we also caution potential analytical bias.

The majority of *T. cruzi* phylogenetic studies use additive retrospective collections, analyzing a sum of samples generated from multiple previous studies, without common hypothesis-driven design, thus with limited control of potential confounder factors and bias. These latter studies compile previously generated gene sequences in order to generate evolutionary information from those obtained, but not for the parasite, i.e. they lack external validity. We have used previous data filtered for certain criteria to analyze new questions regarding associations, rather than a meta-analysis approach, which would generate information on the magnitude and variability of effects. Previous individual studies were included based on reliability and certain core conceptual or experimental criteria, and obviously imply heterogeneous geographical coverage. This latter bias cannot be reconciled for this analysis, but should be considered specifically for future studies.

Many technical and methodological problems have affected the study of *T. cruzi* population genetics, not the least of which is the continued difficulty to sample bug species and reservoir communities in conserved or partially modified habitats, across regions, or the continent. Previous studies provide evidence that hosts do not circulate all parasite populations all the time, in addition to segregation of infra-populations in different tissues and blood [16, 25, 27]. These studies clearly demonstrate that in vitro or in vivo passage of parasite populations further selects for haplotypes, which bias interpretation of genetic diversity [61, 62]. Despite improvement of molecular detection sensitivity and specificity, high polymorphism in primer binding sequences continues to affect parasite detection and hence operative needs for efficient patient and healthcare management. We assume that these gene markers and methods afford equal probability to detect all DTUs when present, but there is no evidence that this may be correct. Few population-based studies have appropriate sampling design and or subpopulation representation, and in order to analyze existing evidence for DTU host specificity and geographical or landscape associations, most studies not analyzing original samples, cannot be used. The present re-analysis uses three robust methods to synthesize and evaluate current evidence related to *T. cruzi* diversity based on DTU or the two existing lineage schemes: similarity of environmental/geographical associations (abiotic ecological niche), biotic niche structure (beta specificity), and mammal as well as vector host associations (frequency analysis). Results from this re-analysis have been contrasted with conclusions regarding associations from individual studies.

DTUIV (and subclades III and I) and DTUII (and subclades V and VI) are primary phylogenetic branches, both reported frequently in non-human Primates [18]. However, while DTUIV was reported significantly in Carnivora, DTUII is reported significantly more in Chiroptera. Since we assume in this analysis that the methodological probability of identifying all DTUs is similar for all taxa, reservoir, or geographical heterogeneity, and considering the reduced number of samples analyzed, differential DTU distributions may be due to historical access, bioclimatic niche, or host specificity. In terms of vectors, DTUII was frequently reported in *Triatoma*, while there was no significant association of DTUIV with any vector genera. Current data suggest a high frequency of DTUIV in human oral transmission, although more robust evidence will be required to analyze potential specificity of its transmission via this mechanism [63, 64].

Subclades DTUIII and I are derived from DTUIV, the former recorded from many mammal orders (5), although significant only with Cingulata (i.e. armadillos), grassland and tropical forest insectivores and one of the most important burrow excavating mammal groups in tropical regions [4]. Interestingly, DTUIII was frequently reported only from *Triatoma* species, which share similar terrestrial habitats. Similar DTU associations would be expected for *Panstrongylus* species, since they have been assumed to be vectors for *T. cruzi* transmission to humans currently in Brazil [65]. However, based on current evidence, the present analysis does not support any specific or significant associations of any DTU with this vector genus, and its importance for human infections will require future population genetics analyses which include vectors and all taxa. Despite previous suggestions of DTUIII having broad geographical distribution and spatial structuring specifically in Cingulata across South America, this was not supported by existing evidence across the continent [66, 67].

Although early studies suggested DTUI (lineage LI) specificity for Didelphimorphia [66], this relationship is not exclusive, since it is the most generalist DTU, having been recorded in seven mammal orders, and is found significantly more in small and medium-sized mammals such as Rodentia, Didelphimorphia and Primates (including humans) [66, 68]. Curiously, vector host interaction frequencies are significant for DTUI only with the vector genus *Rhodnius*, important organisms along with Rodentia, Didelphimorphia and birds, in palm communities [66]. The lack of significant association of the genus *Triatoma* with DTUI is surprising, but cannot be spurious, especially given its distribution across the continent and number of samples reported. The current analysis of beta specificity does not allow us to ascertain whether spill-over of the DTUI is other than a “permissive” event, given the generalist character of most *Triatoma* species. In fact, the spill-over could have occurred from *Triatoma* to *Rhodnius*, but current evidence justifies less this hypothesis than a direct relationship with the latter genus. This DTU’s specificity with the diverse genus *Rhodnius* may have been a key mechanism for its dispersal, both north Central America and North America, and south from its principal distribution in the north of South America, and diversification [69, 70]. Overlapped ranges between *Rhodnius* spp. and the *Triatoma dimidiata* complex in Colombia, Venezuela, French Guyana, Ecuador and Peru, north through Central America to the Neotropical region of Mexico (at least up to the Tehuantepec Isthmus) in medium forest ecotopes, may have provided novel dispersal and diversification opportunities for DTUI to the north [68]. The Mesoamerican biodiversity corridor and regional indigenous human interchange (Mesoamerican and Andean cultures) have provided ample opportunity for movement of DTUI through the *dimidiata* complex species, through humans and additional hosts throughout the Neotropical region north of the Amazon basin. North of the Tehuantepec Isthmus (in Mexico), the distribution range of *dimidiata* complex overlaps with most *phyllosoma* species complex [71], thus providing a spatial network of ecological connectivity between the vectors as well as with other vector complexes (e.g. *protracta*, *rubida* and *lecticularia*) (Ibarra-Cerdeña, personal communication). This sympatry may have provided additional mechanisms for northward dispersal of DTUI, an important strategy when there is high regional species turnover. Similar mechanisms may have driven southward dispersal where *Rhodnius* species are sympatric with the *Triatoma brasiliensis* complex, *T. sordida* in the *infestans* complex, and multiple *Panstrongylus* species [72]. The frequency of DTUI in humans may, however, be related to methodological bias, but given altered reservoir communities in human-modified habitats, its frequency may also be related to the human footprint and mobility in and among modified reservoir communities. A much more specialized analysis of DTUI diversity would need to accompany a more specific sampling effort across regions with or without sympatric *Rhodnius* to test a relevant hypothesis. Future studies should be designed to generate appropriate evidence for the impact of landscape type and modification on this DTU’s transmission. Recently, a new clade designated Tcbat, genetically more closely related to TcI than to any other DTU, was described [73]. Some authors have recently proposed that Tcbat be recognized as an additional DTU within *T. cruzi* [74, 75], even though there are too few results from Tcbat to confirm or reject its validity [76]. Association of this clade with specific hosts or vectors will have to await more appropriate gene markers, since it is poorly recognized by existing techniques, and at least in the Neotropical region of Mexico, it has not been identified using the 18SrRNA (Izeta, personal communication).

The DTUII/V/VI group has the highest relative mammal host dissimilarity due to nestedness, indicating that host communities across regions represent non-random subsets of highly diverse successive assemblages. DTU beta-diversity was generally high, also indicating highly diverse host assemblages across regions. Previously proposed geographical restrictions for certain lineages and these DTUs are not supported by re-analysis of current evidence, despite potential host specificities (for DTUIII and DTUVI) [11, 19, 20]. Host dissimilarity was high across the continent (80–100%) principally due to host species turnover, and to a much lesser degree than to nestedness.

Host-DTU specificities coincide closely with parasite phylogeny, and not with aggregated lineage level clades [14, 18]. Significant frequencies for individual DTUs are lost when they are combined as Lineage II/L1. However, significant frequencies of combined DTUs III and IV in Lineage III/L2 maintain significance. Highest DTU diversity is observed in the higher mammal trophic orders (Carnivora, Primates). Orders inhabiting upper canopy and having long distance dispersal such as Chiroptera and non-human Primates, significantly concentrate DTUII, DTUVI and DTUI. DTUIII, although only significant in the insectivorous Cingulata, is recorded in Carnivora and other lower trophic level orders (Rodentia, Didelphimorphia), perhaps due to food chain interactions, or shared nesting habits. DTUI is the dominant subclade in lower trophic level taxa.

In general, there were no significant niche differences among DTUs (except between DTUIV and VI), or lineage schemes, at coarse spatial scales. This is similar to that recently proposed at the landscape level [77, 78], rather than the long-standing hypothesis of host or geographical segregation [11]. The present study has analyzed 2,377 data records from the continent from all *T. cruzi* hosts, using robust modeling methods, and found that despite greater frequency of DTUI reports, which may be related to detection and isolation methods, its potential distribution is majorly sympatric with other DTUs. Given the high significance of niche models included in this study, the Amazon headwater region and few sparse areas of Central America predict low niche for the DTUII/V/VI, while both regions predict high presence for DTUIV/III/I. The former group may have extended associations with hosts where other DTUs are not present, outside certain latitudinal limits (in northern Mexico, USA and central Argentina). Given that the majority of studies have used selected parasite isolates, it is not possible to discern whether reporting differences are due to methodological bias, or due to real molecular differences in DTU presence or abundance. As an example for the former, the first study using parasite isolates and MLEP in Mexico found greater than 98% Lineage I [79], while recent genetic marker studies using DNA amplification directly from host tissues finds an equal proportion of DTUVI at least in the Neotropical region in Mexico [80].

A key issue that current public health programs must address to limit vector-borne transmission of *T. cruzi* is the source of parasite populations that are in contact with humans. Human contact with infected vectors not only occurs in domestic areas, but in all fragments, with broad vector gene flow within landscapes [80, 81]. Metapopulation dynamics is assisted by human activity and by its impact on resource availability temporally and spatially, although also by altered reservoir assemblages in different landscape fragments [82, 83]. Vector interactions and their contact with numerous host/reservoir communities also affect parasite metapopulation dynamics in humans and domesticated fauna (livestock and pets). There is insufficient evidence currently, in any landscape to understand microevolutionary processes such as gene flow, gene drift, or parasite selection on a local scale [81, 84]. It is hoped that recognizing the void and the importance of this evidence will motivate more robust analyses. Without this evidence, sustainable transmission barriers will lack an evidence base.

There are many studies using assemblages of *T. cruzi* samples collected with different purposes in different regions (commonly by different groups of researchers or health system personnel). The samples are often not collected using specific sampling design and a hypothesis testing framework (i.e. without proper replicates or a common sampling design which affects internal and external validity of designs), yet analyzed and interpreted as though they had*.* Although these contributions are important for certain genetic information, they are affected by statistical noise that masks the nature of the process behind data, leading to flawed conclusions regarding parasite population patterns [62]. It is clear that in order to understand *T. cruzi* metapopulation dynamics and microevolutionary patterns, more appropriate gene markers and methods are necessary, including those that can be used for sylvatic reservoirs, humans and livestock/pets, as well as all triatomine vectors.

## Conclusions

In order to analyze DTU associations with host taxa or geography, a greater number of samples and quantity of DNA is required, which highlights the need for assays with better sensitivity [35, 85, 86]. Marker design and selection, amplification methods, and potential non-specific amplification (including other trypanosome species) need to be tested for broad use and genotyping of parasite clades in order to analyze metapopulation dynamics [87]. Technological advances are urgently needed in this area, since analyses of *T. cruzi* landscape genetics will continue to be partial and biased unless we can confidently identify and analyze metapopulations from all hosts in assemblages over distribution ranges and according to ecological interactions and temporal dynamics.

## Additional files

Additional file 1: Table S1. Data used for re-analysis (some species are not correctly specified in original articles and are georeferenced according to the locality or community). (DOCX 489 kb)
Additional file 2: Table S2. Phylogenetic studies not using analytical methods. (DOCX 30 kb)
Additional file 3: Table S3. Phylogeographic studies not using analytical methods. (DOCX 15 kb)
Additional file 4: Table S4. Number of species and records reported in different orders. (DOCX 16 kb)
Additional file 5: Figure S1. Ecological niche significance values. (TIF 40 kb)

## Abbreviations

AUC: area under the ROC
CA: Central America
CD: Chagas disease
CI: confidence interval
DALYs: disability-adjusted life-years
DNAsat: satellite DNA
DTU: discrete typing unit
ENM: ecological niche models
kDNA: kinetoplast DNA
LSSP: low stringency single specific primer
MLEE: multi-locus enzyme electrophoresis
NA: North America
RAPD: random amplified polymorphic DNA
rDNA: ribosomal DNA
rtPCR: real time PCR
RFLP: restriction fragment length polymorphisms
ROC: receiver operating curve
SNPs: single nucleotide polymorphisms
SA: South America
Tc: *Trypanosoma cruzi*
