# Supplementary material for: Geographical, landscape and host associations of Trypanosoma cruzi DTUs and lineages
Source: Parasit Vectors. 2016 Dec 7;9:631. doi: 10.1186/s13071-016-1918-2 (PMC5142175; doi:10.1186/s13071-016-1918-2)
Supplement: Additional file 1: Table S1. — Data used for re-analysis (some species are not correctly specified in original articles and are georeferenced according to the locality or community). (DOCX 489 kb) [file 13071_2016_1918_MOESM1_ESM.docx]

Table S1. Data used for re-analysis

| **Taxa** | **Class** | **Order** | ***T.cruzi* DTU** | **Latitude** | **Longitude** | **Author** | **Source** | **Georeference*** | **L1** | **L2** |
| --- | --- | --- | --- | --- | --- | --- | --- | --- | --- | --- |
| *Akodon boliviensis* | Host | Rodentia | DTUI | -17.43000000 | -66.27000000 | Llewellyn et al. 2009b | Author | NA | I | I |
| *Akodon boliviensis* | Host | Rodentia | DTUI | -17.43000000 | -66.27000000 | Llewellyn et al. 2009b | Author | NA | I | I |
| *Akodon boliviensis* | Host | Rodentia | DTUI | -17.43000000 | -66.27000000 | Messenger et al. 2012 | Author | NA | I | I |
| *Alouatta spp* | Host | Primates | DTUI | 5.30000000 | -72.40000000 | Ramirez et al. 2013 | Author | NA | I | I |
| *Aotus sp* | Host | Primates | DTUI | -27.03300000 | -48.48300000 | Marcili et al. 2009b | Google earth | Locality | I | I |
| *Aotus sp* | Host | Primates | DTUIV | -27.03300000 | -48.48300000 | Marcili et al. 2009b | Google earth | Locality | II | III |
| *Artibeus fimbriatus* | Host | Chiroptera | DTUI | -0.08300 | -48.90000 | Lisboa et al. 2008 | Google earth | Biomas Brasil | I | I |
| *Artibeus jamaicensis* | Host | Chiroptera | DTUI | 8.58300000 | -63.95000000 | Carrasco et al. 2012 | Google earth | State | I | I |
| *Artibeus jamaicensis* | Host | Chiroptera | ND | -19.23400 | -57.01600 | Lisboa et al. 2008 | Google earth | Biomas Brasil | II | ND |
| *Artibeus lituratus* | Host | Chiroptera | DTUVI | 18.59570338 | -89.41598419 | Lopez-Cancino et al. 2015 | Author | NA | II | ND |
| *Artibeus planirostris* | Host | Chiroptera | DTUI | -4.0925 | -75.1544 | Lima et al. 2015 | Google earth | State | I | I |
| *Canis lupus familiaris* | Host | Carnivora | DTUI | 10.95000000 | -73.46700000 | Rodriguez et al. 2009 | Google earth | Area/Unspecified communities | I | I |
| *Canis lupus familiaris* | Host | Carnivora | DTUI | 10.95000000 | -73.46700000 | Rodriguez et al. 2009 | Google earth | Area/Unspecified communities | I | I |
| *Canis lupus familiaris* | Host | Carnivora | DTUI | 10.95000000 | -73.46700000 | Rodriguez et al. 2009 | Google earth | Area/Unspecified communities | I | I |
| *Canis lupus familiaris* | Host | Carnivora | DTUI | 10.95000000 | -73.46700000 | Rodriguez et al. 2009 | Google earth | Area/Unspecified communities | I | I |
| *Canis lupus familiaris* | Host | Carnivora | DTUI | 10.95000000 | -73.46700000 | Rodriguez et al. 2009 | Google earth | Area/Unspecified communities | I | I |
| *Canis lupus familiaris* | Host | Carnivora | DTUI | 10.95000000 | -73.46700000 | Rodriguez et al. 2009 | Google earth | Area/Unspecified communities | I | I |
| *Canis lupus familiaris* | Host | Carnivora | DTUI | 10.95000000 | -73.46700000 | Rodriguez et al. 2009 | Google earth | Area/Unspecified communities | I | I |
| *Canis lupus familiaris* | Host | Carnivora | DTUI | 10.95000000 | -73.46700000 | Rodriguez et al. 2009 | Google earth | Area/Unspecified communities | I | I |
| *Canis lupus familiaris* | Host | Carnivora | DTUI | 10.95000000 | -73.46700000 | Rodriguez et al. 2009 | Google earth | Area/Unspecified communities | I | I |
| *Canis lupus familiaris* | Host | Carnivora | DTUI | 10.95000000 | -73.46700000 | Rodriguez et al. 2009 | Google earth | Area/Unspecified communities | I | I |
| *Canis lupus familiaris* | Host | Carnivora | DTUI | 10.95000000 | -73.46700000 | Rodriguez et al. 2009 | Google earth | Area/Unspecified communities | I | I |
| *Canis lupus familiaris* | Host | Carnivora | DTUI | 10.95000000 | -73.46700000 | Rodriguez et al. 2009 | Google earth | Area/Unspecified communities | I | I |
| *Canis lupus familiaris* | Host | Carnivora | DTUI | 10.95000000 | -73.46700000 | Rodriguez et al. 2009 | Google earth | Area/Unspecified communities | I | I |
| *Canis lupus familiaris* | Host | Carnivora | DTUI | 10.95000000 | -73.46700000 | Rodriguez et al. 2009 | Google earth | Area/Unspecified communities | I | I |
| *Canis lupus familiaris* | Host | Carnivora | DTUI | 10.95000000 | -73.46700000 | Rodriguez et al. 2009 | Google earth | Area/Unspecified communities | I | I |
| *Canis lupus familiaris* | Host | Carnivora | DTUI | 10.95000000 | -73.46700000 | Rodriguez et al. 2009 | Google earth | Area/Unspecified communities | I | I |
| *Canis lupus familiaris* | Host | Carnivora | DTUI | 10.95000000 | -73.46700000 | Rodriguez et al. 2009 | Google earth | Area/Unspecified communities | I | I |
| *Canis lupus familiaris* | Host | Carnivora | DTUI | 10.95000000 | -73.46700000 | Rodriguez et al. 2009 | Google earth | Area/Unspecified communities | I | I |
| *Canis lupus familiaris* | Host | Carnivora | DTUI | 10.95000000 | -73.46700000 | Rodriguez et al. 2009 | Google earth | Area/Unspecified communities | I | I |
| *Canis lupus familiaris* | Host | Carnivora | DTUI | 10.95000000 | -73.46700000 | Rodriguez et al. 2009 | Google earth | Area/Unspecified communities | I | I |
| *Canis lupus familiaris* | Host | Carnivora | DTUI | 10.95000000 | -73.46700000 | Rodriguez et al. 2009 | Google earth | Area/Unspecified communities | I | I |
| *Canis lupus familiaris* | Host | Carnivora | DTUI | 10.95000000 | -73.46700000 | Rodriguez et al. 2009 | Google earth | Area/Unspecified communities | I | I |
| *Canis lupus familiaris* | Host | Carnivora | DTUI | 10.95000000 | -73.46700000 | Rodriguez et al. 2009 | Google earth | Area/Unspecified communities | I | I |
| *Canis lupus familiaris* | Host | Carnivora | DTUI | 10.95000000 | -73.46700000 | Rodriguez et al. 2009 | Google earth | Area/Unspecified communities | I | I |
| *Canis lupus familiaris* | Host | Carnivora | DTUI | 10.95000000 | -73.46700000 | Rodriguez et al. 2009 | Google earth | Area/Unspecified communities | I | I |
| *Canis lupus familiaris* | Host | Carnivora | DTUI | 10.95000000 | -73.46700000 | Rodriguez et al. 2009 | Google earth | Area/Unspecified communities | I | I |
| *Canis lupus familiaris* | Host | Carnivora | DTUI | 5.10000000 | -71.60000000 | Ramirez et al. 2013 | Author | NA | I | I |
| *Canis lupus familiaris* | Host | Carnivora | DTUI | 7.18300000 | -75.03300000 | Mejia-Jaramillo et al. 2009 | Google earth | Department/State | I | I |
| *Canis lupus familiaris* | Host | Carnivora | DTUI | 9.08300000 | -69.08300000 | Carrasco et al. 2012 | Google earth | State | I | I |
| *Canis lupus familiaris* | Host | Carnivora | DTUI | 20.98138889 | -89.59250000 | Cura et al. 2015 | Google earth | Locality | I | I |
| *Canis lupus familiaris* | Host | Carnivora | DTUI | 20.98138889 | -89.59250000 | Cura et al. 2015 | Google earth | Locality | I | I |
| *Canis lupus familiaris* | Host | Carnivora | DTUI | 20.98138889 | -89.59250000 | Cura et al. 2015 | Google earth | Locality | I | I |
| *Canis lupus familiaris* | Host | Carnivora | DTUI | 20.98138889 | -89.59250000 | Cura et al. 2015 | Google earth | Locality | I | I |
| *Canis lupus familiaris* | Host | Carnivora | DTUIII | 4.88250000 | -52.28666667 | Cura et al. 2015 | Google earth | Locality | II | III |
| *Canis lupus familiaris* | Host | Carnivora | DTUIII | -27.42555556 | -59.02416667 | Cura et al. 2015 | Google earth | Area/Unspecified communities | II | III |
| *Canis lupus familiaris* | Host | Carnivora | DTUIII | -27.42555556 | -59.02416667 | Cura et al. 2015 | Google earth | Area/Unspecified communities | II | III |
| *Canis lupus familiaris* | Host | Carnivora | DTUIII | -25.08333333 | -56.96666667 | Enriquez et al. 2014 | Google earth | Locality | II | III |
| *Canis lupus familiaris* | Host | Carnivora | DTUIII | -25.08333333 | -56.96666667 | Enriquez et al. 2014 | Google earth | Locality | II | III |
| *Canis lupus familiaris* | Host | Carnivora | DTUIV | 35.40000000 | -85.96700000 | Roellig et al. 2013 | Google earth | Community | II | III |
| *Canis lupus familiaris* | Host | Carnivora | DTUIV | 36.73300000 | -95.96700000 | Roellig et al. 2013 | Google earth | Community | II | III |
| *Canis lupus familiaris* | Host | Carnivora | DTUIV | 33.08300000 | -81.15000000 | Roellig et al. 2013 | Google earth | Community | II | III |
| *Canis lupus familiaris* | Host | Carnivora | DTUIV | 33.08300000 | -81.15000000 | Roellig et al. 2013 | Google earth | Community | II | III |
| *Canis lupus familiaris* | Host | Carnivora | DTUIV | 36.76600000 | -119.41560000 | Roellig et al. 2013 | Google earth | Community | II | III |
| *Canis lupus familiaris* | Host | Carnivora | DTUV | -25.08333333 | -56.96666667 | Enriquez et al. 2014 | Google earth | Locality | II | ND |
| *Canis lupus familiaris* | Host | Carnivora | DTUV | -25.08333333 | -56.96666667 | Enriquez et al. 2014 | Google earth | Locality | II | ND |
| *Canis lupus familiaris* | Host | Carnivora | DTUV | -25.08333333 | -56.96666667 | Enriquez et al. 2014 | Google earth | Locality | II | ND |
| *Canis lupus familiaris* | Host | Carnivora | DTUV | -25.08333333 | -56.96666667 | Enriquez et al. 2014 | Google earth | Locality | II | ND |
| *Canis lupus familiaris* | Host | Carnivora | DTUV | -25.08333333 | -56.96666667 | Enriquez et al. 2014 | Google earth | Locality | II | ND |
| *Canis lupus familiaris* | Host | Carnivora | DTUVI | -26.58300000 | -60.95000000 | Lauthier et al. 2012 | Google earth | Province | II | ND |
| *Canis lupus familiaris* | Host | Carnivora | DTUVI | -26.58300000 | -60.95000000 | Lauthier et al. 2012 | Google earth | Province | II | ND |
| *Canis lupus familiaris* | Host | Carnivora | DTUVI | -26.58300000 | -60.95000000 | Lauthier et al. 2012 | Google earth | Province | II | ND |
| *Canis lupus familiaris* | Host | Carnivora | DTUVI | -26.58300000 | -60.95000000 | Lauthier et al. 2012 | Google earth | Province | II | ND |
| *Canis lupus familiaris* | Host | Carnivora | DTUVI | 18.59040361 | -89.41808771 | Lopez-Cancino et al. 2015 | Author | NA | II | ND |
| *Canis lupus familiaris* | Host | Carnivora | DTUVI | 18.59040362 | -89.41808771 | Lopez-Cancino et al. 2015 | Author | NA | II | ND |
| *Canis lupus familiaris* | Host | Carnivora | DTUVI | 18.59040363 | -89.41808771 | Lopez-Cancino et al. 2015 | Author | NA | II | ND |
| *Canis lupus familiaris* | Host | Carnivora | DTUVI | 18.59000855 | -89.41777892 | Lopez-Cancino et al. 2015 | Author | NA | II | ND |
| *Canis lupus familiaris* | Host | Carnivora | DTUVI | 18.59074398 | -89.41760575 | Lopez-Cancino et al. 2015 | Author | NA | II | ND |
| *Canis lupus familiaris* | Host | Carnivora | DTUVI | 18.59305001 | -89.41725254 | Lopez-Cancino et al. 2015 | Author | NA | II | ND |
| *Canis lupus familiaris* | Host | Carnivora | DTUVI | 18.59182063 | -89.41638242 | Lopez-Cancino et al. 2015 | Author | NA | II | ND |
| *Canis lupus familiaris* | Host | Carnivora | DTUVI | 18.59182063 | -89.41638242 | Lopez-Cancino et al. 2015 | Author | NA | II | ND |
| *Canis lupus familiaris* | Host | Carnivora | DTUVI | -27.42555556 | -59.02416667 | Cura et al. 2015 | Google earth | Area/Unspecified communities | II | ND |
| *Canis lupus familiaris* | Host | Carnivora | DTUVI | -27.42555556 | -59.02416667 | Cura et al. 2015 | Google earth | Area/Unspecified communities | II | ND |
| *Canis lupus familiaris* | Host | Carnivora | DTUVI | -27.42555556 | -59.02416667 | Cura et al. 2015 | Google earth | Area/Unspecified communities | II | ND |
| *Canis lupus familiaris* | Host | Carnivora | DTUVI | -27.42555556 | -59.02416667 | Cura et al. 2015 | Google earth | Area/Unspecified communities | II | ND |
| *Canis lupus familiaris* | Host | Carnivora | DTUVI | -27.42555556 | -59.02416667 | Cura et al. 2015 | Google earth | Area/Unspecified communities | II | ND |
| *Canis lupus familiaris* | Host | Carnivora | DTUVI | -27.42555556 | -59.02416667 | Cura et al. 2015 | Google earth | Area/Unspecified communities | II | ND |
| *Canis lupus familiaris* | Host | Carnivora | DTUVI | -25.08333333 | -56.96666667 | Enriquez et al. 2014 | Google earth | Locality | II | ND |
| *Canis lupus familiaris* | Host | Carnivora | DTUVI | -25.08333333 | -56.96666667 | Enriquez et al. 2014 | Google earth | Locality | II | ND |
| *Canis lupus familiaris* | Host | Carnivora | DTUVI | -25.08333333 | -56.96666667 | Enriquez et al. 2014 | Google earth | Locality | II | ND |
| *Canis lupus familiaris* | Host | Carnivora | DTUVI | -25.08333333 | -56.96666667 | Enriquez et al. 2014 | Google earth | Locality | II | ND |
| *Canis lupus familiaris* | Host | Carnivora | DTUVI | -25.08333333 | -56.96666667 | Enriquez et al. 2014 | Google earth | Locality | II | ND |
| *Canis lupus familiaris* | Host | Carnivora | DTUVI | -25.08333333 | -56.96666667 | Enriquez et al. 2014 | Google earth | Locality | II | ND |
| *Canis lupus familiaris* | Host | Carnivora | DTUVI | -25.08333333 | -56.96666667 | Enriquez et al. 2014 | Google earth | Locality | II | ND |
| *Canis lupus familiaris* | Host | Carnivora | DTUVI | -25.08333333 | -56.96666667 | Enriquez et al. 2014 | Google earth | Locality | II | ND |
| *Canis lupus familiaris* | Host | Carnivora | DTUVI | -25.08333333 | -56.96666667 | Enriquez et al. 2014 | Google earth | Locality | II | ND |
| *Canis lupus familiaris* | Host | Carnivora | DTUVI | -25.08333333 | -56.96666667 | Enriquez et al. 2014 | Google earth | Locality | II | ND |
| *Canis lupus familiaris* | Host | Carnivora | DTUVI | -25.08333333 | -56.96666667 | Enriquez et al. 2014 | Google earth | Locality | II | ND |
| *Canis lupus familiaris* | Host | Carnivora | DTUVI | -25.08333333 | -56.96666667 | Enriquez et al. 2014 | Google earth | Locality | II | ND |
| *Canis lupus familiaris* | Host | Carnivora | DTUVI | -25.08333333 | -56.96666667 | Enriquez et al. 2014 | Google earth | Locality | II | ND |
| *Canis lupus familiaris* | Host | Carnivora | DTUVI | -25.08333333 | -56.96666667 | Enriquez et al. 2014 | Google earth | Locality | II | ND |
| *Canis lupus familiaris* | Host | Carnivora | DTUVI | -25.08333333 | -56.96666667 | Enriquez et al. 2014 | Google earth | Locality | II | ND |
| *Canis lupus familiaris* | Host | Carnivora | DTUVI | -25.08333333 | -56.96666667 | Enriquez et al. 2014 | Google earth | Locality | II | ND |
| *Canis lupus familiaris* | Host | Carnivora | DTUVI | -25.08333333 | -56.96666667 | Enriquez et al. 2014 | Google earth | Locality | II | ND |
| *Canis lupus familiaris* | Host | Carnivora | DTUVI | -25.08333333 | -56.96666667 | Enriquez et al. 2014 | Google earth | Locality | II | ND |
| *Canis lupus familiaris* | Host | Carnivora | DTUVI | -25.08333333 | -56.96666667 | Enriquez et al. 2014 | Google earth | Locality | II | ND |
| *Canis lupus familiaris* | Host | Carnivora | DTUVI | -25.08333333 | -56.96666667 | Enriquez et al. 2014 | Google earth | Locality | II | ND |
| *Canis lupus familiaris* | Host | Carnivora | DTUVI | -25.08333333 | -56.96666667 | Enriquez et al. 2014 | Google earth | Locality | II | ND |
| *Canis lupus familiaris* | Host | Carnivora | DTUVI | -25.08333333 | -56.96666667 | Enriquez et al. 2014 | Google earth | Locality | II | ND |
| *Canis lupus familiaris* | Host | Carnivora | DTUVI | -25.08333333 | -56.96666667 | Enriquez et al. 2014 | Google earth | Locality | II | ND |
| *Canis lupus familiaris* | Host | Carnivora | DTUVI | -25.08333333 | -56.96666667 | Enriquez et al. 2014 | Google earth | Locality | II | ND |
| *Canis lupus familiaris* | Host | Carnivora | DTUVI | -25.08333333 | -56.96666667 | Enriquez et al. 2014 | Google earth | Locality | II | ND |
| *Canis lupus familiaris* | Host | Carnivora | DTUVI | -25.08333333 | -56.96666667 | Enriquez et al. 2014 | Google earth | Locality | II | ND |
| *Canis lupus familiaris* | Host | Carnivora | DTUVI | -25.08333333 | -56.96666667 | Enriquez et al. 2014 | Google earth | Locality | II | ND |
| *Canis lupus familiaris* | Host | Carnivora | DTUVI | -25.08333333 | -56.96666667 | Enriquez et al. 2014 | Google earth | Locality | II | ND |
| *Canis lupus familiaris* | Host | Carnivora | DTUVI | -25.08333333 | -56.96666667 | Enriquez et al. 2014 | Google earth | Locality | II | ND |
| *Canis lupus familiaris* | Host | Carnivora | DTUVI | -25.08333333 | -56.96666667 | Enriquez et al. 2014 | Google earth | Locality | II | ND |
| *Canis lupus familiaris* | Host | Carnivora | DTUVI | -25.08333333 | -56.96666667 | Enriquez et al. 2014 | Google earth | Locality | II | ND |
| *Canis lupus familiaris* | Host | Carnivora | DTUVI | -25.08333333 | -56.96666667 | Enriquez et al. 2014 | Google earth | Locality | II | ND |
| *Canis lupus familiaris* | Host | Carnivora | DTUVI | -25.08333333 | -56.96666667 | Enriquez et al. 2014 | Google earth | Locality | II | ND |
| *Canis lupus familiaris* | Host | Carnivora | DTUVI | -25.08333333 | -56.96666667 | Enriquez et al. 2014 | Google earth | Locality | II | ND |
| *Canis lupus familiaris* | Host | Carnivora | DTUVI | -25.08333333 | -56.96666667 | Enriquez et al. 2014 | Google earth | Locality | II | ND |
| *Canis lupus familiaris* | Host | Carnivora | DTUVI | -25.08333333 | -56.96666667 | Enriquez et al. 2014 | Google earth | Locality | II | ND |
| *Canis lupus familiaris* | Host | Carnivora | DTUVI | -25.08333333 | -56.96666667 | Enriquez et al. 2014 | Google earth | Locality | II | ND |
| *Carollia perspicillata* | Host | Chiroptera | DTUI | -11.505833 | -63.58056 | Marcili et al. 2009a | Google earth | State | I | I |
| *Carollia perspicillata* | Host | Chiroptera | DTUI | -11.505834 | -63.58056 | Marcili et al. 2009a | Google earth | State | I | I |
| *Carollia perspicillata* | Host | Chiroptera | DTUI | -11.505835 | -63.58056 | Marcili et al. 2009a | Google earth | State | I | I |
| *Carollia perspicillata* | Host | Chiroptera | DTUII | -11,505833 | -63,58056 | Lima et al. 2015 | Google earth | State | II | II |
| *Cavia porcellus* | Host | Rodentia | DTUI | -16.408889 | -71.537222 | Cura et al. 2015 | Google earth | Area/Unspecified communities | I | I |
| *Cebuella pygmea* | Host | Primates | DTUI | -9.96600000 | -67.81600000 | Marcili et al. 2009b | Google earth | Locality | I | I |
| *Cebus albifrons* | Host | Primates | DTUIV | -0.26700000 | -63.20000000 | Marcili et al. 2009b | Google earth | Locality | II | III |
| *Cebus apella* | Host | Primates | DTUII | -22.906944 | -43.173056 | Araujo et al. 2011 | Google earth | Locality | II | II |
| *Cebus apella* | Host | Primates | DTUI | -9.96700000 | -67.81600000 | Marcili et al. 2009b | Google earth | Locality | I | I |
| *Chaetophractus vellorosus* | Host | Cingulata | DTUIII | -19.21000000 | -63.43000000 | Llewellyn et al. 2009a | Author | NA | II | III |
| *Chaetophractus vellorosus* | Host | Cingulata | DTUIII | -22.33000000 | -58.93000000 | Llewellyn et al. 2009a | Author | NA | II | III |
| *Conepatus leuconotus* | Host | Carnivora | DTUIV | 31.70000000 | -81.73400000 | Roellig et al. 2013 | Google earth | Community | II | III |
| *Dasyprocta fugilinosa* | Host | Rodentia | DTUIII | 3.30000000 | -73.00000000 | Llewellyn et al. 2009a | Author | NA | II | III |
| *Dasypus novemcinctus* | Host | Cingulata | DTUI | 29.95000000 | -90.06700000 | Roellig et al. 2013 | Google earth | Community | I | I |
| *Dasypus novemcinctus* | Host | Cingulata | DTUIV | 31.75000000 | -81.10000000 | Roellig et al. 2013 | Google earth | Community | II | III |
| *Dasypus novemcinctus* | Host | Cingulata | DTUI | 30.50000000 | -91.00000000 | Llewellyn et al. 2009b | Author | NA | I | I |
| *Dasypus novemcinctus* | Host | Cingulata | DTUI | 30.50000000 | -91.00000000 | Messenger et al. 2012 | Author | NA | I | I |
| *Dasypus novemcinctus* | Host | Cingulata | DTUIII | -15.50000000 | -67.50000000 | Llewellyn et al. 2009a | Author | NA | II | III |
| *Dasypus novemcinctus* | Host | Cingulata | DTUIII | -14.81000000 | -64.60000000 | Llewellyn et al. 2009a | Author | NA | II | III |
| *Dasypus novemcinctus* | Host | Cingulata | DTUIII | -14.81000000 | -64.60000000 | Llewellyn et al. 2009a | Author | NA | II | III |
| *Dasypus novemcinctus* | Host | Cingulata | DTUIII | -15.12000000 | -64.32000000 | Llewellyn et al. 2009a | Author | NA | II | III |
| *Dasypus novemcinctus* | Host | Cingulata | DTUIII | -14.13000000 | -65.36000000 | Llewellyn et al. 2009a | Author | NA | II | III |
| *Dasypus novemcinctus* | Host | Cingulata | DTUIII | -14.13000000 | -65.36000000 | Llewellyn et al. 2009a | Author | NA | II | III |
| *Dasypus novemcinctus* | Host | Cingulata | DTUIII | -14.13000000 | -65.36000000 | Llewellyn et al. 2009a | Author | NA | II | III |
| *Dasypus novemcinctus* | Host | Cingulata | DTUIII | -14.13000000 | -65.36000000 | Llewellyn et al. 2009a | Author | NA | II | III |
| *Dasypus novemcinctus* | Host | Cingulata | DTUIII | -17.50000000 | -61.50000000 | Llewellyn et al. 2009a | Author | NA | II | III |
| *Dasypus novemcinctus* | Host | Cingulata | DTUIII | -17.50000000 | -61.50000000 | Llewellyn et al. 2009a | Author | NA | II | III |
| *Dasypus novemcinctus* | Host | Cingulata | DTUIII | -17.50000000 | -61.50000000 | Llewellyn et al. 2009a | Author | NA | II | III |
| *Dasypus novemcinctus* | Host | Cingulata | DTUIII | -17.50000000 | -61.50000000 | Llewellyn et al. 2009a | Author | NA | II | III |
| *Dasypus novemcinctus* | Host | Cingulata | DTUIII | -17.50000000 | -61.50000000 | Llewellyn et al. 2009a | Author | NA | II | III |
| *Dasypus novemcinctus* | Host | Cingulata | DTUIII | -17.50000000 | -61.50000000 | Llewellyn et al. 2009a | Author | NA | II | III |
| *Dasypus novemcinctus* | Host | Cingulata | DTUIII | -19.21000000 | -63.43000000 | Llewellyn et al. 2009a | Author | NA | II | III |
| *Dasypus novemcinctus* | Host | Cingulata | DTUIII | -19.21000000 | -63.43000000 | Llewellyn et al. 2009a | Author | NA | II | III |
| *Dasypus novemcinctus* | Host | Cingulata | DTUIII | -19.21000000 | -63.43000000 | Llewellyn et al. 2009a | Author | NA | II | III |
| *Dasypus novemcinctus* | Host | Cingulata | DTUIII | -19.21000000 | -63.43000000 | Llewellyn et al. 2009a | Author | NA | II | III |
| *Dasypus novemcinctus* | Host | Cingulata | DTUIII | -20.02000000 | -63.02000000 | Llewellyn et al. 2009a | Author | NA | II | III |
| *Dasypus novemcinctus* | Host | Cingulata | DTUIII | 8.48000000 | -70.73000000 | Llewellyn et al. 2009a | Author | NA | II | III |
| *Dasypus novemcinctus* | Host | Cingulata | DTUIII | 8.48000000 | -70.73000000 | Llewellyn et al. 2009a | Author | NA | II | III |
| *Dasypus novemcinctus* | Host | Cingulata | DTUIII | -1.00000000 | -49.50000000 | Llewellyn et al. 2009a | Author | NA | II | III |
| *Dasypus novemcinctus* | Host | Cingulata | DTUIII | 8.48000000 | -70.73000000 | Llewellyn et al. 2009a | Author | NA | II | III |
| *Dasypus novemcinctus* | Host | Cingulata | DTUIII | 7.50000000 | -71.23000000 | Llewellyn et al. 2009a | Author | NA | II | III |
| *Dasypus novemcinctus* | Host | Cingulata | DTUIII | 8.43000000 | -70.55000000 | Llewellyn et al. 2009a | Author | NA | II | III |
| *Dasypus novemcinctus* | Host | Cingulata | DTUIII | 8.43000000 | -70.55000000 | Llewellyn et al. 2009a | Author | NA | II | III |
| *Dasypus novemcinctus* | Host | Cingulata | DTUIII | 8.43000000 | -70.55000000 | Llewellyn et al. 2009a | Author | NA | II | III |
| *Dasypus novemcinctus* | Host | Cingulata | DTUIII | 8.43000000 | -70.55000000 | Llewellyn et al. 2009a | Author | NA | II | III |
| *Dasypus novemcinctus* | Host | Cingulata | DTUIII | -14.81000000 | -64.60000000 | Llewellyn et al. 2009a | Author | NA | II | III |
| *Dasypus novemcinctus* | Host | Cingulata | DTUIII | -22.33000000 | -58.93000000 | Llewellyn et al. 2009a | Author | NA | II | III |
| *Dasypus novemcinctus* | Host | Cingulata | DTUIII | -22.33000000 | -58.93000000 | Llewellyn et al. 2009a | Author | NA | II | III |
| *Dasypus novemcinctus* | Host | Cingulata | DTUIII | -22.33000000 | -58.93000000 | Llewellyn et al. 2009a | Author | NA | II | III |
| *Dasypus novemcinctus* | Host | Cingulata | DTUIII | -24.00000000 | -57.00000000 | Llewellyn et al. 2009a | Author | NA | II | III |
| *Dasypus novemcinctus* | Host | Cingulata | DTUIII | -24.00000000 | -57.00000000 | Llewellyn et al. 2009a | Author | NA | II | III |
| *Dasypus novemcinctus* | Host | Cingulata | DTUIII | -24.00000000 | -57.00000000 | Llewellyn et al. 2009a | Author | NA | II | III |
| *Dasypus novemcinctus* | Host | Cingulata | DTUIII | -24.00000000 | -57.00000000 | Llewellyn et al. 2009a | Author | NA | II | III |
| *Dasypus novemcinctus* | Host | Cingulata | DTUIII | -22.33000000 | -58.93000000 | Llewellyn et al. 2009a | Author | NA | II | III |
| *Dasypus novemcinctus* | Host | Cingulata | DTUIII | -22.33000000 | -58.93000000 | Llewellyn et al. 2009a | Author | NA | II | III |
| *Dasypus novemcinctus* | Host | Cingulata | DTUIII | -24.00000000 | -57.00000000 | Llewellyn et al. 2009a | Author | NA | II | III |
| *Dasypus novemcinctus* | Host | Cingulata | DTUIII | -24.00000000 | -57.00000000 | Llewellyn et al. 2009a | Author | NA | II | III |
| *Dasypus novemcinctus* | Host | Cingulata | DTUIII | -24.00000000 | -57.00000000 | Llewellyn et al. 2009a | Author | NA | II | III |
| *Dasypus novemcinctus* | Host | Cingulata | DTUIII | -24.00000000 | -57.00000000 | Llewellyn et al. 2009a | Author | NA | II | III |
| *Dasypus novemcinctus* | Host | Cingulata | DTUIII | -27.42555556 | -59.02416667 | Cura et al. 2015 | Google earth | Area/Unspecified communities | II | III |
| *Dasypus novemcinctus* | Host | Cingulata | DTUIII | -27.42555556 | -59.02416667 | Cura et al. 2015 | Google earth | Area/Unspecified communities | II | III |
| *Dasypus novemcinctus* | Host | Cingulata | DTUIII | -27.42555556 | -59.02416667 | Cura et al. 2015 | Google earth | Area/Unspecified communities | II | III |
| *Dasypus novemcinctus* | Host | Cingulata | DTUIII | -27.42555556 | -59.02416667 | Cura et al. 2015 | Google earth | Area/Unspecified communities | II | III |
| *Dasypus novemcinctus* | Host | Cingulata | DTUIII | -27.42555556 | -59.02416667 | Cura et al. 2015 | Google earth | Area/Unspecified communities | II | III |
| *Dasypus novemcinctus* | Host | Cingulata | DTUIII | -27.42555556 | -59.02416667 | Cura et al. 2015 | Google earth | Area/Unspecified communities | II | III |
| *Dasypus novemcinctus* | Host | Cingulata | DTUIII | -27.42555556 | -59.02416667 | Cura et al. 2015 | Google earth | Area/Unspecified communities | II | III |
| *Dasypus novemcinctus* | Host | Cingulata | DTUIII | -27.42555556 | -59.02416667 | Cura et al. 2015 | Google earth | Area/Unspecified communities | II | III |
| *Dasypus novemcinctus* | Host | Cingulata | DTUIII | -27.42555556 | -59.02416667 | Cura et al. 2015 | Google earth | Area/Unspecified communities | II | III |
| *Dasypus novemcinctus* | Host | Cingulata | DTUIII | -27.42555556 | -59.02416667 | Cura et al. 2015 | Google earth | Area/Unspecified communities | II | III |
| *Dasypus novemcinctus* | Host | Cingulata | DTUIII | -27.42555556 | -59.02416667 | Cura et al. 2015 | Google earth | Area/Unspecified communities | II | III |
| *Dasypus novemcinctus* | Host | Cingulata | DTUIII | -27.42555556 | -59.02416667 | Cura et al. 2015 | Google earth | Area/Unspecified communities | II | III |
| *Dasypus novemcinctus* | Host | Cingulata | DTUIII | -27.42555556 | -59.02416667 | Cura et al. 2015 | Google earth | Area/Unspecified communities | II | III |
| *Dasypus spp* | Host | Cingulata | DTUIII | 3.30000000 | -73.00000000 | Llewellyn et al. 2009a | Author | NA | II | III |
| *Dicotyles tajacu* | Host | Artiodactyla | DTUI | 8.58300000 | -63.95000000 | Carrasco et al. 2012 | Google earth | State | I | I |
| *Didelphis albiventris* | Host | Didelphimorphia | DTUI | -26.58300000 | -60.95000000 | Lauthier et al. 2012 | Google earth | Province | I | I |
| *Didelphis albiventris* | Host | Didelphimorphia | DTUI | -26.58300000 | -60.95000000 | Lauthier et al. 2012 | Google earth | Province | I | I |
| *Didelphis albiventris* | Host | Didelphimorphia | DTUI | -26.58300000 | -60.95000000 | Lauthier et al. 2012 | Google earth | Province | I | I |
| *Didelphis albiventris* | Host | Didelphimorphia | DTUI | -26.58300000 | -60.95000000 | Lauthier et al. 2012 | Google earth | Province | I | I |
| *Didelphis albiventris* | Host | Didelphimorphia | DTUI | -26.58300000 | -60.95000000 | Lauthier et al. 2012 | Google earth | Province | I | I |
| *Didelphis albiventris* | Host | Didelphimorphia | DTUI | -27.13000000 | -61.46000000 | Llewellyn et al. 2009b | Author | NA | I | I |
| *Didelphis albiventris* | Host | Didelphimorphia | DTUI | -27.13000000 | -61.46000000 | Llewellyn et al. 2009b | Author | NA | I | I |
| *Didelphis albiventris* | Host | Didelphimorphia | DTUI | -27.13300000 | -61.46000000 | Llewellyn et al. 2009b | Author | NA | I | I |
| *Didelphis albiventris* | Host | Didelphimorphia | DTUI | -27.13300000 | -61.46000000 | Llewellyn et al. 2009b | Author | NA | I | I |
| *Didelphis albiventris* | Host | Didelphimorphia | DTUI | -27.13300000 | -61.46000000 | Llewellyn et al. 2009b | Author | NA | I | I |
| *Didelphis albiventris* | Host | Didelphimorphia | DTUI | -27.13300000 | -61.46000000 | Llewellyn et al. 2009b | Author | NA | I | I |
| *Didelphis albiventris* | Host | Didelphimorphia | DTUI | -27.13300000 | -61.46000000 | Llewellyn et al. 2009b | Author | NA | I | I |
| *Didelphis albiventris* | Host | Didelphimorphia | DTUI | -26.93000000 | -61.58000000 | Llewellyn et al. 2009b | Author | NA | I | I |
| *Didelphis albiventris* | Host | Didelphimorphia | DTUI | -27.13300000 | -61.46000000 | Messenger et al. 2012 | Author | NA | I | I |
| *Didelphis albiventris* | Host | Didelphimorphia | DTUI | -27.13300000 | -61.46000000 | Messenger et al. 2012 | Author | NA | I | I |
| *Didelphis albiventris* | Host | Didelphimorphia | DTUI | -27.13300000 | -61.46000000 | Messenger et al. 2012 | Author | NA | I | I |
| *Didelphis albiventris* | Host | Didelphimorphia | DTUI | -27.13300000 | -61.46000000 | Messenger et al. 2012 | Author | NA | I | I |
| *Didelphis marsupialis* | Host | Didelphimorphia | DTUI | 32.43000000 | -83.31000000 | Llewellyn et al. 2009b | Author | NA | I | I |
| *Didelphis marsupialis* | Host | Didelphimorphia | DTUI | 32.43000000 | -83.31000000 | Llewellyn et al. 2009b | Author | NA | I | I |
| *Didelphis marsupialis* | Host | Didelphimorphia | DTUI | 32.43000000 | -83.31000000 | Llewellyn et al. 2009b | Author | NA | I | I |
| *Didelphis marsupialis* | Host | Didelphimorphia | DTUI | 30.50000000 | -91.00000000 | Llewellyn et al. 2009b | Author | NA | I | I |
| *Didelphis marsupialis* | Host | Didelphimorphia | DTUI | -14.81000000 | -64.60000000 | Llewellyn et al. 2009b | Author | NA | I | I |
| *Didelphis marsupialis* | Host | Didelphimorphia | DTUI | -14.81000000 | -64.60000000 | Llewellyn et al. 2009b | Author | NA | I | I |
| *Didelphis marsupialis* | Host | Didelphimorphia | DTUI | -14.81000000 | -64.60000000 | Llewellyn et al. 2009b | Author | NA | I | I |
| *Didelphis marsupialis* | Host | Didelphimorphia | DTUI | -14.81000000 | -64.60000000 | Llewellyn et al. 2009b | Author | NA | I | I |
| *Didelphis marsupialis* | Host | Didelphimorphia | DTUI | -14.81000000 | -64.60000000 | Llewellyn et al. 2009b | Author | NA | I | I |
| *Didelphis marsupialis* | Host | Didelphimorphia | DTUI | -14.81000000 | -64.60000000 | Llewellyn et al. 2009b | Author | NA | I | I |
| *Didelphis marsupialis* | Host | Didelphimorphia | DTUI | -14.81000000 | -64.60000000 | Llewellyn et al. 2009b | Author | NA | I | I |
| *Didelphis marsupialis* | Host | Didelphimorphia | DTUI | -14.81000000 | -64.60000000 | Llewellyn et al. 2009b | Author | NA | I | I |
| *Didelphis marsupialis* | Host | Didelphimorphia | DTUI | -14.81000000 | -64.60000000 | Llewellyn et al. 2009b | Author | NA | I | I |
| *Didelphis marsupialis* | Host | Didelphimorphia | DTUI | -14.81000000 | -64.60000000 | Llewellyn et al. 2009b | Author | NA | I | I |
| *Didelphis marsupialis* | Host | Didelphimorphia | DTUI | -14.81000000 | -64.60000000 | Llewellyn et al. 2009b | Author | NA | I | I |
| *Didelphis marsupialis* | Host | Didelphimorphia | DTUI | -14.81000000 | -64.60000000 | Llewellyn et al. 2009b | Author | NA | I | I |
| *Didelphis marsupialis* | Host | Didelphimorphia | DTUI | -0.15000000 | -50.38000000 | Llewellyn et al. 2009b | Author | NA | I | I |
| *Didelphis marsupialis* | Host | Didelphimorphia | DTUI | -0.15000000 | -50.38000000 | Llewellyn et al. 2009b | Author | NA | I | I |
| *Didelphis marsupialis* | Host | Didelphimorphia | DTUI | -1.38000000 | -48.86000000 | Llewellyn et al. 2009b | Author | NA | I | I |
| *Didelphis marsupialis* | Host | Didelphimorphia | DTUI | -5.98000000 | -51.33000000 | Llewellyn et al. 2009b | Author | NA | I | I |
| *Didelphis marsupialis* | Host | Didelphimorphia | DTUI | -5.98000000 | -51.33000000 | Llewellyn et al. 2009b | Author | NA | I | I |
| *Didelphis marsupialis* | Host | Didelphimorphia | DTUI | -3.07000000 | -60.16000000 | Llewellyn et al. 2009b | Author | NA | I | I |
| *Didelphis marsupialis* | Host | Didelphimorphia | DTUI | -5.98000000 | -51.33000000 | Llewellyn et al. 2009b | Author | NA | I | I |
| *Didelphis marsupialis* | Host | Didelphimorphia | DTUI | -5.98000000 | -51.33000000 | Llewellyn et al. 2009b | Author | NA | I | I |
| *Didelphis marsupialis* | Host | Didelphimorphia | DTUI | -5.98000000 | -51.33000000 | Llewellyn et al. 2009b | Author | NA | I | I |
| *Didelphis marsupialis* | Host | Didelphimorphia | DTUI | -1.36000000 | -48.36000000 | Llewellyn et al. 2009b | Author | NA | I | I |
| *Didelphis marsupialis* | Host | Didelphimorphia | DTUI | -1.36000000 | -48.36000000 | Llewellyn et al. 2009b | Author | NA | I | I |
| *Didelphis marsupialis* | Host | Didelphimorphia | DTUI | -5.83000000 | -48.03000000 | Llewellyn et al. 2009b | Author | NA | I | I |
| *Didelphis marsupialis* | Host | Didelphimorphia | DTUI | -1.36000000 | -48.36600000 | Llewellyn et al. 2009b | Author | NA | I | I |
| *Didelphis marsupialis* | Host | Didelphimorphia | DTUI | -1.51000000 | -49.21000000 | Llewellyn et al. 2009b | Author | NA | I | I |
| *Didelphis marsupialis* | Host | Didelphimorphia | DTUI | -0.15000000 | -50.38300000 | Llewellyn et al. 2009b | Author | NA | I | I |
| *Didelphis marsupialis* | Host | Didelphimorphia | DTUI | -5.98000000 | -51.33000000 | Llewellyn et al. 2009b | Author | NA | I | I |
| *Didelphis marsupialis* | Host | Didelphimorphia | DTUI | -1.51000000 | -49.21000000 | Llewellyn et al. 2009b | Author | NA | I | I |
| *Didelphis marsupialis* | Host | Didelphimorphia | DTUI | -1.05000000 | -46.76000000 | Llewellyn et al. 2009b | Author | NA | I | I |
| *Didelphis marsupialis* | Host | Didelphimorphia | DTUI | -1.05000000 | -46.76000000 | Llewellyn et al. 2009b | Author | NA | I | I |
| *Didelphis marsupialis* | Host | Didelphimorphia | DTUI | -1.05000000 | -46.76000000 | Llewellyn et al. 2009b | Author | NA | I | I |
| *Didelphis marsupialis* | Host | Didelphimorphia | DTUI | -1.71000000 | -48.88000000 | Llewellyn et al. 2009b | Author | NA | I | I |
| *Didelphis marsupialis* | Host | Didelphimorphia | DTUI | -1.71000000 | -48.88000000 | Llewellyn et al. 2009b | Author | NA | I | I |
| *Didelphis marsupialis* | Host | Didelphimorphia | DTUI | -1.71000000 | -48.88000000 | Llewellyn et al. 2009b | Author | NA | I | I |
| *Didelphis marsupialis* | Host | Didelphimorphia | DTUI | -1.05000000 | -46.76000000 | Llewellyn et al. 2009b | Author | NA | I | I |
| *Didelphis marsupialis* | Host | Didelphimorphia | DTUI | -1.71000000 | -48.88000000 | Llewellyn et al. 2009b | Author | NA | I | I |
| *Didelphis marsupialis* | Host | Didelphimorphia | DTUI | -1.71000000 | -48.88000000 | Llewellyn et al. 2009b | Author | NA | I | I |
| *Didelphis marsupialis* | Host | Didelphimorphia | DTUI | -9.18000000 | -48.18300000 | Llewellyn et al. 2009b | Author | NA | I | I |
| *Didelphis marsupialis* | Host | Didelphimorphia | DTUI | -1.38000000 | -48.86000000 | Llewellyn et al. 2009b | Author | NA | I | I |
| *Didelphis marsupialis* | Host | Didelphimorphia | DTUI | 2.76000000 | -60.53000000 | Llewellyn et al. 2009b | Author | NA | I | I |
| *Didelphis marsupialis* | Host | Didelphimorphia | DTUI | 1.71000000 | -77.91000000 | Llewellyn et al. 2009b | Author | NA | I | I |
| *Didelphis marsupialis* | Host | Didelphimorphia | DTUI | 10.11000000 | -64.55000000 | Llewellyn et al. 2009b | Author | NA | I | I |
| *Didelphis marsupialis* | Host | Didelphimorphia | DTUI | 10.11000000 | -64.55000000 | Llewellyn et al. 2009b | Author | NA | I | I |
| *Didelphis marsupialis* | Host | Didelphimorphia | DTUI | 10.35000000 | -67.03000000 | Llewellyn et al. 2009b | Author | NA | I | I |
| *Didelphis marsupialis* | Host | Didelphimorphia | DTUI | 10.54000000 | -67.80000000 | Llewellyn et al. 2009b | Author | NA | I | I |
| *Didelphis marsupialis* | Host | Didelphimorphia | DTUI | 10.54000000 | -67.80000000 | Llewellyn et al. 2009b | Author | NA | I | I |
| *Didelphis marsupialis* | Host | Didelphimorphia | DTUI | 9.55000000 | -70.51000000 | Llewellyn et al. 2009b | Author | NA | I | I |
| *Didelphis marsupialis* | Host | Didelphimorphia | DTUI | 9.55000000 | -70.51000000 | Llewellyn et al. 2009b | Author | NA | I | I |
| *Didelphis marsupialis* | Host | Didelphimorphia | DTUI | 10.46000000 | -63.61000000 | Llewellyn et al. 2009b | Author | NA | I | I |
| *Didelphis marsupialis* | Host | Didelphimorphia | DTUI | 10.46000000 | -63.61000000 | Llewellyn et al. 2009b | Author | NA | I | I |
| *Didelphis marsupialis* | Host | Didelphimorphia | DTUI | 7.50000000 | -71.23000000 | Llewellyn et al. 2009b | Author | NA | I | I |
| *Didelphis marsupialis* | Host | Didelphimorphia | DTUI | 7.50000000 | -71.23000000 | Llewellyn et al. 2009b | Author | NA | I | I |
| *Didelphis marsupialis* | Host | Didelphimorphia | DTUI | 7.50000000 | -71.23000000 | Llewellyn et al. 2009b | Author | NA | I | I |
| *Didelphis marsupialis* | Host | Didelphimorphia | DTUI | 7.50000000 | -71.23000000 | Llewellyn et al. 2009b | Author | NA | I | I |
| *Didelphis marsupialis* | Host | Didelphimorphia | DTUI | 7.50000000 | -71.23000000 | Llewellyn et al. 2009b | Author | NA | I | I |
| *Didelphis marsupialis* | Host | Didelphimorphia | DTUI | 7.50000000 | -71.23000000 | Llewellyn et al. 2009b | Author | NA | I | I |
| *Didelphis marsupialis* | Host | Didelphimorphia | DTUI | 8.43000000 | -70.55000000 | Llewellyn et al. 2009b | Author | NA | I | I |
| *Didelphis marsupialis* | Host | Didelphimorphia | DTUI | 8.43000000 | -70.55000000 | Llewellyn et al. 2009b | Author | NA | I | I |
| *Didelphis marsupialis* | Host | Didelphimorphia | DTUI | 8.43000000 | -70.55000000 | Llewellyn et al. 2009b | Author | NA | I | I |
| *Didelphis marsupialis* | Host | Didelphimorphia | DTUI | -14.81000000 | -64.60000000 | Messenger et al. 2012 | Author | NA | I | I |
| *Didelphis marsupialis* | Host | Didelphimorphia | DTUI | -14.81000000 | -64.60000000 | Messenger et al. 2012 | Author | NA | I | I |
| *Didelphis marsupialis* | Host | Didelphimorphia | DTUI | -14.81000000 | -64.60000000 | Messenger et al. 2012 | Author | NA | I | I |
| *Didelphis marsupialis* | Host | Didelphimorphia | DTUI | -14.81000000 | -64.60000000 | Messenger et al. 2012 | Author | NA | I | I |
| *Didelphis marsupialis* | Host | Didelphimorphia | DTUI | -1.71000000 | -48.88000000 | Messenger et al. 2012 | Author | NA | I | I |
| *Didelphis marsupialis* | Host | Didelphimorphia | DTUI | -3.07000000 | -60.16000000 | Messenger et al. 2012 | Author | NA | I | I |
| *Didelphis marsupialis* | Host | Didelphimorphia | DTUI | -1.30000000 | -48.36000000 | Messenger et al. 2012 | Author | NA | I | I |
| *Didelphis marsupialis* | Host | Didelphimorphia | DTUI | -5.83000000 | -48.03000000 | Messenger et al. 2012 | Author | NA | I | I |
| *Didelphis marsupialis* | Host | Didelphimorphia | DTUI | 32.43000000 | -83.31000000 | Messenger et al. 2012 | Author | NA | I | I |
| *Didelphis marsupialis* | Host | Didelphimorphia | DTUI | 32.43000000 | -83.31000000 | Messenger et al. 2012 | Author | NA | I | I |
| *Didelphis marsupialis* | Host | Didelphimorphia | DTUI | 30.50000000 | -91.00000000 | Messenger et al. 2012 | Author | NA | I | I |
| *Didelphis marsupialis* | Host | Didelphimorphia | DTUI | 7.50000000 | -71.23000000 | Messenger et al. 2012 | Author | NA | I | I |
| *Didelphis marsupialis* | Host | Didelphimorphia | DTUI | 7.50000000 | -71.23000000 | Messenger et al. 2012 | Author | NA | I | I |
| *Didelphis marsupialis* | Host | Didelphimorphia | DTUI | 7.50000000 | -71.23000000 | Messenger et al. 2012 | Author | NA | I | I |
| *Didelphis marsupialis* | Host | Didelphimorphia | DTUI | 7.50000000 | -71.23000000 | Messenger et al. 2012 | Author | NA | I | I |
| *Didelphis marsupialis* | Host | Didelphimorphia | DTUI | -0.26700000 | -63.20000000 | Marcili et al. 2009b | Google earth | Locality | I | I |
| *Didelphis marsupialis* | Host | Didelphimorphia | DTUI | -6.06700000 | -49.90000000 | Marcili et al. 2009b | Google earth | Locality | I | I |
| *Didelphis marsupialis* | Host | Didelphimorphia | DTUI | 9.13000000 | -75.05000000 | Mejia-Jaramillo et al. 2009 | Google earth | Department/State | I | I |
| *Didelphis marsupialis* | Host | Didelphimorphia | DTUI | 5.75000000 | -71.56700000 | Mejia-Jaramillo et al. 2009 | Google earth | Department/State | I | I |
| *Didelphis marsupialis* | Host | Didelphimorphia | DTUI | 8.58300000 | -63.95000000 | Carrasco et al. 2012 | Google earth | State | I | I |
| *Didelphis marsupialis* | Host | Didelphimorphia | DTUI | 8.58300000 | -63.95000000 | Carrasco et al. 2012 | Google earth | State | I | I |
| *Didelphis marsupialis* | Host | Didelphimorphia | DTUI | 8.58300000 | -63.95000000 | Carrasco et al. 2012 | Google earth | State | I | I |
| *Didelphis marsupialis* | Host | Didelphimorphia | DTUI | 8.58300000 | -63.95000000 | Carrasco et al. 2012 | Google earth | State | I | I |
| *Didelphis marsupialis* | Host | Didelphimorphia | DTUI | 8.58300000 | -63.95000000 | Carrasco et al. 2012 | Google earth | State | I | I |
| *Didelphis marsupialis* | Host | Didelphimorphia | DTUI | 8.58300000 | -63.95000000 | Carrasco et al. 2012 | Google earth | State | I | I |
| *Didelphis marsupialis* | Host | Didelphimorphia | DTUI | 8.58300000 | -63.95000000 | Carrasco et al. 2012 | Google earth | State | I | I |
| *Didelphis marsupialis* | Host | Didelphimorphia | DTUI | 8.58300000 | -63.95000000 | Carrasco et al. 2012 | Google earth | State | I | I |
| *Didelphis marsupialis* | Host | Didelphimorphia | DTUI | 8.58300000 | -63.95000000 | Carrasco et al. 2012 | Google earth | State | I | I |
| *Didelphis marsupialis* | Host | Didelphimorphia | DTUI | 8.58300000 | -63.95000000 | Carrasco et al. 2012 | Google earth | State | I | I |
| *Didelphis marsupialis* | Host | Didelphimorphia | DTUI | 8.58300000 | -63.95000000 | Carrasco et al. 2012 | Google earth | State | I | I |
| *Didelphis marsupialis* | Host | Didelphimorphia | DTUI | 8.58300000 | -63.95000000 | Carrasco et al. 2012 | Google earth | State | I | I |
| *Didelphis marsupialis* | Host | Didelphimorphia | DTUI | 8.58300000 | -63.95000000 | Carrasco et al. 2012 | Google earth | State | I | I |
| *Didelphis marsupialis* | Host | Didelphimorphia | DTUI | 8.58300000 | -63.95000000 | Carrasco et al. 2012 | Google earth | State | I | I |
| *Didelphis marsupialis* | Host | Didelphimorphia | DTUI | 8.58300000 | -63.95000000 | Carrasco et al. 2012 | Google earth | State | I | I |
| *Didelphis marsupialis* | Host | Didelphimorphia | DTUI | 8.58300000 | -63.95000000 | Carrasco et al. 2012 | Google earth | State | I | I |
| *Didelphis marsupialis* | Host | Didelphimorphia | DTUI | 8.58300000 | -63.95000000 | Carrasco et al. 2012 | Google earth | State | I | I |
| *Didelphis marsupialis* | Host | Didelphimorphia | DTUI | 8.58300000 | -63.95000000 | Carrasco et al. 2012 | Google earth | State | I | I |
| *Didelphis marsupialis* | Host | Didelphimorphia | DTUI | 8.30000000 | -70.05000000 | Carrasco et al. 2012 | Google earth | State | I | I |
| *Didelphis marsupialis* | Host | Didelphimorphia | DTUI | 8.30000000 | -70.05000000 | Carrasco et al. 2012 | Google earth | State | I | I |
| *Didelphis marsupialis* | Host | Didelphimorphia | DTUI | 8.30000000 | -70.05000000 | Carrasco et al. 2012 | Google earth | State | I | I |
| *Didelphis marsupialis* | Host | Didelphimorphia | DTUI | 8.30000000 | -70.05000000 | Carrasco et al. 2012 | Google earth | State | I | I |
| *Didelphis marsupialis* | Host | Didelphimorphia | DTUI | 8.30000000 | -70.05000000 | Carrasco et al. 2012 | Google earth | State | I | I |
| *Didelphis marsupialis* | Host | Didelphimorphia | DTUI | 8.30000000 | -70.05000000 | Carrasco et al. 2012 | Google earth | State | I | I |
| *Didelphis marsupialis* | Host | Didelphimorphia | DTUI | 8.30000000 | -70.05000000 | Carrasco et al. 2012 | Google earth | State | I | I |
| *Didelphis marsupialis* | Host | Didelphimorphia | DTUI | 8.30000000 | -70.05000000 | Carrasco et al. 2012 | Google earth | State | I | I |
| *Didelphis marsupialis* | Host | Didelphimorphia | DTUI | 8.30000000 | -70.05000000 | Carrasco et al. 2012 | Google earth | State | I | I |
| *Didelphis marsupialis* | Host | Didelphimorphia | DTUI | 8.30000000 | -70.05000000 | Carrasco et al. 2012 | Google earth | State | I | I |
| *Didelphis marsupialis* | Host | Didelphimorphia | DTUI | 10.11670000 | -68.03300000 | Carrasco et al. 2012 | Google earth | State | I | I |
| *Didelphis marsupialis* | Host | Didelphimorphia | DTUI | 10.05000000 | -66.95000000 | Carrasco et al. 2012 | Google earth | State | I | I |
| *Didelphis marsupialis* | Host | Didelphimorphia | DTUI | 8.73000000 | -66.23000000 | Carrasco et al. 2012 | Google earth | State | I | I |
| *Didelphis marsupialis* | Host | Didelphimorphia | DTUI | 10.25000000 | -66.41670000 | Carrasco et al. 2012 | Google earth | State | I | I |
| *Didelphis marsupialis* | Host | Didelphimorphia | DTUI | 10.25000000 | -66.41670000 | Carrasco et al. 2012 | Google earth | State | I | I |
| *Didelphis marsupialis* | Host | Didelphimorphia | DTUI | 10.25000000 | -66.41670000 | Carrasco et al. 2012 | Google earth | State | I | I |
| *Didelphis marsupialis* | Host | Didelphimorphia | DTUI | 10.25000000 | -66.41670000 | Carrasco et al. 2012 | Google earth | State | I | I |
| *Didelphis marsupialis* | Host | Didelphimorphia | DTUI | 9.08300000 | -69.08300000 | Carrasco et al. 2012 | Google earth | State | I | I |
| *Didelphis marsupialis* | Host | Didelphimorphia | DTUI | 9.08300000 | -69.08300000 | Carrasco et al. 2012 | Google earth | State | I | I |
| *Didelphis marsupialis* | Host | Didelphimorphia | DTUI | 9.08300000 | -69.08300000 | Carrasco et al. 2012 | Google earth | State | I | I |
| *Didelphis marsupialis* | Host | Didelphimorphia | DTUI | 9.08300000 | -69.08300000 | Carrasco et al. 2012 | Google earth | State | I | I |
| *Didelphis marsupialis* | Host | Didelphimorphia | DTUI | 9.08300000 | -69.08300000 | Carrasco et al. 2012 | Google earth | State | I | I |
| *Didelphis marsupialis* | Host | Didelphimorphia | DTUI | 9.08300000 | -69.08300000 | Carrasco et al. 2012 | Google earth | State | I | I |
| *Didelphis marsupialis* | Host | Didelphimorphia | DTUI | 9.08300000 | -69.08300000 | Carrasco et al. 2012 | Google earth | State | I | I |
| *Didelphis marsupialis* | Host | Didelphimorphia | DTUI | 9.08300000 | -69.08300000 | Carrasco et al. 2012 | Google earth | State | I | I |
| *Didelphis marsupialis* | Host | Didelphimorphia | DTUI | 10.40000000 | -63.28300000 | Carrasco et al. 2012 | Google earth | State | I | I |
| *Didelphis marsupialis* | Host | Didelphimorphia | DTUI | 10.40000000 | -63.28300000 | Carrasco et al. 2012 | Google earth | State | I | I |
| *Didelphis marsupialis* | Host | Didelphimorphia | DTUI | 10.40000000 | -63.28300000 | Carrasco et al. 2012 | Google earth | State | I | I |
| *Didelphis marsupialis* | Host | Didelphimorphia | DTUI | 10.40000000 | -63.28300000 | Carrasco et al. 2012 | Google earth | State | I | I |
| *Didelphis marsupialis* | Host | Didelphimorphia | DTUI | 9.36700000 | -70.41670000 | Carrasco et al. 2012 | Google earth | State | I | I |
| *Didelphis marsupialis* | Host | Didelphimorphia | DTUI | 9.36700000 | -70.41670000 | Carrasco et al. 2012 | Google earth | State | I | I |
| *Didelphis marsupialis* | Host | Didelphimorphia | DTUI | -22.416944 | -42.975556 | Araujo et al. 2011 | Google earth | Locality | I | I |
| *Didelphis marsupialis* | Host | Didelphimorphia | DTUII | -22.416944 | -42.975556 | Araujo et al. 2011 | Google earth | Locality | II | II |
| *Didelphis marsupialis* | Host | Didelphimorphia | DTUI | -4.07800000 | -79.80700000 | Ocaña-Mayorga et al. 2010 | Author | NA | I | I |
| *Didelphis marsupialis* | Host | Didelphimorphia | DTUI | -4.09400000 | -79.34900000 | Ocaña-Mayorga et al. 2010 | Author | NA | I | I |
| *Didelphis marsupialis* | Host | Didelphimorphia | DTUI | -4.13500000 | -79.61400000 | Ocaña-Mayorga et al. 2010 | Author | NA | I | I |
| *Didelphis marsupialis* | Host | Didelphimorphia | DTUI | -4.07800000 | -79.80700000 | Ocaña-Mayorga et al. 2010 | Author | NA | I | I |
| *Didelphis novemcinctus* | Host | Didelphimorphia | DTUIII | 8.58300000 | -63.95000000 | Carrasco et al. 2012 | Google earth | State | II | III |
| *Didelphis novemcinctus* | Host | Didelphimorphia | DTUIII | 8.58300000 | -63.95000000 | Carrasco et al. 2012 | Google earth | State | II | III |
| *Didelphis novemcinctus* | Host | Didelphimorphia | DTUIII | 8.58300000 | -63.95000000 | Carrasco et al. 2012 | Google earth | State | II | III |
| *Didelphis novemcinctus* | Host | Didelphimorphia | DTUIII | 8.58300000 | -63.95000000 | Carrasco et al. 2012 | Google earth | State | II | III |
| *Didelphis novemcinctus* | Host | Didelphimorphia | DTUIII | 8.30000000 | -70.05000000 | Carrasco et al. 2012 | Google earth | State | II | III |
| *Didelphis novemcinctus* | Host | Didelphimorphia | DTUIII | 8.30000000 | -70.05000000 | Carrasco et al. 2012 | Google earth | State | II | III |
| *Didelphis novemcinctus* | Host | Didelphimorphia | DTUIII | 8.30000000 | -70.05000000 | Carrasco et al. 2012 | Google earth | State | II | III |
| *Didelphis novemcinctus* | Host | Didelphimorphia | DTUIII | 8.30000000 | -70.05000000 | Carrasco et al. 2012 | Google earth | State | II | III |
| *Didelphis novemcinctus* | Host | Didelphimorphia | DTUIII | 8.30000000 | -70.05000000 | Carrasco et al. 2012 | Google earth | State | II | III |
| *Didelphis novemcinctus* | Host | Didelphimorphia | DTUIII | 8.30000000 | -70.05000000 | Carrasco et al. 2012 | Google earth | State | II | III |
| *Didelphis novemcinctus* | Host | Didelphimorphia | DTUIII | 8.30000000 | -70.05000000 | Carrasco et al. 2012 | Google earth | State | II | III |
| *Didelphis novemcinctus* | Host | Didelphimorphia | DTUIII | 8.30000000 | -70.05000000 | Carrasco et al. 2012 | Google earth | State | II | III |
| *Didelphis novemcinctus* | Host | Didelphimorphia | DTUIII | 8.30000000 | -70.05000000 | Carrasco et al. 2012 | Google earth | State | II | III |
| *Didelphis sp* | Host | Didelphimorphia | DTUI | -23.58300 | -52.08300 | Zalloum et al. 2005 | Google earth | Community | I | I |
| *Didelphis sp* | Host | Didelphimorphia | DTUI | -23.41600 | -51.91670 | Zalloum et al. 2005 | Google earth | Community | I | I |
| *Didelphis sp* | Host | Didelphimorphia | DTUI | -23.55000 | -52.20000 | Zalloum et al. 2005 | Google earth | Community | I | I |
| *Didelphis virginiana* | Host | Didelphimorphia | DTUI | 20.98138889 | -89.59250000 | Cura et al. 2015 | Google earth | Locality | I | I |
| *Didelphis virginiana* | Host | Didelphimorphia | DTUI | 20.81666667 | -89.63027778 | Cura et al. 2015 | Google earth | Locality | I | I |
| *Didelphis virginiana* | Host | Didelphimorphia | DTUI | 20.81666667 | -89.63027778 | Cura et al. 2015 | Google earth | Locality | I | I |
| *Didelphis virginiana* | Host | Didelphimorphia | DTUI | 20.81666667 | -89.63027778 | Cura et al. 2015 | Google earth | Locality | I | I |
| *Didelphis virginiana* | Host | Didelphimorphia | DTUI | 20.81666667 | -89.63027778 | Cura et al. 2015 | Google earth | Locality | I | I |
| *Didelphis virginiana* | Host | Didelphimorphia | DTUI | 20.81666667 | -89.63027778 | Cura et al. 2015 | Google earth | Locality | I | I |
| *Didelphis virginiana* | Host | Didelphimorphia | DTUI | 20.81666667 | -89.63027778 | Cura et al. 2015 | Google earth | Locality | I | I |
| *Didelphis virginiana* | Host | Didelphimorphia | DTUI | 20.81666667 | -89.63027778 | Cura et al. 2015 | Google earth | Locality | I | I |
| *Didelphis virginiana* | Host | Didelphimorphia | DTUI | 20.81666667 | -89.63027778 | Cura et al. 2015 | Google earth | Locality | I | I |
| *Didelphis virginiana* | Host | Didelphimorphia | DTUI | 20.81666667 | -89.63027778 | Cura et al. 2015 | Google earth | Locality | I | I |
| *Didelphis virginiana* | Host | Didelphimorphia | DTUI | 20.81666667 | -89.63027778 | Cura et al. 2015 | Google earth | Locality | I | I |
| *Didelphis virginiana* | Host | Didelphimorphia | DTUI | 20.81666667 | -89.63027778 | Cura et al. 2015 | Google earth | Locality | I | I |
| *Didelphis virginiana* | Host | Didelphimorphia | DTUI | 17.48472222 | -92.04583333 | Cura et al. 2015 | Google earth | Locality | I | I |
| *Didelphis virginiana* | Host | Didelphimorphia | DTUII | 20.96722222 | -89.59250000 | Cura et al. 2015 | Google earth | Locality | II | II |
| *Dipetalogaster maxima* | Vector | Hemiptera | DTUI | 26.04400000 | -111.66600000 | Zumaya-Estrada et al. 2012 | Author | NA | I | I |
| *Eptesicus sp* | Host | Chiroptera | DTUI | -0.08300 | -48.90000 | Lisboa et al. 2008 | Google earth | Biomas Brasil | I | I |
| *Eratyrus cuspidatus* | Vector | Hemiptera | DTUI | 10.40000000 | -74.40000000 | Mejia-Jaramillo et al. 2009 | Google earth | Department/State | I | I |
| *Eratyrus cuspidatus* | Vector | Hemiptera | DTUI | 10.40000000 | -74.40000000 | Mejia-Jaramillo et al. 2009 | Google earth | Department/State | I | I |
| *Eratyrus mucronatus* | Vector | Hemiptera | DTUI | 10.25000000 | -66.41670000 | Carrasco et al. 2012 | Google earth | State | I | I |
| *Eratyrus mucronatus* | Vector | Hemiptera | DTUI | 10.25000000 | -66.41670000 | Carrasco et al. 2012 | Google earth | State | I | I |
| *Eratyrus mucronatus* | Vector | Hemiptera | DTUII | 3.86027800000 | -53.30416670000 | Cura et al. 2015 | Google earth | Locality | II | II |
| *Euphractus sexcinctus* | Host | Cingulata | DTUIII | -17.50000000 | -61.50000000 | Llewellyn et al. 2009a | Author | NA | II | III |
| *Euphractus sexcinctus* | Host | Cingulata | DTUIII | -17.50000000 | -61.50000000 | Llewellyn et al. 2009a | Author | NA | II | III |
| *Euphractus sexcinctus* | Host | Cingulata | DTUIII | -19.21000000 | -63.43000000 | Llewellyn et al. 2009a | Author | NA | II | III |
| *Euphractus sexcinctus* | Host | Cingulata | DTUIII | -22.33000000 | -58.93000000 | Llewellyn et al. 2009a | Author | NA | II | III |
| *Euphractus sexcinctus* | Host | Cingulata | DTUIII | -22.33000000 | -58.93000000 | Llewellyn et al. 2009a | Author | NA | II | III |
| *Felis catus* | Host | Carnivora | DTUI | 20.98138889 | -89.59250000 | Cura et al. 2015 | Google earth | Locality | I | I |
| *Felis catus* | Host | Carnivora | DTUI | 20.98138889 | -89.59250000 | Cura et al. 2015 | Google earth | Locality | I | I |
| *Felis catus* | Host | Carnivora | DTUI | 20.98138889 | -89.59250000 | Cura et al. 2015 | Google earth | Locality | I | I |
| *Felis catus* | Host | Carnivora | DTUI | 20.98138889 | -89.59250000 | Cura et al. 2015 | Google earth | Locality | I | I |
| *Felis catus* | Host | Carnivora | DTUI | 20.98138889 | -89.59250000 | Cura et al. 2015 | Google earth | Locality | I | I |
| *Felis catus* | Host | Carnivora | DTUI | 20.98138889 | -89.59250000 | Cura et al. 2015 | Google earth | Locality | I | I |
| *Felis catus* | Host | Carnivora | DTUI | 20.98138889 | -89.59250000 | Cura et al. 2015 | Google earth | Locality | I | I |
| *Felis catus* | Host | Carnivora | DTUI | 20.98138889 | -89.59250000 | Cura et al. 2015 | Google earth | Locality | I | I |
| *Felis catus* | Host | Carnivora | DTUI | 20.98138889 | -89.59250000 | Cura et al. 2015 | Google earth | Locality | I | I |
| *Felis catus* | Host | Carnivora | DTUI | 20.98138889 | -89.59250000 | Cura et al. 2015 | Google earth | Locality | I | I |
| *Felis catus* | Host | Carnivora | DTUI | 20.98138889 | -89.59250000 | Cura et al. 2015 | Google earth | Locality | I | I |
| *Felis catus* | Host | Carnivora | DTUI | 20.98138889 | -89.59250000 | Cura et al. 2015 | Google earth | Locality | I | I |
| *Felis catus* | Host | Carnivora | DTUI | 20.98138889 | -89.59250000 | Cura et al. 2015 | Google earth | Locality | I | I |
| *Felis catus* | Host | Carnivora | DTUI | 20.98138889 | -89.59250000 | Cura et al. 2015 | Google earth | Locality | I | I |
| *Felis catus* | Host | Carnivora | DTUI | 20.98138889 | -89.59250000 | Cura et al. 2015 | Google earth | Locality | I | I |
| *Felis catus* | Host | Carnivora | DTUI | 20.98138889 | -89.59250000 | Cura et al. 2015 | Google earth | Locality | I | I |
| *Felis catus* | Host | Carnivora | DTUI | 20.98138889 | -89.59250000 | Cura et al. 2015 | Google earth | Locality | I | I |
| *Felis catus* | Host | Carnivora | DTUI | 20.98138889 | -89.59250000 | Cura et al. 2015 | Google earth | Locality | I | I |
| *Felis catus* | Host | Carnivora | DTUI | 20.98138889 | -89.59250000 | Cura et al. 2015 | Google earth | Locality | I | I |
| *Felis catus* | Host | Carnivora | DTUI | 20.98138889 | -89.59250000 | Cura et al. 2015 | Google earth | Locality | I | I |
| *Felis catus* | Host | Carnivora | DTUI | 20.98138889 | -89.59250000 | Cura et al. 2015 | Google earth | Locality | I | I |
| *Felis catus* | Host | Carnivora | DTUI | 20.98138889 | -89.59250000 | Cura et al. 2015 | Google earth | Locality | I | I |
| *Felis catus* | Host | Carnivora | DTUII | 20.96722222 | -89.59250000 | Cura et al. 2015 | Google earth | Locality | II | II |
| *Felis catus* | Host | Carnivora | DTUV | -25.08333333 | -56.96666667 | Enriquez et al. 2014 | Google earth | Locality | II | ND |
| *Felis catus* | Host | Carnivora | DTUV | -25.08333333 | -56.96666667 | Enriquez et al. 2014 | Google earth | Locality | II | ND |
| *Felis catus* | Host | Carnivora | DTUVI | -25.08333333 | -56.96666667 | Enriquez et al. 2014 | Google earth | Locality | II | ND |
| *Felis catus* | Host | Carnivora | DTUVI | -25.08333333 | -56.96666667 | Enriquez et al. 2014 | Google earth | Locality | II | ND |
| *Felis catus* | Host | Carnivora | DTUVI | -25.08333333 | -56.96666667 | Enriquez et al. 2014 | Google earth | Locality | II | ND |
| *Felis catus* | Host | Carnivora | DTUVI | -25.08333333 | -56.96666667 | Enriquez et al. 2014 | Google earth | Locality | II | ND |
| *Felis catus* | Host | Carnivora | DTUVI | -25.08333333 | -56.96666667 | Enriquez et al. 2014 | Google earth | Locality | II | ND |
| *Felis catus* | Host | Carnivora | DTUVI | -25.08333333 | -56.96666667 | Enriquez et al. 2014 | Google earth | Locality | II | ND |
| *Felis catus* | Host | Carnivora | DTUVI | -25.08333333 | -56.96666667 | Enriquez et al. 2014 | Google earth | Locality | II | ND |
| *Felis catus* | Host | Carnivora | DTUVI | -25.08333333 | -56.96666667 | Enriquez et al. 2014 | Google earth | Locality | II | ND |
| *Felis catus* | Host | Carnivora | DTUVI | -25.08333333 | -56.96666667 | Enriquez et al. 2014 | Google earth | Locality | II | ND |
| *Felis catus* | Host | Carnivora | DTUVI | -25.08333333 | -56.96666667 | Enriquez et al. 2014 | Google earth | Locality | II | ND |
| *Heteromys anomalus* | Host | Rodentia | DTUI | 10.95000000 | -73.46700000 | Rodriguez et al. 2009 | Google earth | Area/Unspecified communities | I | I |
| *Heteromys anomalus* | Host | Rodentia | DTUI | 10.95000000 | -73.46700000 | Rodriguez et al. 2009 | Google earth | Area/Unspecified communities | I | I |
| *Homo sapiens* | Host | Primates | DTUI | -17.38000000 | -66.16000000 | Llewellyn et al. 2009b | Author | NA | I | I |
| *Homo sapiens* | Host | Primates | DTUI | -17.38000000 | -66.16000000 | Llewellyn et al. 2009b | Author | NA | I | I |
| *Homo sapiens* | Host | Primates | DTUI | -17.38000000 | -66.16000000 | Llewellyn et al. 2009b | Author | NA | I | I |
| *Homo sapiens* | Host | Primates | DTUI | -17.38000000 | -66.16000000 | Llewellyn et al. 2009b | Author | NA | I | I |
| *Homo sapiens* | Host | Primates | DTUI | 7.76000000 | -72.25000000 | Llewellyn et al. 2009b | Author | NA | I | I |
| *Homo sapiens* | Host | Primates | DTUI | 10.23000000 | -66.66000000 | Llewellyn et al. 2009b | Author | NA | I | I |
| *Homo sapiens* | Host | Primates | DTUI | 10.46000000 | -63.61000000 | Llewellyn et al. 2009b | Author | NA | I | I |
| *Homo sapiens* | Host | Primates | DTUI | 8.37000000 | -70.51000000 | Llewellyn et al. 2009b | Author | NA | I | I |
| *Homo sapiens* | Host | Primates | DTUI | 8.91000000 | -65.38000000 | Llewellyn et al. 2009b | Author | NA | I | I |
| *Homo sapiens* | Host | Primates | DTUI | 9.66000000 | -68.20000000 | Llewellyn et al. 2009b | Author | NA | I | I |
| *Homo sapiens* | Host | Primates | DTUI | 8.66000000 | -69.50000000 | Llewellyn et al. 2009b | Author | NA | I | I |
| *Homo sapiens* | Host | Primates | DTUI | 10.61000000 | -67.04000000 | Llewellyn et al. 2009b | Author | NA | I | I |
| *Homo sapiens* | Host | Primates | DTUI | 8.59000000 | -71.23000000 | Llewellyn et al. 2009b | Author | NA | I | I |
| *Homo sapiens* | Host | Primates | DTUI | 8.59000000 | -71.23000000 | Llewellyn et al. 2009b | Author | NA | I | I |
| *Homo sapiens* | Host | Primates | DTUI | 10.04000000 | -69.32000000 | Llewellyn et al. 2009b | Author | NA | I | I |
| *Homo sapiens* | Host | Primates | DTUI | 9.01000000 | -69.29000000 | Llewellyn et al. 2009b | Author | NA | I | I |
| *Homo sapiens* | Host | Primates | DTUI | 8.71000000 | -66.62000000 | Llewellyn et al. 2009b | Author | NA | I | I |
| *Homo sapiens* | Host | Primates | DTUI | 9.36000000 | -65.12000000 | Llewellyn et al. 2009b | Author | NA | I | I |
| *Homo sapiens* | Host | Primates | DTUI | 9.55000000 | -70.51000000 | Llewellyn et al. 2009b | Author | NA | I | I |
| *Homo sapiens* | Host | Primates | DTUI | 9.01000000 | -64.34000000 | Llewellyn et al. 2009b | Author | NA | I | I |
| *Homo sapiens* | Host | Primates | DTUI | 10.95000000 | -73.46700000 | Rodriguez et al. 2009 | Google earth | Area/Unspecified communities | I | I |
| *Homo sapiens* | Host | Primates | DTUI | 10.95000000 | -73.46700000 | Rodriguez et al. 2009 | Google earth | Area/Unspecified communities | I | I |
| *Homo sapiens* | Host | Primates | DTUI | 10.95000000 | -73.46700000 | Rodriguez et al. 2009 | Google earth | Area/Unspecified communities | I | I |
| *Homo sapiens* | Host | Primates | DTUI | 10.95000000 | -73.46700000 | Rodriguez et al. 2009 | Google earth | Area/Unspecified communities | I | I |
| *Homo sapiens* | Host | Primates | DTUI | 10.95000000 | -73.46700000 | Rodriguez et al. 2009 | Google earth | Area/Unspecified communities | I | I |
| *Homo sapiens* | Host | Primates | DTUI | 10.95000000 | -73.46700000 | Rodriguez et al. 2009 | Google earth | Area/Unspecified communities | I | I |
| *Homo sapiens* | Host | Primates | DTUI | 10.95000000 | -73.46700000 | Rodriguez et al. 2009 | Google earth | Area/Unspecified communities | I | I |
| *Homo sapiens* | Host | Primates | DTUI | 10.95000000 | -73.46700000 | Rodriguez et al. 2009 | Google earth | Area/Unspecified communities | I | I |
| *Homo sapiens* | Host | Primates | DTUI | 10.95000000 | -73.46700000 | Rodriguez et al. 2009 | Google earth | Area/Unspecified communities | I | I |
| *Homo sapiens* | Host | Primates | DTUI | 10.95000000 | -73.46700000 | Rodriguez et al. 2009 | Google earth | Area/Unspecified communities | I | I |
| *Homo sapiens* | Host | Primates | DTUI | 10.95000000 | -73.46700000 | Rodriguez et al. 2009 | Google earth | Area/Unspecified communities | I | I |
| *Homo sapiens* | Host | Primates | DTUI | 10.95000000 | -73.46700000 | Rodriguez et al. 2009 | Google earth | Area/Unspecified communities | I | I |
| *Homo sapiens* | Host | Primates | DTUI | 10.95000000 | -73.46700000 | Rodriguez et al. 2009 | Google earth | Area/Unspecified communities | I | I |
| *Homo sapiens* | Host | Primates | DTUI | 10.95000000 | -73.46700000 | Rodriguez et al. 2009 | Google earth | Area/Unspecified communities | I | I |
| *Homo sapiens* | Host | Primates | DTUI | 10.95000000 | -73.46700000 | Rodriguez et al. 2009 | Google earth | Area/Unspecified communities | I | I |
| *Homo sapiens* | Host | Primates | DTUI | 10.95000000 | -73.46700000 | Rodriguez et al. 2009 | Google earth | Area/Unspecified communities | I | I |
| *Homo sapiens* | Host | Primates | DTUI | 10.95000000 | -73.46700000 | Rodriguez et al. 2009 | Google earth | Area/Unspecified communities | I | I |
| *Homo sapiens* | Host | Primates | DTUI | 10.95000000 | -73.46700000 | Rodriguez et al. 2009 | Google earth | Area/Unspecified communities | I | I |
| *Homo sapiens* | Host | Primates | DTUI | 10.95000000 | -73.46700000 | Rodriguez et al. 2009 | Google earth | Area/Unspecified communities | I | I |
| *Homo sapiens* | Host | Primates | DTUI | 10.95000000 | -73.46700000 | Rodriguez et al. 2009 | Google earth | Area/Unspecified communities | I | I |
| *Homo sapiens* | Host | Primates | DTUI | 10.95000000 | -73.46700000 | Rodriguez et al. 2009 | Google earth | Area/Unspecified communities | I | I |
| *Homo sapiens* | Host | Primates | DTUI | 10.95000000 | -73.46700000 | Rodriguez et al. 2009 | Google earth | Area/Unspecified communities | I | I |
| *Homo sapiens* | Host | Primates | DTUI | 10.95000000 | -73.46700000 | Rodriguez et al. 2009 | Google earth | Area/Unspecified communities | I | I |
| *Homo sapiens* | Host | Primates | DTUI | 10.95000000 | -73.46700000 | Rodriguez et al. 2009 | Google earth | Area/Unspecified communities | I | I |
| *Homo sapiens* | Host | Primates | DTUI | 10.95000000 | -73.46700000 | Rodriguez et al. 2009 | Google earth | Area/Unspecified communities | I | I |
| *Homo sapiens* | Host | Primates | DTUI | 10.95000000 | -73.46700000 | Rodriguez et al. 2009 | Google earth | Area/Unspecified communities | I | I |
| *Homo sapiens* | Host | Primates | DTUI | 10.95000000 | -73.46700000 | Rodriguez et al. 2009 | Google earth | Area/Unspecified communities | I | I |
| *Homo sapiens* | Host | Primates | DTUI | 10.95000000 | -73.46700000 | Rodriguez et al. 2009 | Google earth | Area/Unspecified communities | I | I |
| *Homo sapiens* | Host | Primates | DTUI | 10.95000000 | -73.46700000 | Rodriguez et al. 2009 | Google earth | Area/Unspecified communities | I | I |
| *Homo sapiens* | Host | Primates | DTUI | 10.95000000 | -73.46700000 | Rodriguez et al. 2009 | Google earth | Area/Unspecified communities | I | I |
| *Homo sapiens* | Host | Primates | DTUI | 10.95000000 | -73.46700000 | Rodriguez et al. 2009 | Google earth | Area/Unspecified communities | I | I |
| *Homo sapiens* | Host | Primates | DTUI | 10.95000000 | -73.46700000 | Rodriguez et al. 2009 | Google earth | Area/Unspecified communities | I | I |
| *Homo sapiens* | Host | Primates | DTUI | 10.95000000 | -73.46700000 | Rodriguez et al. 2009 | Google earth | Area/Unspecified communities | I | I |
| *Homo sapiens* | Host | Primates | DTUI | 10.95000000 | -73.46700000 | Rodriguez et al. 2009 | Google earth | Area/Unspecified communities | I | I |
| *Homo sapiens* | Host | Primates | DTUI | 10.95000000 | -73.46700000 | Rodriguez et al. 2009 | Google earth | Area/Unspecified communities | I | I |
| *Homo sapiens* | Host | Primates | DTUI | 10.95000000 | -73.46700000 | Rodriguez et al. 2009 | Google earth | Area/Unspecified communities | I | I |
| *Homo sapiens* | Host | Primates | DTUI | 10.95000000 | -73.46700000 | Rodriguez et al. 2009 | Google earth | Area/Unspecified communities | I | I |
| *Homo sapiens* | Host | Primates | DTUI | 10.95000000 | -73.46700000 | Rodriguez et al. 2009 | Google earth | Area/Unspecified communities | I | I |
| *Homo sapiens* | Host | Primates | DTUI | 10.95000000 | -73.46700000 | Rodriguez et al. 2009 | Google earth | Area/Unspecified communities | I | I |
| *Homo sapiens* | Host | Primates | DTUI | 10.95000000 | -73.46700000 | Rodriguez et al. 2009 | Google earth | Area/Unspecified communities | I | I |
| *Homo sapiens* | Host | Primates | DTUI | 10.95000000 | -73.46700000 | Rodriguez et al. 2009 | Google earth | Area/Unspecified communities | I | I |
| *Homo sapiens* | Host | Primates | DTUI | 10.95000000 | -73.46700000 | Rodriguez et al. 2009 | Google earth | Area/Unspecified communities | I | I |
| *Homo sapiens* | Host | Primates | DTUI | 10.95000000 | -73.46700000 | Rodriguez et al. 2009 | Google earth | Area/Unspecified communities | I | I |
| *Homo sapiens* | Host | Primates | DTUI | 10.95000000 | -73.46700000 | Rodriguez et al. 2009 | Google earth | Area/Unspecified communities | I | I |
| *Homo sapiens* | Host | Primates | DTUI | 10.95000000 | -73.46700000 | Rodriguez et al. 2009 | Google earth | Area/Unspecified communities | I | I |
| *Homo sapiens* | Host | Primates | DTUI | 10.95000000 | -73.46700000 | Rodriguez et al. 2009 | Google earth | Area/Unspecified communities | I | I |
| *Homo sapiens* | Host | Primates | DTUI | 10.95000000 | -73.46700000 | Rodriguez et al. 2009 | Google earth | Area/Unspecified communities | I | I |
| *Homo sapiens* | Host | Primates | DTUI | 10.95000000 | -73.46700000 | Rodriguez et al. 2009 | Google earth | Area/Unspecified communities | I | I |
| *Homo sapiens* | Host | Primates | DTUI | 10.95000000 | -73.46700000 | Rodriguez et al. 2009 | Google earth | Area/Unspecified communities | I | I |
| *Homo sapiens* | Host | Primates | DTUI | 10.95000000 | -73.46700000 | Rodriguez et al. 2009 | Google earth | Area/Unspecified communities | I | I |
| *Homo sapiens* | Host | Primates | DTUI | 10.95000000 | -73.46700000 | Rodriguez et al. 2009 | Google earth | Area/Unspecified communities | I | I |
| *Homo sapiens* | Host | Primates | DTUI | 10.95000000 | -73.46700000 | Rodriguez et al. 2009 | Google earth | Area/Unspecified communities | I | I |
| *Homo sapiens* | Host | Primates | DTUI | 10.95000000 | -73.46700000 | Rodriguez et al. 2009 | Google earth | Area/Unspecified communities | I | I |
| *Homo sapiens* | Host | Primates | DTUI | 10.95000000 | -73.46700000 | Rodriguez et al. 2009 | Google earth | Area/Unspecified communities | I | I |
| *Homo sapiens* | Host | Primates | DTUI | 10.95000000 | -73.46700000 | Rodriguez et al. 2009 | Google earth | Area/Unspecified communities | I | I |
| *Homo sapiens* | Host | Primates | DTUI | 10.95000000 | -73.46700000 | Rodriguez et al. 2009 | Google earth | Area/Unspecified communities | I | I |
| *Homo sapiens* | Host | Primates | DTUI | 10.95000000 | -73.46700000 | Rodriguez et al. 2009 | Google earth | Area/Unspecified communities | I | I |
| *Homo sapiens* | Host | Primates | DTUI | 10.95000000 | -73.46700000 | Rodriguez et al. 2009 | Google earth | Area/Unspecified communities | I | I |
| *Homo sapiens* | Host | Primates | DTUI | 10.95000000 | -73.46700000 | Rodriguez et al. 2009 | Google earth | Area/Unspecified communities | I | I |
| *Homo sapiens* | Host | Primates | DTUI | 10.95000000 | -73.46700000 | Rodriguez et al. 2009 | Google earth | Area/Unspecified communities | I | I |
| *Homo sapiens* | Host | Primates | DTUI | -17.38000000 | -66.16000000 | Messenger et al. 2012 | Author | NA | I | I |
| *Homo sapiens* | Host | Primates | DTUI | -17.38000000 | -66.16000000 | Messenger et al. 2012 | Author | NA | I | I |
| *Homo sapiens* | Host | Primates | DTUI | -17.38000000 | -66.16000000 | Messenger et al. 2012 | Author | NA | I | I |
| *Homo sapiens* | Host | Primates | DTUI | 6.96300000 | -73.42000000 | Ramirez et al. 2013 | Author | NA | I | I |
| *Homo sapiens* | Host | Primates | DTUI | 6.64400000 | -73.65400000 | Ramirez et al. 2013 | Author | NA | I | I |
| *Homo sapiens* | Host | Primates | DTUI | 5.64000000 | -72.89900000 | Ramirez et al. 2013 | Author | NA | I | I |
| *Homo sapiens* | Host | Primates | DTUI | 5.13000000 | -73.11900000 | Ramirez et al. 2013 | Author | NA | I | I |
| *Homo sapiens* | Host | Primates | DTUI | 5.92000000 | -73.50000000 | Ramirez et al. 2013 | Author | NA | I | I |
| *Homo sapiens* | Host | Primates | DTUI | 22.15900000 | -100.99000000 | Zumaya-Estrada et al. 2012 | Author | NA | I | I |
| *Homo sapiens* | Host | Primates | DTUI | 17.05400000 | -96.71400000 | Zumaya-Estrada et al. 2012 | Author | NA | I | I |
| *Homo sapiens* | Host | Primates | DTUI | 10.46000000 | -63.61000000 | Messenger et al. 2012 | Author | NA | I | I |
| *Homo sapiens* | Host | Primates | DTUI | 8.59000000 | -71.23000000 | Messenger et al. 2012 | Author | NA | I | I |
| *Homo sapiens* | Host | Primates | DTUI | 10.23300000 | -69.86600000 | Messenger et al. 2012 | Author | NA | I | I |
| *Homo sapiens* | Host | Primates | DTUI | 9.08400000 | -69.10300000 | Messenger et al. 2012 | Author | NA | I | I |
| *Homo sapiens* | Host | Primates | DTUI | 10.26600000 | -66.48500000 | Zumaya-Estrada et al. 2012 | Author | NA | I | I |
| *Homo sapiens* | Host | Primates | DTUI | 10.08000000 | -66.44900000 | Zumaya-Estrada et al. 2012 | Author | NA | I | I |
| *Homo sapiens* | Host | Primates | DTUI | 10.40600000 | -63.29800000 | Zumaya-Estrada et al. 2012 | Author | NA | I | I |
| *Homo sapiens* | Host | Primates | DTUI | 10.50000000 | -66.95100000 | Zumaya-Estrada et al. 2012 | Author | NA | I | I |
| *Homo sapiens* | Host | Primates | DTUI | -27.03300000 | -48.48300000 | Marcili et al. 2009b | Google earth | Locality | I | I |
| *Homo sapiens* | Host | Primates | DTUI | -0.26700000 | -63.20000000 | Marcili et al. 2009b | Google earth | Locality | I | I |
| *Homo sapiens* | Host | Primates | DTUI | -0.26700000 | -63.20000000 | Marcili et al. 2009b | Google earth | Locality | I | I |
| *Homo sapiens* | Host | Primates | DTUI | -1.08300000 | -47.40000000 | Marcili et al. 2009b | Google earth | Locality | I | I |
| *Homo sapiens* | Host | Primates | DTUI | -27.03300000 | -48.48300000 | Marcili et al. 2009b | Google earth | Locality | I | I |
| *Homo sapiens* | Host | Primates | DTUI | -1.35000000 | -48.36700000 | Marcili et al. 2009b | Google earth | Locality | I | I |
| *Homo sapiens* | Host | Primates | DTUI | -0.03300000 | -51.06700000 | Marcili et al. 2009b | Google earth | Locality | I | I |
| *Homo sapiens* | Host | Primates | DTUI | -0.03300000 | -51.06700000 | Marcili et al. 2009b | Google earth | Locality | I | I |
| *Homo sapiens* | Host | Primates | DTUI | -0.03300000 | -51.06700000 | Marcili et al. 2009b | Google earth | Locality | I | I |
| *Homo sapiens* | Host | Primates | DTUI | -0.83000000 | -48.90000000 | Marcili et al. 2009b | Google earth | Locality | I | I |
| *Homo sapiens* | Host | Primates | DTUI | -0.83000000 | -48.90000000 | Marcili et al. 2009b | Google earth | Locality | I | I |
| *Homo sapiens* | Host | Primates | DTUI | -0.83000000 | -48.90000000 | Marcili et al. 2009b | Google earth | Locality | I | I |
| *Homo sapiens* | Host | Primates | DTUI | -1.06700000 | -50.46700000 | Marcili et al. 2009b | Google earth | Locality | I | I |
| *Homo sapiens* | Host | Primates | DTUI | -1.56700000 | -48.58300000 | Marcili et al. 2009b | Google earth | Locality | I | I |
| *Homo sapiens* | Host | Primates | DTUI | -2.31600000 | -50.25000000 | Marcili et al. 2009b | Google earth | Locality | I | I |
| *Homo sapiens* | Host | Primates | DTUI | 9.13000000 | -75.05000000 | Mejia-Jaramillo et al. 2009 | Google earth | Department/State | I | I |
| *Homo sapiens* | Host | Primates | DTUI | 5.75000000 | -71.56700000 | Mejia-Jaramillo et al. 2009 | Google earth | Department/State | I | I |
| *Homo sapiens* | Host | Primates | DTUI | 8.58300000 | -63.95000000 | Carrasco et al. 2012 | Google earth | State | I | I |
| *Homo sapiens* | Host | Primates | DTUI | 8.58300000 | -63.95000000 | Carrasco et al. 2012 | Google earth | State | I | I |
| *Homo sapiens* | Host | Primates | DTUI | 8.30000000 | -70.05000000 | Carrasco et al. 2012 | Google earth | State | I | I |
| *Homo sapiens* | Host | Primates | DTUI | 8.30000000 | -70.05000000 | Carrasco et al. 2012 | Google earth | State | I | I |
| *Homo sapiens* | Host | Primates | DTUI | 10.11670000 | -68.03300000 | Carrasco et al. 2012 | Google earth | State | I | I |
| *Homo sapiens* | Host | Primates | DTUI | 9.36700000 | -68.03400000 | Carrasco et al. 2012 | Google earth | State | I | I |
| *Homo sapiens* | Host | Primates | DTUI | 9.36700000 | -68.03400000 | Carrasco et al. 2012 | Google earth | State | I | I |
| *Homo sapiens* | Host | Primates | DTUI | 10.05000000 | -66.95000000 | Carrasco et al. 2012 | Google earth | State | I | I |
| *Homo sapiens* | Host | Primates | DTUI | 10.05000000 | -66.95000000 | Carrasco et al. 2012 | Google earth | State | I | I |
| *Homo sapiens* | Host | Primates | DTUI | 10.05000000 | -66.95000000 | Carrasco et al. 2012 | Google earth | State | I | I |
| *Homo sapiens* | Host | Primates | DTUI | 10.05000000 | -66.95000000 | Carrasco et al. 2012 | Google earth | State | I | I |
| *Homo sapiens* | Host | Primates | DTUI | 10.05000000 | -66.95000000 | Carrasco et al. 2012 | Google earth | State | I | I |
| *Homo sapiens* | Host | Primates | DTUI | 10.05000000 | -66.95000000 | Carrasco et al. 2012 | Google earth | State | I | I |
| *Homo sapiens* | Host | Primates | DTUI | 10.05000000 | -66.95000000 | Carrasco et al. 2012 | Google earth | State | I | I |
| *Homo sapiens* | Host | Primates | DTUI | 10.05000000 | -66.95000000 | Carrasco et al. 2012 | Google earth | State | I | I |
| *Homo sapiens* | Host | Primates | DTUI | 10.05000000 | -66.95000000 | Carrasco et al. 2012 | Google earth | State | I | I |
| *Homo sapiens* | Host | Primates | DTUI | 10.05000000 | -66.95000000 | Carrasco et al. 2012 | Google earth | State | I | I |
| *Homo sapiens* | Host | Primates | DTUI | 10.05000000 | -66.95000000 | Carrasco et al. 2012 | Google earth | State | I | I |
| *Homo sapiens* | Host | Primates | DTUI | 10.05000000 | -66.95000000 | Carrasco et al. 2012 | Google earth | State | I | I |
| *Homo sapiens* | Host | Primates | DTUI | 10.05000000 | -66.95000000 | Carrasco et al. 2012 | Google earth | State | I | I |
| *Homo sapiens* | Host | Primates | DTUI | 10.05000000 | -66.95000000 | Carrasco et al. 2012 | Google earth | State | I | I |
| *Homo sapiens* | Host | Primates | DTUI | 10.05000000 | -66.95000000 | Carrasco et al. 2012 | Google earth | State | I | I |
| *Homo sapiens* | Host | Primates | DTUI | 10.05000000 | -66.95000000 | Carrasco et al. 2012 | Google earth | State | I | I |
| *Homo sapiens* | Host | Primates | DTUI | 8.73400000 | -66.23400000 | Carrasco et al. 2012 | Google earth | State | I | I |
| *Homo sapiens* | Host | Primates | DTUI | 8.73400000 | -66.23400000 | Carrasco et al. 2012 | Google earth | State | I | I |
| *Homo sapiens* | Host | Primates | DTUI | 8.73400000 | -66.23400000 | Carrasco et al. 2012 | Google earth | State | I | I |
| *Homo sapiens* | Host | Primates | DTUI | 8.73400000 | -66.23400000 | Carrasco et al. 2012 | Google earth | State | I | I |
| *Homo sapiens* | Host | Primates | DTUI | 8.73400000 | -66.23400000 | Carrasco et al. 2012 | Google earth | State | I | I |
| *Homo sapiens* | Host | Primates | DTUI | 8.73400000 | -66.23400000 | Carrasco et al. 2012 | Google earth | State | I | I |
| *Homo sapiens* | Host | Primates | DTUI | 10.15000000 | -69.85000000 | Carrasco et al. 2012 | Google earth | State | I | I |
| *Homo sapiens* | Host | Primates | DTUI | 10.15000000 | -69.85000000 | Carrasco et al. 2012 | Google earth | State | I | I |
| *Homo sapiens* | Host | Primates | DTUI | 10.15000000 | -69.85000000 | Carrasco et al. 2012 | Google earth | State | I | I |
| *Homo sapiens* | Host | Primates | DTUI | 10.15000000 | -69.85000000 | Carrasco et al. 2012 | Google earth | State | I | I |
| *Homo sapiens* | Host | Primates | DTUI | 10.15000000 | -69.85000000 | Carrasco et al. 2012 | Google earth | State | I | I |
| *Homo sapiens* | Host | Primates | DTUI | 10.15000000 | -69.85000000 | Carrasco et al. 2012 | Google earth | State | I | I |
| *Homo sapiens* | Host | Primates | DTUI | 10.15000000 | -69.85000000 | Carrasco et al. 2012 | Google earth | State | I | I |
| *Homo sapiens* | Host | Primates | DTUI | 10.15000000 | -69.85000000 | Carrasco et al. 2012 | Google earth | State | I | I |
| *Homo sapiens* | Host | Primates | DTUI | 8.60000000 | -71.15000000 | Carrasco et al. 2012 | Google earth | State | I | I |
| *Homo sapiens* | Host | Primates | DTUI | 10.25000000 | -66.41670000 | Carrasco et al. 2012 | Google earth | State | I | I |
| *Homo sapiens* | Host | Primates | DTUI | 10.25000000 | -66.41670000 | Carrasco et al. 2012 | Google earth | State | I | I |
| *Homo sapiens* | Host | Primates | DTUI | 10.25000000 | -66.41670000 | Carrasco et al. 2012 | Google earth | State | I | I |
| *Homo sapiens* | Host | Primates | DTUI | 10.25000000 | -66.41670000 | Carrasco et al. 2012 | Google earth | State | I | I |
| *Homo sapiens* | Host | Primates | DTUI | 10.25000000 | -66.41670000 | Carrasco et al. 2012 | Google earth | State | I | I |
| *Homo sapiens* | Host | Primates | DTUI | 10.25000000 | -66.41670000 | Carrasco et al. 2012 | Google earth | State | I | I |
| *Homo sapiens* | Host | Primates | DTUI | 9.31670000 | -63.00000000 | Carrasco et al. 2012 | Google earth | State | I | I |
| *Homo sapiens* | Host | Primates | DTUI | 9.01600000 | -69.08300000 | Carrasco et al. 2012 | Google earth | State | I | I |
| *Homo sapiens* | Host | Primates | DTUI | 9.01600000 | -69.08300000 | Carrasco et al. 2012 | Google earth | State | I | I |
| *Homo sapiens* | Host | Primates | DTUI | 9.01600000 | -69.08300000 | Carrasco et al. 2012 | Google earth | State | I | I |
| *Homo sapiens* | Host | Primates | DTUI | 10.41670000 | -63.28300000 | Carrasco et al. 2012 | Google earth | State | I | I |
| *Homo sapiens* | Host | Primates | DTUI | 10.41670000 | -63.28300000 | Carrasco et al. 2012 | Google earth | State | I | I |
| *Homo sapiens* | Host | Primates | DTUI | 10.41670000 | -63.28300000 | Carrasco et al. 2012 | Google earth | State | I | I |
| *Homo sapiens* | Host | Primates | DTUI | 10.41670000 | -63.28300000 | Carrasco et al. 2012 | Google earth | State | I | I |
| *Homo sapiens* | Host | Primates | DTUI | 10.41670000 | -63.28300000 | Carrasco et al. 2012 | Google earth | State | I | I |
| *Homo sapiens* | Host | Primates | DTUI | 7.90000000 | -72.13400000 | Carrasco et al. 2012 | Google earth | State | I | I |
| *Homo sapiens* | Host | Primates | DTUI | 7.90000000 | -72.13400000 | Carrasco et al. 2012 | Google earth | State | I | I |
| *Homo sapiens* | Host | Primates | DTUI | 7.90000000 | -72.13400000 | Carrasco et al. 2012 | Google earth | State | I | I |
| *Homo sapiens* | Host | Primates | DTUI | 7.90000000 | -72.13400000 | Carrasco et al. 2012 | Google earth | State | I | I |
| *Homo sapiens* | Host | Primates | DTUI | 7.90000000 | -72.13400000 | Carrasco et al. 2012 | Google earth | State | I | I |
| *Homo sapiens* | Host | Primates | DTUI | 7.90000000 | -72.13400000 | Carrasco et al. 2012 | Google earth | State | I | I |
| *Homo sapiens* | Host | Primates | DTUI | 7.90000000 | -72.13400000 | Carrasco et al. 2012 | Google earth | State | I | I |
| *Homo sapiens* | Host | Primates | DTUI | 7.90000000 | -72.13400000 | Carrasco et al. 2012 | Google earth | State | I | I |
| *Homo sapiens* | Host | Primates | DTUI | 7.90000000 | -72.13400000 | Carrasco et al. 2012 | Google earth | State | I | I |
| *Homo sapiens* | Host | Primates | DTUI | 9.36700000 | -70.41600000 | Carrasco et al. 2012 | Google earth | State | I | I |
| *Homo sapiens* | Host | Primates | DTUI | 9.36700000 | -70.41600000 | Carrasco et al. 2012 | Google earth | State | I | I |
| *Homo sapiens* | Host | Primates | DTUI | 9.36700000 | -70.41600000 | Carrasco et al. 2012 | Google earth | State | I | I |
| *Homo sapiens* | Host | Primates | DTUI | 9.36700000 | -70.41600000 | Carrasco et al. 2012 | Google earth | State | I | I |
| *Homo sapiens* | Host | Primates | DTUI | 9.36700000 | -70.41600000 | Carrasco et al. 2012 | Google earth | State | I | I |
| *Homo sapiens* | Host | Primates | DTUI | 9.36700000 | -70.41600000 | Carrasco et al. 2012 | Google earth | State | I | I |
| *Homo sapiens* | Host | Primates | DTUI | 10.51600000 | -67.06700000 | Carrasco et al. 2012 | Google earth | State | I | I |
| *Homo sapiens* | Host | Primates | DTUI | 10.51600000 | -67.06700000 | Carrasco et al. 2012 | Google earth | State | I | I |
| *Homo sapiens* | Host | Primates | DTUI | 10.51600000 | -67.06700000 | Carrasco et al. 2012 | Google earth | State | I | I |
| *Homo sapiens* | Host | Primates | DTUI | 10.41600000 | -68.70000000 | Carrasco et al. 2012 | Google earth | State | I | I |
| *Homo sapiens* | Host | Primates | DTUI | 10.41600000 | -68.70000000 | Carrasco et al. 2012 | Google earth | State | I | I |
| *Homo sapiens* | Host | Primates | DTUI | 10.41600000 | -68.70000000 | Carrasco et al. 2012 | Google earth | State | I | I |
| *Homo sapiens* | Host | Primates | DTUI | 10.41600000 | -68.70000000 | Carrasco et al. 2012 | Google earth | State | I | I |
| *Homo sapiens* | Host | Primates | DTUI | 14.18300000 | -90.36700000 | Higo et al. 2004 | Google earth | Department/State | I | I |
| *Homo sapiens* | Host | Primates | DTUI | 14.18300000 | -90.36700000 | Higo et al. 2004 | Google earth | Department/State | I | I |
| *Homo sapiens* | Host | Primates | DTUI | 14.18300000 | -90.36700000 | Higo et al. 2004 | Google earth | Department/State | I | I |
| *Homo sapiens* | Host | Primates | DTUI | 14.18300000 | -90.36700000 | Higo et al. 2004 | Google earth | Department/State | I | I |
| *Homo sapiens* | Host | Primates | DTUI | 14.18300000 | -90.36700000 | Higo et al. 2004 | Google earth | Department/State | I | I |
| *Homo sapiens* | Host | Primates | DTUI | 14.18300000 | -90.36700000 | Higo et al. 2004 | Google earth | Department/State | I | I |
| *Homo sapiens* | Host | Primates | DTUI | 14.18300000 | -90.36700000 | Higo et al. 2004 | Google earth | Department/State | I | I |
| *Homo sapiens* | Host | Primates | DTUI | 14.18300000 | -90.36700000 | Higo et al. 2004 | Google earth | Department/State | I | I |
| *Homo sapiens* | Host | Primates | DTUI | 15.06700000 | -89.43000000 | Higo et al. 2004 | Google earth | Department/State | I | I |
| *Homo sapiens* | Host | Primates | DTUI | 15.06700000 | -89.43000000 | Higo et al. 2004 | Google earth | Department/State | I | I |
| *Homo sapiens* | Host | Primates | DTUI | 15.06700000 | -89.43300000 | Higo et al. 2004 | Google earth | Department/State | I | I |
| *Homo sapiens* | Host | Primates | DTUI | 15.06700000 | -89.43300000 | Higo et al. 2004 | Google earth | Department/State | I | I |
| *Homo sapiens* | Host | Primates | DTUI | 15.06700000 | -89.43300000 | Higo et al. 2004 | Google earth | Department/State | I | I |
| *Homo sapiens* | Host | Primates | DTUI | 15.06700000 | -89.43300000 | Higo et al. 2004 | Google earth | Department/State | I | I |
| *Homo sapiens* | Host | Primates | DTUI | 15.06700000 | -89.43300000 | Higo et al. 2004 | Google earth | Department/State | I | I |
| *Homo sapiens* | Host | Primates | DTUI | 15.06700000 | -89.43300000 | Higo et al. 2004 | Google earth | Department/State | I | I |
| *Homo sapiens* | Host | Primates | DTUI | 15.06700000 | -89.43300000 | Higo et al. 2004 | Google earth | Department/State | I | I |
| *Homo sapiens* | Host | Primates | DTUI | 15.06700000 | -89.43300000 | Higo et al. 2004 | Google earth | Department/State | I | I |
| *Homo sapiens* | Host | Primates | DTUI | 15.06700000 | -89.43300000 | Higo et al. 2004 | Google earth | Department/State | I | I |
| *Homo sapiens* | Host | Primates | DTUI | 14.18300000 | -90.36700000 | Higo et al. 2004 | Google earth | Department/State | I | I |
| *Homo sapiens* | Host | Primates | DTUI | 15.06700000 | -89.43300000 | Higo et al. 2004 | Google earth | Department/State | I | I |
| *Homo sapiens* | Host | Primates | DTUI | 20.65000000 | -103.33000000 | Higo et al. 2004 | Google earth | Department/State | I | I |
| *Homo sapiens* | Host | Primates | DTUI | 14.18300000 | -90.36700000 | Higo et al. 2004 | Google earth | Department/State | I | I |
| *Homo sapiens* | Host | Primates | DTUI | 14.18300000 | -90.36700000 | Higo et al. 2004 | Google earth | Department/State | I | I |
| *Homo sapiens* | Host | Primates | DTUI | 15.06700000 | -89.43300000 | Higo et al. 2004 | Google earth | Department/State | I | I |
| *Homo sapiens* | Host | Primates | DTUI | 15.06700000 | -89.43300000 | Higo et al. 2004 | Google earth | Department/State | I | I |
| *Homo sapiens* | Host | Primates | DTUI | 27.80000000 | -97.38300000 | Roellig et al. 2013 | Google earth | Community | I | I |
| *Homo sapiens* | Host | Primates | DTUI | 36.76600000 | -119.41600000 | Roellig et al. 2013 | Google earth | Community | I | I |
| *Homo sapiens* | Host | Primates | DTUI | 29.35000000 | -100.91600000 | Roellig et al. 2013 | Google earth | Community | I | I |
| *Homo sapiens* | Host | Primates | DTUI | -5.70000000 | -37.55000000 | Camara et al. 2010 | Google earth | Area/Unspecified communities | I | I |
| *Homo sapiens* | Host | Primates | DTUI | 17.05410000 | -96.71300000 | Martinez et al. 2013 | Google earth | Department/State | I | I |
| *Homo sapiens* | Host | Primates | DTUI | 17.05410000 | -96.71300000 | Martinez et al. 2013 | Google earth | Department/State | I | I |
| *Homo sapiens* | Host | Primates | DTUI | 17.05410000 | -96.71300000 | Martinez et al. 2013 | Google earth | Department/State | I | I |
| *Homo sapiens* | Host | Primates | DTUI | 20.91670000 | -101.16100000 | Martinez et al. 2013 | Google earth | Department/State | I | I |
| *Homo sapiens* | Host | Primates | DTUI | 20.91670000 | -101.16100000 | Martinez et al. 2013 | Google earth | Department/State | I | I |
| *Homo sapiens* | Host | Primates | DTUI | 19.17360000 | -96.13410000 | Martinez et al. 2013 | Google earth | Department/State | I | I |
| *Homo sapiens* | Host | Primates | DTUI | 19.17360000 | -96.13410000 | Martinez et al. 2013 | Google earth | Department/State | I | I |
| *Homo sapiens* | Host | Primates | DTUI | 17.43910000 | -93.72830000 | Martinez et al. 2013 | Google earth | Department/State | I | I |
| *Homo sapiens* | Host | Primates | DTUI | 20.70970000 | -89.09410000 | Martinez et al. 2013 | Google earth | Department/State | I | I |
| *Homo sapiens* | Host | Primates | DTUI | 20.70970000 | -89.09410000 | Martinez et al. 2013 | Google earth | Department/State | I | I |
| *Homo sapiens* | Host | Primates | DTUI | 18.68110000 | -96.10100000 | Martinez et al. 2013 | Google earth | Department/State | I | I |
| *Homo sapiens* | Host | Primates | DTUI | 18.68110000 | -96.10100000 | Martinez et al. 2013 | Google earth | Department/State | I | I |
| *Homo sapiens* | Host | Primates | DTUI | 18.68110000 | -96.10100000 | Martinez et al. 2013 | Google earth | Department/State | I | I |
| *Homo sapiens* | Host | Primates | DTUI | 18.68110000 | -96.10100000 | Martinez et al. 2013 | Google earth | Department/State | I | I |
| *Homo sapiens* | Host | Primates | DTUI | 18.68110000 | -96.10100000 | Martinez et al. 2013 | Google earth | Department/State | I | I |
| *Homo sapiens* | Host | Primates | DTUI | 18.68110000 | -96.10100000 | Martinez et al. 2013 | Google earth | Department/State | I | I |
| *Homo sapiens* | Host | Primates | DTUI | 18.68110000 | -96.10100000 | Martinez et al. 2013 | Google earth | Department/State | I | I |
| *Homo sapiens* | Host | Primates | DTUI | 18.68110000 | -96.10100000 | Martinez et al. 2013 | Google earth | Department/State | I | I |
| *Homo sapiens* | Host | Primates | DTUI | 20.65940000 | -103.34917000 | Martinez et al. 2013 | Google earth | Department/State | I | I |
| *Homo sapiens* | Host | Primates | DTUI | 20.65940000 | -103.34917000 | Martinez et al. 2013 | Google earth | Department/State | I | I |
| *Homo sapiens* | Host | Primates | DTUII | -5.70000000 | -37.55000000 | Camara et al. 2010 | Google earth | Locality | II | II |
| *Homo sapiens* | Host | Primates | DTUII | -5.70000000 | -37.55000000 | Camara et al. 2010 | Google earth | Locality | II | II |
| *Homo sapiens* | Host | Primates | DTUII | -5.70000000 | -37.55000000 | Camara et al. 2010 | Google earth | Locality | II | II |
| *Homo sapiens* | Host | Primates | DTUII | -5.70000000 | -37.55000000 | Camara et al. 2010 | Google earth | Locality | II | II |
| *Homo sapiens* | Host | Primates | DTUII | -5.70000000 | -37.55000000 | Camara et al. 2010 | Google earth | Locality | II | II |
| *Homo sapiens* | Host | Primates | DTUII | -5.70000000 | -37.55000000 | Camara et al. 2010 | Google earth | Locality | II | II |
| *Homo sapiens* | Host | Primates | DTUII | -5.70000000 | -37.55000000 | Camara et al. 2010 | Google earth | Locality | II | II |
| *Homo sapiens* | Host | Primates | DTUIII | -4.86700000 | -66.90000000 | Marcili et al. 2009b | Google earth | Locality | II | III |
| *Homo sapiens* | Host | Primates | DTUIV | -27.03300000 | -48.48200000 | Marcili et al. 2009b | Google earth | Locality | II | III |
| *Homo sapiens* | Host | Primates | DTUIV | -0.26700000 | -63.20000000 | Marcili et al. 2009b | Google earth | Locality | II | III |
| *Homo sapiens* | Host | Primates | DTUIV | -0.03300000 | -51.06700000 | Marcili et al. 2009b | Google earth | Locality | II | III |
| *Homo sapiens* | Host | Primates | DTUIV | -0.03300000 | -51.06700000 | Marcili et al. 2009b | Google earth | Locality | II | III |
| *Homo sapiens* | Host | Primates | DTUIV | -2.45000000 | -54.70000000 | Marcili et al. 2009b | Google earth | Locality | II | III |
| *Homo sapiens* | Host | Primates | DTUIV | -1.15000000 | -46.73300000 | Marcili et al. 2009b | Google earth | Locality | II | III |
| *Homo sapiens* | Host | Primates | DTUIV | -27.03300000 | -48.48300000 | Marcili et al. 2009b | Google earth | Locality | II | III |
| *Homo sapiens* | Host | Primates | DTUIV | 8.58300000 | -63.95000000 | Carrasco et al. 2012 | Google earth | State | II | III |
| *Homo sapiens* | Host | Primates | DTUIV | 8.58300000 | -63.95000000 | Carrasco et al. 2012 | Google earth | State | II | III |
| *Homo sapiens* | Host | Primates | DTUIV | 8.58300000 | -63.95000000 | Carrasco et al. 2012 | Google earth | State | II | III |
| *Homo sapiens* | Host | Primates | DTUIV | 8.58300000 | -63.95000000 | Carrasco et al. 2012 | Google earth | State | II | III |
| *Homo sapiens* | Host | Primates | DTUIV | 10.21300000 | -67.28300000 | Carrasco et al. 2012 | Google earth | State | II | III |
| *Homo sapiens* | Host | Primates | DTUIV | 8.30000000 | -70.05000000 | Carrasco et al. 2012 | Google earth | State | II | III |
| *Homo sapiens* | Host | Primates | DTUIV | 9.36700000 | -68.03400000 | Carrasco et al. 2012 | Google earth | State | II | III |
| *Homo sapiens* | Host | Primates | DTUIV | 8.73400000 | -66.23400000 | Carrasco et al. 2012 | Google earth | State | II | III |
| *Homo sapiens* | Host | Primates | DTUIV | 8.73400000 | -66.23400000 | Carrasco et al. 2012 | Google earth | State | II | III |
| *Homo sapiens* | Host | Primates | DTUIV | 8.73400000 | -66.23400000 | Carrasco et al. 2012 | Google earth | State | II | III |
| *Homo sapiens* | Host | Primates | DTUIV | 10.15000000 | -69.85000000 | Carrasco et al. 2012 | Google earth | State | II | III |
| *Homo sapiens* | Host | Primates | DTUIV | 10.15000000 | -69.85000000 | Carrasco et al. 2012 | Google earth | State | II | III |
| *Homo sapiens* | Host | Primates | DTUIV | 10.25000000 | -66.41670000 | Carrasco et al. 2012 | Google earth | State | II | III |
| *Homo sapiens* | Host | Primates | DTUIV | 9.01600000 | -69.08300000 | Carrasco et al. 2012 | Google earth | State | II | III |
| *Homo sapiens* | Host | Primates | DTUIV | 9.01600000 | -69.08300000 | Carrasco et al. 2012 | Google earth | State | II | III |
| *Homo sapiens* | Host | Primates | DTUIV | 9.01600000 | -69.08300000 | Carrasco et al. 2012 | Google earth | State | II | III |
| *Homo sapiens* | Host | Primates | DTUIV | 9.01600000 | -69.08300000 | Carrasco et al. 2012 | Google earth | State | II | III |
| *Homo sapiens* | Host | Primates | DTUIV | 10.41670000 | -63.28300000 | Carrasco et al. 2012 | Google earth | State | II | III |
| *Homo sapiens* | Host | Primates | DTUIV | 7.90000000 | -72.13400000 | Carrasco et al. 2012 | Google earth | State | II | III |
| *Homo sapiens* | Host | Primates | DTUIV | 9.36700000 | -70.41600000 | Carrasco et al. 2012 | Google earth | State | II | III |
| *Homo sapiens* | Host | Primates | DTUIV | 14.18300000 | -90.36700000 | Higo et al. 2004 | Google earth | Department/State | II | III |
| *Homo sapiens* | Host | Primates | DTUIV | 15.06700000 | -89.43300000 | Higo et al. 2004 | Google earth | Department/State | II | III |
| *Homo sapiens* | Host | Primates | DTUV | -26.58300000 | -60.95000000 | Lauthier et al. 2012 | Google earth | Province | II | ND |
| *Homo sapiens* | Host | Primates | DTUV | -26.58300000 | -60.95000000 | Lauthier et al. 2012 | Google earth | Province | II | ND |
| *Homo sapiens* | Host | Primates | DTUV | -26.58300000 | -60.95000000 | Lauthier et al. 2012 | Google earth | Province | II | ND |
| *Homo sapiens* | Host | Primates | DTUVI | 18.59182063 | -89.41638242 | Lopez-Cancino et al. 2015 | Author | NA | II | ND |
| *Homo sapiens* | Host | Primates | DTUVI | 18.59182063 | -89.41638242 | Lopez-Cancino et al. 2015 | Author | NA | II | ND |
| *Homo sapiens* | Host | Primates | DTUI | 10.49777778 | -66.84972222 | Cura et al. 2015 | Google earth | Locality | I | I |
| *Homo sapiens* | Host | Primates | DTUI | 10.49777778 | -66.84972222 | Cura et al. 2015 | Google earth | Locality | I | I |
| *Homo sapiens* | Host | Primates | DTUI | 14.86111111 | -92.44833333 | Cura et al. 2015 | Google earth | Locality | I | I |
| *Homo sapiens* | Host | Primates | DTUI | -28.471389 | -65.787778 | Cura et al. 2015 | Google earth | Province | I | I |
| *Homo sapiens* | Host | Primates | DTUI | -27.425556 | -59.024444 | Cura et al. 2015 | Google earth | Province | I | I |
| *Homo sapiens* | Host | Primates | DTUI | -20.024722 | -64.148056 | Cura et al. 2015 | Google earth | Department/State | I | I |
| *Homo sapiens* | Host | Primates | DTUI | -20.024722 | -64.148056 | Cura et al. 2015 | Google earth | Department/State | I | I |
| *Homo sapiens* | Host | Primates | DTUI | -17.414167 | -66.165278 | Cura et al. 2015 | Google earth | Locality | I | I |
| *Homo sapiens* | Host | Primates | DTUI | -17.414167 | -66.165278 | Cura et al. 2015 | Google earth | Locality | I | I |
| *Homo sapiens* | Host | Primates | DTUI | -17.414167 | -66.165278 | Cura et al. 2015 | Google earth | Locality | I | I |
| *Homo sapiens* | Host | Primates | DTUI | -17.414167 | -66.165278 | Cura et al. 2015 | Google earth | Locality | I | I |
| *Homo sapiens* | Host | Primates | DTUI | -17.414167 | -66.165278 | Cura et al. 2015 | Google earth | Locality | I | I |
| *Homo sapiens* | Host | Primates | DTUI | -17.414167 | -66.165278 | Cura et al. 2015 | Google earth | Locality | I | I |
| *Homo sapiens* | Host | Primates | DTUI | -17.414167 | -66.165278 | Cura et al. 2015 | Google earth | Locality | I | I |
| *Homo sapiens* | Host | Primates | DTUI | -17.414167 | -66.165278 | Cura et al. 2015 | Google earth | Locality | I | I |
| *Homo sapiens* | Host | Primates | DTUI | -17.414167 | -66.165278 | Cura et al. 2015 | Google earth | Locality | I | I |
| *Homo sapiens* | Host | Primates | DTUI | 5.480833 | -53.210000 | Cura et al. 2015 | Google earth | Locality | I | I |
| *Homo sapiens* | Host | Primates | DTUI | 5.480833 | -53.210000 | Cura et al. 2015 | Google earth | Locality | I | I |
| *Homo sapiens* | Host | Primates | DTUI | -16.489722 | -64.283889 | Cura et al. 2015 | Google earth | Locality | I | I |
| *Homo sapiens* | Host | Primates | DTUI | -19.572500 | -65.755000 | Cura et al. 2015 | Google earth | Locality | I | I |
| *Homo sapiens* | Host | Primates | DTUI | -24.799722 | -65.415278 | Cura et al. 2015 | Google earth | Province | I | I |
| *Homo sapiens* | Host | Primates | DTUI | -17.814444 | -63.156111 | Cura et al. 2015 | Google earth | Locality | I | I |
| *Homo sapiens* | Host | Primates | DTUI | -17.814444 | -63.156111 | Cura et al. 2015 | Google earth | Locality | I | I |
| *Homo sapiens* | Host | Primates | DTUI | -17.814444 | -63.156111 | Cura et al. 2015 | Google earth | Locality | I | I |
| *Homo sapiens* | Host | Primates | DTUI | -17.814444 | -63.156111 | Cura et al. 2015 | Google earth | Locality | I | I |
| *Homo sapiens* | Host | Primates | DTUI | -17.814444 | -63.156111 | Cura et al. 2015 | Google earth | Locality | I | I |
| *Homo sapiens* | Host | Primates | DTUI | -27.791111 | -64.273611 | Cura et al. 2015 | Google earth | Province | I | I |
| *Homo sapiens* | Host | Primates | DTUI | -19.019444 | -65.261944 | Cura et al. 2015 | Google earth | Locality | I | I |
| *Homo sapiens* | Host | Primates | DTUI | -19.019444 | -65.261944 | Cura et al. 2015 | Google earth | Locality | I | I |
| *Homo sapiens* | Host | Primates | DTUI | -19.019444 | -65.261944 | Cura et al. 2015 | Google earth | Locality | I | I |
| *Homo sapiens* | Host | Primates | DTUI | -19.019444 | -65.261944 | Cura et al. 2015 | Google earth | Locality | I | I |
| *Homo sapiens* | Host | Primates | DTUI | -21.521389 | -64.728333 | Cura et al. 2015 | Google earth | Locality | I | I |
| *Homo sapiens* | Host | Primates | DTUI | -21.521389 | -64.728333 | Cura et al. 2015 | Google earth | Locality | I | I |
| *Homo sapiens* | Host | Primates | DTUII | 6.64361100000 | 73.65361110000 | Zafra et al. 2008 | Google earth | State | II | II |
| *Homo sapiens* | Host | Primates | DTUII | -8.151389 | -42.472500 | Araujo et al. 2011 | Google earth | Locality | II | II |
| *Homo sapiens* | Host | Primates | DTUII | -36.615000 | -64.283889 | Cura et al. 2015 | Google earth | Province | II | II |
| *Homo sapiens* | Host | Primates | DTUII | -23.550556 | -46.633056 | Araujo et al. 2011 | Google earth | Locality | II | II |
| *Homo sapiens* | Host | Primates | DTUIV | -10.823611 | -65.368889 | Cura et al. 2015 | Google earth | Locality | II | III |
| *Homo sapiens* | Host | Primates | DTUIV | -10.823611 | -65.368889 | Cura et al. 2015 | Google earth | Locality | II | III |
| *Homo sapiens* | Host | Primates | DTUIV | -10.823611 | -65.368889 | Cura et al. 2015 | Google earth | Locality | II | III |
| *Homo sapiens* | Host | Primates | DTUIV | -10.823611 | -65.368889 | Cura et al. 2015 | Google earth | Locality | II | III |
| *Homo sapiens* | Host | Primates | DTUIV | -10.823611 | -65.368889 | Cura et al. 2015 | Google earth | Locality | II | III |
| *Homo sapiens* | Host | Primates | DTUIV | -10.823611 | -65.368889 | Cura et al. 2015 | Google earth | Locality | II | III |
| *Homo sapiens* | Host | Primates | DTUV | -34.603611 | -58.381667 | Cura et al. 2015 | Google earth | Locality | II | ND |
| *Homo sapiens* | Host | Primates | DTUV | -28.471389 | -65.787778 | Cura et al. 2015 | Google earth | Province | II | ND |
| *Homo sapiens* | Host | Primates | DTUV | -27.425556 | -59.024444 | Cura et al. 2015 | Google earth | Province | II | ND |
| *Homo sapiens* | Host | Primates | DTUV | -27.425556 | -59.024444 | Cura et al. 2015 | Google earth | Province | II | ND |
| *Homo sapiens* | Host | Primates | DTUV | -27.425556 | -59.024444 | Cura et al. 2015 | Google earth | Province | II | ND |
| *Homo sapiens* | Host | Primates | DTUV | -27.425556 | -59.024444 | Cura et al. 2015 | Google earth | Province | II | ND |
| *Homo sapiens* | Host | Primates | DTUV | -27.425556 | -59.024444 | Cura et al. 2015 | Google earth | Province | II | ND |
| *Homo sapiens* | Host | Primates | DTUV | -27.425556 | -59.024444 | Cura et al. 2015 | Google earth | Province | II | ND |
| *Homo sapiens* | Host | Primates | DTUV | -27.425556 | -59.024444 | Cura et al. 2015 | Google earth | Province | II | ND |
| *Homo sapiens* | Host | Primates | DTUV | -27.425556 | -59.024444 | Cura et al. 2015 | Google earth | Province | II | ND |
| *Homo sapiens* | Host | Primates | DTUV | -27.425556 | -59.024444 | Cura et al. 2015 | Google earth | Province | II | ND |
| *Homo sapiens* | Host | Primates | DTUV | -27.425556 | -59.024444 | Cura et al. 2015 | Google earth | Province | II | ND |
| *Homo sapiens* | Host | Primates | DTUV | -27.425556 | -59.024444 | Cura et al. 2015 | Google earth | Province | II | ND |
| *Homo sapiens* | Host | Primates | DTUV | -27.425556 | -59.024444 | Cura et al. 2015 | Google earth | Province | II | ND |
| *Homo sapiens* | Host | Primates | DTUV | -27.425556 | -59.024444 | Cura et al. 2015 | Google earth | Province | II | ND |
| *Homo sapiens* | Host | Primates | DTUV | -27.425556 | -59.024444 | Cura et al. 2015 | Google earth | Province | II | ND |
| *Homo sapiens* | Host | Primates | DTUV | -27.425556 | -59.024444 | Cura et al. 2015 | Google earth | Province | II | ND |
| *Homo sapiens* | Host | Primates | DTUV | -27.425556 | -59.024444 | Cura et al. 2015 | Google earth | Province | II | ND |
| *Homo sapiens* | Host | Primates | DTUV | -27.425556 | -59.024444 | Cura et al. 2015 | Google earth | Province | II | ND |
| *Homo sapiens* | Host | Primates | DTUV | -27.425556 | -59.024444 | Cura et al. 2015 | Google earth | Province | II | ND |
| *Homo sapiens* | Host | Primates | DTUV | -27.425556 | -59.024444 | Cura et al. 2015 | Google earth | Province | II | ND |
| *Homo sapiens* | Host | Primates | DTUV | -27.425556 | -59.024444 | Cura et al. 2015 | Google earth | Province | II | ND |
| *Homo sapiens* | Host | Primates | DTUV | -27.425556 | -59.024444 | Cura et al. 2015 | Google earth | Province | II | ND |
| *Homo sapiens* | Host | Primates | DTUV | -27.425556 | -59.024444 | Cura et al. 2015 | Google earth | Province | II | ND |
| *Homo sapiens* | Host | Primates | DTUV | -27.425556 | -59.024444 | Cura et al. 2015 | Google earth | Province | II | ND |
| *Homo sapiens* | Host | Primates | DTUV | -27.425556 | -59.024444 | Cura et al. 2015 | Google earth | Province | II | ND |
| *Homo sapiens* | Host | Primates | DTUV | -27.425556 | -59.024444 | Cura et al. 2015 | Google earth | Province | II | ND |
| *Homo sapiens* | Host | Primates | DTUV | -27.469167 | -58.830833 | Cura et al. 2015 | Google earth | Locality | II | ND |
| *Homo sapiens* | Host | Primates | DTUV | -27.469167 | -58.830833 | Cura et al. 2015 | Google earth | Locality | II | ND |
| *Homo sapiens* | Host | Primates | DTUV | -27.469167 | -58.830833 | Cura et al. 2015 | Google earth | Locality | II | ND |
| *Homo sapiens* | Host | Primates | DTUV | -24.184167 | -65.301944 | Cura et al. 2015 | Google earth | Locality | II | ND |
| *Homo sapiens* | Host | Primates | DTUVI | -17.41388889 | -66.16527778 | Cura et al. 2015 | Google earth | Locality | II | ND |
| *Homo sapiens* | Host | Primates | ND | -22.93000 | -51.48300 | Zalloum et al. 2005 | Google earth | Community | II | ND |
| *Homo sapiens* | Host | Primates | ND | -23.53400 | -50.55000 | Zalloum et al. 2005 | Google earth | Community | II | ND |
| *Homo sapiens* | Host | Primates | ND | -23.30000 | -51.01670 | Zalloum et al. 2005 | Google earth | Community | II | ND |
| *Homo sapiens* | Host | Primates | ND | -22.85000 | -51.01670 | Zalloum et al. 2005 | Google earth | Community | II | ND |
| *Lemur spp.* | Host | Primates | DTUIV | 31.70000000 | -81.26700000 | Roellig et al. 2013 | Google earth | Community | II | III |
| *Lemur spp.* | Host | Primates | DTUIV | 31.70000000 | -81.26700000 | Roellig et al. 2013 | Google earth | Community | II | III |
| *Lemur spp.* | Host | Primates | DTUIV | 31.70000000 | -81.26700000 | Roellig et al. 2013 | Google earth | Community | II | III |
| *Leontopithecus chrysomelas* | Host | Primates | DTUII | -14.793333 | -39.046389 | Araujo et al. 2011 | Google earth | Locality | II | II |
| *Leontopithecus rosalia* | Host | Primates | DTUI | -22.906944 | -43.173056 | Araujo et al. 2011 | Google earth | Locality | I | I |
| *Leontopithecus rosalia* | Host | Primates | DTUI | -22.906944 | -43.173056 | Araujo et al. 2011 | Google earth | Locality | I | I |
| *Leontopithecus rosalia* | Host | Primates | DTUI | -22.906944 | -43.173056 | Araujo et al. 2011 | Google earth | Locality | I | I |
| *Leontopithecus rosalia* | Host | Primates | DTUII | -22.906944 | -43.173056 | Araujo et al. 2011 | Google earth | Locality | II | II |
| *Leontopithecus rosalia* | Host | Primates | DTUII | -22.906944 | -43.173056 | Araujo et al. 2011 | Google earth | Locality | II | II |
| *Leontopithecus rosalia* | Host | Primates | DTUII | -22.906944 | -43.173056 | Araujo et al. 2011 | Google earth | Locality | II | II |
| *Leontopithecus rosalia* | Host | Primates | DTUII | -22.906944 | -43.173056 | Araujo et al. 2011 | Google earth | Locality | II | II |
| *Leontopithecus rosalia* | Host | Primates | DTUII | -22.906944 | -43.173056 | Araujo et al. 2011 | Google earth | Locality | II | II |
| *Leontopithecus rosalia* | Host | Primates | DTUII | -22.906944 | -43.173056 | Araujo et al. 2011 | Google earth | Locality | II | II |
| *Macaca fascicularis* | Host | Primates | DTUI | 31.96833333 | -99.90166667 | Cura et al. 2015 | Google earth | State | I | I |
| *Macaca fascicularis* | Host | Primates | DTUI | 31.96833333 | -99.90166667 | Cura et al. 2015 | Google earth | State | I | I |
| *Macaca fascicularis* | Host | Primates | DTUI | 31.96833333 | -99.90166667 | Cura et al. 2015 | Google earth | State | I | I |
| *Macaca fascicularis* | Host | Primates | DTUI | 31.96833333 | -99.90166667 | Cura et al. 2015 | Google earth | State | I | I |
| *Macaca fascicularis* | Host | Primates | DTUI | 31.96833333 | -99.90166667 | Cura et al. 2015 | Google earth | State | I | I |
| *Macaca fascicularis* | Host | Primates | DTUI | 31.96833333 | -99.90166667 | Cura et al. 2015 | Google earth | State | I | I |
| *Macaca fascicularis* | Host | Primates | DTUI | 31.96833333 | -99.90166667 | Cura et al. 2015 | Google earth | State | I | I |
| *Macaca fascicularis* | Host | Primates | DTUIV | 31.96833333 | -99.90166667 | Cura et al. 2015 | Google earth | State | II | III |
| *Macaca fascicularis* | Host | Primates | DTUIV | 31.96833333 | -99.90166667 | Cura et al. 2015 | Google earth | State | II | III |
| *Marmosa cinerea* | Host | Didelphimorphia | DTUI | 0.03000000 | -51.05000000 | Llewellyn et al. 2009b | Author | NA | I | I |
| *Triatoma longipennis* | Vector | Hemiptera | DTUI | 20.09080000 | -101.74020000 | Ibañez-Cervantes et al. 2013 | Google earth | Area/Unspecified communities | I | I |
| *Triatoma longipennis* | Vector | Hemiptera | DTUI | 19.14610000 | -102.21861000 | Ibañez-Cervantes et al. 2013 | Google earth | Area/Unspecified communities | I | I |
| *Triatoma pallidipennis* | Vector | Hemiptera | DTUI | 19.58500000 | -101.75560000 | Ibañez-Cervantes et al. 2013 | Google earth | Area/Unspecified communities | I | I |
| *Triatoma pallidipennis* | Vector | Hemiptera | DTUI | 19.58500000 | -101.75560000 | Ibañez-Cervantes et al. 2013 | Google earth | Area/Unspecified communities | I | I |
| *Triatoma pallidipennis* | Vector | Hemiptera | DTUI | 19.58500000 | -101.75560000 | Ibañez-Cervantes et al. 2013 | Google earth | Area/Unspecified communities | I | I |
| *Triatoma pallidipennis* | Vector | Hemiptera | DTUI | 19.58500000 | -101.75560000 | Ibañez-Cervantes et al. 2013 | Google earth | Area/Unspecified communities | I | I |
| *Triatoma pallidipennis* | Vector | Hemiptera | DTUI | 19.58500000 | -101.75560000 | Ibañez-Cervantes et al. 2013 | Google earth | Area/Unspecified communities | I | I |
| *Triatoma pallidipennis* | Vector | Hemiptera | DTUI | 19.58500000 | -101.75560000 | Ibañez-Cervantes et al. 2013 | Google earth | Area/Unspecified communities | I | I |
| *Triatoma pallidipennis* | Vector | Hemiptera | DTUI | 19.58500000 | -101.75560000 | Ibañez-Cervantes et al. 2013 | Google earth | Area/Unspecified communities | I | I |
| *Triatoma pallidipennis* | Vector | Hemiptera | DTUI | 19.58500000 | -101.75560000 | Ibañez-Cervantes et al. 2013 | Google earth | Area/Unspecified communities | I | I |
| *Triatoma pallidipennis* | Vector | Hemiptera | DTUI | 19.58500000 | -101.75560000 | Ibañez-Cervantes et al. 2013 | Google earth | Area/Unspecified communities | I | I |
| *Triatoma pallidipennis* | Vector | Hemiptera | DTUI | 19.58500000 | -101.75560000 | Ibañez-Cervantes et al. 2013 | Google earth | Area/Unspecified communities | I | I |
| *Triatoma pallidipennis* | Vector | Hemiptera | DTUI | 19.58500000 | -101.75560000 | Ibañez-Cervantes et al. 2013 | Google earth | Area/Unspecified communities | I | I |
| *Triatoma pallidipennis* | Vector | Hemiptera | DTUII | 19.58500000 | -101.75560000 | Ibañez-Cervantes et al. 2013 | Google earth | Area/Unspecified communities | II | II |
| *Triatoma pallidipennis* | Vector | Hemiptera | DTUII | 19.58500000 | -101.75560000 | Ibañez-Cervantes et al. 2013 | Google earth | Area/Unspecified communities | II | II |
| *Triatoma pallidipennis* | Vector | Hemiptera | DTUII | 19.58500000 | -101.75560000 | Ibañez-Cervantes et al. 2013 | Google earth | Area/Unspecified communities | II | II |
| *Triatoma pallidipennis* | Vector | Hemiptera | DTUII | 19.58500000 | -101.75560000 | Ibañez-Cervantes et al. 2013 | Google earth | Area/Unspecified communities | II | II |
| *Triatoma pallidipennis* | Vector | Hemiptera | DTUIII | 19.58500000 | -101.75560000 | Ibañez-Cervantes et al. 2013 | Google earth | Area/Unspecified communities | II | III |
| *Triatoma pallidipennis* | Vector | Hemiptera | DTUIII | 19.58500000 | -101.75560000 | Ibañez-Cervantes et al. 2013 | Google earth | Area/Unspecified communities | II | III |
| *Triatoma pallidipennis* | Vector | Hemiptera | DTUIV | 19.58500000 | -101.75560000 | Ibañez-Cervantes et al. 2013 | Google earth | Area/Unspecified communities | II | III |
| *Triatoma pallidipennis* | Vector | Hemiptera | DTUIV | 19.58500000 | -101.75560000 | Ibañez-Cervantes et al. 2013 | Google earth | Area/Unspecified communities | II | III |
| *Triatoma pallidipennis* | Vector | Hemiptera | DTUIV | 19.58500000 | -101.75560000 | Ibañez-Cervantes et al. 2013 | Google earth | Area/Unspecified communities | II | III |
| *Triatoma pallidipennis* | Vector | Hemiptera | DTUIV | 19.58500000 | -101.75560000 | Ibañez-Cervantes et al. 2013 | Google earth | Area/Unspecified communities | II | III |
| *Triatoma pallidipennis* | Vector | Hemiptera | DTUIV | 19.58500000 | -101.75560000 | Ibañez-Cervantes et al. 2013 | Google earth | Area/Unspecified communities | II | III |
| *Triatoma pallidipennis* | Vector | Hemiptera | DTUIV | 19.58500000 | -101.75560000 | Ibañez-Cervantes et al. 2013 | Google earth | Area/Unspecified communities | II | III |
| *Mepraia spinolai* | Vector | Hemiptera | DTUI | -30.31000000 | -71.22000000 | Llewellyn et al. 2009b | Author | NA | I | I |
| *Mepraia spinolai* | Vector | Hemiptera | DTUI | -30.31000000 | -71.22000000 | Llewellyn et al. 2009b | Author | NA | I | I |
| *Micronycteris megalotis* | Host | Chiroptera | DTUI | -4.0925 | -75.1544 | Lima et al. 2015 | Google earth | State | I | I |
| *Molossus molossus* | Host | Chiroptera | DTUI | -5.4025 | -36.95389 | Lima et al. 2015 | Google earth | State | I | I |
| *Molossus molossus* | Host | Chiroptera | DTUI | -5.4025 | -36.95389 | Lima et al. 2015 | Google earth | State | I | I |
| *Monodelphis brevicaudata* | Host | Didelphimorphia | DTUIII | -0.26700000 | -63.20000000 | Marcili et al. 2009b | Google earth | Locality | II | III |
| *Monodelphis domestica* | Host | Didelphimorphia | DTUIII | -24.00000000 | -57.00000000 | Llewellyn et al. 2009a | Author | NA | II | III |
| *Mus musculus* | Host | Rodentia | DTUI | 9.08300000 | -69.08300000 | Carrasco et al. 2012 | Google earth | State | I | I |
| *Myotis keaysi* | Host | Chiroptera | DTUVI | 18.59119900 | -89.39888400 | Lopez-Cancino et al. 2015 | Author | NA | II | ND |
| *Myotis nigricans* | Host | Chiroptera | DTUII | -23.5505 | -46.633 | Lima et al. 2015 | Google earth | State | II | II |
| *Myotis riparius* | Host | Chiroptera | DTUI | -4.0925 | -75.1544 | Lima et al. 2015 | Google earth | State | I | I |
| *Odocoileus virginianus* | Host | Artiodactyla | DTUI | 8.58300000 | -63.95000000 | Carrasco et al. 2012 | Google earth | State | I | I |
| *Ovis aries* | Host | Artiodactyla | DTUVI | 18.59040359 | -89.41808771 | Lopez-Cancino et al. 2015 | Author | NA | II | ND |
| *Ovis aries* | Host | Artiodactyla | DTUVI | 18.59040360 | -89.41808771 | Lopez-Cancino et al. 2015 | Author | NA | II | ND |
| *Ovis aries* | Host | Artiodactyla | DTUVI | 18.59165451 | -89.41611704 | Lopez-Cancino et al. 2015 | Author | NA | II | ND |
| *Panstrongylus chinai* | Vector | Hemiptera | DTUI | -4.25400000 | -79.58000000 | Ocaña-Mayorga et al. 2010 | Author | NA | I | I |
| *Panstrongylus chinai* | Vector | Hemiptera | DTUI | -4.12300000 | -79.60500000 | Ocaña-Mayorga et al. 2010 | Author | NA | I | I |
| *Panstrongylus chinai* | Vector | Hemiptera | DTUI | -4.11600000 | -79.34800000 | Ocaña-Mayorga et al. 2010 | Author | NA | I | I |
| *Panstrongylus chinai* | Vector | Hemiptera | DTUI | -4.04500000 | -79.36100000 | Ocaña-Mayorga et al. 2010 | Author | NA | I | I |
| *Panstrongylus chinai* | Vector | Hemiptera | DTUI | -4.04500000 | -79.36100000 | Ocaña-Mayorga et al. 2010 | Author | NA | I | I |
| *Panstrongylus chinai* | Vector | Hemiptera | DTUI | -4.12500000 | -79.55800000 | Ocaña-Mayorga et al. 2010 | Author | NA | I | I |
| *Panstrongylus chinai* | Vector | Hemiptera | DTUI | -4.08300000 | -79.82900000 | Ocaña-Mayorga et al. 2010 | Author | NA | I | I |
| *Panstrongylus geniculatus* | Vector | Hemiptera | DTUI | 8.58300000 | -63.95000000 | Carrasco et al. 2012 | Google earth | State | I | I |
| *Panstrongylus geniculatus* | Vector | Hemiptera | DTUI | 8.58300000 | -63.95000000 | Carrasco et al. 2012 | Google earth | State | I | I |
| *Panstrongylus geniculatus* | Vector | Hemiptera | DTUI | 10.21670000 | -67.28340000 | Carrasco et al. 2012 | Google earth | State | I | I |
| *Panstrongylus geniculatus* | Vector | Hemiptera | DTUI | 10.21670000 | -67.28340000 | Carrasco et al. 2012 | Google earth | State | I | I |
| *Panstrongylus geniculatus* | Vector | Hemiptera | DTUI | 10.21670000 | -67.28340000 | Carrasco et al. 2012 | Google earth | State | I | I |
| *Panstrongylus geniculatus* | Vector | Hemiptera | DTUI | 10.21670000 | -67.28340000 | Carrasco et al. 2012 | Google earth | State | I | I |
| *Panstrongylus geniculatus* | Vector | Hemiptera | DTUI | 10.11670000 | -68.03400000 | Carrasco et al. 2012 | Google earth | State | I | I |
| *Panstrongylus geniculatus* | Vector | Hemiptera | DTUI | 9.36700000 | -68.03400000 | Carrasco et al. 2012 | Google earth | State | I | I |
| *Panstrongylus geniculatus* | Vector | Hemiptera | DTUI | 9.36700000 | -68.03400000 | Carrasco et al. 2012 | Google earth | State | I | I |
| *Panstrongylus geniculatus* | Vector | Hemiptera | DTUI | 10.05000000 | -66.95000000 | Carrasco et al. 2012 | Google earth | State | I | I |
| *Panstrongylus geniculatus* | Vector | Hemiptera | DTUI | 10.05000000 | -66.95000000 | Carrasco et al. 2012 | Google earth | State | I | I |
| *Panstrongylus geniculatus* | Vector | Hemiptera | DTUI | 10.05000000 | -66.95000000 | Carrasco et al. 2012 | Google earth | State | I | I |
| *Panstrongylus geniculatus* | Vector | Hemiptera | DTUI | 10.05000000 | -66.95000000 | Carrasco et al. 2012 | Google earth | State | I | I |
| *Panstrongylus geniculatus* | Vector | Hemiptera | DTUI | 10.05000000 | -66.95000000 | Carrasco et al. 2012 | Google earth | State | I | I |
| *Panstrongylus geniculatus* | Vector | Hemiptera | DTUI | 10.05000000 | -66.95000000 | Carrasco et al. 2012 | Google earth | State | I | I |
| *Panstrongylus geniculatus* | Vector | Hemiptera | DTUI | 10.05000000 | -66.95000000 | Carrasco et al. 2012 | Google earth | State | I | I |
| *Panstrongylus geniculatus* | Vector | Hemiptera | DTUI | 10.05000000 | -66.95000000 | Carrasco et al. 2012 | Google earth | State | I | I |
| *Panstrongylus geniculatus* | Vector | Hemiptera | DTUI | 10.05000000 | -66.95000000 | Carrasco et al. 2012 | Google earth | State | I | I |
| *Panstrongylus geniculatus* | Vector | Hemiptera | DTUI | 10.05000000 | -66.95000000 | Carrasco et al. 2012 | Google earth | State | I | I |
| *Panstrongylus geniculatus* | Vector | Hemiptera | DTUI | 10.05000000 | -66.95000000 | Carrasco et al. 2012 | Google earth | State | I | I |
| *Panstrongylus geniculatus* | Vector | Hemiptera | DTUI | 10.05000000 | -66.95000000 | Carrasco et al. 2012 | Google earth | State | I | I |
| *Panstrongylus geniculatus* | Vector | Hemiptera | DTUI | 10.05000000 | -66.95000000 | Carrasco et al. 2012 | Google earth | State | I | I |
| *Panstrongylus geniculatus* | Vector | Hemiptera | DTUI | 10.05000000 | -66.95000000 | Carrasco et al. 2012 | Google earth | State | I | I |
| *Panstrongylus geniculatus* | Vector | Hemiptera | DTUI | 10.05000000 | -66.95000000 | Carrasco et al. 2012 | Google earth | State | I | I |
| *Panstrongylus geniculatus* | Vector | Hemiptera | DTUI | 10.05000000 | -66.95000000 | Carrasco et al. 2012 | Google earth | State | I | I |
| *Panstrongylus geniculatus* | Vector | Hemiptera | DTUI | 10.05000000 | -66.95000000 | Carrasco et al. 2012 | Google earth | State | I | I |
| *Panstrongylus geniculatus* | Vector | Hemiptera | DTUI | 10.05000000 | -66.95000000 | Carrasco et al. 2012 | Google earth | State | I | I |
| *Panstrongylus geniculatus* | Vector | Hemiptera | DTUI | 10.05000000 | -66.95000000 | Carrasco et al. 2012 | Google earth | State | I | I |
| *Panstrongylus geniculatus* | Vector | Hemiptera | DTUI | 10.05000000 | -66.95000000 | Carrasco et al. 2012 | Google earth | State | I | I |
| *Panstrongylus geniculatus* | Vector | Hemiptera | DTUI | 10.05000000 | -66.95000000 | Carrasco et al. 2012 | Google earth | State | I | I |
| *Panstrongylus geniculatus* | Vector | Hemiptera | DTUI | 10.05000000 | -66.95000000 | Carrasco et al. 2012 | Google earth | State | I | I |
| *Panstrongylus geniculatus* | Vector | Hemiptera | DTUI | 10.05000000 | -66.95000000 | Carrasco et al. 2012 | Google earth | State | I | I |
| *Panstrongylus geniculatus* | Vector | Hemiptera | DTUI | 10.05000000 | -66.95000000 | Carrasco et al. 2012 | Google earth | State | I | I |
| *Panstrongylus geniculatus* | Vector | Hemiptera | DTUI | 10.05000000 | -66.95000000 | Carrasco et al. 2012 | Google earth | State | I | I |
| *Panstrongylus geniculatus* | Vector | Hemiptera | DTUI | 10.05000000 | -66.95000000 | Carrasco et al. 2012 | Google earth | State | I | I |
| *Panstrongylus geniculatus* | Vector | Hemiptera | DTUI | 10.05000000 | -66.95000000 | Carrasco et al. 2012 | Google earth | State | I | I |
| *Panstrongylus geniculatus* | Vector | Hemiptera | DTUI | 10.05000000 | -66.95000000 | Carrasco et al. 2012 | Google earth | State | I | I |
| *Panstrongylus geniculatus* | Vector | Hemiptera | DTUI | 10.05000000 | -66.95000000 | Carrasco et al. 2012 | Google earth | State | I | I |
| *Panstrongylus geniculatus* | Vector | Hemiptera | DTUI | 10.05000000 | -66.95000000 | Carrasco et al. 2012 | Google earth | State | I | I |
| *Panstrongylus geniculatus* | Vector | Hemiptera | DTUI | 10.05000000 | -66.95000000 | Carrasco et al. 2012 | Google earth | State | I | I |
| *Panstrongylus geniculatus* | Vector | Hemiptera | DTUI | 10.05000000 | -66.95000000 | Carrasco et al. 2012 | Google earth | State | I | I |
| *Panstrongylus geniculatus* | Vector | Hemiptera | DTUI | 10.05000000 | -66.95000000 | Carrasco et al. 2012 | Google earth | State | I | I |
| *Panstrongylus geniculatus* | Vector | Hemiptera | DTUI | 10.05000000 | -66.95000000 | Carrasco et al. 2012 | Google earth | State | I | I |
| *Panstrongylus geniculatus* | Vector | Hemiptera | DTUI | 10.05000000 | -66.95000000 | Carrasco et al. 2012 | Google earth | State | I | I |
| *Panstrongylus geniculatus* | Vector | Hemiptera | DTUI | 10.05000000 | -66.95000000 | Carrasco et al. 2012 | Google earth | State | I | I |
| *Panstrongylus geniculatus* | Vector | Hemiptera | DTUI | 10.05000000 | -66.95000000 | Carrasco et al. 2012 | Google earth | State | I | I |
| *Panstrongylus geniculatus* | Vector | Hemiptera | DTUI | 10.05000000 | -66.95000000 | Carrasco et al. 2012 | Google earth | State | I | I |
| *Panstrongylus geniculatus* | Vector | Hemiptera | DTUI | 10.05000000 | -66.95000000 | Carrasco et al. 2012 | Google earth | State | I | I |
| *Panstrongylus geniculatus* | Vector | Hemiptera | DTUI | 10.05000000 | -66.95000000 | Carrasco et al. 2012 | Google earth | State | I | I |
| *Panstrongylus geniculatus* | Vector | Hemiptera | DTUI | 10.05000000 | -66.95000000 | Carrasco et al. 2012 | Google earth | State | I | I |
| *Panstrongylus geniculatus* | Vector | Hemiptera | DTUI | 10.05000000 | -66.95000000 | Carrasco et al. 2012 | Google earth | State | I | I |
| *Panstrongylus geniculatus* | Vector | Hemiptera | DTUI | 10.05000000 | -66.95000000 | Carrasco et al. 2012 | Google earth | State | I | I |
| *Panstrongylus geniculatus* | Vector | Hemiptera | DTUI | 10.05000000 | -66.95000000 | Carrasco et al. 2012 | Google earth | State | I | I |
| *Panstrongylus geniculatus* | Vector | Hemiptera | DTUI | 10.05000000 | -66.95000000 | Carrasco et al. 2012 | Google earth | State | I | I |
| *Panstrongylus geniculatus* | Vector | Hemiptera | DTUI | 10.05000000 | -66.95000000 | Carrasco et al. 2012 | Google earth | State | I | I |
| *Panstrongylus geniculatus* | Vector | Hemiptera | DTUI | 10.05000000 | -66.95000000 | Carrasco et al. 2012 | Google earth | State | I | I |
| *Panstrongylus geniculatus* | Vector | Hemiptera | DTUI | 10.05000000 | -66.95000000 | Carrasco et al. 2012 | Google earth | State | I | I |
| *Panstrongylus geniculatus* | Vector | Hemiptera | DTUI | 10.05000000 | -66.95000000 | Carrasco et al. 2012 | Google earth | State | I | I |
| *Panstrongylus geniculatus* | Vector | Hemiptera | DTUI | 10.05000000 | -66.95000000 | Carrasco et al. 2012 | Google earth | State | I | I |
| *Panstrongylus geniculatus* | Vector | Hemiptera | DTUI | 10.05000000 | -66.95000000 | Carrasco et al. 2012 | Google earth | State | I | I |
| *Panstrongylus geniculatus* | Vector | Hemiptera | DTUI | 10.05000000 | -66.95000000 | Carrasco et al. 2012 | Google earth | State | I | I |
| *Panstrongylus geniculatus* | Vector | Hemiptera | DTUI | 10.05000000 | -66.95000000 | Carrasco et al. 2012 | Google earth | State | I | I |
| *Panstrongylus geniculatus* | Vector | Hemiptera | DTUI | 10.05000000 | -66.95000000 | Carrasco et al. 2012 | Google earth | State | I | I |
| *Panstrongylus geniculatus* | Vector | Hemiptera | DTUI | 10.05000000 | -66.95000000 | Carrasco et al. 2012 | Google earth | State | I | I |
| *Panstrongylus geniculatus* | Vector | Hemiptera | DTUI | 10.05000000 | -66.95000000 | Carrasco et al. 2012 | Google earth | State | I | I |
| *Panstrongylus geniculatus* | Vector | Hemiptera | DTUI | 10.05000000 | -66.95000000 | Carrasco et al. 2012 | Google earth | State | I | I |
| *Panstrongylus geniculatus* | Vector | Hemiptera | DTUI | 10.05000000 | -66.95000000 | Carrasco et al. 2012 | Google earth | State | I | I |
| *Panstrongylus geniculatus* | Vector | Hemiptera | DTUI | 10.05000000 | -66.95000000 | Carrasco et al. 2012 | Google earth | State | I | I |
| *Panstrongylus geniculatus* | Vector | Hemiptera | DTUI | 10.05000000 | -66.95000000 | Carrasco et al. 2012 | Google earth | State | I | I |
| *Panstrongylus geniculatus* | Vector | Hemiptera | DTUI | 10.05000000 | -66.95000000 | Carrasco et al. 2012 | Google earth | State | I | I |
| *Panstrongylus geniculatus* | Vector | Hemiptera | DTUI | 10.05000000 | -66.95000000 | Carrasco et al. 2012 | Google earth | State | I | I |
| *Panstrongylus geniculatus* | Vector | Hemiptera | DTUI | 10.05000000 | -66.95000000 | Carrasco et al. 2012 | Google earth | State | I | I |
| *Panstrongylus geniculatus* | Vector | Hemiptera | DTUI | 10.05000000 | -66.95000000 | Carrasco et al. 2012 | Google earth | State | I | I |
| *Panstrongylus geniculatus* | Vector | Hemiptera | DTUI | 10.05000000 | -66.95000000 | Carrasco et al. 2012 | Google earth | State | I | I |
| *Panstrongylus geniculatus* | Vector | Hemiptera | DTUI | 10.05000000 | -66.95000000 | Carrasco et al. 2012 | Google earth | State | I | I |
| *Panstrongylus geniculatus* | Vector | Hemiptera | DTUI | 10.05000000 | -66.95000000 | Carrasco et al. 2012 | Google earth | State | I | I |
| *Panstrongylus geniculatus* | Vector | Hemiptera | DTUI | 10.05000000 | -66.95000000 | Carrasco et al. 2012 | Google earth | State | I | I |
| *Panstrongylus geniculatus* | Vector | Hemiptera | DTUI | 10.05000000 | -66.95000000 | Carrasco et al. 2012 | Google earth | State | I | I |
| *Panstrongylus geniculatus* | Vector | Hemiptera | DTUI | 10.05000000 | -66.95000000 | Carrasco et al. 2012 | Google earth | State | I | I |
| *Panstrongylus geniculatus* | Vector | Hemiptera | DTUI | 10.05000000 | -66.95000000 | Carrasco et al. 2012 | Google earth | State | I | I |
| *Panstrongylus geniculatus* | Vector | Hemiptera | DTUI | 10.05000000 | -66.95000000 | Carrasco et al. 2012 | Google earth | State | I | I |
| *Panstrongylus geniculatus* | Vector | Hemiptera | DTUI | 10.05000000 | -66.95000000 | Carrasco et al. 2012 | Google earth | State | I | I |
| *Panstrongylus geniculatus* | Vector | Hemiptera | DTUI | 10.05000000 | -66.95000000 | Carrasco et al. 2012 | Google earth | State | I | I |
| *Panstrongylus geniculatus* | Vector | Hemiptera | DTUI | 10.05000000 | -66.95000000 | Carrasco et al. 2012 | Google earth | State | I | I |
| *Panstrongylus geniculatus* | Vector | Hemiptera | DTUI | 10.05000000 | -66.95000000 | Carrasco et al. 2012 | Google earth | State | I | I |
| *Panstrongylus geniculatus* | Vector | Hemiptera | DTUI | 10.05000000 | -66.95000000 | Carrasco et al. 2012 | Google earth | State | I | I |
| *Panstrongylus geniculatus* | Vector | Hemiptera | DTUI | 10.05000000 | -66.95000000 | Carrasco et al. 2012 | Google earth | State | I | I |
| *Panstrongylus geniculatus* | Vector | Hemiptera | DTUI | 10.05000000 | -66.95000000 | Carrasco et al. 2012 | Google earth | State | I | I |
| *Panstrongylus geniculatus* | Vector | Hemiptera | DTUI | 10.05000000 | -66.95000000 | Carrasco et al. 2012 | Google earth | State | I | I |
| *Panstrongylus geniculatus* | Vector | Hemiptera | DTUI | 10.05000000 | -66.95000000 | Carrasco et al. 2012 | Google earth | State | I | I |
| *Panstrongylus geniculatus* | Vector | Hemiptera | DTUI | 10.05000000 | -66.95000000 | Carrasco et al. 2012 | Google earth | State | I | I |
| *Panstrongylus geniculatus* | Vector | Hemiptera | DTUI | 10.05000000 | -66.95000000 | Carrasco et al. 2012 | Google earth | State | I | I |
| *Panstrongylus geniculatus* | Vector | Hemiptera | DTUI | 10.05000000 | -66.95000000 | Carrasco et al. 2012 | Google earth | State | I | I |
| *Panstrongylus geniculatus* | Vector | Hemiptera | DTUI | 10.05000000 | -66.95000000 | Carrasco et al. 2012 | Google earth | State | I | I |
| *Panstrongylus geniculatus* | Vector | Hemiptera | DTUI | 10.05000000 | -66.95000000 | Carrasco et al. 2012 | Google earth | State | I | I |
| *Panstrongylus geniculatus* | Vector | Hemiptera | DTUI | 10.05000000 | -66.95000000 | Carrasco et al. 2012 | Google earth | State | I | I |
| *Panstrongylus geniculatus* | Vector | Hemiptera | DTUI | 10.05000000 | -66.95000000 | Carrasco et al. 2012 | Google earth | State | I | I |
| *Panstrongylus geniculatus* | Vector | Hemiptera | DTUI | 10.05000000 | -66.95000000 | Carrasco et al. 2012 | Google earth | State | I | I |
| *Panstrongylus geniculatus* | Vector | Hemiptera | DTUI | 10.05000000 | -66.95000000 | Carrasco et al. 2012 | Google earth | State | I | I |
| *Panstrongylus geniculatus* | Vector | Hemiptera | DTUI | 10.05000000 | -66.95000000 | Carrasco et al. 2012 | Google earth | State | I | I |
| *Panstrongylus geniculatus* | Vector | Hemiptera | DTUI | 10.05000000 | -66.95000000 | Carrasco et al. 2012 | Google earth | State | I | I |
| *Panstrongylus geniculatus* | Vector | Hemiptera | DTUI | 10.05000000 | -66.95000000 | Carrasco et al. 2012 | Google earth | State | I | I |
| *Panstrongylus geniculatus* | Vector | Hemiptera | DTUI | 10.05000000 | -66.95000000 | Carrasco et al. 2012 | Google earth | State | I | I |
| *Panstrongylus geniculatus* | Vector | Hemiptera | DTUI | 10.05000000 | -66.95000000 | Carrasco et al. 2012 | Google earth | State | I | I |
| *Panstrongylus geniculatus* | Vector | Hemiptera | DTUI | 10.05000000 | -66.95000000 | Carrasco et al. 2012 | Google earth | State | I | I |
| *Panstrongylus geniculatus* | Vector | Hemiptera | DTUI | 10.05000000 | -66.95000000 | Carrasco et al. 2012 | Google earth | State | I | I |
| *Panstrongylus geniculatus* | Vector | Hemiptera | DTUI | 10.05000000 | -66.95000000 | Carrasco et al. 2012 | Google earth | State | I | I |
| *Panstrongylus geniculatus* | Vector | Hemiptera | DTUI | 10.05000000 | -66.95000000 | Carrasco et al. 2012 | Google earth | State | I | I |
| *Panstrongylus geniculatus* | Vector | Hemiptera | DTUI | 10.05000000 | -66.95000000 | Carrasco et al. 2012 | Google earth | State | I | I |
| *Panstrongylus geniculatus* | Vector | Hemiptera | DTUI | 10.05000000 | -66.95000000 | Carrasco et al. 2012 | Google earth | State | I | I |
| *Panstrongylus geniculatus* | Vector | Hemiptera | DTUI | 10.05000000 | -66.95000000 | Carrasco et al. 2012 | Google earth | State | I | I |
| *Panstrongylus geniculatus* | Vector | Hemiptera | DTUI | 10.05000000 | -66.95000000 | Carrasco et al. 2012 | Google earth | State | I | I |
| *Panstrongylus geniculatus* | Vector | Hemiptera | DTUI | 10.05000000 | -66.95000000 | Carrasco et al. 2012 | Google earth | State | I | I |
| *Panstrongylus geniculatus* | Vector | Hemiptera | DTUI | 10.05000000 | -66.95000000 | Carrasco et al. 2012 | Google earth | State | I | I |
| *Panstrongylus geniculatus* | Vector | Hemiptera | DTUI | 10.05000000 | -66.95000000 | Carrasco et al. 2012 | Google earth | State | I | I |
| *Panstrongylus geniculatus* | Vector | Hemiptera | DTUI | 10.05000000 | -66.95000000 | Carrasco et al. 2012 | Google earth | State | I | I |
| *Panstrongylus geniculatus* | Vector | Hemiptera | DTUI | 10.05000000 | -66.95000000 | Carrasco et al. 2012 | Google earth | State | I | I |
| *Panstrongylus geniculatus* | Vector | Hemiptera | DTUI | 10.05000000 | -66.95000000 | Carrasco et al. 2012 | Google earth | State | I | I |
| *Panstrongylus geniculatus* | Vector | Hemiptera | DTUI | 10.05000000 | -66.95000000 | Carrasco et al. 2012 | Google earth | State | I | I |
| *Panstrongylus geniculatus* | Vector | Hemiptera | DTUI | 10.05000000 | -66.95000000 | Carrasco et al. 2012 | Google earth | State | I | I |
| *Panstrongylus geniculatus* | Vector | Hemiptera | DTUI | 10.05000000 | -66.95000000 | Carrasco et al. 2012 | Google earth | State | I | I |
| *Panstrongylus geniculatus* | Vector | Hemiptera | DTUI | 10.05000000 | -66.95000000 | Carrasco et al. 2012 | Google earth | State | I | I |
| *Panstrongylus geniculatus* | Vector | Hemiptera | DTUI | 10.05000000 | -66.95000000 | Carrasco et al. 2012 | Google earth | State | I | I |
| *Panstrongylus geniculatus* | Vector | Hemiptera | DTUI | 10.05000000 | -66.95000000 | Carrasco et al. 2012 | Google earth | State | I | I |
| *Panstrongylus geniculatus* | Vector | Hemiptera | DTUI | 10.05000000 | -66.95000000 | Carrasco et al. 2012 | Google earth | State | I | I |
| *Panstrongylus geniculatus* | Vector | Hemiptera | DTUI | 10.05000000 | -66.95000000 | Carrasco et al. 2012 | Google earth | State | I | I |
| *Panstrongylus geniculatus* | Vector | Hemiptera | DTUI | 10.05000000 | -66.95000000 | Carrasco et al. 2012 | Google earth | State | I | I |
| *Panstrongylus geniculatus* | Vector | Hemiptera | DTUI | 10.05000000 | -66.95000000 | Carrasco et al. 2012 | Google earth | State | I | I |
| *Panstrongylus geniculatus* | Vector | Hemiptera | DTUI | 10.05000000 | -66.95000000 | Carrasco et al. 2012 | Google earth | State | I | I |
| *Panstrongylus geniculatus* | Vector | Hemiptera | DTUI | 10.05000000 | -66.95000000 | Carrasco et al. 2012 | Google earth | State | I | I |
| *Panstrongylus geniculatus* | Vector | Hemiptera | DTUI | 10.05000000 | -66.95000000 | Carrasco et al. 2012 | Google earth | State | I | I |
| *Panstrongylus geniculatus* | Vector | Hemiptera | DTUI | 10.05000000 | -66.95000000 | Carrasco et al. 2012 | Google earth | State | I | I |
| *Panstrongylus geniculatus* | Vector | Hemiptera | DTUI | 10.05000000 | -66.95000000 | Carrasco et al. 2012 | Google earth | State | I | I |
| *Panstrongylus geniculatus* | Vector | Hemiptera | DTUI | 10.05000000 | -66.95000000 | Carrasco et al. 2012 | Google earth | State | I | I |
| *Panstrongylus geniculatus* | Vector | Hemiptera | DTUI | 10.05000000 | -66.95000000 | Carrasco et al. 2012 | Google earth | State | I | I |
| *Panstrongylus geniculatus* | Vector | Hemiptera | DTUI | 10.05000000 | -66.95000000 | Carrasco et al. 2012 | Google earth | State | I | I |
| *Panstrongylus geniculatus* | Vector | Hemiptera | DTUI | 10.05000000 | -66.95000000 | Carrasco et al. 2012 | Google earth | State | I | I |
| *Panstrongylus geniculatus* | Vector | Hemiptera | DTUI | 10.05000000 | -66.95000000 | Carrasco et al. 2012 | Google earth | State | I | I |
| *Panstrongylus geniculatus* | Vector | Hemiptera | DTUI | 10.05000000 | -66.95000000 | Carrasco et al. 2012 | Google earth | State | I | I |
| *Panstrongylus geniculatus* | Vector | Hemiptera | DTUI | 10.05000000 | -66.95000000 | Carrasco et al. 2012 | Google earth | State | I | I |
| *Panstrongylus geniculatus* | Vector | Hemiptera | DTUI | 10.05000000 | -66.95000000 | Carrasco et al. 2012 | Google earth | State | I | I |
| *Panstrongylus geniculatus* | Vector | Hemiptera | DTUI | 10.05000000 | -66.95000000 | Carrasco et al. 2012 | Google earth | State | I | I |
| *Panstrongylus geniculatus* | Vector | Hemiptera | DTUI | 10.05000000 | -66.95000000 | Carrasco et al. 2012 | Google earth | State | I | I |
| *Panstrongylus geniculatus* | Vector | Hemiptera | DTUI | 10.05000000 | -66.95000000 | Carrasco et al. 2012 | Google earth | State | I | I |
| *Panstrongylus geniculatus* | Vector | Hemiptera | DTUI | 10.05000000 | -66.95000000 | Carrasco et al. 2012 | Google earth | State | I | I |
| *Panstrongylus geniculatus* | Vector | Hemiptera | DTUI | 10.05000000 | -66.95000000 | Carrasco et al. 2012 | Google earth | State | I | I |
| *Panstrongylus geniculatus* | Vector | Hemiptera | DTUI | 10.05000000 | -66.95000000 | Carrasco et al. 2012 | Google earth | State | I | I |
| *Panstrongylus geniculatus* | Vector | Hemiptera | DTUI | 10.05000000 | -66.95000000 | Carrasco et al. 2012 | Google earth | State | I | I |
| *Panstrongylus geniculatus* | Vector | Hemiptera | DTUI | 10.05000000 | -66.95000000 | Carrasco et al. 2012 | Google earth | State | I | I |
| *Panstrongylus geniculatus* | Vector | Hemiptera | DTUI | 10.05000000 | -66.95000000 | Carrasco et al. 2012 | Google earth | State | I | I |
| *Panstrongylus geniculatus* | Vector | Hemiptera | DTUI | 10.05000000 | -66.95000000 | Carrasco et al. 2012 | Google earth | State | I | I |
| *Panstrongylus geniculatus* | Vector | Hemiptera | DTUI | 10.05000000 | -66.95000000 | Carrasco et al. 2012 | Google earth | State | I | I |
| *Panstrongylus geniculatus* | Vector | Hemiptera | DTUI | 10.05000000 | -66.95000000 | Carrasco et al. 2012 | Google earth | State | I | I |
| *Panstrongylus geniculatus* | Vector | Hemiptera | DTUI | 10.05000000 | -66.95000000 | Carrasco et al. 2012 | Google earth | State | I | I |
| *Panstrongylus geniculatus* | Vector | Hemiptera | DTUI | 10.05000000 | -66.95000000 | Carrasco et al. 2012 | Google earth | State | I | I |
| *Panstrongylus geniculatus* | Vector | Hemiptera | DTUI | 10.05000000 | -66.95000000 | Carrasco et al. 2012 | Google earth | State | I | I |
| *Panstrongylus geniculatus* | Vector | Hemiptera | DTUI | 10.05000000 | -66.95000000 | Carrasco et al. 2012 | Google earth | State | I | I |
| *Panstrongylus geniculatus* | Vector | Hemiptera | DTUI | 10.05000000 | -66.95000000 | Carrasco et al. 2012 | Google earth | State | I | I |
| *Panstrongylus geniculatus* | Vector | Hemiptera | DTUI | 10.05000000 | -66.95000000 | Carrasco et al. 2012 | Google earth | State | I | I |
| *Panstrongylus geniculatus* | Vector | Hemiptera | DTUI | 10.05000000 | -66.95000000 | Carrasco et al. 2012 | Google earth | State | I | I |
| *Panstrongylus geniculatus* | Vector | Hemiptera | DTUI | 10.05000000 | -66.95000000 | Carrasco et al. 2012 | Google earth | State | I | I |
| *Panstrongylus geniculatus* | Vector | Hemiptera | DTUI | 10.05000000 | -66.95000000 | Carrasco et al. 2012 | Google earth | State | I | I |
| *Panstrongylus geniculatus* | Vector | Hemiptera | DTUI | 10.05000000 | -66.95000000 | Carrasco et al. 2012 | Google earth | State | I | I |
| *Panstrongylus geniculatus* | Vector | Hemiptera | DTUI | 10.05000000 | -66.95000000 | Carrasco et al. 2012 | Google earth | State | I | I |
| *Panstrongylus geniculatus* | Vector | Hemiptera | DTUI | 10.05000000 | -66.95000000 | Carrasco et al. 2012 | Google earth | State | I | I |
| *Panstrongylus geniculatus* | Vector | Hemiptera | DTUI | 10.05000000 | -66.95000000 | Carrasco et al. 2012 | Google earth | State | I | I |
| *Panstrongylus geniculatus* | Vector | Hemiptera | DTUI | 10.05000000 | -66.95000000 | Carrasco et al. 2012 | Google earth | State | I | I |
| *Panstrongylus geniculatus* | Vector | Hemiptera | DTUI | 10.05000000 | -66.95000000 | Carrasco et al. 2012 | Google earth | State | I | I |
| *Panstrongylus geniculatus* | Vector | Hemiptera | DTUI | 10.05000000 | -66.95000000 | Carrasco et al. 2012 | Google earth | State | I | I |
| *Panstrongylus geniculatus* | Vector | Hemiptera | DTUI | 10.05000000 | -66.95000000 | Carrasco et al. 2012 | Google earth | State | I | I |
| *Panstrongylus geniculatus* | Vector | Hemiptera | DTUI | 10.05000000 | -66.95000000 | Carrasco et al. 2012 | Google earth | State | I | I |
| *Panstrongylus geniculatus* | Vector | Hemiptera | DTUI | 10.05000000 | -66.95000000 | Carrasco et al. 2012 | Google earth | State | I | I |
| *Panstrongylus geniculatus* | Vector | Hemiptera | DTUI | 10.25000000 | -66.41670000 | Carrasco et al. 2012 | Google earth | State | I | I |
| *Panstrongylus geniculatus* | Vector | Hemiptera | DTUI | 10.25000000 | -66.41670000 | Carrasco et al. 2012 | Google earth | State | I | I |
| *Panstrongylus geniculatus* | Vector | Hemiptera | DTUI | 10.25000000 | -66.41670000 | Carrasco et al. 2012 | Google earth | State | I | I |
| *Panstrongylus geniculatus* | Vector | Hemiptera | DTUI | 10.25000000 | -66.41670000 | Carrasco et al. 2012 | Google earth | State | I | I |
| *Panstrongylus geniculatus* | Vector | Hemiptera | DTUI | 10.25000000 | -66.41670000 | Carrasco et al. 2012 | Google earth | State | I | I |
| *Panstrongylus geniculatus* | Vector | Hemiptera | DTUI | 10.25000000 | -66.41670000 | Carrasco et al. 2012 | Google earth | State | I | I |
| *Panstrongylus geniculatus* | Vector | Hemiptera | DTUI | 10.25000000 | -66.41670000 | Carrasco et al. 2012 | Google earth | State | I | I |
| *Panstrongylus geniculatus* | Vector | Hemiptera | DTUI | 10.25000000 | -66.41670000 | Carrasco et al. 2012 | Google earth | State | I | I |
| *Panstrongylus geniculatus* | Vector | Hemiptera | DTUI | 10.25000000 | -66.41670000 | Carrasco et al. 2012 | Google earth | State | I | I |
| *Panstrongylus geniculatus* | Vector | Hemiptera | DTUI | 10.25000000 | -66.41670000 | Carrasco et al. 2012 | Google earth | State | I | I |
| *Panstrongylus geniculatus* | Vector | Hemiptera | DTUI | 10.25000000 | -66.41670000 | Carrasco et al. 2012 | Google earth | State | I | I |
| *Panstrongylus geniculatus* | Vector | Hemiptera | DTUI | 10.25000000 | -66.41670000 | Carrasco et al. 2012 | Google earth | State | I | I |
| *Panstrongylus geniculatus* | Vector | Hemiptera | DTUI | 10.25000000 | -66.41670000 | Carrasco et al. 2012 | Google earth | State | I | I |
| *Panstrongylus geniculatus* | Vector | Hemiptera | DTUI | 10.25000000 | -66.41670000 | Carrasco et al. 2012 | Google earth | State | I | I |
| *Panstrongylus geniculatus* | Vector | Hemiptera | DTUI | 10.25000000 | -66.41670000 | Carrasco et al. 2012 | Google earth | State | I | I |
| *Panstrongylus geniculatus* | Vector | Hemiptera | DTUI | 10.25000000 | -66.41670000 | Carrasco et al. 2012 | Google earth | State | I | I |
| *Panstrongylus geniculatus* | Vector | Hemiptera | DTUI | 10.25000000 | -66.41670000 | Carrasco et al. 2012 | Google earth | State | I | I |
| *Panstrongylus geniculatus* | Vector | Hemiptera | DTUI | 10.25000000 | -66.41670000 | Carrasco et al. 2012 | Google earth | State | I | I |
| *Panstrongylus geniculatus* | Vector | Hemiptera | DTUI | 10.25000000 | -66.41670000 | Carrasco et al. 2012 | Google earth | State | I | I |
| *Panstrongylus geniculatus* | Vector | Hemiptera | DTUI | 10.25000000 | -66.41670000 | Carrasco et al. 2012 | Google earth | State | I | I |
| *Panstrongylus geniculatus* | Vector | Hemiptera | DTUI | 10.25000000 | -66.41670000 | Carrasco et al. 2012 | Google earth | State | I | I |
| *Panstrongylus geniculatus* | Vector | Hemiptera | DTUI | 10.25000000 | -66.41670000 | Carrasco et al. 2012 | Google earth | State | I | I |
| *Panstrongylus geniculatus* | Vector | Hemiptera | DTUI | 10.25000000 | -66.41670000 | Carrasco et al. 2012 | Google earth | State | I | I |
| *Panstrongylus geniculatus* | Vector | Hemiptera | DTUI | 10.25000000 | -66.41670000 | Carrasco et al. 2012 | Google earth | State | I | I |
| *Panstrongylus geniculatus* | Vector | Hemiptera | DTUI | 10.25000000 | -66.41670000 | Carrasco et al. 2012 | Google earth | State | I | I |
| *Panstrongylus geniculatus* | Vector | Hemiptera | DTUI | 10.25000000 | -66.41670000 | Carrasco et al. 2012 | Google earth | State | I | I |
| *Panstrongylus geniculatus* | Vector | Hemiptera | DTUI | 10.25000000 | -66.41670000 | Carrasco et al. 2012 | Google earth | State | I | I |
| *Panstrongylus geniculatus* | Vector | Hemiptera | DTUI | 10.25000000 | -66.41670000 | Carrasco et al. 2012 | Google earth | State | I | I |
| *Panstrongylus geniculatus* | Vector | Hemiptera | DTUI | 10.25000000 | -66.41670000 | Carrasco et al. 2012 | Google earth | State | I | I |
| *Panstrongylus geniculatus* | Vector | Hemiptera | DTUI | 10.25000000 | -66.41670000 | Carrasco et al. 2012 | Google earth | State | I | I |
| *Panstrongylus geniculatus* | Vector | Hemiptera | DTUI | 10.25000000 | -66.41670000 | Carrasco et al. 2012 | Google earth | State | I | I |
| *Panstrongylus geniculatus* | Vector | Hemiptera | DTUI | 10.25000000 | -66.41670000 | Carrasco et al. 2012 | Google earth | State | I | I |
| *Panstrongylus geniculatus* | Vector | Hemiptera | DTUI | 10.25000000 | -66.41670000 | Carrasco et al. 2012 | Google earth | State | I | I |
| *Panstrongylus geniculatus* | Vector | Hemiptera | DTUI | 10.25000000 | -66.41670000 | Carrasco et al. 2012 | Google earth | State | I | I |
| *Panstrongylus geniculatus* | Vector | Hemiptera | DTUI | 10.25000000 | -66.41670000 | Carrasco et al. 2012 | Google earth | State | I | I |
| *Panstrongylus geniculatus* | Vector | Hemiptera | DTUI | 10.25000000 | -66.41670000 | Carrasco et al. 2012 | Google earth | State | I | I |
| *Panstrongylus geniculatus* | Vector | Hemiptera | DTUI | 10.25000000 | -66.41670000 | Carrasco et al. 2012 | Google earth | State | I | I |
| *Panstrongylus geniculatus* | Vector | Hemiptera | DTUI | 10.25000000 | -66.41670000 | Carrasco et al. 2012 | Google earth | State | I | I |
| *Panstrongylus geniculatus* | Vector | Hemiptera | DTUI | 10.25000000 | -66.41670000 | Carrasco et al. 2012 | Google earth | State | I | I |
| *Panstrongylus geniculatus* | Vector | Hemiptera | DTUI | 10.25000000 | -66.41670000 | Carrasco et al. 2012 | Google earth | State | I | I |
| *Panstrongylus geniculatus* | Vector | Hemiptera | DTUI | 10.25000000 | -66.41670000 | Carrasco et al. 2012 | Google earth | State | I | I |
| *Panstrongylus geniculatus* | Vector | Hemiptera | DTUI | 10.25000000 | -66.41670000 | Carrasco et al. 2012 | Google earth | State | I | I |
| *Panstrongylus geniculatus* | Vector | Hemiptera | DTUI | 10.25000000 | -66.41670000 | Carrasco et al. 2012 | Google earth | State | I | I |
| *Panstrongylus geniculatus* | Vector | Hemiptera | DTUI | 10.25000000 | -66.41670000 | Carrasco et al. 2012 | Google earth | State | I | I |
| *Panstrongylus geniculatus* | Vector | Hemiptera | DTUI | 10.25000000 | -66.41670000 | Carrasco et al. 2012 | Google earth | State | I | I |
| *Panstrongylus geniculatus* | Vector | Hemiptera | DTUI | 10.25000000 | -66.41670000 | Carrasco et al. 2012 | Google earth | State | I | I |
| *Panstrongylus geniculatus* | Vector | Hemiptera | DTUI | 10.25000000 | -66.41670000 | Carrasco et al. 2012 | Google earth | State | I | I |
| *Panstrongylus geniculatus* | Vector | Hemiptera | DTUI | 10.25000000 | -66.41670000 | Carrasco et al. 2012 | Google earth | State | I | I |
| *Panstrongylus geniculatus* | Vector | Hemiptera | DTUI | 10.25000000 | -66.41670000 | Carrasco et al. 2012 | Google earth | State | I | I |
| *Panstrongylus geniculatus* | Vector | Hemiptera | DTUI | 10.25000000 | -66.41670000 | Carrasco et al. 2012 | Google earth | State | I | I |
| *Panstrongylus geniculatus* | Vector | Hemiptera | DTUI | 10.25000000 | -66.41670000 | Carrasco et al. 2012 | Google earth | State | I | I |
| *Panstrongylus geniculatus* | Vector | Hemiptera | DTUI | 10.25000000 | -66.41670000 | Carrasco et al. 2012 | Google earth | State | I | I |
| *Panstrongylus geniculatus* | Vector | Hemiptera | DTUI | 10.25000000 | -66.41670000 | Carrasco et al. 2012 | Google earth | State | I | I |
| *Panstrongylus geniculatus* | Vector | Hemiptera | DTUI | 10.25000000 | -66.41670000 | Carrasco et al. 2012 | Google earth | State | I | I |
| *Panstrongylus geniculatus* | Vector | Hemiptera | DTUI | 10.25000000 | -66.41670000 | Carrasco et al. 2012 | Google earth | State | I | I |
| *Panstrongylus geniculatus* | Vector | Hemiptera | DTUI | 10.25000000 | -66.41670000 | Carrasco et al. 2012 | Google earth | State | I | I |
| *Panstrongylus geniculatus* | Vector | Hemiptera | DTUI | 10.25000000 | -66.41670000 | Carrasco et al. 2012 | Google earth | State | I | I |
| *Panstrongylus geniculatus* | Vector | Hemiptera | DTUI | 10.25000000 | -66.41670000 | Carrasco et al. 2012 | Google earth | State | I | I |
| *Panstrongylus geniculatus* | Vector | Hemiptera | DTUI | 10.25000000 | -66.41670000 | Carrasco et al. 2012 | Google earth | State | I | I |
| *Panstrongylus geniculatus* | Vector | Hemiptera | DTUI | 10.25000000 | -66.41670000 | Carrasco et al. 2012 | Google earth | State | I | I |
| *Panstrongylus geniculatus* | Vector | Hemiptera | DTUI | 10.25000000 | -66.41670000 | Carrasco et al. 2012 | Google earth | State | I | I |
| *Panstrongylus geniculatus* | Vector | Hemiptera | DTUI | 10.25000000 | -66.41670000 | Carrasco et al. 2012 | Google earth | State | I | I |
| *Panstrongylus geniculatus* | Vector | Hemiptera | DTUI | 10.25000000 | -66.41670000 | Carrasco et al. 2012 | Google earth | State | I | I |
| *Panstrongylus geniculatus* | Vector | Hemiptera | DTUI | 10.25000000 | -66.41670000 | Carrasco et al. 2012 | Google earth | State | I | I |
| *Panstrongylus geniculatus* | Vector | Hemiptera | DTUI | 10.25000000 | -66.41670000 | Carrasco et al. 2012 | Google earth | State | I | I |
| *Panstrongylus geniculatus* | Vector | Hemiptera | DTUI | 10.25000000 | -66.41670000 | Carrasco et al. 2012 | Google earth | State | I | I |
| *Panstrongylus geniculatus* | Vector | Hemiptera | DTUI | 10.25000000 | -66.41670000 | Carrasco et al. 2012 | Google earth | State | I | I |
| *Panstrongylus geniculatus* | Vector | Hemiptera | DTUI | 10.25000000 | -66.41670000 | Carrasco et al. 2012 | Google earth | State | I | I |
| *Panstrongylus geniculatus* | Vector | Hemiptera | DTUI | 10.25000000 | -66.41670000 | Carrasco et al. 2012 | Google earth | State | I | I |
| *Panstrongylus geniculatus* | Vector | Hemiptera | DTUI | 10.25000000 | -66.41670000 | Carrasco et al. 2012 | Google earth | State | I | I |
| *Panstrongylus geniculatus* | Vector | Hemiptera | DTUI | 10.25000000 | -66.41670000 | Carrasco et al. 2012 | Google earth | State | I | I |
| *Panstrongylus geniculatus* | Vector | Hemiptera | DTUI | 10.25000000 | -66.41670000 | Carrasco et al. 2012 | Google earth | State | I | I |
| *Panstrongylus geniculatus* | Vector | Hemiptera | DTUI | 10.25000000 | -66.41670000 | Carrasco et al. 2012 | Google earth | State | I | I |
| *Panstrongylus geniculatus* | Vector | Hemiptera | DTUI | 10.25000000 | -66.41670000 | Carrasco et al. 2012 | Google earth | State | I | I |
| *Panstrongylus geniculatus* | Vector | Hemiptera | DTUI | 10.25000000 | -66.41670000 | Carrasco et al. 2012 | Google earth | State | I | I |
| *Panstrongylus geniculatus* | Vector | Hemiptera | DTUI | 10.25000000 | -66.41670000 | Carrasco et al. 2012 | Google earth | State | I | I |
| *Panstrongylus geniculatus* | Vector | Hemiptera | DTUI | 10.25000000 | -66.41670000 | Carrasco et al. 2012 | Google earth | State | I | I |
| *Panstrongylus geniculatus* | Vector | Hemiptera | DTUI | 10.25000000 | -66.41670000 | Carrasco et al. 2012 | Google earth | State | I | I |
| *Panstrongylus geniculatus* | Vector | Hemiptera | DTUI | 10.25000000 | -66.41670000 | Carrasco et al. 2012 | Google earth | State | I | I |
| *Panstrongylus geniculatus* | Vector | Hemiptera | DTUI | 10.25000000 | -66.41670000 | Carrasco et al. 2012 | Google earth | State | I | I |
| *Panstrongylus geniculatus* | Vector | Hemiptera | DTUI | 10.25000000 | -66.41670000 | Carrasco et al. 2012 | Google earth | State | I | I |
| *Panstrongylus geniculatus* | Vector | Hemiptera | DTUI | 10.25000000 | -66.41670000 | Carrasco et al. 2012 | Google earth | State | I | I |
| *Panstrongylus geniculatus* | Vector | Hemiptera | DTUI | 10.25000000 | -66.41670000 | Carrasco et al. 2012 | Google earth | State | I | I |
| *Panstrongylus geniculatus* | Vector | Hemiptera | DTUI | 10.25000000 | -66.41670000 | Carrasco et al. 2012 | Google earth | State | I | I |
| *Panstrongylus geniculatus* | Vector | Hemiptera | DTUI | 10.25000000 | -66.41670000 | Carrasco et al. 2012 | Google earth | State | I | I |
| *Panstrongylus geniculatus* | Vector | Hemiptera | DTUI | 10.25000000 | -66.41670000 | Carrasco et al. 2012 | Google earth | State | I | I |
| *Panstrongylus geniculatus* | Vector | Hemiptera | DTUI | 10.25000000 | -66.41670000 | Carrasco et al. 2012 | Google earth | State | I | I |
| *Panstrongylus geniculatus* | Vector | Hemiptera | DTUI | 10.25000000 | -66.41670000 | Carrasco et al. 2012 | Google earth | State | I | I |
| *Panstrongylus geniculatus* | Vector | Hemiptera | DTUI | 10.25000000 | -66.41670000 | Carrasco et al. 2012 | Google earth | State | I | I |
| *Panstrongylus geniculatus* | Vector | Hemiptera | DTUI | 10.25000000 | -66.41670000 | Carrasco et al. 2012 | Google earth | State | I | I |
| *Panstrongylus geniculatus* | Vector | Hemiptera | DTUI | 10.25000000 | -66.41670000 | Carrasco et al. 2012 | Google earth | State | I | I |
| *Panstrongylus geniculatus* | Vector | Hemiptera | DTUI | 10.25000000 | -66.41670000 | Carrasco et al. 2012 | Google earth | State | I | I |
| *Panstrongylus geniculatus* | Vector | Hemiptera | DTUI | 10.25000000 | -66.41670000 | Carrasco et al. 2012 | Google earth | State | I | I |
| *Panstrongylus geniculatus* | Vector | Hemiptera | DTUI | 10.25000000 | -66.41670000 | Carrasco et al. 2012 | Google earth | State | I | I |
| *Panstrongylus geniculatus* | Vector | Hemiptera | DTUI | 10.25000000 | -66.41670000 | Carrasco et al. 2012 | Google earth | State | I | I |
| *Panstrongylus geniculatus* | Vector | Hemiptera | DTUI | 10.25000000 | -66.41670000 | Carrasco et al. 2012 | Google earth | State | I | I |
| *Panstrongylus geniculatus* | Vector | Hemiptera | DTUI | 10.25000000 | -66.41670000 | Carrasco et al. 2012 | Google earth | State | I | I |
| *Panstrongylus geniculatus* | Vector | Hemiptera | DTUI | 10.25000000 | -66.41670000 | Carrasco et al. 2012 | Google earth | State | I | I |
| *Panstrongylus geniculatus* | Vector | Hemiptera | DTUI | 10.25000000 | -66.41670000 | Carrasco et al. 2012 | Google earth | State | I | I |
| *Panstrongylus geniculatus* | Vector | Hemiptera | DTUI | 10.25000000 | -66.41670000 | Carrasco et al. 2012 | Google earth | State | I | I |
| *Panstrongylus geniculatus* | Vector | Hemiptera | DTUI | 10.25000000 | -66.41670000 | Carrasco et al. 2012 | Google earth | State | I | I |
| *Panstrongylus geniculatus* | Vector | Hemiptera | DTUI | 10.25000000 | -66.41670000 | Carrasco et al. 2012 | Google earth | State | I | I |
| *Panstrongylus geniculatus* | Vector | Hemiptera | DTUI | 10.25000000 | -66.41670000 | Carrasco et al. 2012 | Google earth | State | I | I |
| *Panstrongylus geniculatus* | Vector | Hemiptera | DTUI | 10.25000000 | -66.41670000 | Carrasco et al. 2012 | Google earth | State | I | I |
| *Panstrongylus geniculatus* | Vector | Hemiptera | DTUI | 10.25000000 | -66.41670000 | Carrasco et al. 2012 | Google earth | State | I | I |
| *Panstrongylus geniculatus* | Vector | Hemiptera | DTUI | 10.25000000 | -66.41670000 | Carrasco et al. 2012 | Google earth | State | I | I |
| *Panstrongylus geniculatus* | Vector | Hemiptera | DTUI | 10.25000000 | -66.41670000 | Carrasco et al. 2012 | Google earth | State | I | I |
| *Panstrongylus geniculatus* | Vector | Hemiptera | DTUI | 10.25000000 | -66.41670000 | Carrasco et al. 2012 | Google earth | State | I | I |
| *Panstrongylus geniculatus* | Vector | Hemiptera | DTUI | 10.25000000 | -66.41670000 | Carrasco et al. 2012 | Google earth | State | I | I |
| *Panstrongylus geniculatus* | Vector | Hemiptera | DTUI | 10.25000000 | -66.41670000 | Carrasco et al. 2012 | Google earth | State | I | I |
| *Panstrongylus geniculatus* | Vector | Hemiptera | DTUI | 10.25000000 | -66.41670000 | Carrasco et al. 2012 | Google earth | State | I | I |
| *Panstrongylus geniculatus* | Vector | Hemiptera | DTUI | 10.25000000 | -66.41670000 | Carrasco et al. 2012 | Google earth | State | I | I |
| *Panstrongylus geniculatus* | Vector | Hemiptera | DTUI | 10.25000000 | -66.41670000 | Carrasco et al. 2012 | Google earth | State | I | I |
| *Panstrongylus geniculatus* | Vector | Hemiptera | DTUI | 10.25000000 | -66.41670000 | Carrasco et al. 2012 | Google earth | State | I | I |
| *Panstrongylus geniculatus* | Vector | Hemiptera | DTUI | 10.25000000 | -66.41670000 | Carrasco et al. 2012 | Google earth | State | I | I |
| *Panstrongylus geniculatus* | Vector | Hemiptera | DTUI | 10.25000000 | -66.41670000 | Carrasco et al. 2012 | Google earth | State | I | I |
| *Panstrongylus geniculatus* | Vector | Hemiptera | DTUI | 10.25000000 | -66.41670000 | Carrasco et al. 2012 | Google earth | State | I | I |
| *Panstrongylus geniculatus* | Vector | Hemiptera | DTUI | 10.25000000 | -66.41670000 | Carrasco et al. 2012 | Google earth | State | I | I |
| *Panstrongylus geniculatus* | Vector | Hemiptera | DTUI | 10.25000000 | -66.41670000 | Carrasco et al. 2012 | Google earth | State | I | I |
| *Panstrongylus geniculatus* | Vector | Hemiptera | DTUI | 10.25000000 | -66.41670000 | Carrasco et al. 2012 | Google earth | State | I | I |
| *Panstrongylus geniculatus* | Vector | Hemiptera | DTUI | 10.25000000 | -66.41670000 | Carrasco et al. 2012 | Google earth | State | I | I |
| *Panstrongylus geniculatus* | Vector | Hemiptera | DTUI | 10.25000000 | -66.41670000 | Carrasco et al. 2012 | Google earth | State | I | I |
| *Panstrongylus geniculatus* | Vector | Hemiptera | DTUI | 10.25000000 | -66.41670000 | Carrasco et al. 2012 | Google earth | State | I | I |
| *Panstrongylus geniculatus* | Vector | Hemiptera | DTUI | 10.25000000 | -66.41670000 | Carrasco et al. 2012 | Google earth | State | I | I |
| *Panstrongylus geniculatus* | Vector | Hemiptera | DTUI | 10.25000000 | -66.41670000 | Carrasco et al. 2012 | Google earth | State | I | I |
| *Panstrongylus geniculatus* | Vector | Hemiptera | DTUI | 10.25000000 | -66.41670000 | Carrasco et al. 2012 | Google earth | State | I | I |
| *Panstrongylus geniculatus* | Vector | Hemiptera | DTUI | 10.25000000 | -66.41670000 | Carrasco et al. 2012 | Google earth | State | I | I |
| *Panstrongylus geniculatus* | Vector | Hemiptera | DTUI | 10.25000000 | -66.41670000 | Carrasco et al. 2012 | Google earth | State | I | I |
| *Panstrongylus geniculatus* | Vector | Hemiptera | DTUI | 10.25000000 | -66.41670000 | Carrasco et al. 2012 | Google earth | State | I | I |
| *Panstrongylus geniculatus* | Vector | Hemiptera | DTUI | 10.25000000 | -66.41670000 | Carrasco et al. 2012 | Google earth | State | I | I |
| *Panstrongylus geniculatus* | Vector | Hemiptera | DTUI | 10.25000000 | -66.41670000 | Carrasco et al. 2012 | Google earth | State | I | I |
| *Panstrongylus geniculatus* | Vector | Hemiptera | DTUI | 10.25000000 | -66.41670000 | Carrasco et al. 2012 | Google earth | State | I | I |
| *Panstrongylus geniculatus* | Vector | Hemiptera | DTUI | 10.25000000 | -66.41670000 | Carrasco et al. 2012 | Google earth | State | I | I |
| *Panstrongylus geniculatus* | Vector | Hemiptera | DTUI | 10.25000000 | -66.41670000 | Carrasco et al. 2012 | Google earth | State | I | I |
| *Panstrongylus geniculatus* | Vector | Hemiptera | DTUI | 10.25000000 | -66.41670000 | Carrasco et al. 2012 | Google earth | State | I | I |
| *Panstrongylus geniculatus* | Vector | Hemiptera | DTUI | 10.25000000 | -66.41670000 | Carrasco et al. 2012 | Google earth | State | I | I |
| *Panstrongylus geniculatus* | Vector | Hemiptera | DTUI | 10.25000000 | -66.41670000 | Carrasco et al. 2012 | Google earth | State | I | I |
| *Panstrongylus geniculatus* | Vector | Hemiptera | DTUI | 10.25000000 | -66.41670000 | Carrasco et al. 2012 | Google earth | State | I | I |
| *Panstrongylus geniculatus* | Vector | Hemiptera | DTUI | 10.25000000 | -66.41670000 | Carrasco et al. 2012 | Google earth | State | I | I |
| *Panstrongylus geniculatus* | Vector | Hemiptera | DTUI | 10.25000000 | -66.41670000 | Carrasco et al. 2012 | Google earth | State | I | I |
| *Panstrongylus geniculatus* | Vector | Hemiptera | DTUI | 10.25000000 | -66.41670000 | Carrasco et al. 2012 | Google earth | State | I | I |
| *Panstrongylus geniculatus* | Vector | Hemiptera | DTUI | 10.25000000 | -66.41670000 | Carrasco et al. 2012 | Google earth | State | I | I |
| *Panstrongylus geniculatus* | Vector | Hemiptera | DTUI | 10.25000000 | -66.41670000 | Carrasco et al. 2012 | Google earth | State | I | I |
| *Panstrongylus geniculatus* | Vector | Hemiptera | DTUI | 10.25000000 | -66.41670000 | Carrasco et al. 2012 | Google earth | State | I | I |
| *Panstrongylus geniculatus* | Vector | Hemiptera | DTUI | 10.25000000 | -66.41670000 | Carrasco et al. 2012 | Google earth | State | I | I |
| *Panstrongylus geniculatus* | Vector | Hemiptera | DTUI | 10.25000000 | -66.41670000 | Carrasco et al. 2012 | Google earth | State | I | I |
| *Panstrongylus geniculatus* | Vector | Hemiptera | DTUI | 10.25000000 | -66.41670000 | Carrasco et al. 2012 | Google earth | State | I | I |
| *Panstrongylus geniculatus* | Vector | Hemiptera | DTUI | 10.25000000 | -66.41670000 | Carrasco et al. 2012 | Google earth | State | I | I |
| *Panstrongylus geniculatus* | Vector | Hemiptera | DTUI | 10.25000000 | -66.41670000 | Carrasco et al. 2012 | Google earth | State | I | I |
| *Panstrongylus geniculatus* | Vector | Hemiptera | DTUI | 10.25000000 | -66.41670000 | Carrasco et al. 2012 | Google earth | State | I | I |
| *Panstrongylus geniculatus* | Vector | Hemiptera | DTUI | 10.25000000 | -66.41670000 | Carrasco et al. 2012 | Google earth | State | I | I |
| *Panstrongylus geniculatus* | Vector | Hemiptera | DTUI | 10.25000000 | -66.41670000 | Carrasco et al. 2012 | Google earth | State | I | I |
| *Panstrongylus geniculatus* | Vector | Hemiptera | DTUI | 10.25000000 | -66.41670000 | Carrasco et al. 2012 | Google earth | State | I | I |
| *Panstrongylus geniculatus* | Vector | Hemiptera | DTUI | 10.25000000 | -66.41670000 | Carrasco et al. 2012 | Google earth | State | I | I |
| *Panstrongylus geniculatus* | Vector | Hemiptera | DTUI | 10.25000000 | -66.41670000 | Carrasco et al. 2012 | Google earth | State | I | I |
| *Panstrongylus geniculatus* | Vector | Hemiptera | DTUI | 10.25000000 | -66.41670000 | Carrasco et al. 2012 | Google earth | State | I | I |
| *Panstrongylus geniculatus* | Vector | Hemiptera | DTUI | 10.25000000 | -66.41670000 | Carrasco et al. 2012 | Google earth | State | I | I |
| *Panstrongylus geniculatus* | Vector | Hemiptera | DTUI | 10.25000000 | -66.41670000 | Carrasco et al. 2012 | Google earth | State | I | I |
| *Panstrongylus geniculatus* | Vector | Hemiptera | DTUI | 10.25000000 | -66.41670000 | Carrasco et al. 2012 | Google earth | State | I | I |
| *Panstrongylus geniculatus* | Vector | Hemiptera | DTUI | 10.25000000 | -66.41670000 | Carrasco et al. 2012 | Google earth | State | I | I |
| *Panstrongylus geniculatus* | Vector | Hemiptera | DTUI | 10.25000000 | -66.41670000 | Carrasco et al. 2012 | Google earth | State | I | I |
| *Panstrongylus geniculatus* | Vector | Hemiptera | DTUI | 10.25000000 | -66.41670000 | Carrasco et al. 2012 | Google earth | State | I | I |
| *Panstrongylus geniculatus* | Vector | Hemiptera | DTUI | 10.25000000 | -66.41670000 | Carrasco et al. 2012 | Google earth | State | I | I |
| *Panstrongylus geniculatus* | Vector | Hemiptera | DTUI | 10.25000000 | -66.41670000 | Carrasco et al. 2012 | Google earth | State | I | I |
| *Panstrongylus geniculatus* | Vector | Hemiptera | DTUI | 10.25000000 | -66.41670000 | Carrasco et al. 2012 | Google earth | State | I | I |
| *Panstrongylus geniculatus* | Vector | Hemiptera | DTUI | 10.25000000 | -66.41670000 | Carrasco et al. 2012 | Google earth | State | I | I |
| *Panstrongylus geniculatus* | Vector | Hemiptera | DTUI | 10.25000000 | -66.41670000 | Carrasco et al. 2012 | Google earth | State | I | I |
| *Panstrongylus geniculatus* | Vector | Hemiptera | DTUI | 10.25000000 | -66.41670000 | Carrasco et al. 2012 | Google earth | State | I | I |
| *Panstrongylus geniculatus* | Vector | Hemiptera | DTUI | 10.25000000 | -66.41670000 | Carrasco et al. 2012 | Google earth | State | I | I |
| *Panstrongylus geniculatus* | Vector | Hemiptera | DTUI | 10.25000000 | -66.41670000 | Carrasco et al. 2012 | Google earth | State | I | I |
| *Panstrongylus geniculatus* | Vector | Hemiptera | DTUI | 10.40000000 | -63.28300000 | Carrasco et al. 2012 | Google earth | State | I | I |
| *Panstrongylus geniculatus* | Vector | Hemiptera | DTUI | 10.40000000 | -63.28300000 | Carrasco et al. 2012 | Google earth | State | I | I |
| *Panstrongylus geniculatus* | Vector | Hemiptera | DTUI | 10.40000000 | -63.28300000 | Carrasco et al. 2012 | Google earth | State | I | I |
| *Panstrongylus geniculatus* | Vector | Hemiptera | DTUI | 10.51670000 | -67.06700000 | Carrasco et al. 2012 | Google earth | State | I | I |
| *Panstrongylus geniculatus* | Vector | Hemiptera | DTUI | 10.51670000 | -67.06700000 | Carrasco et al. 2012 | Google earth | State | I | I |
| *Panstrongylus geniculatus* | Vector | Hemiptera | DTUI | 10.51670000 | -67.06700000 | Carrasco et al. 2012 | Google earth | State | I | I |
| *Panstrongylus geniculatus* | Vector | Hemiptera | DTUI | 10.51670000 | -67.06700000 | Carrasco et al. 2012 | Google earth | State | I | I |
| *Panstrongylus geniculatus* | Vector | Hemiptera | DTUI | 10.51670000 | -67.06700000 | Carrasco et al. 2012 | Google earth | State | I | I |
| *Panstrongylus geniculatus* | Vector | Hemiptera | DTUI | 10.51670000 | -67.06700000 | Carrasco et al. 2012 | Google earth | State | I | I |
| *Panstrongylus geniculatus* | Vector | Hemiptera | DTUI | 10.51670000 | -67.06700000 | Carrasco et al. 2012 | Google earth | State | I | I |
| *Panstrongylus geniculatus* | Vector | Hemiptera | DTUI | 10.51670000 | -67.06700000 | Carrasco et al. 2012 | Google earth | State | I | I |
| *Panstrongylus geniculatus* | Vector | Hemiptera | DTUI | 10.51670000 | -67.06700000 | Carrasco et al. 2012 | Google earth | State | I | I |
| *Panstrongylus geniculatus* | Vector | Hemiptera | DTUI | 10.51670000 | -67.06700000 | Carrasco et al. 2012 | Google earth | State | I | I |
| *Panstrongylus geniculatus* | Vector | Hemiptera | DTUI | 10.51670000 | -67.06700000 | Carrasco et al. 2012 | Google earth | State | I | I |
| *Panstrongylus geniculatus* | Vector | Hemiptera | DTUI | 10.51670000 | -67.06700000 | Carrasco et al. 2012 | Google earth | State | I | I |
| *Panstrongylus geniculatus* | Vector | Hemiptera | DTUI | 10.51670000 | -67.06700000 | Carrasco et al. 2012 | Google earth | State | I | I |
| *Panstrongylus geniculatus* | Vector | Hemiptera | DTUI | 10.51670000 | -67.06700000 | Carrasco et al. 2012 | Google earth | State | I | I |
| *Panstrongylus geniculatus* | Vector | Hemiptera | DTUI | 10.51670000 | -67.06700000 | Carrasco et al. 2012 | Google earth | State | I | I |
| *Panstrongylus geniculatus* | Vector | Hemiptera | DTUI | 10.51670000 | -67.06700000 | Carrasco et al. 2012 | Google earth | State | I | I |
| *Panstrongylus geniculatus* | Vector | Hemiptera | DTUI | 10.51670000 | -67.06700000 | Carrasco et al. 2012 | Google earth | State | I | I |
| *Panstrongylus geniculatus* | Vector | Hemiptera | DTUI | 10.51670000 | -67.06700000 | Carrasco et al. 2012 | Google earth | State | I | I |
| *Panstrongylus geniculatus* | Vector | Hemiptera | DTUIII | 8.30000000 | -70.05000000 | Carrasco et al. 2012 | Google earth | State | II | III |
| *Panstrongylus geniculatus* | Vector | Hemiptera | DTUIII | 10.25000000 | -66.41670000 | Carrasco et al. 2012 | Google earth | State | II | III |
| *Panstrongylus geniculatus* | Vector | Hemiptera | DTUIII | 10.25000000 | -66.41670000 | Carrasco et al. 2012 | Google earth | State | II | III |
| *Panstrongylus geniculatus* | Vector | Hemiptera | DTUIII | 9.08300000 | -69.08300000 | Carrasco et al. 2012 | Google earth | State | II | III |
| *Panstrongylus geniculatus* | Vector | Hemiptera | DTUIII | 10.40000000 | -63.28300000 | Carrasco et al. 2012 | Google earth | State | II | III |
| *Panstrongylus geniculatus* | Vector | Hemiptera | DTUIII | 10.40000000 | -63.28300000 | Carrasco et al. 2012 | Google earth | State | II | III |
| *Panstrongylus geniculatus* | Vector | Hemiptera | DTUIII | 10.40000000 | -63.28300000 | Carrasco et al. 2012 | Google earth | State | II | III |
| *Panstrongylus geniculatus* | Vector | Hemiptera | DTUIII | 10.40000000 | -63.28300000 | Carrasco et al. 2012 | Google earth | State | II | III |
| *Panstrongylus geniculatus* | Vector | Hemiptera | DTUIII | 10.51670000 | -67.06700000 | Carrasco et al. 2012 | Google earth | State | II | III |
| *Panstrongylus geniculatus* | Vector | Hemiptera | DTUIV | 10.51670000 | -67.06700000 | Carrasco et al. 2012 | Google earth | State | II | III |
| *Panstrongylus geniculatus* | Vector | Hemiptera | DTUI | 10.04000000 | -64.32000000 | Llewellyn et al. 2009b | Author | NA | I | I |
| *Panstrongylus geniculatus* | Vector | Hemiptera | DTUI | 4.92222222 | -52.31333333 | Cura et al. 2015 | Google earth | Locality | I | I |
| *Panstrongylus geniculatus* | Vector | Hemiptera | DTUI | 4.92222222 | -52.31333333 | Cura et al. 2015 | Google earth | Locality | I | I |
| *Panstrongylus geniculatus* | Vector | Hemiptera | DTUI | 4.83166667 | -52.34527778 | Cura et al. 2015 | Google earth | Locality | I | I |
| *Panstrongylus geniculatus* | Vector | Hemiptera | DTUI | 4.83166667 | -52.34527778 | Cura et al. 2015 | Google earth | Locality | I | I |
| *Panstrongylus geniculatus* | Vector | Hemiptera | DTUI | 4.88250000 | -52.28666667 | Cura et al. 2015 | Google earth | Locality | I | I |
| *Panstrongylus geniculatus* | Vector | Hemiptera | DTUI | 4.88250000 | -52.28666667 | Cura et al. 2015 | Google earth | Locality | I | I |
| *Panstrongylus geniculatus* | Vector | Hemiptera | DTUI | 4.88250000 | -52.28666667 | Cura et al. 2015 | Google earth | Locality | I | I |
| *Panstrongylus geniculatus* | Vector | Hemiptera | DTUIII | 4.51444444 | -52.06638889 | Cura et al. 2015 | Google earth | Locality | II | III |
| *Panstrongylus geniculatus* | Vector | Hemiptera | DTUIII | 4.83166667 | -52.34527778 | Cura et al. 2015 | Google earth | Locality | II | III |
| *Panstrongylus geniculatus* | Vector | Hemiptera | DTUIII | 4.88250000 | -52.28666667 | Cura et al. 2015 | Google earth | Locality | II | III |
| *Panstrongylus geniculatus* | Vector | Hemiptera | DTUIII | 4.88250000 | -52.28666667 | Cura et al. 2015 | Google earth | Locality | II | III |
| *Panstrongylus geniculatus* | Vector | Hemiptera | DTUIV | 5.48083333 | -53.20972222 | Cura et al. 2015 | Google earth | Locality | II | III |
| *Panstrongylus geniculatus* | Vector | Hemiptera | DTUIV | 4.51444444 | -52.06638889 | Cura et al. 2015 | Google earth | Locality | II | III |
| *Panstrongylus geniculatus* | Vector | Hemiptera | DTUI | 4.92111111 | -52.48916667 | Cura et al. 2015 | Google earth | Locality | I | I |
| *Panstrongylus geniculatus* | Vector | Hemiptera | DTUIII | 4.92111111 | -52.48916667 | Cura et al. 2015 | Google earth | Locality | II | III |
| *Panstrongylus herreri* | Vector | Hemiptera | DTUI | -7.161667 | -78.512778 | Cura et al. 2015 | Google earth | Locality | I | I |
| *Panstrongylus herreri* | Vector | Hemiptera | DTUI | -7.161667 | -78.512778 | Cura et al. 2015 | Google earth | Locality | I | I |
| *Panstrongylus herreri* | Vector | Hemiptera | DTUIV | -7.161667 | -78.512778 | Cura et al. 2015 | Google earth | Locality | II | III |
| *Panstrongylus herreri* | Vector | Hemiptera | DTUIV | -7.161667 | -78.512778 | Cura et al. 2015 | Google earth | Locality | II | III |
| *Panstrongylus herreri* | Vector | Hemiptera | DTUIV | -7.161667 | -78.512778 | Cura et al. 2015 | Google earth | Locality | II | III |
| *Panstrongylus herreri* | Vector | Hemiptera | DTUIV | -7.161667 | -78.512778 | Cura et al. 2015 | Google earth | Locality | II | III |
| *Panstrongylus lignarius* | Vector | Hemiptera | DTUI | 4.27638889 | -52.64305556 | Cura et al. 2015 | Google earth | Locality | I | I |
| *Panstrongylus lignarius* | Vector | Hemiptera | DTUI | 4.51444444 | -52.06638889 | Cura et al. 2015 | Google earth | Locality | I | I |
| *Panstrongylus lignarius* | Vector | Hemiptera | DTUI | 3.86027778 | -53.30416667 | Cura et al. 2015 | Google earth | Locality | I | I |
| *Panstrongylus lignarius* | Vector | Hemiptera | DTUIV | 4.27648500 | -52.64305556 | Cura et al. 2015 | Google earth | Locality | II | III |
| *Panstrongylus lignarius* | Vector | Hemiptera | DTUIV | 3.86027778 | -52.06638889 | Cura et al. 2015 | Google earth | Locality | II | III |
| *Panstrongylus lignarius* | Vector | Hemiptera | DTUIV | 31.96833333 | -99.90166667 | Cura et al. 2015 | Google earth | Locality | II | III |
| *Panstrongylus lutzi* | Vector | Hemiptera | DTUIII | -5.70000000 | -37.55000000 | Camara et al. 2010 | Google earth | Area/Unspecified communities | II | III |
| *Panstrongylus lutzi* | Vector | Hemiptera | DTUIII | -5.70000000 | -37.55000000 | Camara et al. 2010 | Google earth | Area/Unspecified communities | II | III |
| *Panstrongylus megistus* | Vector | Hemiptera | DTUI | -23.55000 | -52.20000 | Zalloum et al. 2005 | Google earth | Community | I | I |
| *Panstrongylus rufotuberculatus* | Vector | Hemiptera | DTUI | 10.25000000 | -66.41670000 | Carrasco et al. 2012 | Google earth | State | I | I |
| *Panstrongylus rufotuberculatus* | Vector | Hemiptera | DTUI | 17.48472222 | -92.04583333 | Cura et al. 2015 | Google earth | Locality | I | I |
| *Panstrongylus rufotuberculatus* | Vector | Hemiptera | DTUI | 17.48472222 | -92.04583333 | Cura et al. 2015 | Google earth | Locality | I | I |
| *Panstrongylus spp* | Vector | Hemiptera | DTUIII | 8.48000000 | -70.73000000 | Llewellyn et al. 2009a | Author | NA | II | III |
| *Paramelomys levipes* | Host | Rodentia | DTUI | 18.68916667 | -98.77527778 | Cura et al. 2015 | Google earth | Locality | I | I |
| *Panstrongylus geniculatus* | Vector | Hemiptera | DTUI | 7.18300000 | -75.03300000 | Mejia-Jaramillo et al. 2009 | Google earth | Department/State | I | I |
| *Panstrongylus geniculatus* | Vector | Hemiptera | DTUI | 9.13000000 | -75.05000000 | Mejia-Jaramillo et al. 2009 | Google earth | Department/State | I | I |
| *Panstrongylus geniculatus* | Vector | Hemiptera | DTUI | 9.13000000 | -75.05000000 | Mejia-Jaramillo et al. 2009 | Google earth | Department/State | I | I |
| *Panstrongylus geniculatus* | Vector | Hemiptera | DTUI | 10.40000000 | -74.40000000 | Mejia-Jaramillo et al. 2009 | Google earth | Department/State | I | I |
| *Panstrongylus geniculatus* | Vector | Hemiptera | DTUIII | -3.11600000 | -60.01600000 | Marcili et al. 2009b | Google earth | Locality | II | III |
| *Peropteryx macrotis* | Host | Chiroptera | DTUI | -5.4025 | -36.95389 | Lima et al. 2015 | Google earth | State | I | I |
| *Philander frenata* | Host | Didelphimorphia | DTUIII | -23.76700000 | -45.35000000 | Marcili et al. 2009b | Google earth | Locality | II | III |
| *Philander opossum* | Host | Didelphimorphia | DTUI | 32.43300000 | -81.76700000 | Roellig et al. 2013 | Google earth | Community | I | I |
| *Philander opossum* | Host | Didelphimorphia | DTUI | 31.86700000 | -81.60000000 | Roellig et al. 2013 | Google earth | Community | I | I |
| *Philander opossum* | Host | Didelphimorphia | DTUI | 31.21670000 | -84.30000000 | Roellig et al. 2013 | Google earth | Community | I | I |
| *Philander opossum* | Host | Didelphimorphia | DTUI | 30.23400000 | -84.30000000 | Roellig et al. 2013 | Google earth | Community | I | I |
| *Philander opossum* | Host | Didelphimorphia | DTUI | 30.51600000 | -84.25000000 | Roellig et al. 2013 | Google earth | Community | I | I |
| *Philander opossum* | Host | Didelphimorphia | DTUI | 29.95000000 | -90.06700000 | Roellig et al. 2013 | Google earth | Community | I | I |
| *Philander opossum* | Host | Didelphimorphia | DTUI | -14.81000000 | -64.60000000 | Llewellyn et al. 2009b | Author | NA | I | I |
| *Philander opossum* | Host | Didelphimorphia | DTUI | -14.81000000 | -64.60000000 | Llewellyn et al. 2009b | Author | NA | I | I |
| *Philander opossum* | Host | Didelphimorphia | DTUI | -14.81000000 | -64.60000000 | Llewellyn et al. 2009b | Author | NA | I | I |
| *Philander opossum* | Host | Didelphimorphia | DTUI | -1.50000000 | -48.18000000 | Llewellyn et al. 2009b | Author | NA | I | I |
| *Philander opossum* | Host | Didelphimorphia | DTUI | -2.61000000 | -49.53000000 | Llewellyn et al. 2009b | Author | NA | I | I |
| *Philander opossum* | Host | Didelphimorphia | DTUI | -1.36000000 | -48.36000000 | Llewellyn et al. 2009b | Author | NA | I | I |
| *Philander opossum* | Host | Didelphimorphia | DTUI | -5.98000000 | -51.33000000 | Llewellyn et al. 2009b | Author | NA | I | I |
| *Philander opossum* | Host | Didelphimorphia | DTUI | -1.36000000 | -48.36000000 | Llewellyn et al. 2009b | Author | NA | I | I |
| *Philander opossum* | Host | Didelphimorphia | DTUI | -2.32000000 | -49.52000000 | Llewellyn et al. 2009b | Author | NA | I | I |
| *Philander opossum* | Host | Didelphimorphia | DTUI | -3.66000000 | -49.63000000 | Llewellyn et al. 2009b | Author | NA | I | I |
| *Philander opossum* | Host | Didelphimorphia | DTUI | -1.86000000 | -52.20000000 | Llewellyn et al. 2009b | Author | NA | I | I |
| *Philander opossum* | Host | Didelphimorphia | DTUI | -14.81000000 | -64.60000000 | Messenger et al. 2012 | Author | NA | I | I |
| *Philander opossum* | Host | Didelphimorphia | DTUI | -14.81000000 | -64.60000000 | Messenger et al. 2012 | Author | NA | I | I |
| *Phyllostomus hastatus* | Host | Chiroptera | DTUI | -5.13400 | -49.31670 | Lisboa et al. 2008 | Google earth | Biomas Brasil | I | I |
| *Phyllostomus hastatus* | Host | Chiroptera | ND | -18.78300 | -51.91600 | Lisboa et al. 2008 | Google earth | Biomas Brasil | II | ND |
| *Phyllostomus hastatus* | Host | Chiroptera | ND | -18.78300 | -51.91600 | Lisboa et al. 2008 | Google earth | Biomas Brasil | II | ND |
| *Phyllostomus hastatus* | Host | Chiroptera | ND | -18.78300 | -51.91600 | Lisboa et al. 2008 | Google earth | Biomas Brasil | II | ND |
| *Phyllostomus hastatus* | Host | Chiroptera | ND | -18.78300 | -51.91600 | Lisboa et al. 2008 | Google earth | Biomas Brasil | II | ND |
| *Phyllostomus hastatus* | Host | Chiroptera | ND | -18.78300 | -51.91600 | Lisboa et al. 2008 | Google earth | Biomas Brasil | II | ND |
| *Phyllostomus hastatus* | Host | Chiroptera | ND | -18.78300 | -51.91600 | Lisboa et al. 2008 | Google earth | Biomas Brasil | II | ND |
| *Phyllostomus hastatus* | Host | Chiroptera | ND | -18.78300 | -51.91600 | Lisboa et al. 2008 | Google earth | Biomas Brasil | II | ND |
| *Phyllostomus hastatus* | Host | Chiroptera | ND | -19.23400 | -57.01600 | Lisboa et al. 2008 | Google earth | Biomas Brasil | II | ND |
| *Phyllostomus hastatus* | Host | Chiroptera | ND | -19.23400 | -57.01600 | Lisboa et al. 2008 | Google earth | Biomas Brasil | II | ND |
| *Phyllostomus hastatus* | Host | Chiroptera | DTUII | -23.5505 | -46.633 | Lima et al. 2015 | Google earth | State | II | II |
| *Phyllotis osae* | Host | Rodentia | DTUI | -17.43000000 | -66.27000000 | Llewellyn et al. 2009b | Author | NA | I | I |
| *Phyllotis osae* | Host | Rodentia | DTUI | -17.43000000 | -66.27000000 | Llewellyn et al. 2009b | Author | NA | I | I |
| *Phyllotis osae* | Host | Rodentia | DTUI | -17.43000000 | -66.27000000 | Llewellyn et al. 2009b | Author | NA | I | I |
| *Platyrrhinus lineatus* | Host | Chiroptera | DTUI | -10.175278 | -48,29805 | Lima et al. 2015 | Google earth | State | I | I |
| *Platyrrhinus lineatus* | Host | Chiroptera | DTUII | -10.175278 | -48,29805 | Lima et al. 2015 | Google earth | State | II | II |
| *Potos flavus* | Host | Carnivora | DTUI | 3.98000000 | -76.96000000 | Llewellyn et al. 2009b | Author | NA | I | I |
| *Procyon lotor* | Host | Carnivora | DTUIV | 32.43300000 | -81.76600000 | Roellig et al. 2013 | Google earth | Community | II | III |
| *Procyon lotor* | Host | Carnivora | DTUIV | 32.43300000 | -81.76600000 | Roellig et al. 2013 | Google earth | Community | II | III |
| *Procyon lotor* | Host | Carnivora | DTUIV | 31.86600000 | -81.60000000 | Roellig et al. 2013 | Google earth | Community | II | III |
| *Procyon lotor* | Host | Carnivora | DTUIV | 31.86600000 | -81.60000000 | Roellig et al. 2013 | Google earth | Community | II | III |
| *Procyon lotor* | Host | Carnivora | DTUIV | 30.51670000 | -84.25000000 | Roellig et al. 2013 | Google earth | Community | II | III |
| *Procyon lotor* | Host | Carnivora | DTUIV | 30.23400000 | -84.30000000 | Roellig et al. 2013 | Google earth | Community | II | III |
| *Procyon lotor* | Host | Carnivora | DTUIV | 30.23400000 | -84.30000000 | Roellig et al. 2013 | Google earth | Community | II | III |
| *Procyon lotor* | Host | Carnivora | DTUIV | 30.65000000 | -84.20000000 | Roellig et al. 2013 | Google earth | Community | II | III |
| *Procyon lotor* | Host | Carnivora | DTUIV | 30.56600000 | -84.93300000 | Roellig et al. 2013 | Google earth | Community | II | III |
| *Procyon lotor* | Host | Carnivora | DTUIV | 30.56600000 | -84.93300000 | Roellig et al. 2013 | Google earth | Community | II | III |
| *Procyon lotor* | Host | Carnivora | DTUIV | 30.43300000 | -84.56600000 | Roellig et al. 2013 | Google earth | Community | II | III |
| *Procyon lotor* | Host | Carnivora | DTUIV | 31.75000000 | -81.10000000 | Roellig et al. 2013 | Google earth | Community | II | III |
| *Procyon lotor* | Host | Carnivora | DTUIV | 33.68300000 | -84.30000000 | Roellig et al. 2013 | Google earth | Community | II | III |
| *Procyon lotor* | Host | Carnivora | DTUIV | 33.95000000 | -83.38300000 | Roellig et al. 2013 | Google earth | Community | II | III |
| *Procyon lotor* | Host | Carnivora | DTUIV | 33.95000000 | -83.38300000 | Roellig et al. 2013 | Google earth | Community | II | III |
| *Procyon lotor* | Host | Carnivora | DTUIV | 31.93000000 | -81.03300000 | Roellig et al. 2013 | Google earth | Community | II | III |
| *Procyon lotor* | Host | Carnivora | DTUIV | 39.08300000 | -76.08300000 | Roellig et al. 2013 | Google earth | Community | II | III |
| *Procyon lotor* | Host | Carnivora | DTUIV | 31.70000000 | -81.26700000 | Roellig et al. 2013 | Google earth | Community | II | III |
| *Procyon lotor* | Host | Carnivora | DTUIV | 31.70000000 | -81.26700000 | Roellig et al. 2013 | Google earth | Community | II | III |
| *Procyon lotor* | Host | Carnivora | DTUIV | 35.76700000 | -86.03400000 | Roellig et al. 2013 | Google earth | Community | II | III |
| *Proechimys semiespinosus* | Host | Rodentia | DTUI | 10.95000000 | -73.46700000 | Rodriguez et al. 2009 | Google earth | Area/Unspecified communities | I | I |
| *Proechimys semiespinosus* | Host | Rodentia | DTUI | 10.95000000 | -73.46700000 | Rodriguez et al. 2009 | Google earth | Area/Unspecified communities | I | I |
| *Rattus norvegicus* | Host | Rodentia | DTUI | 8.30000000 | -70.05000000 | Carrasco et al. 2012 | Google earth | State | I | I |
| *Rattus rattus* | Host | Rodentia | DTUI | 10.54000000 | -67.80000000 | Llewellyn et al. 2009b | Author | NA | I | I |
| *Rattus rattus* | Host | Rodentia | DTUI | 10.31000000 | -66.39000000 | Llewellyn et al. 2009b | Author | NA | I | I |
| *Rattus rattus* | Host | Rodentia | DTUI | 9.55000000 | -70.51000000 | Llewellyn et al. 2009b | Author | NA | I | I |
| *Rattus rattus* | Host | Rodentia | DTUI | 10.05000000 | -66.95000000 | Carrasco et al. 2012 | Google earth | State | I | I |
| *Rattus rattus* | Host | Rodentia | DTUI | 10.05000000 | -66.95000000 | Carrasco et al. 2012 | Google earth | State | I | I |
| *Rattus rattus* | Host | Rodentia | DTUI | 10.05000000 | -66.95000000 | Carrasco et al. 2012 | Google earth | State | I | I |
| *Rattus rattus* | Host | Rodentia | DTUI | 10.05000000 | -66.95000000 | Carrasco et al. 2012 | Google earth | State | I | I |
| *Rattus rattus* | Host | Rodentia | DTUI | 10.05000000 | -66.95000000 | Carrasco et al. 2012 | Google earth | State | I | I |
| *Rattus rattus* | Host | Rodentia | DTUI | 10.05000000 | -66.95000000 | Carrasco et al. 2012 | Google earth | State | I | I |
| *Rattus rattus* | Host | Rodentia | DTUI | 10.05000000 | -66.95000000 | Carrasco et al. 2012 | Google earth | State | I | I |
| *Rattus rattus* | Host | Rodentia | DTUI | 10.05000000 | -66.95000000 | Carrasco et al. 2012 | Google earth | State | I | I |
| *Rattus rattus* | Host | Rodentia | DTUI | 10.05000000 | -66.95000000 | Carrasco et al. 2012 | Google earth | State | I | I |
| *Rattus rattus* | Host | Rodentia | DTUI | 10.05000000 | -66.95000000 | Carrasco et al. 2012 | Google earth | State | I | I |
| *Rattus rattus* | Host | Rodentia | DTUI | 10.05000000 | -66.95000000 | Carrasco et al. 2012 | Google earth | State | I | I |
| *Rattus rattus* | Host | Rodentia | DTUI | 10.05000000 | -66.95000000 | Carrasco et al. 2012 | Google earth | State | I | I |
| *Rattus rattus* | Host | Rodentia | DTUI | 10.05000000 | -66.95000000 | Carrasco et al. 2012 | Google earth | State | I | I |
| *Rattus rattus* | Host | Rodentia | DTUI | 10.05000000 | -66.95000000 | Carrasco et al. 2012 | Google earth | State | I | I |
| *Rattus rattus* | Host | Rodentia | DTUI | 10.05000000 | -66.95000000 | Carrasco et al. 2012 | Google earth | State | I | I |
| *Rattus rattus* | Host | Rodentia | DTUI | 10.05000000 | -66.95000000 | Carrasco et al. 2012 | Google earth | State | I | I |
| *Rattus rattus* | Host | Rodentia | DTUI | 10.05000000 | -66.95000000 | Carrasco et al. 2012 | Google earth | State | I | I |
| *Rattus rattus* | Host | Rodentia | DTUI | 10.05000000 | -66.95000000 | Carrasco et al. 2012 | Google earth | State | I | I |
| *Rattus rattus* | Host | Rodentia | DTUI | 10.05000000 | -66.95000000 | Carrasco et al. 2012 | Google earth | State | I | I |
| *Rattus rattus* | Host | Rodentia | DTUI | 10.05000000 | -66.95000000 | Carrasco et al. 2012 | Google earth | State | I | I |
| *Rattus rattus* | Host | Rodentia | DTUI | 10.05000000 | -66.95000000 | Carrasco et al. 2012 | Google earth | State | I | I |
| *Rattus rattus* | Host | Rodentia | DTUI | 10.05000000 | -66.95000000 | Carrasco et al. 2012 | Google earth | State | I | I |
| *Rattus rattus* | Host | Rodentia | DTUI | 10.05000000 | -66.95000000 | Carrasco et al. 2012 | Google earth | State | I | I |
| *Rattus rattus* | Host | Rodentia | DTUI | 10.05000000 | -66.95000000 | Carrasco et al. 2012 | Google earth | State | I | I |
| *Rattus rattus* | Host | Rodentia | DTUI | 10.05000000 | -66.95000000 | Carrasco et al. 2012 | Google earth | State | I | I |
| *Rattus rattus* | Host | Rodentia | DTUI | 10.05000000 | -66.95000000 | Carrasco et al. 2012 | Google earth | State | I | I |
| *Rattus rattus* | Host | Rodentia | DTUI | 10.05000000 | -66.95000000 | Carrasco et al. 2012 | Google earth | State | I | I |
| *Rattus rattus* | Host | Rodentia | DTUI | 10.05000000 | -66.95000000 | Carrasco et al. 2012 | Google earth | State | I | I |
| *Rattus rattus* | Host | Rodentia | DTUI | 10.05000000 | -66.95000000 | Carrasco et al. 2012 | Google earth | State | I | I |
| *Rattus rattus* | Host | Rodentia | DTUI | 10.05000000 | -66.95000000 | Carrasco et al. 2012 | Google earth | State | I | I |
| *Rattus rattus* | Host | Rodentia | DTUI | 10.05000000 | -66.95000000 | Carrasco et al. 2012 | Google earth | State | I | I |
| *Rattus rattus* | Host | Rodentia | DTUI | 10.05000000 | -66.95000000 | Carrasco et al. 2012 | Google earth | State | I | I |
| *Rattus rattus* | Host | Rodentia | DTUI | 10.05000000 | -66.95000000 | Carrasco et al. 2012 | Google earth | State | I | I |
| *Rattus rattus* | Host | Rodentia | DTUI | 10.05000000 | -66.95000000 | Carrasco et al. 2012 | Google earth | State | I | I |
| *Rattus rattus* | Host | Rodentia | DTUI | 10.05000000 | -66.95000000 | Carrasco et al. 2012 | Google earth | State | I | I |
| *Rattus rattus* | Host | Rodentia | DTUI | 10.05000000 | -66.95000000 | Carrasco et al. 2012 | Google earth | State | I | I |
| *Rattus rattus* | Host | Rodentia | DTUI | 10.05000000 | -66.95000000 | Carrasco et al. 2012 | Google earth | State | I | I |
| *Rattus rattus* | Host | Rodentia | DTUI | 10.05000000 | -66.95000000 | Carrasco et al. 2012 | Google earth | State | I | I |
| *Rattus rattus* | Host | Rodentia | DTUI | 10.05000000 | -66.95000000 | Carrasco et al. 2012 | Google earth | State | I | I |
| *Rattus rattus* | Host | Rodentia | DTUI | 10.05000000 | -66.95000000 | Carrasco et al. 2012 | Google earth | State | I | I |
| *Rattus rattus* | Host | Rodentia | DTUI | 10.05000000 | -66.95000000 | Carrasco et al. 2012 | Google earth | State | I | I |
| *Rattus rattus* | Host | Rodentia | DTUI | 10.05000000 | -66.95000000 | Carrasco et al. 2012 | Google earth | State | I | I |
| *Rattus rattus* | Host | Rodentia | DTUI | 10.05000000 | -66.95000000 | Carrasco et al. 2012 | Google earth | State | I | I |
| *Rattus rattus* | Host | Rodentia | DTUI | 10.05000000 | -66.95000000 | Carrasco et al. 2012 | Google earth | State | I | I |
| *Rattus rattus* | Host | Rodentia | DTUI | 10.25000000 | -66.41670000 | Carrasco et al. 2012 | Google earth | State | I | I |
| *Rattus rattus* | Host | Rodentia | DTUI | 10.25000000 | -66.41670000 | Carrasco et al. 2012 | Google earth | State | I | I |
| *Rattus rattus* | Host | Rodentia | DTUI | 9.36700000 | -70.41670000 | Carrasco et al. 2012 | Google earth | State | I | I |
| *Rattus rattus* | Host | Rodentia | DTUI | -4.07200000 | -79.83400000 | Ocaña-Mayorga et al. 2010 | Author | NA | I | I |
| *Rattus rattus* | Host | Rodentia | DTUI | -4.21000000 | -80.09900000 | Ocaña-Mayorga et al. 2010 | Author | NA | I | I |
| *Rattus rattus* | Host | Rodentia | DTUI | -4.09500000 | -79.35200000 | Ocaña-Mayorga et al. 2010 | Author | NA | I | I |
| *Rattus rattus* | Host | Rodentia | DTUI | -4.06200000 | -79.20500000 | Ocaña-Mayorga et al. 2010 | Author | NA | I | I |
| *Rattus spp* | Host | Rodentia | DTUI | 14.18300000 | -90.36700000 | Higo et al. 2004 | Google earth | Department/State | I | I |
| *Rhodnius brethesi* | Vector | Hemiptera | DTUI | -0.26700000 | -63.20000000 | Marcili et al. 2009b | Google earth | Locality | I | I |
| *Rhodnius brethesi* | Vector | Hemiptera | DTUI | -0.26700000 | -63.20000000 | Marcili et al. 2009b | Google earth | Locality | I | I |
| *Rhodnius brethesi* | Vector | Hemiptera | DTUIV | -0.26700000 | -63.20000000 | Marcili et al. 2009b | Google earth | Locality | II | III |
| *Rhodnius brethesi* | Vector | Hemiptera | DTUIV | -0.26700000 | -63.20000000 | Marcili et al. 2009b | Google earth | Locality | II | III |
| *Rhodnius brethesi* | Vector | Hemiptera | DTUIV | -0.26700000 | -63.20000000 | Marcili et al. 2009b | Google earth | Locality | II | III |
| *Rhodnius brethesi* | Vector | Hemiptera | DTUIV | -0.26700000 | -63.20000000 | Marcili et al. 2009b | Google earth | Locality | II | III |
| *Rhodnius ecuadoriensis* | Vector | Hemiptera | DTUI | -4.07900000 | -79.82500000 | Ocaña-Mayorga et al. 2010 | Author | NA | I | I |
| *Rhodnius ecuadoriensis* | Vector | Hemiptera | DTUI | -4.10600000 | -79.34700000 | Ocaña-Mayorga et al. 2010 | Author | NA | I | I |
| *Rhodnius ecuadoriensis* | Vector | Hemiptera | DTUI | -4.09600000 | -79.34400000 | Ocaña-Mayorga et al. 2010 | Author | NA | I | I |
| *Rhodnius ecuadoriensis* | Vector | Hemiptera | DTUI | -4.35300000 | -79.43200000 | Ocaña-Mayorga et al. 2010 | Author | NA | I | I |
| *Rhodnius ecuadoriensis* | Vector | Hemiptera | DTUI | -4.23700000 | -79.59900000 | Ocaña-Mayorga et al. 2010 | Author | NA | I | I |
| *Rhodnius ecuadoriensis* | Vector | Hemiptera | DTUI | -4.09800000 | -79.34300000 | Ocaña-Mayorga et al. 2010 | Author | NA | I | I |
| *Rhodnius ecuadoriensis* | Vector | Hemiptera | DTUI | -4.07700000 | -79.80800000 | Ocaña-Mayorga et al. 2010 | Author | NA | I | I |
| *Rhodnius ecuadoriensis* | Vector | Hemiptera | DTUI | -4.19600000 | -80.03400000 | Ocaña-Mayorga et al. 2010 | Author | NA | I | I |
| *Rhodnius ecuadoriensis* | Vector | Hemiptera | DTUI | -4.07900000 | -79.82500000 | Ocaña-Mayorga et al. 2010 | Author | NA | I | I |
| *Rhodnius ecuadoriensis* | Vector | Hemiptera | DTUI | -4.07500000 | -79.80900000 | Ocaña-Mayorga et al. 2010 | Author | NA | I | I |
| *Rhodnius ecuadoriensis* | Vector | Hemiptera | DTUI | -4.07700000 | -79.82200000 | Ocaña-Mayorga et al. 2010 | Author | NA | I | I |
| *Rhodnius ecuadoriensis* | Vector | Hemiptera | DTUI | -4.07700000 | -79.80900000 | Ocaña-Mayorga et al. 2010 | Author | NA | I | I |
| *Rhodnius ecuadoriensis* | Vector | Hemiptera | DTUI | -4.07600000 | -79.80900000 | Ocaña-Mayorga et al. 2010 | Author | NA | I | I |
| *Rhodnius ecuadoriensis* | Vector | Hemiptera | DTUI | -4.07800000 | -79.80700000 | Ocaña-Mayorga et al. 2010 | Author | NA | I | I |
| *Rhodnius ecuadoriensis* | Vector | Hemiptera | DTUI | -4.08000000 | -79.82400000 | Ocaña-Mayorga et al. 2010 | Author | NA | I | I |
| *Rhodnius ecuadoriensis* | Vector | Hemiptera | DTUI | -4.03900000 | -79.76100000 | Ocaña-Mayorga et al. 2010 | Author | NA | I | I |
| *Rhodnius ecuadoriensis* | Vector | Hemiptera | DTUI | -4.09600000 | -79.34400000 | Ocaña-Mayorga et al. 2010 | Author | NA | I | I |
| *Rhodnius ecuadoriensis* | Vector | Hemiptera | DTUI | -4.10600000 | -79.34700000 | Ocaña-Mayorga et al. 2010 | Author | NA | I | I |
| *Rhodnius ecuadoriensis* | Vector | Hemiptera | DTUI | -4.10600000 | -79.34700000 | Ocaña-Mayorga et al. 2010 | Author | NA | I | I |
| *Rhodnius ecuadoriensis* | Vector | Hemiptera | DTUI | -4.10000000 | -79.35100000 | Ocaña-Mayorga et al. 2010 | Author | NA | I | I |
| *Rhodnius ecuadoriensis* | Vector | Hemiptera | DTUI | -4.10600000 | -79.34700000 | Ocaña-Mayorga et al. 2010 | Author | NA | I | I |
| *Rhodnius ecuadoriensis* | Vector | Hemiptera | DTUI | -4.09800000 | -79.34300000 | Ocaña-Mayorga et al. 2010 | Author | NA | I | I |
| *Rhodnius ecuadoriensis* | Vector | Hemiptera | DTUI | -4.10500000 | -79.34700000 | Ocaña-Mayorga et al. 2010 | Author | NA | I | I |
| *Rhodnius ecuadoriensis* | Vector | Hemiptera | DTUI | -4.10000000 | -79.35100000 | Ocaña-Mayorga et al. 2010 | Author | NA | I | I |
| *Rhodnius ecuadoriensis* | Vector | Hemiptera | DTUI | -4.09600000 | -79.34400000 | Ocaña-Mayorga et al. 2010 | Author | NA | I | I |
| *Rhodnius ecuadoriensis* | Vector | Hemiptera | DTUI | -4.09600000 | -79.34400000 | Ocaña-Mayorga et al. 2010 | Author | NA | I | I |
| *Rhodnius ecuadoriensis* | Vector | Hemiptera | DTUI | -4.09600000 | -79.34400000 | Ocaña-Mayorga et al. 2010 | Author | NA | I | I |
| *Rhodnius ecuadoriensis* | Vector | Hemiptera | DTUI | -4.10000000 | -79.35100000 | Ocaña-Mayorga et al. 2010 | Author | NA | I | I |
| *Rhodnius ecuadoriensis* | Vector | Hemiptera | DTUI | -4.09600000 | -79.34400000 | Ocaña-Mayorga et al. 2010 | Author | NA | I | I |
| *Rhodnius ecuadoriensis* | Vector | Hemiptera | DTUI | -4.09800000 | -79.34300000 | Ocaña-Mayorga et al. 2010 | Author | NA | I | I |
| *Rhodnius ecuadoriensis* | Vector | Hemiptera | DTUI | -4.08000000 | -79.82400000 | Ocaña-Mayorga et al. 2010 | Author | NA | I | I |
| *Rhodnius ecuadoriensis* | Vector | Hemiptera | DTUI | -4.07600000 | -79.80900000 | Ocaña-Mayorga et al. 2010 | Author | NA | I | I |
| *Rhodnius ecuadoriensis* | Vector | Hemiptera | DTUI | -4.10000000 | -79.35100000 | Ocaña-Mayorga et al. 2010 | Author | NA | I | I |
| *Rhodnius ecuadoriensis* | Vector | Hemiptera | DTUI | -4.07600000 | -79.80900000 | Ocaña-Mayorga et al. 2010 | Author | NA | I | I |
| *Rhodnius ecuadoriensis* | Vector | Hemiptera | DTUI | -4.07600000 | -79.80900000 | Ocaña-Mayorga et al. 2010 | Author | NA | I | I |
| *Rhodnius ecuadoriensis* | Vector | Hemiptera | DTUI | -4.07600000 | -79.80900000 | Ocaña-Mayorga et al. 2010 | Author | NA | I | I |
| *Rhodnius ecuadoriensis* | Vector | Hemiptera | DTUI | -4.07900000 | -79.80700000 | Ocaña-Mayorga et al. 2010 | Author | NA | I | I |
| *Rhodnius ecuadoriensis* | Vector | Hemiptera | DTUI | -4.07600000 | -79.80900000 | Ocaña-Mayorga et al. 2010 | Author | NA | I | I |
| *Rhodnius ecuadoriensis* | Vector | Hemiptera | DTUI | -4.07900000 | -79.80700000 | Ocaña-Mayorga et al. 2010 | Author | NA | I | I |
| *Rhodnius ecuadoriensis* | Vector | Hemiptera | DTUI | -4.07900000 | -79.80700000 | Ocaña-Mayorga et al. 2010 | Author | NA | I | I |
| *Rhodnius ecuadoriensis* | Vector | Hemiptera | DTUI | -4.07900000 | -79.80700000 | Ocaña-Mayorga et al. 2010 | Author | NA | I | I |
| *Rhodnius ecuadoriensis* | Vector | Hemiptera | DTUI | -4.07200000 | -79.83400000 | Ocaña-Mayorga et al. 2010 | Author | NA | I | I |
| *Rhodnius ecuadoriensis* | Vector | Hemiptera | DTUI | -4.07600000 | -79.80900000 | Ocaña-Mayorga et al. 2010 | Author | NA | I | I |
| *Rhodnius ecuadoriensis* | Vector | Hemiptera | DTUI | -4.24100000 | -79.58300000 | Ocaña-Mayorga et al. 2010 | Author | NA | I | I |
| *Rhodnius ecuadoriensis* | Vector | Hemiptera | DTUI | -4.19500000 | -80.03500000 | Ocaña-Mayorga et al. 2010 | Author | NA | I | I |
| *Rhodnius ecuadoriensis* | Vector | Hemiptera | DTUI | -4.21300000 | -80.10300000 | Ocaña-Mayorga et al. 2010 | Author | NA | I | I |
| *Rhodnius ecuadoriensis* | Vector | Hemiptera | DTUI | -4.04400000 | -79.36000000 | Ocaña-Mayorga et al. 2010 | Author | NA | I | I |
| *Rhodnius ecuadoriensis* | Vector | Hemiptera | DTUI | -4.36400000 | -79.41600000 | Ocaña-Mayorga et al. 2010 | Author | NA | I | I |
| *Rhodnius ecuadoriensis* | Vector | Hemiptera | DTUI | -4.35300000 | -79.43200000 | Ocaña-Mayorga et al. 2010 | Author | NA | I | I |
| *Rhodnius ecuadoriensis* | Vector | Hemiptera | DTUI | -4.35300000 | -79.43200000 | Ocaña-Mayorga et al. 2010 | Author | NA | I | I |
| *Rhodnius ecuadoriensis* | Vector | Hemiptera | DTUI | -4.35200000 | -79.43400000 | Ocaña-Mayorga et al. 2010 | Author | NA | I | I |
| *Rhodnius ecuadoriensis* | Vector | Hemiptera | DTUI | -4.35300000 | -79.43200000 | Ocaña-Mayorga et al. 2010 | Author | NA | I | I |
| *Rhodnius ecuadoriensis* | Vector | Hemiptera | DTUI | -4.34800000 | -79.43200000 | Ocaña-Mayorga et al. 2010 | Author | NA | I | I |
| *Rhodnius ecuadoriensis* | Vector | Hemiptera | DTUI | -4.07600000 | -79.80900000 | Ocaña-Mayorga et al. 2010 | Author | NA | I | I |
| *Rhodnius ecuadoriensis* | Vector | Hemiptera | DTUI | -4.07600000 | -79.80900000 | Ocaña-Mayorga et al. 2010 | Author | NA | I | I |
| *Rhodnius ecuadoriensis* | Vector | Hemiptera | DTUI | -4.07700000 | -79.80800000 | Ocaña-Mayorga et al. 2010 | Author | NA | I | I |
| *Rhodnius ecuadoriensis* | Vector | Hemiptera | DTUI | -4.07700000 | -79.80800000 | Ocaña-Mayorga et al. 2010 | Author | NA | I | I |
| *Rhodnius ecuadoriensis* | Vector | Hemiptera | DTUI | -4.07600000 | -79.80900000 | Ocaña-Mayorga et al. 2010 | Author | NA | I | I |
| *Rhodnius ecuadoriensis* | Vector | Hemiptera | DTUI | -4.07900000 | -79.82500000 | Ocaña-Mayorga et al. 2010 | Author | NA | I | I |
| *Rhodnius ecuadoriensis* | Vector | Hemiptera | DTUI | -4.12500000 | -79.55800000 | Ocaña-Mayorga et al. 2010 | Author | NA | I | I |
| *Rhodnius ecuadoriensis* | Vector | Hemiptera | DTUI | -4.12500000 | -79.55800000 | Ocaña-Mayorga et al. 2010 | Author | NA | I | I |
| *Rhodnius ecuadoriensis* | Vector | Hemiptera | DTUI | -4.08200000 | -79.80400000 | Ocaña-Mayorga et al. 2010 | Author | NA | I | I |
| *Rhodnius neglectus* | Vector | Hemiptera | DTUI | -20.193611 | -47.710556 | Cura et al. 2015 | Google earth | Locality | I | I |
| *Rhodnius neglectus* | Vector | Hemiptera | DTUI | -18.974722 | -49.460556 | Cura et al. 2015 | Google earth | Locality | I | I |
| *Rhodnius neglectus* | Vector | Hemiptera | DTUI | -9.754167 | -45.303889 | Cura et al. 2015 | Google earth | Locality | I | I |
| *Rhodnius neglectus* | Vector | Hemiptera | DTUI | -9.754167 | -45.303889 | Cura et al. 2015 | Google earth | Locality | I | I |
| *Rhodnius neglectus* | Vector | Hemiptera | DTUI | -9.754167 | -45.303889 | Cura et al. 2015 | Google earth | Locality | I | I |
| *Rhodnius neglectus* | Vector | Hemiptera | DTUI | -9.754167 | -45.303889 | Cura et al. 2015 | Google earth | Locality | I | I |
| *Rhodnius neglectus* | Vector | Hemiptera | DTUI | -9.754167 | -45.303889 | Cura et al. 2015 | Google earth | Locality | I | I |
| *Rhodnius neglectus* | Vector | Hemiptera | DTUI | -9.754167 | -45.303889 | Cura et al. 2015 | Google earth | Locality | I | I |
| *Rhodnius neglectus* | Vector | Hemiptera | DTUI | -9.754167 | -45.303889 | Cura et al. 2015 | Google earth | Locality | I | I |
| *Rhodnius neglectus* | Vector | Hemiptera | DTUI | -9.754167 | -45.303889 | Cura et al. 2015 | Google earth | Locality | I | I |
| *Rhodnius neglectus* | Vector | Hemiptera | DTUI | -10.248889 | -48.324444 | Cura et al. 2015 | Google earth | Locality | I | I |
| *Rhodnius neglectus* | Vector | Hemiptera | DTUI | -14.086389 | -46.370833 | Cura et al. 2015 | Google earth | Locality | I | I |
| *Rhodnius neglectus* | Vector | Hemiptera | DTUI | -18.447778 | -50.455278 | Cura et al. 2015 | Google earth | Locality | I | I |
| *Rhodnius neglectus* | Vector | Hemiptera | DTUI | -12.937222 | -51.825000 | Cura et al. 2015 | Google earth | Locality | I | I |
| *Rhodnius neglectus* | Vector | Hemiptera | DTUI | -12.937222 | -51.825000 | Cura et al. 2015 | Google earth | Locality | I | I |
| *Rhodnius neglectus* | Vector | Hemiptera | DTUI | -15.810833 | -48.065833 | Cura et al. 2015 | Google earth | Locality | I | I |
| *Rhodnius neglectus* | Vector | Hemiptera | DTUI | -15.810833 | -48.065833 | Cura et al. 2015 | Google earth | Locality | I | I |
| *Rhodnius pallescens* | Vector | Hemiptera | DTUI | 5.25000000 | -76.81600000 | Mejia-Jaramillo et al. 2009 | Google earth | Department/State | I | I |
| *Rhodnius pallescens* | Vector | Hemiptera | DTUI | 8.40000000 | -75.88300000 | Mejia-Jaramillo et al. 2009 | Google earth | Department/State | I | I |
| *Rhodnius pallescens* | Vector | Hemiptera | DTUI | 8.40000000 | -75.88300000 | Mejia-Jaramillo et al. 2009 | Google earth | Department/State | I | I |
| *Rhodnius pallescens* | Vector | Hemiptera | DTUI | 9.13000000 | -75.05000000 | Mejia-Jaramillo et al. 2009 | Google earth | Department/State | I | I |
| *Rhodnius pallescens* | Vector | Hemiptera | DTUI | 9.13000000 | -75.05000000 | Mejia-Jaramillo et al. 2009 | Google earth | Department/State | I | I |
| *Rhodnius pallescens* | Vector | Hemiptera | DTUI | 9.13000000 | -75.05000000 | Mejia-Jaramillo et al. 2009 | Google earth | Department/State | I | I |
| *Rhodnius pallescens* | Vector | Hemiptera | DTUI | 9.13000000 | -75.05000000 | Mejia-Jaramillo et al. 2009 | Google earth | Department/State | I | I |
| *Rhodnius pallescens* | Vector | Hemiptera | DTUI | 10.40000000 | -74.40000000 | Mejia-Jaramillo et al. 2009 | Google earth | Department/State | I | I |
| *Rhodnius pallescens* | Vector | Hemiptera | DTUI | 10.40000000 | -74.40000000 | Mejia-Jaramillo et al. 2009 | Google earth | Department/State | I | I |
| *Rhodnius pallescens* | Vector | Hemiptera | DTUI | 10.40000000 | -74.40000000 | Mejia-Jaramillo et al. 2009 | Google earth | Department/State | I | I |
| *Rhodnius pallescens* | Vector | Hemiptera | DTUI | 8.06700000 | -74.01670000 | Mejia-Jaramillo et al. 2009 | Google earth | Department/State | I | I |
| *Rhodnius pallescens* | Vector | Hemiptera | DTUI | 8.06700000 | -74.01670000 | Mejia-Jaramillo et al. 2009 | Google earth | Department/State | I | I |
| *Rhodnius pallescens* | Vector | Hemiptera | DTUI | 10.40000000 | -74.40000000 | Mejia-Jaramillo et al. 2009 | Google earth | Department/State | I | I |
| *Rhodnius pallescens* | Vector | Hemiptera | DTUI | 10.40000000 | -74.40000000 | Mejia-Jaramillo et al. 2009 | Google earth | Department/State | I | I |
| *Rhodnius pallescens* | Vector | Hemiptera | DTUI | 10.40000000 | -74.40000000 | Mejia-Jaramillo et al. 2009 | Google earth | Department/State | I | I |
| *Rhodnius pallescens* | Vector | Hemiptera | DTUI | 10.40000000 | -74.40000000 | Mejia-Jaramillo et al. 2009 | Google earth | Department/State | I | I |
| *Rhodnius pallescens* | Vector | Hemiptera | DTUI | 10.40000000 | -74.40000000 | Mejia-Jaramillo et al. 2009 | Google earth | Department/State | I | I |
| *Rhodnius pallescens* | Vector | Hemiptera | DTUI | 10.40000000 | -74.40000000 | Mejia-Jaramillo et al. 2009 | Google earth | Department/State | I | I |
| *Rhodnius pallescens* | Vector | Hemiptera | DTUI | 10.40000000 | -74.40000000 | Mejia-Jaramillo et al. 2009 | Google earth | Department/State | I | I |
| *Rhodnius pallescens* | Vector | Hemiptera | DTUI | 10.40000000 | -74.40000000 | Mejia-Jaramillo et al. 2009 | Google earth | Department/State | I | I |
| *Rhodnius pallescens* | Vector | Hemiptera | DTUI | 10.40000000 | -74.40000000 | Mejia-Jaramillo et al. 2009 | Google earth | Department/State | I | I |
| *Rhodnius pallescens* | Vector | Hemiptera | DTUI | 10.40000000 | -74.40000000 | Mejia-Jaramillo et al. 2009 | Google earth | Department/State | I | I |
| *Rhodnius pallescens* | Vector | Hemiptera | DTUI | 10.40000000 | -74.40000000 | Mejia-Jaramillo et al. 2009 | Google earth | Department/State | I | I |
| *Rhodnius pallescens* | Vector | Hemiptera | DTUI | 10.40000000 | -74.40000000 | Mejia-Jaramillo et al. 2009 | Google earth | Department/State | I | I |
| *Rhodnius pallescens* | Vector | Hemiptera | DTUI | 10.40000000 | -74.40000000 | Mejia-Jaramillo et al. 2009 | Google earth | Department/State | I | I |
| *Rhodnius pallescens* | Vector | Hemiptera | DTUI | 10.40000000 | -74.40000000 | Mejia-Jaramillo et al. 2009 | Google earth | Department/State | I | I |
| *Rhodnius pallescens* | Vector | Hemiptera | DTUI | 10.40000000 | -74.40000000 | Mejia-Jaramillo et al. 2009 | Google earth | Department/State | I | I |
| *Rhodnius pallescens* | Vector | Hemiptera | DTUI | 10.40000000 | -74.40000000 | Mejia-Jaramillo et al. 2009 | Google earth | Department/State | I | I |
| *Rhodnius pictipes* | Vector | Hemiptera | DTUI | 0.03300000000 | -51.07055556 | Marcili et al. 2009b | Google earth | Locality | I | I |
| *Rhodnius pictipes* | Vector | Hemiptera | DTUI | -3.13300000 | -58.43300000 | Marcili et al. 2009b | Google earth | Locality | I | I |
| *Rhodnius pictipes* | Vector | Hemiptera | DTUI | 4.27638889 | -52.64305556 | Cura et al. 2015 | Google earth | Locality | I | I |
| *Rhodnius pictipes* | Vector | Hemiptera | DTUI | 4.83166667 | -52.34527778 | Cura et al. 2015 | Google earth | Locality | I | I |
| *Rhodnius pictipes* | Vector | Hemiptera | DTUI | 4.72833333 | -52.32527778 | Cura et al. 2015 | Google earth | Locality | I | I |
| *Rhodnius pictipes* | Vector | Hemiptera | DTUI | 4.92222222 | -52.31333333 | Cura et al. 2015 | Google earth | Locality | I | I |
| *Rhodnius pictipes* | Vector | Hemiptera | DTUI | 4.88250000 | -52.28666667 | Cura et al. 2015 | Google earth | Locality | I | I |
| *Rhodnius pictipes* | Vector | Hemiptera | DTUI | 4.88250000 | -52.28666667 | Cura et al. 2015 | Google earth | Locality | I | I |
| *Rhodnius prolixus* | Vector | Hemiptera | DTUI | 9.08300000 | -69.08300000 | Carrasco et al. 2012 | Google earth | State | I | I |
| *Rhodnius prolixus* | Vector | Hemiptera | DTUI | 9.08300000 | -69.08300000 | Carrasco et al. 2012 | Google earth | State | I | I |
| *Rhodnius prolixus* | Vector | Hemiptera | DTUI | 9.08300000 | -69.08300000 | Carrasco et al. 2012 | Google earth | State | I | I |
| *Rhodnius prolixus* | Vector | Hemiptera | DTUI | 9.08300000 | -69.08300000 | Carrasco et al. 2012 | Google earth | State | I | I |
| *Rhodnius prolixus* | Vector | Hemiptera | DTUI | 9.08300000 | -69.08300000 | Carrasco et al. 2012 | Google earth | State | I | I |
| *Rhodnius prolixus* | Vector | Hemiptera | DTUI | 9.08300000 | -69.08300000 | Carrasco et al. 2012 | Google earth | State | I | I |
| *Rhodnius prolixus* | Vector | Hemiptera | DTUI | 9.08300000 | -69.08300000 | Carrasco et al. 2012 | Google earth | State | I | I |
| *Rhodnius prolixus* | Vector | Hemiptera | DTUI | 9.08300000 | -69.08300000 | Carrasco et al. 2012 | Google earth | State | I | I |
| *Rhodnius prolixus* | Vector | Hemiptera | DTUI | 9.08300000 | -69.08300000 | Carrasco et al. 2012 | Google earth | State | I | I |
| *Rhodnius prolixus* | Vector | Hemiptera | DTUI | 9.08300000 | -69.08300000 | Carrasco et al. 2012 | Google earth | State | I | I |
| *Rhodnius prolixus* | Vector | Hemiptera | DTUI | 9.08300000 | -69.08300000 | Carrasco et al. 2012 | Google earth | State | I | I |
| *Rhodnius prolixus* | Vector | Hemiptera | DTUI | 9.08300000 | -69.08300000 | Carrasco et al. 2012 | Google earth | State | I | I |
| *Rhodnius prolixus* | Vector | Hemiptera | DTUI | 9.08300000 | -69.08300000 | Carrasco et al. 2012 | Google earth | State | I | I |
| *Rhodnius prolixus* | Vector | Hemiptera | DTUI | 9.08300000 | -69.08300000 | Carrasco et al. 2012 | Google earth | State | I | I |
| *Rhodnius prolixus* | Vector | Hemiptera | DTUI | 9.08300000 | -69.08300000 | Carrasco et al. 2012 | Google earth | State | I | I |
| *Rhodnius prolixus* | Vector | Hemiptera | DTUI | 9.08300000 | -69.08300000 | Carrasco et al. 2012 | Google earth | State | I | I |
| *Rhodnius prolixus* | Vector | Hemiptera | DTUI | 9.08300000 | -69.08300000 | Carrasco et al. 2012 | Google earth | State | I | I |
| *Rhodnius prolixus* | Vector | Hemiptera | DTUI | 9.08300000 | -69.08300000 | Carrasco et al. 2012 | Google earth | State | I | I |
| *Rhodnius prolixus* | Vector | Hemiptera | DTUI | 9.08300000 | -69.08300000 | Carrasco et al. 2012 | Google earth | State | I | I |
| *Rhodnius prolixus* | Vector | Hemiptera | DTUI | 9.08300000 | -69.08300000 | Carrasco et al. 2012 | Google earth | State | I | I |
| *Rhodnius prolixus* | Vector | Hemiptera | DTUI | 9.08300000 | -69.08300000 | Carrasco et al. 2012 | Google earth | State | I | I |
| *Rhodnius prolixus* | Vector | Hemiptera | DTUI | 9.08300000 | -69.08300000 | Carrasco et al. 2012 | Google earth | State | I | I |
| *Rhodnius prolixus* | Vector | Hemiptera | DTUI | 9.08300000 | -69.08300000 | Carrasco et al. 2012 | Google earth | State | I | I |
| *Rhodnius prolixus* | Vector | Hemiptera | DTUI | 9.08300000 | -69.08300000 | Carrasco et al. 2012 | Google earth | State | I | I |
| *Rhodnius prolixus* | Vector | Hemiptera | DTUI | 9.08300000 | -69.08300000 | Carrasco et al. 2012 | Google earth | State | I | I |
| *Rhodnius prolixus* | Vector | Hemiptera | DTUI | 9.08300000 | -69.08300000 | Carrasco et al. 2012 | Google earth | State | I | I |
| *Rhodnius prolixus* | Vector | Hemiptera | DTUI | 9.08300000 | -69.08300000 | Carrasco et al. 2012 | Google earth | State | I | I |
| *Rhodnius prolixus* | Vector | Hemiptera | DTUI | 9.08300000 | -69.08300000 | Carrasco et al. 2012 | Google earth | State | I | I |
| *Rhodnius prolixus* | Vector | Hemiptera | DTUI | 9.08300000 | -69.08300000 | Carrasco et al. 2012 | Google earth | State | I | I |
| *Rhodnius prolixus* | Vector | Hemiptera | DTUI | 9.08300000 | -69.08300000 | Carrasco et al. 2012 | Google earth | State | I | I |
| *Rhodnius prolixus* | Vector | Hemiptera | DTUI | 9.08300000 | -69.08300000 | Carrasco et al. 2012 | Google earth | State | I | I |
| *Rhodnius prolixus* | Vector | Hemiptera | DTUI | 9.08300000 | -69.08300000 | Carrasco et al. 2012 | Google earth | State | I | I |
| *Rhodnius prolixus* | Vector | Hemiptera | DTUI | 9.08300000 | -69.08300000 | Carrasco et al. 2012 | Google earth | State | I | I |
| *Rhodnius prolixus* | Vector | Hemiptera | DTUI | 9.08300000 | -69.08300000 | Carrasco et al. 2012 | Google earth | State | I | I |
| *Rhodnius prolixus* | Vector | Hemiptera | DTUI | 9.08300000 | -69.08300000 | Carrasco et al. 2012 | Google earth | State | I | I |
| *Rhodnius prolixus* | Vector | Hemiptera | DTUI | 9.08300000 | -69.08300000 | Carrasco et al. 2012 | Google earth | State | I | I |
| *Rhodnius prolixus* | Vector | Hemiptera | DTUI | 9.08300000 | -69.08300000 | Carrasco et al. 2012 | Google earth | State | I | I |
| *Rhodnius prolixus* | Vector | Hemiptera | DTUI | 9.08300000 | -69.08300000 | Carrasco et al. 2012 | Google earth | State | I | I |
| *Rhodnius prolixus* | Vector | Hemiptera | DTUI | 9.08300000 | -69.08300000 | Carrasco et al. 2012 | Google earth | State | I | I |
| *Rhodnius prolixus* | Vector | Hemiptera | DTUI | 9.08300000 | -69.08300000 | Carrasco et al. 2012 | Google earth | State | I | I |
| *Rhodnius prolixus* | Vector | Hemiptera | DTUI | 9.08300000 | -69.08300000 | Carrasco et al. 2012 | Google earth | State | I | I |
| *Rhodnius prolixus* | Vector | Hemiptera | DTUI | 9.08300000 | -69.08300000 | Carrasco et al. 2012 | Google earth | State | I | I |
| *Rhodnius prolixus* | Vector | Hemiptera | DTUI | 9.08300000 | -69.08300000 | Carrasco et al. 2012 | Google earth | State | I | I |
| *Rhodnius prolixus* | Vector | Hemiptera | DTUI | 9.08300000 | -69.08300000 | Carrasco et al. 2012 | Google earth | State | I | I |
| *Rhodnius prolixus* | Vector | Hemiptera | DTUI | 9.08300000 | -69.08300000 | Carrasco et al. 2012 | Google earth | State | I | I |
| *Rhodnius prolixus* | Vector | Hemiptera | DTUI | 9.08300000 | -69.08300000 | Carrasco et al. 2012 | Google earth | State | I | I |
| *Rhodnius prolixus* | Vector | Hemiptera | DTUI | 9.08300000 | -69.08300000 | Carrasco et al. 2012 | Google earth | State | I | I |
| *Rhodnius prolixus* | Vector | Hemiptera | DTUI | 9.08300000 | -69.08300000 | Carrasco et al. 2012 | Google earth | State | I | I |
| *Rhodnius prolixus* | Vector | Hemiptera | DTUI | 9.08300000 | -69.08300000 | Carrasco et al. 2012 | Google earth | State | I | I |
| *Rhodnius prolixus* | Vector | Hemiptera | DTUI | 9.08300000 | -69.08300000 | Carrasco et al. 2012 | Google earth | State | I | I |
| *Rhodnius prolixus* | Vector | Hemiptera | DTUI | 9.08300000 | -69.08300000 | Carrasco et al. 2012 | Google earth | State | I | I |
| *Rhodnius prolixus* | Vector | Hemiptera | DTUI | 9.08300000 | -69.08300000 | Carrasco et al. 2012 | Google earth | State | I | I |
| *Rhodnius prolixus* | Vector | Hemiptera | DTUI | 9.08300000 | -69.08300000 | Carrasco et al. 2012 | Google earth | State | I | I |
| *Rhodnius prolixus* | Vector | Hemiptera | DTUI | 9.08300000 | -69.08300000 | Carrasco et al. 2012 | Google earth | State | I | I |
| *Rhodnius prolixus* | Vector | Hemiptera | DTUI | 9.08300000 | -69.08300000 | Carrasco et al. 2012 | Google earth | State | I | I |
| *Rhodnius prolixus* | Vector | Hemiptera | DTUI | 9.08300000 | -69.08300000 | Carrasco et al. 2012 | Google earth | State | I | I |
| *Rhodnius prolixus* | Vector | Hemiptera | DTUI | 9.08300000 | -69.08300000 | Carrasco et al. 2012 | Google earth | State | I | I |
| *Rhodnius prolixus* | Vector | Hemiptera | DTUI | 9.08300000 | -69.08300000 | Carrasco et al. 2012 | Google earth | State | I | I |
| *Rhodnius prolixus* | Vector | Hemiptera | DTUI | 9.08300000 | -69.08300000 | Carrasco et al. 2012 | Google earth | State | I | I |
| *Rhodnius prolixus* | Vector | Hemiptera | DTUI | 9.08300000 | -69.08300000 | Carrasco et al. 2012 | Google earth | State | I | I |
| *Rhodnius prolixus* | Vector | Hemiptera | DTUI | 9.08300000 | -69.08300000 | Carrasco et al. 2012 | Google earth | State | I | I |
| *Rhodnius prolixus* | Vector | Hemiptera | DTUI | 9.08300000 | -69.08300000 | Carrasco et al. 2012 | Google earth | State | I | I |
| *Rhodnius prolixus* | Vector | Hemiptera | DTUI | 9.08300000 | -69.08300000 | Carrasco et al. 2012 | Google earth | State | I | I |
| *Rhodnius prolixus* | Vector | Hemiptera | DTUI | 9.08300000 | -69.08300000 | Carrasco et al. 2012 | Google earth | State | I | I |
| *Rhodnius prolixus* | Vector | Hemiptera | DTUI | 9.08300000 | -69.08300000 | Carrasco et al. 2012 | Google earth | State | I | I |
| *Rhodnius prolixus* | Vector | Hemiptera | DTUI | 9.08300000 | -69.08300000 | Carrasco et al. 2012 | Google earth | State | I | I |
| *Rhodnius prolixus* | Vector | Hemiptera | DTUI | 9.08300000 | -69.08300000 | Carrasco et al. 2012 | Google earth | State | I | I |
| *Rhodnius prolixus* | Vector | Hemiptera | DTUI | 9.08300000 | -69.08300000 | Carrasco et al. 2012 | Google earth | State | I | I |
| *Rhodnius prolixus* | Vector | Hemiptera | DTUI | 9.08300000 | -69.08300000 | Carrasco et al. 2012 | Google earth | State | I | I |
| *Rhodnius prolixus* | Vector | Hemiptera | DTUI | 9.08300000 | -69.08300000 | Carrasco et al. 2012 | Google earth | State | I | I |
| *Rhodnius prolixus* | Vector | Hemiptera | DTUI | 9.08300000 | -69.08300000 | Carrasco et al. 2012 | Google earth | State | I | I |
| *Rhodnius prolixus* | Vector | Hemiptera | DTUI | 9.08300000 | -69.08300000 | Carrasco et al. 2012 | Google earth | State | I | I |
| *Rhodnius prolixus* | Vector | Hemiptera | DTUI | 9.08300000 | -69.08300000 | Carrasco et al. 2012 | Google earth | State | I | I |
| *Rhodnius prolixus* | Vector | Hemiptera | DTUI | 9.08300000 | -69.08300000 | Carrasco et al. 2012 | Google earth | State | I | I |
| *Rhodnius prolixus* | Vector | Hemiptera | DTUI | 9.08300000 | -69.08300000 | Carrasco et al. 2012 | Google earth | State | I | I |
| *Rhodnius prolixus* | Vector | Hemiptera | DTUI | 9.08300000 | -69.08300000 | Carrasco et al. 2012 | Google earth | State | I | I |
| *Rhodnius prolixus* | Vector | Hemiptera | DTUI | 9.08300000 | -69.08300000 | Carrasco et al. 2012 | Google earth | State | I | I |
| *Rhodnius prolixus* | Vector | Hemiptera | DTUI | 9.08300000 | -69.08300000 | Carrasco et al. 2012 | Google earth | State | I | I |
| *Rhodnius prolixus* | Vector | Hemiptera | DTUI | 9.08300000 | -69.08300000 | Carrasco et al. 2012 | Google earth | State | I | I |
| *Rhodnius prolixus* | Vector | Hemiptera | DTUI | 9.08300000 | -69.08300000 | Carrasco et al. 2012 | Google earth | State | I | I |
| *Rhodnius prolixus* | Vector | Hemiptera | DTUIV | 9.08300000 | -69.08300000 | Carrasco et al. 2012 | Google earth | State | II | III |
| *Rhodnius prolixus* | Vector | Hemiptera | DTUIV | 9.08300000 | -69.08300000 | Carrasco et al. 2012 | Google earth | State | II | III |
| *Rhodnius prolixus* | Vector | Hemiptera | DTUIV | 9.08300000 | -69.08300000 | Carrasco et al. 2012 | Google earth | State | II | III |
| *Rhodnius prolixus* | Vector | Hemiptera | DTUI | 8.30000000 | -70.05000000 | Carrasco et al. 2012 | Google earth | State | I | I |
| *Rhodnius prolixus* | Vector | Hemiptera | DTUI | 8.30000000 | -70.05000000 | Carrasco et al. 2012 | Google earth | State | I | I |
| *Rhodnius prolixus* | Vector | Hemiptera | DTUI | 8.30000000 | -70.05000000 | Carrasco et al. 2012 | Google earth | State | I | I |
| *Rhodnius prolixus* | Vector | Hemiptera | DTUI | 8.30000000 | -70.05000000 | Carrasco et al. 2012 | Google earth | State | I | I |
| *Rhodnius prolixus* | Vector | Hemiptera | DTUI | 8.30000000 | -70.05000000 | Carrasco et al. 2012 | Google earth | State | I | I |
| *Rhodnius prolixus* | Vector | Hemiptera | DTUI | 8.30000000 | -70.05000000 | Carrasco et al. 2012 | Google earth | State | I | I |
| *Rhodnius prolixus* | Vector | Hemiptera | DTUI | 8.30000000 | -70.05000000 | Carrasco et al. 2012 | Google earth | State | I | I |
| *Rhodnius prolixus* | Vector | Hemiptera | DTUI | 8.30000000 | -70.05000000 | Carrasco et al. 2012 | Google earth | State | I | I |
| *Rhodnius prolixus* | Vector | Hemiptera | DTUI | 8.30000000 | -70.05000000 | Carrasco et al. 2012 | Google earth | State | I | I |
| *Rhodnius prolixus* | Vector | Hemiptera | DTUI | 8.30000000 | -70.05000000 | Carrasco et al. 2012 | Google earth | State | I | I |
| *Rhodnius prolixus* | Vector | Hemiptera | DTUI | 8.30000000 | -70.05000000 | Carrasco et al. 2012 | Google earth | State | I | I |
| *Rhodnius prolixus* | Vector | Hemiptera | DTUI | 8.30000000 | -70.05000000 | Carrasco et al. 2012 | Google earth | State | I | I |
| *Rhodnius prolixus* | Vector | Hemiptera | DTUI | 8.30000000 | -70.05000000 | Carrasco et al. 2012 | Google earth | State | I | I |
| *Rhodnius prolixus* | Vector | Hemiptera | DTUI | 8.30000000 | -70.05000000 | Carrasco et al. 2012 | Google earth | State | I | I |
| *Rhodnius prolixus* | Vector | Hemiptera | DTUI | 8.30000000 | -70.05000000 | Carrasco et al. 2012 | Google earth | State | I | I |
| *Rhodnius prolixus* | Vector | Hemiptera | DTUI | 8.30000000 | -70.05000000 | Carrasco et al. 2012 | Google earth | State | I | I |
| *Rhodnius prolixus* | Vector | Hemiptera | DTUI | 8.30000000 | -70.05000000 | Carrasco et al. 2012 | Google earth | State | I | I |
| *Rhodnius prolixus* | Vector | Hemiptera | DTUI | 8.30000000 | -70.05000000 | Carrasco et al. 2012 | Google earth | State | I | I |
| *Rhodnius prolixus* | Vector | Hemiptera | DTUI | 8.30000000 | -70.05000000 | Carrasco et al. 2012 | Google earth | State | I | I |
| *Rhodnius prolixus* | Vector | Hemiptera | DTUI | 8.30000000 | -70.05000000 | Carrasco et al. 2012 | Google earth | State | I | I |
| *Rhodnius prolixus* | Vector | Hemiptera | DTUI | 8.30000000 | -70.05000000 | Carrasco et al. 2012 | Google earth | State | I | I |
| *Rhodnius prolixus* | Vector | Hemiptera | DTUI | 8.30000000 | -70.05000000 | Carrasco et al. 2012 | Google earth | State | I | I |
| *Rhodnius prolixus* | Vector | Hemiptera | DTUI | 8.30000000 | -70.05000000 | Carrasco et al. 2012 | Google earth | State | I | I |
| *Rhodnius prolixus* | Vector | Hemiptera | DTUI | 8.30000000 | -70.05000000 | Carrasco et al. 2012 | Google earth | State | I | I |
| *Rhodnius prolixus* | Vector | Hemiptera | DTUI | 8.30000000 | -70.05000000 | Carrasco et al. 2012 | Google earth | State | I | I |
| *Rhodnius prolixus* | Vector | Hemiptera | DTUI | 8.30000000 | -70.05000000 | Carrasco et al. 2012 | Google earth | State | I | I |
| *Rhodnius prolixus* | Vector | Hemiptera | DTUI | 8.30000000 | -70.05000000 | Carrasco et al. 2012 | Google earth | State | I | I |
| *Rhodnius prolixus* | Vector | Hemiptera | DTUI | 8.30000000 | -70.05000000 | Carrasco et al. 2012 | Google earth | State | I | I |
| *Rhodnius prolixus* | Vector | Hemiptera | DTUI | 8.30000000 | -70.05000000 | Carrasco et al. 2012 | Google earth | State | I | I |
| *Rhodnius prolixus* | Vector | Hemiptera | DTUI | 8.30000000 | -70.05000000 | Carrasco et al. 2012 | Google earth | State | I | I |
| *Rhodnius prolixus* | Vector | Hemiptera | DTUI | 8.30000000 | -70.05000000 | Carrasco et al. 2012 | Google earth | State | I | I |
| *Rhodnius prolixus* | Vector | Hemiptera | DTUI | 8.30000000 | -70.05000000 | Carrasco et al. 2012 | Google earth | State | I | I |
| *Rhodnius prolixus* | Vector | Hemiptera | DTUI | 8.30000000 | -70.05000000 | Carrasco et al. 2012 | Google earth | State | I | I |
| *Rhodnius prolixus* | Vector | Hemiptera | DTUI | 8.30000000 | -70.05000000 | Carrasco et al. 2012 | Google earth | State | I | I |
| *Rhodnius prolixus* | Vector | Hemiptera | DTUI | 8.30000000 | -70.05000000 | Carrasco et al. 2012 | Google earth | State | I | I |
| *Rhodnius prolixus* | Vector | Hemiptera | DTUI | 8.30000000 | -70.05000000 | Carrasco et al. 2012 | Google earth | State | I | I |
| *Rhodnius prolixus* | Vector | Hemiptera | DTUI | 8.30000000 | -70.05000000 | Carrasco et al. 2012 | Google earth | State | I | I |
| *Rhodnius prolixus* | Vector | Hemiptera | DTUI | 10.11670000 | -68.03400000 | Carrasco et al. 2012 | Google earth | State | I | I |
| *Rhodnius prolixus* | Vector | Hemiptera | DTUI | 10.11670000 | -68.03400000 | Carrasco et al. 2012 | Google earth | State | I | I |
| *Rhodnius prolixus* | Vector | Hemiptera | DTUI | 10.11670000 | -68.03400000 | Carrasco et al. 2012 | Google earth | State | I | I |
| *Rhodnius prolixus* | Vector | Hemiptera | DTUI | 10.11670000 | -68.03400000 | Carrasco et al. 2012 | Google earth | State | I | I |
| *Rhodnius prolixus* | Vector | Hemiptera | DTUI | 10.11670000 | -68.03400000 | Carrasco et al. 2012 | Google earth | State | I | I |
| *Rhodnius prolixus* | Vector | Hemiptera | DTUI | 10.11670000 | -68.03400000 | Carrasco et al. 2012 | Google earth | State | I | I |
| *Rhodnius prolixus* | Vector | Hemiptera | DTUI | 10.11670000 | -68.03400000 | Carrasco et al. 2012 | Google earth | State | I | I |
| *Rhodnius prolixus* | Vector | Hemiptera | DTUI | 9.36700000 | -68.03400000 | Carrasco et al. 2012 | Google earth | State | I | I |
| *Rhodnius prolixus* | Vector | Hemiptera | DTUI | 9.36700000 | -68.03400000 | Carrasco et al. 2012 | Google earth | State | I | I |
| *Rhodnius prolixus* | Vector | Hemiptera | DTUI | 9.36700000 | -68.03400000 | Carrasco et al. 2012 | Google earth | State | I | I |
| *Rhodnius prolixus* | Vector | Hemiptera | DTUI | 9.36700000 | -68.03400000 | Carrasco et al. 2012 | Google earth | State | I | I |
| *Rhodnius prolixus* | Vector | Hemiptera | DTUI | 9.36700000 | -68.03400000 | Carrasco et al. 2012 | Google earth | State | I | I |
| *Rhodnius prolixus* | Vector | Hemiptera | DTUI | 10.05000000 | -66.95000000 | Carrasco et al. 2012 | Google earth | State | I | I |
| *Rhodnius prolixus* | Vector | Hemiptera | DTUI | 10.15000000 | -69.85000000 | Carrasco et al. 2012 | Google earth | State | I | I |
| *Rhodnius prolixus* | Vector | Hemiptera | DTUI | 10.15000000 | -69.85000000 | Carrasco et al. 2012 | Google earth | State | I | I |
| *Rhodnius prolixus* | Vector | Hemiptera | DTUI | 10.15000000 | -69.85000000 | Carrasco et al. 2012 | Google earth | State | I | I |
| *Rhodnius prolixus* | Vector | Hemiptera | DTUI | 10.15000000 | -69.85000000 | Carrasco et al. 2012 | Google earth | State | I | I |
| *Rhodnius prolixus* | Vector | Hemiptera | DTUI | 10.15000000 | -69.85000000 | Carrasco et al. 2012 | Google earth | State | I | I |
| *Rhodnius prolixus* | Vector | Hemiptera | DTUI | 9.36700000 | -70.41670000 | Carrasco et al. 2012 | Google earth | State | I | I |
| *Rhodnius prolixus* | Vector | Hemiptera | DTUI | 9.36700000 | -70.41670000 | Carrasco et al. 2012 | Google earth | State | I | I |
| *Rhodnius prolixus* | Vector | Hemiptera | DTUI | 9.36700000 | -70.41670000 | Carrasco et al. 2012 | Google earth | State | I | I |
| *Rhodnius prolixus* | Vector | Hemiptera | DTUI | 15.06700000 | -89.43300000 | Higo et al. 2004 | Google earth | Department/State | I | I |
| *Rhodnius prolixus* | Vector | Hemiptera | DTUI | 15.06700000 | -89.43300000 | Higo et al. 2004 | Google earth | Department/State | I | I |
| *Rhodnius prolixus* | Vector | Hemiptera | DTUI | 15.06700000 | -89.43300000 | Higo et al. 2004 | Google earth | Department/State | I | I |
| *Rhodnius prolixus* | Vector | Hemiptera | DTUI | 15.06700000 | -89.43300000 | Higo et al. 2004 | Google earth | Department/State | I | I |
| *Rhodnius prolixus* | Vector | Hemiptera | DTUI | 9.01000000 | -69.29000000 | Llewellyn et al. 2009b | Author | NA | I | I |
| *Rhodnius prolixus* | Vector | Hemiptera | DTUI | 9.01000000 | -64.34000000 | Llewellyn et al. 2009b | Author | NA | I | I |
| *Rhodnius prolixus* | Vector | Hemiptera | DTUI | 10.19000000 | -68.00000000 | Llewellyn et al. 2009b | Author | NA | I | I |
| *Rhodnius prolixus* | Vector | Hemiptera | DTUI | 7.50000000 | -71.23000000 | Llewellyn et al. 2009b | Author | NA | I | I |
| *Rhodnius prolixus* | Vector | Hemiptera | DTUI | 8.48000000 | -70.73000000 | Llewellyn et al. 2009b | Author | NA | I | I |
| *Rhodnius prolixus* | Vector | Hemiptera | DTUI | 8.48000000 | -70.73000000 | Llewellyn et al. 2009b | Author | NA | I | I |
| *Rhodnius prolixus* | Vector | Hemiptera | DTUI | 8.48000000 | -70.73000000 | Llewellyn et al. 2009b | Author | NA | I | I |
| *Rhodnius prolixus* | Vector | Hemiptera | DTUI | 9.82800000 | -68.43000000 | Llewellyn et al. 2009b | Author | NA | I | I |
| *Rhodnius prolixus* | Vector | Hemiptera | DTUI | 8.34000000 | -68.68000000 | Llewellyn et al. 2009b | Author | NA | I | I |
| *Rhodnius prolixus* | Vector | Hemiptera | DTUI | 10.04000000 | -64.32000000 | Llewellyn et al. 2009b | Author | NA | I | I |
| *Rhodnius prolixus* | Vector | Hemiptera | DTUI | 10.95000000 | -73.46700000 | Rodriguez et al. 2009 | Google earth | Area/Unspecified communities | I | I |
| *Rhodnius prolixus* | Vector | Hemiptera | DTUI | 10.95000000 | -73.46700000 | Rodriguez et al. 2009 | Google earth | Area/Unspecified communities | I | I |
| *Rhodnius prolixus* | Vector | Hemiptera | DTUI | 10.95000000 | -73.46700000 | Rodriguez et al. 2009 | Google earth | Area/Unspecified communities | I | I |
| *Rhodnius prolixus* | Vector | Hemiptera | DTUI | 10.95000000 | -73.46700000 | Rodriguez et al. 2009 | Google earth | Area/Unspecified communities | I | I |
| *Rhodnius prolixus* | Vector | Hemiptera | DTUI | 10.95000000 | -73.46700000 | Rodriguez et al. 2009 | Google earth | Area/Unspecified communities | I | I |
| *Rhodnius prolixus* | Vector | Hemiptera | DTUI | 10.95000000 | -73.46700000 | Rodriguez et al. 2009 | Google earth | Area/Unspecified communities | I | I |
| *Rhodnius prolixus* | Vector | Hemiptera | DTUI | 10.95000000 | -73.46700000 | Rodriguez et al. 2009 | Google earth | Area/Unspecified communities | I | I |
| *Rhodnius prolixus* | Vector | Hemiptera | DTUI | 10.95000000 | -73.46700000 | Rodriguez et al. 2009 | Google earth | Area/Unspecified communities | I | I |
| *Rhodnius prolixus* | Vector | Hemiptera | DTUI | 10.95000000 | -73.46700000 | Rodriguez et al. 2009 | Google earth | Area/Unspecified communities | I | I |
| *Rhodnius prolixus* | Vector | Hemiptera | DTUI | 10.95000000 | -73.46700000 | Rodriguez et al. 2009 | Google earth | Area/Unspecified communities | I | I |
| *Rhodnius prolixus* | Vector | Hemiptera | DTUI | 10.95000000 | -73.46700000 | Rodriguez et al. 2009 | Google earth | Area/Unspecified communities | I | I |
| *Rhodnius prolixus* | Vector | Hemiptera | DTUI | 10.95000000 | -73.46700000 | Rodriguez et al. 2009 | Google earth | Area/Unspecified communities | I | I |
| *Rhodnius prolixus* | Vector | Hemiptera | DTUI | 10.95000000 | -73.46700000 | Rodriguez et al. 2009 | Google earth | Area/Unspecified communities | I | I |
| *Rhodnius prolixus* | Vector | Hemiptera | DTUI | 10.95000000 | -73.46700000 | Rodriguez et al. 2009 | Google earth | Area/Unspecified communities | I | I |
| *Rhodnius prolixus* | Vector | Hemiptera | DTUI | 10.95000000 | -73.46700000 | Rodriguez et al. 2009 | Google earth | Area/Unspecified communities | I | I |
| *Rhodnius prolixus* | Vector | Hemiptera | DTUI | 10.95000000 | -73.46700000 | Rodriguez et al. 2009 | Google earth | Area/Unspecified communities | I | I |
| *Rhodnius prolixus* | Vector | Hemiptera | DTUI | 10.95000000 | -73.46700000 | Rodriguez et al. 2009 | Google earth | Area/Unspecified communities | I | I |
| *Rhodnius prolixus* | Vector | Hemiptera | DTUI | 10.95000000 | -73.46700000 | Rodriguez et al. 2009 | Google earth | Area/Unspecified communities | I | I |
| *Rhodnius prolixus* | Vector | Hemiptera | DTUI | 10.95000000 | -73.46700000 | Rodriguez et al. 2009 | Google earth | Area/Unspecified communities | I | I |
| *Rhodnius prolixus* | Vector | Hemiptera | DTUI | 10.95000000 | -73.46700000 | Rodriguez et al. 2009 | Google earth | Area/Unspecified communities | I | I |
| *Rhodnius prolixus* | Vector | Hemiptera | DTUI | 10.95000000 | -73.46700000 | Rodriguez et al. 2009 | Google earth | Area/Unspecified communities | I | I |
| *Rhodnius prolixus* | Vector | Hemiptera | DTUI | 10.95000000 | -73.46700000 | Rodriguez et al. 2009 | Google earth | Area/Unspecified communities | I | I |
| *Rhodnius prolixus* | Vector | Hemiptera | DTUI | 10.95000000 | -73.46700000 | Rodriguez et al. 2009 | Google earth | Area/Unspecified communities | I | I |
| *Rhodnius prolixus* | Vector | Hemiptera | DTUI | 4.15000000 | -71.20000000 | Ramirez et al. 2013 | Author | NA | I | I |
| *Rhodnius prolixus* | Vector | Hemiptera | DTUI | 5.10000000 | -71.60000000 | Ramirez et al. 2013 | Author | NA | I | I |
| *Rhodnius prolixus* | Vector | Hemiptera | DTUI | 4.15000000 | -71.20000000 | Ramirez et al. 2013 | Author | NA | I | I |
| *Rhodnius prolixus* | Vector | Hemiptera | DTUI | 5.10000000 | -71.60000000 | Ramirez et al. 2013 | Author | NA | I | I |
| *Rhodnius prolixus* | Vector | Hemiptera | DTUI | 5.10000000 | -71.60000000 | Ramirez et al. 2013 | Author | NA | I | I |
| *Rhodnius prolixus* | Vector | Hemiptera | DTUI | 4.96000000 | -73.63000000 | Ramirez et al. 2013 | Author | NA | I | I |
| *Rhodnius prolixus* | Vector | Hemiptera | DTUI | 4.96000000 | -73.63000000 | Ramirez et al. 2013 | Author | NA | I | I |
| *Rhodnius prolixus* | Vector | Hemiptera | DTUI | 5.75000000 | -71.56700000 | Mejia-Jaramillo et al. 2009 | Google earth | Department/State | I | I |
| *Rhodnius prolixus* | Vector | Hemiptera | DTUI | 5.75000000 | -71.56700000 | Mejia-Jaramillo et al. 2009 | Google earth | Department/State | I | I |
| *Rhodnius prolixus* | Vector | Hemiptera | DTUI | 5.75000000 | -71.56700000 | Mejia-Jaramillo et al. 2009 | Google earth | Department/State | I | I |
| *Rhodnius prolixus* | Vector | Hemiptera | DTUI | 8.58300000 | -63.95000000 | Carrasco et al. 2012 | Google earth | State | I | I |
| *Rhodnius prolixus* | Vector | Hemiptera | DTUI | 8.58300000 | -63.95000000 | Carrasco et al. 2012 | Google earth | State | I | I |
| *Rhodnius prolixus* | Vector | Hemiptera | DTUI | 8.58300000 | -63.95000000 | Carrasco et al. 2012 | Google earth | State | I | I |
| *Rhodnius prolixus* | Vector | Hemiptera | DTUI | 8.58300000 | -63.95000000 | Carrasco et al. 2012 | Google earth | State | I | I |
| *Rhodnius prolixus* | Vector | Hemiptera | DTUI | 8.58300000 | -63.95000000 | Carrasco et al. 2012 | Google earth | State | I | I |
| *Rhodnius prolixus* | Vector | Hemiptera | DTUI | 8.58300000 | -63.95000000 | Carrasco et al. 2012 | Google earth | State | I | I |
| *Rhodnius prolixus* | Vector | Hemiptera | DTUI | 8.58300000 | -63.95000000 | Carrasco et al. 2012 | Google earth | State | I | I |
| *Rhodnius prolixus* | Vector | Hemiptera | DTUI | 8.58300000 | -63.95000000 | Carrasco et al. 2012 | Google earth | State | I | I |
| *Rhodnius prolixus* | Vector | Hemiptera | DTUI | 8.58300000 | -63.95000000 | Carrasco et al. 2012 | Google earth | State | I | I |
| *Rhodnius prolixus* | Vector | Hemiptera | DTUI | 8.58300000 | -63.95000000 | Carrasco et al. 2012 | Google earth | State | I | I |
| *Rhodnius prolixus* | Vector | Hemiptera | DTUI | 8.58300000 | -63.95000000 | Carrasco et al. 2012 | Google earth | State | I | I |
| *Rhodnius prolixus* | Vector | Hemiptera | DTUI | 8.58300000 | -63.95000000 | Carrasco et al. 2012 | Google earth | State | I | I |
| *Rhodnius prolixus* | Vector | Hemiptera | DTUI | 8.58300000 | -63.95000000 | Carrasco et al. 2012 | Google earth | State | I | I |
| *Rhodnius prolixus* | Vector | Hemiptera | DTUI | 8.58300000 | -63.95000000 | Carrasco et al. 2012 | Google earth | State | I | I |
| *Rhodnius prolixus* | Vector | Hemiptera | DTUI | 8.58300000 | -63.95000000 | Carrasco et al. 2012 | Google earth | State | I | I |
| *Rhodnius prolixus* | Vector | Hemiptera | DTUI | 8.58300000 | -63.95000000 | Carrasco et al. 2012 | Google earth | State | I | I |
| *Rhodnius prolixus* | Vector | Hemiptera | DTUI | 8.58300000 | -63.95000000 | Carrasco et al. 2012 | Google earth | State | I | I |
| *Rhodnius prolixus* | Vector | Hemiptera | DTUI | 8.58300000 | -63.95000000 | Carrasco et al. 2012 | Google earth | State | I | I |
| *Rhodnius prolixus* | Vector | Hemiptera | DTUI | 8.58300000 | -63.95000000 | Carrasco et al. 2012 | Google earth | State | I | I |
| *Rhodnius prolixus* | Vector | Hemiptera | DTUI | 8.58300000 | -63.95000000 | Carrasco et al. 2012 | Google earth | State | I | I |
| *Rhodnius prolixus* | Vector | Hemiptera | DTUI | 8.58300000 | -63.95000000 | Carrasco et al. 2012 | Google earth | State | I | I |
| *Rhodnius prolixus* | Vector | Hemiptera | DTUI | 8.58300000 | -63.95000000 | Carrasco et al. 2012 | Google earth | State | I | I |
| *Rhodnius prolixus* | Vector | Hemiptera | DTUI | 8.58300000 | -63.95000000 | Carrasco et al. 2012 | Google earth | State | I | I |
| *Rhodnius prolixus* | Vector | Hemiptera | DTUI | 8.58300000 | -63.95000000 | Carrasco et al. 2012 | Google earth | State | I | I |
| *Rhodnius prolixus* | Vector | Hemiptera | DTUI | 8.58300000 | -63.95000000 | Carrasco et al. 2012 | Google earth | State | I | I |
| *Rhodnius prolixus* | Vector | Hemiptera | DTUI | 8.58300000 | -63.95000000 | Carrasco et al. 2012 | Google earth | State | I | I |
| *Rhodnius prolixus* | Vector | Hemiptera | DTUI | 8.58300000 | -63.95000000 | Carrasco et al. 2012 | Google earth | State | I | I |
| *Rhodnius prolixus* | Vector | Hemiptera | DTUI | 8.58300000 | -63.95000000 | Carrasco et al. 2012 | Google earth | State | I | I |
| *Rhodnius prolixus* | Vector | Hemiptera | DTUI | 8.58300000 | -63.95000000 | Carrasco et al. 2012 | Google earth | State | I | I |
| *Rhodnius prolixus* | Vector | Hemiptera | DTUI | 8.58300000 | -63.95000000 | Carrasco et al. 2012 | Google earth | State | I | I |
| *Rhodnius prolixus* | Vector | Hemiptera | DTUI | 8.58300000 | -63.95000000 | Carrasco et al. 2012 | Google earth | State | I | I |
| *Rhodnius prolixus* | Vector | Hemiptera | DTUI | 8.58300000 | -63.95000000 | Carrasco et al. 2012 | Google earth | State | I | I |
| *Rhodnius prolixus* | Vector | Hemiptera | DTUI | 8.58300000 | -63.95000000 | Carrasco et al. 2012 | Google earth | State | I | I |
| *Rhodnius prolixus* | Vector | Hemiptera | DTUI | 8.58300000 | -63.95000000 | Carrasco et al. 2012 | Google earth | State | I | I |
| *Rhodnius prolixus* | Vector | Hemiptera | DTUI | 8.58300000 | -63.95000000 | Carrasco et al. 2012 | Google earth | State | I | I |
| *Rhodnius robustus* | Vector | Hemiptera | DTUI | -10.20000000 | -63.03000000 | Marcili et al. 2009b | Google earth | Locality | I | I |
| *Rhodnius robustus* | Vector | Hemiptera | DTUI | -10.20000000 | -63.03000000 | Marcili et al. 2009b | Google earth | Locality | I | I |
| *Rhodnius robustus* | Vector | Hemiptera | DTUI | -0.83000000 | -48.90000000 | Marcili et al. 2009b | Google earth | Locality | I | I |
| *Rhodnius robustus* | Vector | Hemiptera | DTUI | -0.03300000 | -51.01600000 | Marcili et al. 2009b | Google earth | Locality | I | I |
| *Rhodnius robustus* | Vector | Hemiptera | DTUI | 3.16583333 | -52.34111111 | Cura et al. 2015 | Google earth | Locality | I | I |
| *Rhodnius robustus* | Vector | Hemiptera | DTUIV | -10.20000000 | -63.03000000 | Marcili et al. 2009b | Google earth | Locality | II | III |
| *Rhodnius robustus* | Vector | Hemiptera | DTUIV | -10.20000000 | -63.03000000 | Marcili et al. 2009b | Google earth | Locality | II | III |
| *Rhodnius robustus* | Vector | Hemiptera | DTUIV | -10.20000000 | -63.03000000 | Marcili et al. 2009b | Google earth | Locality | II | III |
| *Rhodnius robustus* | Vector | Hemiptera | DTUIV | -10.20000000 | -63.03000000 | Marcili et al. 2009b | Google earth | Locality | II | III |
| *Rhodnius robustus* | Vector | Hemiptera | DTUIV | 3.16602222 | -52.34111111 | Cura et al. 2015 | Google earth | Locality | II | III |
| *Rhodnius robustus* | Vector | Hemiptera | DTUI | 4.92222222 | -52.31333333 | Cura et al. 2015 | Google earth | Locality | I | I |
| *Rhodnius robustus* | Vector | Hemiptera | DTUI | 4.92222222 | -52.31333333 | Cura et al. 2015 | Google earth | Locality | I | I |
| *Rhodnius robustus* | Vector | Hemiptera | DTUI | 4.92222222 | -52.31333333 | Cura et al. 2015 | Google earth | Locality | I | I |
| *Rhodnius robustus* | Vector | Hemiptera | DTUI | 4.92222222 | -52.31333333 | Cura et al. 2015 | Google earth | Locality | I | I |
| *Rhodnius robustus* | Vector | Hemiptera | DTUI | 4.88250000 | -52.28666667 | Cura et al. 2015 | Google earth | Locality | I | I |
| *Rhodnius robustus* | Vector | Hemiptera | DTUI | 4.88250000 | -52.28666667 | Cura et al. 2015 | Google earth | Locality | I | I |
| *Rhodnius robustus* | Vector | Hemiptera | DTUI | 4.88250000 | -52.28666667 | Cura et al. 2015 | Google earth | Locality | I | I |
| *Rhodnius robustus* | Vector | Hemiptera | DTUI | 4.88250000 | -52.28666667 | Cura et al. 2015 | Google earth | Locality | I | I |
| *Rhodnius robustus* | Vector | Hemiptera | DTUI | 3.86027778 | -53.30416667 | Cura et al. 2015 | Google earth | Locality | I | I |
| *Rhodnius stali* | Vector | Hemiptera | DTUI | -15.08300000 | -67.55000000 | Barnabe et al. 2011 | Google earth | Province | I | I |
| *Rhodnius stali* | Vector | Hemiptera | DTUI | -15.08300000 | -67.55000000 | Barnabe et al. 2011 | Google earth | Province | I | I |
| *Rhodnius stali* | Vector | Hemiptera | DTUI | -15.08300000 | -67.55000000 | Barnabe et al. 2011 | Google earth | Province | I | I |
| *Rhodnius pictipes* | Vector | Hemiptera | DTUI | 10.25000000 | -66.41670000 | Carrasco et al. 2012 | Google earth | State | I | I |
| *Rhodnius pictipes* | Vector | Hemiptera | DTUI | 10.21670000 | -67.28340000 | Carrasco et al. 2012 | Google earth | State | I | I |
| *Saguimus bicolor* | Host | Primates | DTUI | -3.11600000 | -60.01600000 | Marcili et al. 2009b | Google earth | Locality | I | I |
| *Saguimus bicolor* | Host | Primates | DTUI | -3.11600000 | -60.01600000 | Marcili et al. 2009b | Google earth | Locality | I | I |
| *Saguimus bicolor* | Host | Primates | DTUI | -3.11600000 | -60.01600000 | Marcili et al. 2009b | Google earth | Locality | I | I |
| *Saguimus bicolor* | Host | Primates | DTUI | -3.11600000 | -60.01600000 | Marcili et al. 2009b | Google earth | Locality | I | I |
| *Saguimus bicolor* | Host | Primates | DTUI | -3.11600000 | -60.01600000 | Marcili et al. 2009b | Google earth | Locality | I | I |
| *Saguimus fuscicollis* | Host | Primates | DTUIV | -10.21600000 | -67.28300000 | Marcili et al. 2009b | Google earth | Locality | II | III |
| *Saguimus labiatus* | Host | Primates | DTUIV | -10.21600000 | -67.28300000 | Marcili et al. 2009b | Google earth | Locality | II | III |
| *Saguimus midas* | Host | Primates | DTUI | -3.11600000 | -60.01600000 | Marcili et al. 2009b | Google earth | Locality | I | I |
| *Saguimus ustus* | Host | Primates | DTUIV | -3.11600000 | -60.01600000 | Marcili et al. 2009b | Google earth | Locality | II | III |
| *Saimiri sciureus* | Host | Primates | DTUI | -0.68300000 | -49.68300000 | Marcili et al. 2009b | Google earth | Locality | I | I |
| *Saimiri sciureus* | Host | Rodentia | DTUI | -0.68300000 | -49.68300000 | Marcili et al. 2009b | Google earth | Locality | I | I |
| *Saimiri sciureus* | Host | Rodentia | DTUI | 14.60000000 | -90.53300000 | Higo et al. 2004 | Google earth | Department/State | I | I |
| *Sciurus spadiceus* | Host | Rodentia | DTUI | -14.81000000 | -64.60000000 | Llewellyn et al. 2009b | Author | NA | I | I |
| *Sciurus stramineus* | Host | Rodentia | DTUI | -4.07800000 | -79.80600000 | Ocaña-Mayorga et al. 2010 | Author | NA | I | I |
| *Sigmodon hispidus* | Host | Rodentia | DTUVI | 18.58709584 | -89.42113386 | Lopez-Cancino et al. 2015 | Author | NA | II | ND |
| *Sus scrofa domestica* | Host | Artiodactyla | DTUVI | 18.59165452 | -89.41611704 | Lopez-Cancino et al. 2015 | Author | NA | II | ND |
| *Thrichomys a. laurentius* | Host | Rodentia | DTUIII | -8.833333 | -42.566667 | Araujo et al. 2011 | Google earth | Locality | II | III |
| *Thrichomys a. laurentius* | Host | Rodentia | DTUVI | -9.71666667 | -45.28333333 | Araujo et al. 2011 | Google earth | Locality | II | ND |
| *Thyroptera tricolor* | Host | Chiroptera | DTUI | -3.4652778 | -62.21583 | Marcili et al. 2009a | Google earth | State | I | I |
| *Triatoma barberi* | Vector | Hemiptera | DTUI | 20.59400000 | -100.39300000 | Zumaya-Estrada et al. 2012 | Author | NA | I | I |
| *Triatoma barberi* | Vector | Hemiptera | DTUI | 20.09080000 | -101.74020000 | Ibañez-Cervantes et al. 2013 | Google earth | Area/Unspecified communities | I | I |
| *Triatoma brasiliensis* | Vector | Hemiptera | DTUII | -8.151389 | -42.472500 | Araujo et al. 2011 | Google earth | Locality | II | II |
| *Triatoma brasiliensis* | Vector | Hemiptera | DTUII | -8.151389 | -42.472500 | Araujo et al. 2011 | Google earth | Locality | II | II |
| *Triatoma brasiliensis* | Vector | Hemiptera | DTUII | -8.151389 | -42.472500 | Araujo et al. 2011 | Google earth | Locality | II | II |
| *Triatoma brasiliensis* | Vector | Hemiptera | DTUII | -6.45000000 | -37.08300000 | Camara et al. 2010 | Google earth | Area/Unspecified communities | II | II |
| *Triatoma carrioni* | Vector | Hemiptera | DTUI | -4.18300000 | -79.52100000 | Ocaña-Mayorga et al. 2010 | Author | NA | I | I |
| *Triatoma carrioni* | Vector | Hemiptera | DTUI | -4.18800000 | -79.51700000 | Ocaña-Mayorga et al. 2010 | Author | NA | I | I |
| *Triatoma carrioni* | Vector | Hemiptera | DTUI | -4.18400000 | -79.52100000 | Ocaña-Mayorga et al. 2010 | Author | NA | I | I |
| *Triatoma dimidiata* | Vector | Hemiptera | DTUI | 18.60000000 | -96.68300000 | Ramos-Ligonio et al. 2012 | Google earth | Author | I | I |
| *Triatoma dimidiata* | Vector | Hemiptera | DTUI | 18.60000000 | -96.68300000 | Ramos-Ligonio et al. 2012 | Google earth | Author | I | I |
| *Triatoma dimidiata* | Vector | Hemiptera | DTUI | 18.60000000 | -96.68300000 | Ramos-Ligonio et al. 2012 | Google earth | Author | I | I |
| *Triatoma dimidiata* | Vector | Hemiptera | DTUI | 18.60000000 | -96.68300000 | Ramos-Ligonio et al. 2012 | Google earth | Author | I | I |
| *Triatoma dimidiata* | Vector | Hemiptera | DTUI | 18.60000000 | -96.68300000 | Ramos-Ligonio et al. 2012 | Google earth | Author | I | I |
| *Triatoma dimidiata* | Vector | Hemiptera | DTUI | 18.60000000 | -96.68300000 | Ramos-Ligonio et al. 2012 | Google earth | Author | I | I |
| *Triatoma dimidiata* | Vector | Hemiptera | DTUI | 18.60000000 | -96.68300000 | Ramos-Ligonio et al. 2012 | Google earth | Author | I | I |
| *Triatoma dimidiata* | Vector | Hemiptera | DTUI | 18.60000000 | -96.68300000 | Ramos-Ligonio et al. 2012 | Google earth | Author | I | I |
| *Triatoma dimidiata* | Vector | Hemiptera | DTUI | 18.60000000 | -96.68300000 | Ramos-Ligonio et al. 2012 | Google earth | Author | I | I |
| *Triatoma dimidiata* | Vector | Hemiptera | DTUI | 14.18300000 | -90.36700000 | Higo et al. 2004 | Google earth | Department/State | I | I |
| *Triatoma dimidiata* | Vector | Hemiptera | DTUI | 14.18300000 | -90.36700000 | Higo et al. 2004 | Google earth | Department/State | I | I |
| *Triatoma dimidiata* | Vector | Hemiptera | DTUI | 14.18300000 | -90.36700000 | Higo et al. 2004 | Google earth | Department/State | I | I |
| *Triatoma dimidiata* | Vector | Hemiptera | DTUI | 14.18300000 | -90.36700000 | Higo et al. 2004 | Google earth | Department/State | I | I |
| *Triatoma dimidiata* | Vector | Hemiptera | DTUI | 14.18300000 | -90.36700000 | Higo et al. 2004 | Google earth | Department/State | I | I |
| *Triatoma dimidiata* | Vector | Hemiptera | DTUI | 14.18300000 | -90.36700000 | Higo et al. 2004 | Google earth | Department/State | I | I |
| *Triatoma dimidiata* | Vector | Hemiptera | DTUI | 14.18300000 | -90.36700000 | Higo et al. 2004 | Google earth | Department/State | I | I |
| *Triatoma dimidiata* | Vector | Hemiptera | DTUI | 14.18300000 | -90.36700000 | Higo et al. 2004 | Google earth | Department/State | I | I |
| *Triatoma dimidiata* | Vector | Hemiptera | DTUI | 14.18300000 | -90.36700000 | Higo et al. 2004 | Google earth | Department/State | I | I |
| *Triatoma dimidiata* | Vector | Hemiptera | DTUI | 14.18300000 | -90.36700000 | Higo et al. 2004 | Google earth | Department/State | I | I |
| *Triatoma dimidiata* | Vector | Hemiptera | DTUI | 14.18300000 | -90.36700000 | Higo et al. 2004 | Google earth | Department/State | I | I |
| *Triatoma dimidiata* | Vector | Hemiptera | DTUI | 14.18300000 | -90.36700000 | Higo et al. 2004 | Google earth | Department/State | I | I |
| *Triatoma dimidiata* | Vector | Hemiptera | DTUI | 14.18300000 | -90.36700000 | Higo et al. 2004 | Google earth | Department/State | I | I |
| *Triatoma dimidiata* | Vector | Hemiptera | DTUI | 14.18300000 | -90.36700000 | Higo et al. 2004 | Google earth | Department/State | I | I |
| *Triatoma dimidiata* | Vector | Hemiptera | DTUI | 14.18300000 | -90.36700000 | Higo et al. 2004 | Google earth | Department/State | I | I |
| *Triatoma dimidiata* | Vector | Hemiptera | DTUI | 14.18300000 | -90.36700000 | Higo et al. 2004 | Google earth | Department/State | I | I |
| *Triatoma dimidiata* | Vector | Hemiptera | DTUI | 14.18300000 | -90.36700000 | Higo et al. 2004 | Google earth | Department/State | I | I |
| *Triatoma dimidiata* | Vector | Hemiptera | DTUI | 14.18300000 | -90.36700000 | Higo et al. 2004 | Google earth | Department/State | I | I |
| *Triatoma dimidiata* | Vector | Hemiptera | DTUI | 14.18300000 | -90.36700000 | Higo et al. 2004 | Google earth | Department/State | I | I |
| *Triatoma dimidiata* | Vector | Hemiptera | DTUI | 14.18300000 | -90.36700000 | Higo et al. 2004 | Google earth | Department/State | I | I |
| *Triatoma dimidiata* | Vector | Hemiptera | DTUI | 14.18300000 | -90.36700000 | Higo et al. 2004 | Google earth | Department/State | I | I |
| *Triatoma dimidiata* | Vector | Hemiptera | DTUI | 14.18300000 | -90.36700000 | Higo et al. 2004 | Google earth | Department/State | I | I |
| *Triatoma dimidiata* | Vector | Hemiptera | DTUI | 14.18300000 | -90.36700000 | Higo et al. 2004 | Google earth | Department/State | I | I |
| *Triatoma dimidiata* | Vector | Hemiptera | DTUI | 14.18300000 | -90.36700000 | Higo et al. 2004 | Google earth | Department/State | I | I |
| *Triatoma dimidiata* | Vector | Hemiptera | DTUI | 14.18300000 | -90.36700000 | Higo et al. 2004 | Google earth | Department/State | I | I |
| *Triatoma dimidiata* | Vector | Hemiptera | DTUI | 14.18300000 | -90.36700000 | Higo et al. 2004 | Google earth | Department/State | I | I |
| *Triatoma dimidiata* | Vector | Hemiptera | DTUI | 14.18300000 | -90.36700000 | Higo et al. 2004 | Google earth | Department/State | I | I |
| *Triatoma dimidiata* | Vector | Hemiptera | DTUI | 14.18300000 | -90.36700000 | Higo et al. 2004 | Google earth | Department/State | I | I |
| *Triatoma dimidiata* | Vector | Hemiptera | DTUI | 14.18300000 | -90.36700000 | Higo et al. 2004 | Google earth | Department/State | I | I |
| *Triatoma dimidiata* | Vector | Hemiptera | DTUI | 14.18300000 | -90.36700000 | Higo et al. 2004 | Google earth | Department/State | I | I |
| *Triatoma dimidiata* | Vector | Hemiptera | DTUI | 14.18300000 | -90.36700000 | Higo et al. 2004 | Google earth | Department/State | I | I |
| *Triatoma dimidiata* | Vector | Hemiptera | DTUI | 14.18300000 | -90.36700000 | Higo et al. 2004 | Google earth | Department/State | I | I |
| *Triatoma dimidiata* | Vector | Hemiptera | DTUI | 14.18300000 | -90.36700000 | Higo et al. 2004 | Google earth | Department/State | I | I |
| *Triatoma dimidiata* | Vector | Hemiptera | DTUI | 14.18300000 | -90.36700000 | Higo et al. 2004 | Google earth | Department/State | I | I |
| *Triatoma dimidiata* | Vector | Hemiptera | DTUI | 14.60000000 | -90.53300000 | Higo et al. 2004 | Google earth | Department/State | I | I |
| *Triatoma dimidiata* | Vector | Hemiptera | DTUI | 14.60000000 | -90.53300000 | Higo et al. 2004 | Google earth | Department/State | I | I |
| *Triatoma dimidiata* | Vector | Hemiptera | DTUI | 14.18300000 | -90.36700000 | Higo et al. 2004 | Google earth | Department/State | I | I |
| *Triatoma dimidiata* | Vector | Hemiptera | DTUI | 14.18300000 | -90.36700000 | Higo et al. 2004 | Google earth | Department/State | I | I |
| *Triatoma dimidiata* | Vector | Hemiptera | DTUI | 14.18300000 | -90.36700000 | Higo et al. 2004 | Google earth | Department/State | I | I |
| *Triatoma dimidiata* | Vector | Hemiptera | DTUI | 14.18330000 | -90.36700000 | Higo et al. 2004 | Google earth | Department/State | I | I |
| *Triatoma dimidiata* | Vector | Hemiptera | DTUI | 14.18300000 | -90.36700000 | Higo et al. 2004 | Google earth | Department/State | I | I |
| *Triatoma dimidiata* | Vector | Hemiptera | DTUI | 14.18300000 | -90.36700000 | Higo et al. 2004 | Google earth | Department/State | I | I |
| *Triatoma dimidiata* | Vector | Hemiptera | DTUII | 18.60000000 | -96.68300000 | Ramos-Ligonio et al. 2012 | Google earth | Author | II | II |
| *Triatoma dimidiata* | Vector | Hemiptera | DTUII | 18.60000000 | -96.68300000 | Ramos-Ligonio et al. 2012 | Google earth | Author | II | II |
| *Triatoma dimidiata* | Vector | Hemiptera | DTUII | 18.60000000 | -96.68300000 | Ramos-Ligonio et al. 2012 | Google earth | Author | II | II |
| *Triatoma dimidiata* | Vector | Hemiptera | DTUII | 18.60000000 | -96.68300000 | Ramos-Ligonio et al. 2012 | Google earth | Author | II | II |
| *Triatoma dimidiata* | Vector | Hemiptera | DTUIII | 18.60000000 | -96.68300000 | Ramos-Ligonio et al. 2012 | Google earth | Author | II | III |
| *Triatoma dimidiata* | Vector | Hemiptera | DTUIII | 18.60000000 | -96.68300000 | Ramos-Ligonio et al. 2012 | Google earth | Author | II | III |
| *Triatoma dimidiata* | Vector | Hemiptera | DTUIII | 18.60000000 | -96.68300000 | Ramos-Ligonio et al. 2012 | Google earth | Author | II | III |
| *Triatoma dimidiata* | Vector | Hemiptera | DTUIII | 18.60000000 | -96.68300000 | Ramos-Ligonio et al. 2012 | Google earth | Author | II | III |
| *Triatoma dimidiata* | Vector | Hemiptera | DTUIII | 18.60000000 | -96.68300000 | Ramos-Ligonio et al. 2012 | Google earth | Author | II | III |
| *Triatoma dimidiata* | Vector | Hemiptera | DTUIV | 18.60000000 | -96.68300000 | Ramos-Ligonio et al. 2012 | Google earth | Author | II | III |
| *Triatoma dimidiata* | Vector | Hemiptera | DTUIV | 18.60000000 | -96.68300000 | Ramos-Ligonio et al. 2012 | Google earth | Author | II | III |
| *Triatoma dimidiata* | Vector | Hemiptera | DTUIV | 18.60000000 | -96.68300000 | Ramos-Ligonio et al. 2012 | Google earth | Author | II | III |
| *Triatoma dimidiata* | Vector | Hemiptera | DTUIV | 18.60000000 | -96.68300000 | Ramos-Ligonio et al. 2012 | Google earth | Author | II | III |
| *Triatoma dimidiata* | Vector | Hemiptera | DTUIV | 18.60000000 | -96.68300000 | Ramos-Ligonio et al. 2012 | Google earth | Author | II | III |
| *Triatoma dimidiata* | Vector | Hemiptera | DTUIV | 18.60000000 | -96.68300000 | Ramos-Ligonio et al. 2012 | Google earth | Author | II | III |
| *Triatoma dimidiata* | Vector | Hemiptera | DTUV | 18.60000000 | -96.68300000 | Ramos-Ligonio et al. 2012 | Google earth | Author | II | ND |
| *Triatoma dimidiata* | Vector | Hemiptera | DTUV | 18.60000000 | -96.68300000 | Ramos-Ligonio et al. 2012 | Google earth | Author | II | ND |
| *Triatoma dimidiata* | Vector | Hemiptera | DTUV | 18.60000000 | -96.68300000 | Ramos-Ligonio et al. 2012 | Google earth | Author | II | ND |
| *Triatoma dimidiata* | Vector | Hemiptera | DTUV | 18.60000000 | -96.68300000 | Ramos-Ligonio et al. 2012 | Google earth | Author | II | ND |
| *Triatoma dimidiata* | Vector | Hemiptera | DTUV | 18.60000000 | -96.68300000 | Ramos-Ligonio et al. 2012 | Google earth | Author | II | ND |
| *Triatoma dimidiata* | Vector | Hemiptera | DTUV | 18.60000000 | -96.68300000 | Ramos-Ligonio et al. 2012 | Google earth | Author | II | ND |
| *Triatoma dimidiata* | Vector | Hemiptera | DTUV | 18.60000000 | -96.68300000 | Ramos-Ligonio et al. 2012 | Google earth | Author | II | ND |
| *Triatoma dimidiata* | Vector | Hemiptera | DTUV | 18.60000000 | -96.68300000 | Ramos-Ligonio et al. 2012 | Google earth | Author | II | ND |
| *Triatoma dimidiata* | Vector | Hemiptera | DTUV | 18.60000000 | -96.68300000 | Ramos-Ligonio et al. 2012 | Google earth | Author | II | ND |
| *Triatoma dimidiata* | Vector | Hemiptera | DTUVI | 18.59472320 | -89.41829014 | Lopez-Cancino et al. 2015 | Author | NA | II | ND |
| *Triatoma dimidiata* | Vector | Hemiptera | DTUVI | 18.59472279 | -89.41828427 | Lopez-Cancino et al. 2015 | Author | NA | II | ND |
| *Triatoma dimidiata* | Vector | Hemiptera | DTUVI | 18.59524984 | -89.41610129 | Lopez-Cancino et al. 2015 | Author | NA | II | ND |
| *Triatoma dimidiata* | Vector | Hemiptera | DTUVI | 18.59524984 | -89.41610129 | Lopez-Cancino et al. 2015 | Author | NA | II | ND |
| *Triatoma dimidiata* | Vector | Hemiptera | DTUVI | 18.57787000 | -89.42621000 | Lopez-Cancino et al. 2015 | Author | NA | II | ND |
| *Triatoma dimidiata* | Vector | Hemiptera | DTUI | 14.08000000 | -87.20000000 | Llewellyn et al. 2009b | Author | NA | I | I |
| *Triatoma dimidiata* | Vector | Hemiptera | DTUI | 10.95000000 | -73.46700000 | Rodriguez et al. 2009 | Google earth | Area/Unspecified communities | I | I |
| *Triatoma dimidiata* | Vector | Hemiptera | DTUI | 10.95000000 | -73.46700000 | Rodriguez et al. 2009 | Google earth | Area/Unspecified communities | I | I |
| *Triatoma dimidiata* | Vector | Hemiptera | DTUI | 10.95000000 | -73.46700000 | Rodriguez et al. 2009 | Google earth | Area/Unspecified communities | I | I |
| *Triatoma dimidiata* | Vector | Hemiptera | DTUI | 10.95000000 | -73.46700000 | Rodriguez et al. 2009 | Google earth | Area/Unspecified communities | I | I |
| *Triatoma dimidiata* | Vector | Hemiptera | DTUI | 10.95000000 | -73.46700000 | Rodriguez et al. 2009 | Google earth | Area/Unspecified communities | I | I |
| *Triatoma dimidiata* | Vector | Hemiptera | DTUI | 10.95000000 | -73.46700000 | Rodriguez et al. 2009 | Google earth | Area/Unspecified communities | I | I |
| *Triatoma dimidiata* | Vector | Hemiptera | DTUI | 10.95000000 | -73.46700000 | Rodriguez et al. 2009 | Google earth | Area/Unspecified communities | I | I |
| *Triatoma dimidiata* | Vector | Hemiptera | DTUI | 10.95000000 | -73.46700000 | Rodriguez et al. 2009 | Google earth | Area/Unspecified communities | I | I |
| *Triatoma dimidiata* | Vector | Hemiptera | DTUI | 10.95000000 | -73.46700000 | Rodriguez et al. 2009 | Google earth | Area/Unspecified communities | I | I |
| *Triatoma dimidiata* | Vector | Hemiptera | DTUI | 10.95000000 | -73.46700000 | Rodriguez et al. 2009 | Google earth | Area/Unspecified communities | I | I |
| *Triatoma dimidiata* | Vector | Hemiptera | DTUI | 6.27000000 | -71.20000000 | Ramirez et al. 2013 | Author | NA | I | I |
| *Triatoma dimidiata* | Vector | Hemiptera | DTUI | 14.28700000 | -89.84400000 | Zumaya-Estrada et al. 2012 | Author | NA | I | I |
| *Triatoma dimidiata* | Vector | Hemiptera | DTUI | 14.17700000 | -90.30300000 | Zumaya-Estrada et al. 2012 | Author | NA | I | I |
| *Triatoma dimidiata* | Vector | Hemiptera | DTUI | 14.63300000 | -89.98900000 | Zumaya-Estrada et al. 2012 | Author | NA | I | I |
| *Triatoma dimidiata* | Vector | Hemiptera | DTUI | 14.28700000 | -89.84400000 | Zumaya-Estrada et al. 2012 | Author | NA | I | I |
| *Triatoma dimidiata* | Vector | Hemiptera | DTUI | 14.28700000 | -89.84400000 | Zumaya-Estrada et al. 2012 | Author | NA | I | I |
| *Triatoma dimidiata* | Vector | Hemiptera | DTUI | 14.63300000 | -89.98900000 | Zumaya-Estrada et al. 2012 | Author | NA | I | I |
| *Triatoma dimidiata* | Vector | Hemiptera | DTUI | 14.76800000 | -84.45800000 | Zumaya-Estrada et al. 2012 | Author | NA | I | I |
| *Triatoma dimidiata* | Vector | Hemiptera | DTUI | 14.76800000 | -84.45800000 | Zumaya-Estrada et al. 2012 | Author | NA | I | I |
| *Triatoma dimidiata* | Vector | Hemiptera | DTUI | 14.17700000 | -90.30300000 | Zumaya-Estrada et al. 2012 | Author | NA | I | I |
| *Triatoma dimidiata* | Vector | Hemiptera | DTUI | 14.76800000 | -89.45800000 | Zumaya-Estrada et al. 2012 | Author | NA | I | I |
| *Triatoma dimidiata* | Vector | Hemiptera | DTUI | 15.07900000 | -90.41300000 | Zumaya-Estrada et al. 2012 | Author | NA | I | I |
| *Triatoma dimidiata* | Vector | Hemiptera | DTUI | 15.59400000 | -90.14900000 | Zumaya-Estrada et al. 2012 | Author | NA | I | I |
| *Triatoma dimidiata* | Vector | Hemiptera | DTUI | 14.08000000 | -87.20000000 | Messenger et al. 2012 | Author | NA | I | I |
| *Triatoma dimidiata* | Vector | Hemiptera | DTUI | 19.18800000 | -90.30000000 | Zumaya-Estrada et al. 2012 | Author | NA | I | I |
| *Triatoma dimidiata* | Vector | Hemiptera | DTUI | 19.18800000 | -90.30000000 | Zumaya-Estrada et al. 2012 | Author | NA | I | I |
| *Triatoma dimidiata* | Vector | Hemiptera | DTUI | 19.56700000 | -101.70700000 | Zumaya-Estrada et al. 2012 | Author | NA | I | I |
| *Triatoma dimidiata* | Vector | Hemiptera | DTUI | 19.17300000 | -96.13300000 | Zumaya-Estrada et al. 2012 | Author | NA | I | I |
| *Triatoma dimidiata* | Vector | Hemiptera | DTUI | 8.40000000 | -75.88300000 | Mejia-Jaramillo et al. 2009 | Google earth | Department/State | I | I |
| *Triatoma dimidiata* | Vector | Hemiptera | DTUI | 8.40000000 | -75.88300000 | Mejia-Jaramillo et al. 2009 | Google earth | Department/State | I | I |
| *Triatoma dimidiata* | Vector | Hemiptera | DTUI | 10.40000000 | -74.40000000 | Mejia-Jaramillo et al. 2009 | Google earth | Department/State | I | I |
| *Triatoma dimidiata* | Vector | Hemiptera | DTUI | 10.40000000 | -74.40000000 | Mejia-Jaramillo et al. 2009 | Google earth | Department/State | I | I |
| *Triatoma dimidiata* | Vector | Hemiptera | DTUI | 10.40000000 | -74.40000000 | Mejia-Jaramillo et al. 2009 | Google earth | Department/State | I | I |
| *Triatoma dimidiata* | Vector | Hemiptera | DTUI | 20.63916667 | -88.29777778 | Cura et al. 2015 | Google earth | Locality | I | I |
| *Triatoma dimidiata* | Vector | Hemiptera | DTUI | 20.63916667 | -88.29777778 | Cura et al. 2015 | Google earth | Locality | I | I |
| *Triatoma dimidiata* | Vector | Hemiptera | DTUI | 17.48472222 | -92.04583333 | Cura et al. 2015 | Google earth | Locality | I | I |
| *Triatoma dimidiata* | Vector | Hemiptera | DTUI | 17.48472222 | -92.04583333 | Cura et al. 2015 | Google earth | Locality | I | I |
| *Triatoma dimidiata* | Vector | Hemiptera | DTUI | 17.48472222 | -92.04583333 | Cura et al. 2015 | Google earth | Locality | I | I |
| *Triatoma dimidiata* | Vector | Hemiptera | DTUI | 20.09805556 | -89.46277778 | Cura et al. 2015 | Google earth | Locality | I | I |
| *Triatoma gerstaeckeri* | Vector | Hemiptera | DTUI | 31.96833333 | -99.90166667 | Cura et al. 2015 | Google earth | State | I | I |
| *Triatoma gerstaeckeri* | Vector | Hemiptera | DTUIV | 31.96833333 | -99.90166667 | Cura et al. 2015 | Google earth | State | II | III |
| *Triatoma infestans* | Vector | Hemiptera | DTUI | -26.58300000 | -60.95000000 | Lauthier et al. 2012 | Google earth | Province | I | I |
| *Triatoma infestans* | Vector | Hemiptera | DTUI | -26.58300000 | -60.95000000 | Lauthier et al. 2012 | Google earth | Province | I | I |
| *Triatoma infestans* | Vector | Hemiptera | DTUI | -26.58300000 | -60.95000000 | Lauthier et al. 2012 | Google earth | Province | I | I |
| *Triatoma infestans* | Vector | Hemiptera | DTUV | -26.58300000 | -60.95000000 | Lauthier et al. 2012 | Google earth | Province | II | ND |
| *Triatoma infestans* | Vector | Hemiptera | DTUV | -26.58300000 | -60.95000000 | Lauthier et al. 2012 | Google earth | Province | II | ND |
| *Triatoma infestans* | Vector | Hemiptera | DTUV | -25.08333333 | -56.96666667 | Maffey et al. 2012 | Google earth | Locality | II | ND |
| *Triatoma infestans* | Vector | Hemiptera | DTUV | -25.08333333 | -56.96666667 | Maffey et al. 2012 | Google earth | Locality | II | ND |
| *Triatoma infestans* | Vector | Hemiptera | DTUV | -25.08333333 | -56.96666667 | Maffey et al. 2012 | Google earth | Locality | II | ND |
| *Triatoma infestans* | Vector | Hemiptera | DTUV | -25.08333333 | -56.96666667 | Maffey et al. 2012 | Google earth | Locality | II | ND |
| *Triatoma infestans* | Vector | Hemiptera | DTUV | -25.08333333 | -56.96666667 | Maffey et al. 2012 | Google earth | Locality | II | ND |
| *Triatoma infestans* | Vector | Hemiptera | DTUV | -25.08333333 | -56.96666667 | Maffey et al. 2012 | Google earth | Locality | II | ND |
| *Triatoma infestans* | Vector | Hemiptera | DTUV | -25.08333333 | -56.96666667 | Maffey et al. 2012 | Google earth | Locality | II | ND |
| *Triatoma infestans* | Vector | Hemiptera | DTUV | -25.08333333 | -56.96666667 | Maffey et al. 2012 | Google earth | Locality | II | ND |
| *Triatoma infestans* | Vector | Hemiptera | DTUV | -25.08333333 | -56.96666667 | Maffey et al. 2012 | Google earth | Locality | II | ND |
| *Triatoma infestans* | Vector | Hemiptera | DTUV | -25.08333333 | -56.96666667 | Maffey et al. 2012 | Google earth | Locality | II | ND |
| *Triatoma infestans* | Vector | Hemiptera | DTUV | -25.08333333 | -56.96666667 | Maffey et al. 2012 | Google earth | Locality | II | ND |
| *Triatoma infestans* | Vector | Hemiptera | DTUVI | -26.58300000 | -60.95000000 | Lauthier et al. 2012 | Google earth | Province | II | ND |
| *Triatoma infestans* | Vector | Hemiptera | DTUVI | -26.58300000 | -60.95000000 | Lauthier et al. 2012 | Google earth | Province | II | ND |
| *Triatoma infestans* | Vector | Hemiptera | DTUVI | -26.58300000 | -60.95000000 | Lauthier et al. 2012 | Google earth | Province | II | ND |
| *Triatoma infestans* | Vector | Hemiptera | DTUVI | -25.08333333 | -56.96666667 | Maffey et al. 2012 | Google earth | Locality | II | ND |
| *Triatoma infestans* | Vector | Hemiptera | DTUVI | -25.08333333 | -56.96666667 | Maffey et al. 2012 | Google earth | Locality | II | ND |
| *Triatoma infestans* | Vector | Hemiptera | DTUVI | -25.08333333 | -56.96666667 | Maffey et al. 2012 | Google earth | Locality | II | ND |
| *Triatoma infestans* | Vector | Hemiptera | DTUVI | -25.08333333 | -56.96666667 | Maffey et al. 2012 | Google earth | Locality | II | ND |
| *Triatoma infestans* | Vector | Hemiptera | DTUVI | -25.08333333 | -56.96666667 | Maffey et al. 2012 | Google earth | Locality | II | ND |
| *Triatoma infestans* | Vector | Hemiptera | DTUVI | -25.08333333 | -56.96666667 | Maffey et al. 2012 | Google earth | Locality | II | ND |
| *Triatoma infestans* | Vector | Hemiptera | DTUVI | -25.08333333 | -56.96666667 | Maffey et al. 2012 | Google earth | Locality | II | ND |
| *Triatoma infestans* | Vector | Hemiptera | DTUVI | -25.08333333 | -56.96666667 | Maffey et al. 2012 | Google earth | Locality | II | ND |
| *Triatoma infestans* | Vector | Hemiptera | DTUVI | -25.08333333 | -56.96666667 | Maffey et al. 2012 | Google earth | Locality | II | ND |
| *Triatoma infestans* | Vector | Hemiptera | DTUVI | -25.08333333 | -56.96666667 | Maffey et al. 2012 | Google earth | Locality | II | ND |
| *Triatoma infestans* | Vector | Hemiptera | DTUVI | -25.08333333 | -56.96666667 | Maffey et al. 2012 | Google earth | Locality | II | ND |
| *Triatoma infestans* | Vector | Hemiptera | DTUVI | -25.08333333 | -56.96666667 | Maffey et al. 2012 | Google earth | Locality | II | ND |
| *Triatoma infestans* | Vector | Hemiptera | DTUVI | -25.08333333 | -56.96666667 | Maffey et al. 2012 | Google earth | Locality | II | ND |
| *Triatoma infestans* | Vector | Hemiptera | DTUVI | -25.08333333 | -56.96666667 | Maffey et al. 2012 | Google earth | Locality | II | ND |
| *Triatoma infestans* | Vector | Hemiptera | DTUVI | -25.08333333 | -56.96666667 | Maffey et al. 2012 | Google earth | Locality | II | ND |
| *Triatoma infestans* | Vector | Hemiptera | DTUVI | -25.08333333 | -56.96666667 | Maffey et al. 2012 | Google earth | Locality | II | ND |
| *Triatoma infestans* | Vector | Hemiptera | DTUVI | -25.08333333 | -56.96666667 | Maffey et al. 2012 | Google earth | Locality | II | ND |
| *Triatoma infestans* | Vector | Hemiptera | DTUVI | -25.08333333 | -56.96666667 | Maffey et al. 2012 | Google earth | Locality | II | ND |
| *Triatoma infestans* | Vector | Hemiptera | DTUVI | -25.08333333 | -56.96666667 | Maffey et al. 2012 | Google earth | Locality | II | ND |
| *Triatoma infestans* | Vector | Hemiptera | DTUVI | -25.08333333 | -56.96666667 | Maffey et al. 2012 | Google earth | Locality | II | ND |
| *Triatoma infestans* | Vector | Hemiptera | DTUVI | -25.08333333 | -56.96666667 | Maffey et al. 2012 | Google earth | Locality | II | ND |
| *Triatoma infestans* | Vector | Hemiptera | DTUVI | -25.08333333 | -56.96666667 | Maffey et al. 2012 | Google earth | Locality | II | ND |
| *Triatoma infestans* | Vector | Hemiptera | DTUVI | -25.08333333 | -56.96666667 | Maffey et al. 2012 | Google earth | Locality | II | ND |
| *Triatoma infestans* | Vector | Hemiptera | DTUVI | -25.08333333 | -56.96666667 | Maffey et al. 2012 | Google earth | Locality | II | ND |
| *Triatoma infestans* | Vector | Hemiptera | DTUVI | -25.08333333 | -56.96666667 | Maffey et al. 2012 | Google earth | Locality | II | ND |
| *Triatoma infestans* | Vector | Hemiptera | DTUVI | -25.08333333 | -56.96666667 | Maffey et al. 2012 | Google earth | Locality | II | ND |
| *Triatoma infestans* | Vector | Hemiptera | DTUVI | -25.08333333 | -56.96666667 | Maffey et al. 2012 | Google earth | Locality | II | ND |
| *Triatoma infestans* | Vector | Hemiptera | DTUVI | -25.08333333 | -56.96666667 | Maffey et al. 2012 | Google earth | Locality | II | ND |
| *Triatoma infestans* | Vector | Hemiptera | DTUVI | -25.08333333 | -56.96666667 | Maffey et al. 2012 | Google earth | Locality | II | ND |
| *Triatoma infestans* | Vector | Hemiptera | DTUVI | -25.08333333 | -56.96666667 | Maffey et al. 2012 | Google earth | Locality | II | ND |
| *Triatoma infestans* | Vector | Hemiptera | DTUVI | -25.08333333 | -56.96666667 | Maffey et al. 2012 | Google earth | Locality | II | ND |
| *Triatoma infestans* | Vector | Hemiptera | DTUVI | -25.08333333 | -56.96666667 | Maffey et al. 2012 | Google earth | Locality | II | ND |
| *Triatoma infestans* | Vector | Hemiptera | DTUVI | -25.08333333 | -56.96666667 | Maffey et al. 2012 | Google earth | Locality | II | ND |
| *Triatoma infestans* | Vector | Hemiptera | DTUVI | -25.08333333 | -56.96666667 | Maffey et al. 2012 | Google earth | Locality | II | ND |
| *Triatoma infestans* | Vector | Hemiptera | DTUVI | -25.08333333 | -56.96666667 | Maffey et al. 2012 | Google earth | Locality | II | ND |
| *Triatoma infestans* | Vector | Hemiptera | DTUVI | -25.08333333 | -56.96666667 | Maffey et al. 2012 | Google earth | Locality | II | ND |
| *Triatoma infestans* | Vector | Hemiptera | DTUVI | -25.08333333 | -56.96666667 | Maffey et al. 2012 | Google earth | Locality | II | ND |
| *Triatoma infestans* | Vector | Hemiptera | DTUVI | -25.08333333 | -56.96666667 | Maffey et al. 2012 | Google earth | Locality | II | ND |
| *Triatoma infestans* | Vector | Hemiptera | DTUVI | -25.08333333 | -56.96666667 | Maffey et al. 2012 | Google earth | Locality | II | ND |
| *Triatoma infestans* | Vector | Hemiptera | DTUVI | -25.08333333 | -56.96666667 | Maffey et al. 2012 | Google earth | Locality | II | ND |
| *Triatoma infestans* | Vector | Hemiptera | DTUVI | -25.08333333 | -56.96666667 | Maffey et al. 2012 | Google earth | Locality | II | ND |
| *Triatoma infestans* | Vector | Hemiptera | DTUVI | -25.08333333 | -56.96666667 | Maffey et al. 2012 | Google earth | Locality | II | ND |
| *Triatoma infestans* | Vector | Hemiptera | DTUI | -27.13300000 | -61.46000000 | Llewellyn et al. 2009b | Author | NA | I | I |
| *Triatoma infestans* | Vector | Hemiptera | DTUI | -26.93000000 | -61.58000000 | Llewellyn et al. 2009b | Author | NA | I | I |
| *Triatoma infestans* | Vector | Hemiptera | DTUI | -27.45000000 | -70.25000000 | Venegas et al. 2011 | Author | NA | I | I |
| *Triatoma infestans* | Vector | Hemiptera | DTUI | -27.45000000 | -70.25000000 | Venegas et al. 2011 | Author | NA | I | I |
| *Triatoma infestans* | Vector | Hemiptera | DTUI | -27.45000000 | -70.25000000 | Venegas et al. 2011 | Author | NA | I | I |
| *Triatoma infestans* | Vector | Hemiptera | DTUI | -27.45000000 | -70.25000000 | Venegas et al. 2011 | Author | NA | I | I |
| *Triatoma infestans* | Vector | Hemiptera | DTUI | -32.26700000 | -70.96700000 | Venegas et al. 2011 | Author | NA | I | I |
| *Triatoma infestans* | Vector | Hemiptera | DTUI | -32.26700000 | -70.96700000 | Venegas et al. 2011 | Author | NA | I | I |
| *Triatoma infestans* | Vector | Hemiptera | DTUI | -32.26700000 | -70.96700000 | Venegas et al. 2011 | Author | NA | I | I |
| *Triatoma infestans* | Vector | Hemiptera | DTUI | -32.26700000 | -70.96700000 | Venegas et al. 2011 | Author | NA | I | I |
| *Triatoma infestans* | Vector | Hemiptera | DTUI | -32.26700000 | -70.96700000 | Venegas et al. 2011 | Author | NA | I | I |
| *Triatoma infestans* | Vector | Hemiptera | DTUI | -32.26700000 | -70.96700000 | Venegas et al. 2011 | Author | NA | I | I |
| *Triatoma infestans* | Vector | Hemiptera | DTUI | -32.26700000 | -70.96700000 | Venegas et al. 2011 | Author | NA | I | I |
| *Triatoma infestans* | Vector | Hemiptera | DTUI | -32.26700000 | -70.96700000 | Venegas et al. 2011 | Author | NA | I | I |
| *Triatoma infestans* | Vector | Hemiptera | DTUI | -32.26700000 | -70.96700000 | Venegas et al. 2011 | Author | NA | I | I |
| *Triatoma infestans* | Vector | Hemiptera | DTUI | -32.26700000 | -70.96700000 | Venegas et al. 2011 | Author | NA | I | I |
| *Triatoma infestans* | Vector | Hemiptera | DTUI | -32.26700000 | -70.96700000 | Venegas et al. 2011 | Author | NA | I | I |
| *Triatoma infestans* | Vector | Hemiptera | DTUI | -32.26700000 | -70.96700000 | Venegas et al. 2011 | Author | NA | I | I |
| *Triatoma infestans* | Vector | Hemiptera | DTUI | -32.26700000 | -70.96700000 | Venegas et al. 2011 | Author | NA | I | I |
| *Triatoma infestans* | Vector | Hemiptera | DTUI | -32.26700000 | -70.96700000 | Venegas et al. 2011 | Author | NA | I | I |
| *Triatoma infestans* | Vector | Hemiptera | DTUI | -33.61700000 | -70.78300000 | Venegas et al. 2011 | Author | NA | I | I |
| *Triatoma infestans* | Vector | Hemiptera | DTUI | -33.61700000 | -70.78300000 | Venegas et al. 2011 | Author | NA | I | I |
| *Triatoma infestans* | Vector | Hemiptera | DTUI | -33.61700000 | -70.78300000 | Venegas et al. 2011 | Author | NA | I | I |
| *Triatoma infestans* | Vector | Hemiptera | DTUI | -33.61700000 | -70.78300000 | Venegas et al. 2011 | Author | NA | I | I |
| *Triatoma infestans* | Vector | Hemiptera | DTUI | -33.61700000 | -70.78300000 | Venegas et al. 2011 | Author | NA | I | I |
| *Triatoma infestans* | Vector | Hemiptera | DTUI | -27.13300000 | -61.46000000 | Messenger et al. 2012 | Author | NA | I | I |
| *Triatoma infestans* | Vector | Hemiptera | DTUI | -17.38300000 | -66.01670000 | Barnabe et al. 2011 | Google earth | Province | I | I |
| *Triatoma infestans* | Vector | Hemiptera | DTUI | -17.38300000 | -66.01670000 | Barnabe et al. 2011 | Google earth | Province | I | I |
| *Triatoma infestans* | Vector | Hemiptera | DTUI | -17.38300000 | -66.01670000 | Barnabe et al. 2011 | Google earth | Province | I | I |
| *Triatoma infestans* | Vector | Hemiptera | DTUI | -17.70000000 | -66.25000000 | Barnabe et al. 2011 | Google earth | Province | I | I |
| *Triatoma infestans* | Vector | Hemiptera | DTUI | -17.70000000 | -66.25000000 | Barnabe et al. 2011 | Google earth | Province | I | I |
| *Triatoma infestans* | Vector | Hemiptera | DTUI | -17.70000000 | -66.25000000 | Barnabe et al. 2011 | Google earth | Province | I | I |
| *Triatoma infestans* | Vector | Hemiptera | DTUI | -17.70000000 | -66.25000000 | Barnabe et al. 2011 | Google earth | Province | I | I |
| *Triatoma infestans* | Vector | Hemiptera | DTUI | -17.70000000 | -66.25000000 | Barnabe et al. 2011 | Google earth | Province | I | I |
| *Triatoma infestans* | Vector | Hemiptera | DTUI | -17.70000000 | -66.25000000 | Barnabe et al. 2011 | Google earth | Province | I | I |
| *Triatoma infestans* | Vector | Hemiptera | DTUI | -17.70000000 | -66.25000000 | Barnabe et al. 2011 | Google earth | Province | I | I |
| *Triatoma infestans* | Vector | Hemiptera | DTUI | -17.70000000 | -66.25000000 | Barnabe et al. 2011 | Google earth | Province | I | I |
| *Triatoma infestans* | Vector | Hemiptera | DTUI | -17.70000000 | -66.25000000 | Barnabe et al. 2011 | Google earth | Province | I | I |
| *Triatoma infestans* | Vector | Hemiptera | DTUI | -16.15000000 | -67.65000000 | Barnabe et al. 2011 | Google earth | Province | I | I |
| *Triatoma infestans* | Vector | Hemiptera | DTUI | -16.15000000 | -67.65000000 | Barnabe et al. 2011 | Google earth | Province | I | I |
| *Triatoma infestans* | Vector | Hemiptera | DTUI | -16.15000000 | -67.65000000 | Barnabe et al. 2011 | Google earth | Province | I | I |
| *Triatoma infestans* | Vector | Hemiptera | DTUI | -16.15000000 | -67.65000000 | Barnabe et al. 2011 | Google earth | Province | I | I |
| *Triatoma infestans* | Vector | Hemiptera | DTUI | -16.15000000 | -67.65000000 | Barnabe et al. 2011 | Google earth | Province | I | I |
| *Triatoma infestans* | Vector | Hemiptera | DTUI | -16.40000000 | -71.53400000 | Barnabe et al. 2011 | Google earth | Province | I | I |
| *Triatoma infestans* | Vector | Hemiptera | DTUI | -16.40000000 | -71.53400000 | Barnabe et al. 2011 | Google earth | Province | I | I |
| *Triatoma infestans* | Vector | Hemiptera | DTUI | -16.40000000 | -71.53400000 | Barnabe et al. 2011 | Google earth | Province | I | I |
| *Triatoma infestans* | Vector | Hemiptera | DTUI | -16.40000000 | -71.53400000 | Barnabe et al. 2011 | Google earth | Province | I | I |
| *Triatoma infestans* | Vector | Hemiptera | DTUI | -16.40000000 | -71.53400000 | Barnabe et al. 2011 | Google earth | Province | I | I |
| *Triatoma infestans* | Vector | Hemiptera | DTUI | -16.40000000 | -71.53400000 | Barnabe et al. 2011 | Google earth | Province | I | I |
| *Triatoma infestans* | Vector | Hemiptera | DTUI | -16.408889 | -71.537222 | Cura et al. 2015 | Google earth | Locality | I | I |
| *Triatoma infestans* | Vector | Hemiptera | DTUI | -16.408889 | -71.537222 | Cura et al. 2015 | Google earth | Locality | I | I |
| *Triatoma infestans* | Vector | Hemiptera | DTUI | -16.408889 | -71.537222 | Cura et al. 2015 | Google earth | Locality | I | I |
| *Triatoma infestans* | Vector | Hemiptera | DTUI | -16.408889 | -71.537222 | Cura et al. 2015 | Google earth | Locality | I | I |
| *Triatoma infestans* | Vector | Hemiptera | DTUI | -16.408889 | -71.537222 | Cura et al. 2015 | Google earth | Locality | I | I |
| *Triatoma infestans* | Vector | Hemiptera | DTUI | -16.408889 | -71.537222 | Cura et al. 2015 | Google earth | Locality | I | I |
| *Triatoma infestans* | Vector | Hemiptera | DTUI | -16.408889 | -71.537222 | Cura et al. 2015 | Google earth | Locality | I | I |
| *Triatoma infestans* | Vector | Hemiptera | DTUI | -16.408889 | -71.537222 | Cura et al. 2015 | Google earth | Locality | I | I |
| *Triatoma infestans* | Vector | Hemiptera | DTUI | -16.408889 | -71.537222 | Cura et al. 2015 | Google earth | Locality | I | I |
| *Triatoma infestans* | Vector | Hemiptera | DTUII | -33.61700000 | -70.78300000 | Venegas et al. 2011 | Author | NA | II | II |
| *Triatoma infestans* | Vector | Hemiptera | DTUIII | -27.45000000 | -70.25000000 | Venegas et al. 2011 | Author | NA | II | III |
| *Triatoma infestans* | Vector | Hemiptera | DTUIII | -27.45000000 | -70.25000000 | Venegas et al. 2011 | Author | NA | II | III |
| *Triatoma infestans* | Vector | Hemiptera | DTUIII | -27.45000000 | -70.25000000 | Venegas et al. 2011 | Author | NA | II | III |
| *Triatoma infestans* | Vector | Hemiptera | DTUIII | -27.45000000 | -70.25000000 | Venegas et al. 2011 | Author | NA | II | III |
| *Triatoma infestans* | Vector | Hemiptera | DTUIII | -27.45000000 | -70.25000000 | Venegas et al. 2011 | Author | NA | II | III |
| *Triatoma infestans* | Vector | Hemiptera | DTUIII | -27.45000000 | -70.25000000 | Venegas et al. 2011 | Author | NA | II | III |
| *Triatoma infestans* | Vector | Hemiptera | DTUIII | -27.45000000 | -70.25000000 | Venegas et al. 2011 | Author | NA | II | III |
| *Triatoma infestans* | Vector | Hemiptera | DTUIII | -32.26700000 | -70.96700000 | Venegas et al. 2011 | Author | NA | II | III |
| *Triatoma infestans* | Vector | Hemiptera | DTUIII | -32.26700000 | -70.96700000 | Venegas et al. 2011 | Author | NA | II | III |
| *Triatoma infestans* | Vector | Hemiptera | DTUIII | -32.26700000 | -70.96700000 | Venegas et al. 2011 | Author | NA | II | III |
| *Triatoma infestans* | Vector | Hemiptera | DTUIII | -32.26700000 | -70.96700000 | Venegas et al. 2011 | Author | NA | II | III |
| *Triatoma infestans* | Vector | Hemiptera | DTUIII | -32.26700000 | -70.96700000 | Venegas et al. 2011 | Author | NA | II | III |
| *Triatoma infestans* | Vector | Hemiptera | DTUIII | -32.26700000 | -70.96700000 | Venegas et al. 2011 | Author | NA | II | III |
| *Triatoma infestans* | Vector | Hemiptera | DTUIII | -32.26700000 | -70.96700000 | Venegas et al. 2011 | Author | NA | II | III |
| *Triatoma infestans* | Vector | Hemiptera | DTUIII | -32.26700000 | -70.96700000 | Venegas et al. 2011 | Author | NA | II | III |
| *Triatoma infestans* | Vector | Hemiptera | DTUIII | -32.26700000 | -70.96700000 | Venegas et al. 2011 | Author | NA | II | III |
| *Triatoma infestans* | Vector | Hemiptera | DTUIII | -32.26700000 | -70.96700000 | Venegas et al. 2011 | Author | NA | II | III |
| *Triatoma infestans* | Vector | Hemiptera | DTUIII | -32.26700000 | -70.96700000 | Venegas et al. 2011 | Author | NA | II | III |
| *Triatoma infestans* | Vector | Hemiptera | DTUIII | -32.26700000 | -70.96700000 | Venegas et al. 2011 | Author | NA | II | III |
| *Triatoma infestans* | Vector | Hemiptera | DTUIII | -32.26700000 | -70.96700000 | Venegas et al. 2011 | Author | NA | II | III |
| *Triatoma infestans* | Vector | Hemiptera | DTUIII | -32.26700000 | -70.96700000 | Venegas et al. 2011 | Author | NA | II | III |
| *Triatoma infestans* | Vector | Hemiptera | DTUIII | -33.61700000 | -70.78300000 | Venegas et al. 2011 | Author | NA | II | III |
| *Triatoma infestans* | Vector | Hemiptera | DTUIII | -33.61700000 | -70.78300000 | Venegas et al. 2011 | Author | NA | II | III |
| *Triatoma infestans* | Vector | Hemiptera | DTUIII | -33.61700000 | -70.78300000 | Venegas et al. 2011 | Author | NA | II | III |
| *Triatoma infestans* | Vector | Hemiptera | DTUIII | -33.61700000 | -70.78300000 | Venegas et al. 2011 | Author | NA | II | III |
| *Triatoma infestans* | Vector | Hemiptera | DTUIII | -33.61700000 | -70.78300000 | Venegas et al. 2011 | Author | NA | II | III |
| *Triatoma infestans* | Vector | Hemiptera | DTUIII | -33.61700000 | -70.78300000 | Venegas et al. 2011 | Author | NA | II | III |
| *Triatoma infestans* | Vector | Hemiptera | DTUV | -17.939167 | -65.34194 | Barnabe et al. 2011 | Google earth | Community | II | ND |
| *Triatoma infestans* | Vector | Hemiptera | DTUV | -17.939167 | -65.34194 | Barnabe et al. 2011 | Google earth | Community | II | ND |
| *Triatoma infestans* | Vector | Hemiptera | DTUV | -17.939167 | -65.34194 | Barnabe et al. 2011 | Google earth | Community | II | ND |
| *Triatoma infestans* | Vector | Hemiptera | DTUV | -17.81670000 | -66.31670000 | Barnabe et al. 2011 | Google earth | Community | II | ND |
| *Triatoma infestans* | Vector | Hemiptera | DTUV | -17.81670000 | -66.31670000 | Barnabe et al. 2011 | Google earth | Community | II | ND |
| *Triatoma infestans* | Vector | Hemiptera | DTUV | -17.70000000 | -66.25000000 | Barnabe et al. 2011 | Google earth | Province | II | ND |
| *Triatoma infestans* | Vector | Hemiptera | DTUV | -17.70000000 | -66.25000000 | Barnabe et al. 2011 | Google earth | Province | II | ND |
| *Triatoma infestans* | Vector | Hemiptera | DTUV | -16.47638000 | -68.17583300 | Barnabe et al. 2011 | Google earth | Community | II | ND |
| *Triatoma infestans* | Vector | Hemiptera | DTUV | -15.67000000 | -68.16700000 | Barnabe et al. 2011 | Google earth | Community | II | ND |
| *Triatoma infestans* | Vector | Hemiptera | DTUV | -16.49610000 | -68.11940000 | Barnabe et al. 2011 | Google earth | Community | II | ND |
| *Triatoma infestans* | Vector | Hemiptera | DTUV | -16.55220000 | -68.07083000 | Barnabe et al. 2011 | Google earth | Community | II | ND |
| *Triatoma infestans* | Vector | Hemiptera | DTUV | -16.33440000 | -67.62083000 | Barnabe et al. 2011 | Google earth | Community | II | ND |
| *Triatoma infestans* | Vector | Hemiptera | DTUV | -16.33440000 | -67.62083000 | Barnabe et al. 2011 | Google earth | Community | II | ND |
| *Triatoma infestans* | Vector | Hemiptera | DTUV | -16.33440000 | -67.62083000 | Barnabe et al. 2011 | Google earth | Community | II | ND |
| *Triatoma infestans* | Vector | Hemiptera | DTUV | -16.46805000 | -68.17750000 | Barnabe et al. 2011 | Google earth | Community | II | ND |
| *Triatoma infestans* | Vector | Hemiptera | DTUV | -16.46805000 | -68.17750000 | Barnabe et al. 2011 | Google earth | Community | II | ND |
| *Triatoma infestans* | Vector | Hemiptera | DTUV | -16.46805000 | -68.17750000 | Barnabe et al. 2011 | Google earth | Community | II | ND |
| *Triatoma infestans* | Vector | Hemiptera | DTUV | -27.425556 | -59.024444 | Cura et al. 2015 | Google earth | Province | II | ND |
| *Triatoma infestans* | Vector | Hemiptera | DTUV | -27.425556 | -59.024444 | Cura et al. 2015 | Google earth | Province | II | ND |
| *Triatoma infestans* | Vector | Hemiptera | DTUI | -16.68900 | -68.01340 | Breniere et al. 2012 | Author | NA | I | I |
| *Triatoma infestans* | Vector | Hemiptera | DTUI | -16.68900 | -68.01340 | Breniere et al. 2012 | Author | NA | I | I |
| *Triatoma infestans* | Vector | Hemiptera | DTUI | -16.68900 | -68.01340 | Breniere et al. 2012 | Author | NA | I | I |
| *Triatoma infestans* | Vector | Hemiptera | DTUI | -16.68900 | -68.01340 | Breniere et al. 2012 | Author | NA | I | I |
| *Triatoma infestans* | Vector | Hemiptera | DTUI | -16.68900 | -68.01340 | Breniere et al. 2012 | Author | NA | I | I |
| *Triatoma infestans* | Vector | Hemiptera | DTUI | -16.70200 | -68.00450 | Breniere et al. 2012 | Author | NA | I | I |
| *Triatoma infestans* | Vector | Hemiptera | DTUI | -16.71190 | -67.99030 | Breniere et al. 2012 | Author | NA | I | I |
| *Triatoma infestans* | Vector | Hemiptera | DTUI | -16.71190 | -67.99030 | Breniere et al. 2012 | Author | NA | I | I |
| *Triatoma infestans* | Vector | Hemiptera | DTUI | -16.71190 | -67.99030 | Breniere et al. 2012 | Author | NA | I | I |
| *Triatoma infestans* | Vector | Hemiptera | DTUI | -16.71190 | -67.99030 | Breniere et al. 2012 | Author | NA | I | I |
| *Triatoma infestans* | Vector | Hemiptera | DTUI | -16.71190 | -67.99030 | Breniere et al. 2012 | Author | NA | I | I |
| *Triatoma infestans* | Vector | Hemiptera | DTUI | -16.71190 | -67.99030 | Breniere et al. 2012 | Author | NA | I | I |
| *Triatoma infestans* | Vector | Hemiptera | DTUI | -16.71190 | -67.99030 | Breniere et al. 2012 | Author | NA | I | I |
| *Triatoma infestans* | Vector | Hemiptera | DTUI | -16.71190 | -67.99030 | Breniere et al. 2012 | Author | NA | I | I |
| *Triatoma infestans* | Vector | Hemiptera | DTUI | -16.71190 | -67.99030 | Breniere et al. 2012 | Author | NA | I | I |
| *Triatoma infestans* | Vector | Hemiptera | DTUI | -16.71190 | -67.99030 | Breniere et al. 2012 | Author | NA | I | I |
| *Triatoma infestans* | Vector | Hemiptera | DTUI | -16.71190 | -67.99030 | Breniere et al. 2012 | Author | NA | I | I |
| *Triatoma infestans* | Vector | Hemiptera | DTUI | -16.71190 | -67.99030 | Breniere et al. 2012 | Author | NA | I | I |
| *Triatoma infestans* | Vector | Hemiptera | DTUI | -16.71190 | -67.99030 | Breniere et al. 2012 | Author | NA | I | I |
| *Triatoma infestans* | Vector | Hemiptera | DTUI | -16.71190 | -67.99030 | Breniere et al. 2012 | Author | NA | I | I |
| *Triatoma infestans* | Vector | Hemiptera | DTUI | -16.71190 | -67.99030 | Breniere et al. 2012 | Author | NA | I | I |
| *Triatoma infestans* | Vector | Hemiptera | DTUI | -16.71190 | -67.99030 | Breniere et al. 2012 | Author | NA | I | I |
| *Triatoma infestans* | Vector | Hemiptera | DTUI | -16.71190 | -67.99030 | Breniere et al. 2012 | Author | NA | I | I |
| *Triatoma infestans* | Vector | Hemiptera | DTUI | -16.71190 | -67.99030 | Breniere et al. 2012 | Author | NA | I | I |
| *Triatoma infestans* | Vector | Hemiptera | DTUI | -16.71190 | -67.99030 | Breniere et al. 2012 | Author | NA | I | I |
| *Triatoma infestans* | Vector | Hemiptera | DTUI | -16.71190 | -67.99030 | Breniere et al. 2012 | Author | NA | I | I |
| *Triatoma infestans* | Vector | Hemiptera | DTUI | -16.71190 | -67.99030 | Breniere et al. 2012 | Author | NA | I | I |
| *Triatoma infestans* | Vector | Hemiptera | DTUI | -16.71560 | -67.87040 | Breniere et al. 2012 | Author | NA | I | I |
| *Triatoma infestans* | Vector | Hemiptera | DTUI | -16.71560 | -67.87040 | Breniere et al. 2012 | Author | NA | I | I |
| *Triatoma infestans* | Vector | Hemiptera | DTUI | -16.71560 | -67.87040 | Breniere et al. 2012 | Author | NA | I | I |
| *Triatoma infestans* | Vector | Hemiptera | DTUI | -16.71560 | -67.87040 | Breniere et al. 2012 | Author | NA | I | I |
| *Triatoma infestans* | Vector | Hemiptera | DTUI | -16.71560 | -67.87040 | Breniere et al. 2012 | Author | NA | I | I |
| *Triatoma infestans* | Vector | Hemiptera | DTUI | -16.71900 | -67.87400 | Breniere et al. 2012 | Author | NA | I | I |
| *Triatoma infestans* | Vector | Hemiptera | DTUI | -16.71900 | -67.87400 | Breniere et al. 2012 | Author | NA | I | I |
| *Triatoma infestans* | Vector | Hemiptera | DTUI | -16.71900 | -67.87400 | Breniere et al. 2012 | Author | NA | I | I |
| *Triatoma infestans* | Vector | Hemiptera | DTUI | -16.71900 | -67.87400 | Breniere et al. 2012 | Author | NA | I | I |
| *Triatoma infestans* | Vector | Hemiptera | DTUI | -16.71900 | -67.87400 | Breniere et al. 2012 | Author | NA | I | I |
| *Triatoma infestans* | Vector | Hemiptera | DTUI | -16.71900 | -67.87400 | Breniere et al. 2012 | Author | NA | I | I |
| *Triatoma infestans* | Vector | Hemiptera | DTUI | -16.71900 | -67.87400 | Breniere et al. 2012 | Author | NA | I | I |
| *Triatoma infestans* | Vector | Hemiptera | DTUI | -16.71900 | -67.87400 | Breniere et al. 2012 | Author | NA | I | I |
| *Triatoma infestans* | Vector | Hemiptera | DTUI | -16.71900 | -67.87400 | Breniere et al. 2012 | Author | NA | I | I |
| *Triatoma infestans* | Vector | Hemiptera | DTUI | -16.81400 | -67.70500 | Breniere et al. 2012 | Author | NA | I | I |
| *Triatoma infestans* | Vector | Hemiptera | DTUI | -16.81400 | -67.70500 | Breniere et al. 2012 | Author | NA | I | I |
| *Triatoma infestans* | Vector | Hemiptera | DTUI | -16.81400 | -67.70500 | Breniere et al. 2012 | Author | NA | I | I |
| *Triatoma infestans* | Vector | Hemiptera | DTUI | -16.81400 | -67.70500 | Breniere et al. 2012 | Author | NA | I | I |
| *Triatoma infestans* | Vector | Hemiptera | DTUI | -16.81400 | -67.70500 | Breniere et al. 2012 | Author | NA | I | I |
| *Triatoma infestans* | Vector | Hemiptera | DTUI | -16.81400 | -67.70500 | Breniere et al. 2012 | Author | NA | I | I |
| *Triatoma infestans* | Vector | Hemiptera | DTUI | -16.81400 | -67.70500 | Breniere et al. 2012 | Author | NA | I | I |
| *Triatoma infestans* | Vector | Hemiptera | DTUI | -16.81400 | -67.70500 | Breniere et al. 2012 | Author | NA | I | I |
| *Triatoma infestans* | Vector | Hemiptera | DTUI | -16.81400 | -67.70500 | Breniere et al. 2012 | Author | NA | I | I |
| *Triatoma infestans* | Vector | Hemiptera | DTUI | -16.81400 | -67.70500 | Breniere et al. 2012 | Author | NA | I | I |
| *Triatoma infestans* | Vector | Hemiptera | DTUI | -16.81400 | -67.70500 | Breniere et al. 2012 | Author | NA | I | I |
| *Triatoma infestans* | Vector | Hemiptera | DTUI | -16.81400 | -67.70500 | Breniere et al. 2012 | Author | NA | I | I |
| *Triatoma infestans* | Vector | Hemiptera | DTUI | -16.81400 | -67.70500 | Breniere et al. 2012 | Author | NA | I | I |
| *Triatoma infestans* | Vector | Hemiptera | DTUI | -16.81400 | -67.70500 | Breniere et al. 2012 | Author | NA | I | I |
| *Triatoma infestans* | Vector | Hemiptera | DTUI | -16.81400 | -67.70500 | Breniere et al. 2012 | Author | NA | I | I |
| *Triatoma infestans* | Vector | Hemiptera | DTUI | -16.81400 | -67.70500 | Breniere et al. 2012 | Author | NA | I | I |
| *Triatoma infestans* | Vector | Hemiptera | DTUI | -16.81400 | -67.70500 | Breniere et al. 2012 | Author | NA | I | I |
| *Triatoma infestans* | Vector | Hemiptera | DTUI | -16.81400 | -67.70500 | Breniere et al. 2012 | Author | NA | I | I |
| *Triatoma infestans* | Vector | Hemiptera | DTUI | -16.81400 | -67.70500 | Breniere et al. 2012 | Author | NA | I | I |
| *Triatoma infestans* | Vector | Hemiptera | DTUI | -16.82860 | -67.70470 | Breniere et al. 2012 | Author | NA | I | I |
| *Triatoma infestans* | Vector | Hemiptera | DTUI | -16.82860 | -67.70470 | Breniere et al. 2012 | Author | NA | I | I |
| *Triatoma infestans* | Vector | Hemiptera | DTUI | -16.82860 | -67.70470 | Breniere et al. 2012 | Author | NA | I | I |
| *Triatoma infestans* | Vector | Hemiptera | DTUI | -16.82860 | -67.70470 | Breniere et al. 2012 | Author | NA | I | I |
| *Triatoma infestans* | Vector | Hemiptera | DTUI | -16.82860 | -67.70470 | Breniere et al. 2012 | Author | NA | I | I |
| *Triatoma infestans* | Vector | Hemiptera | DTUI | -16.82860 | -67.70470 | Breniere et al. 2012 | Author | NA | I | I |
| *Triatoma infestans* | Vector | Hemiptera | DTUI | -16.82860 | -67.70470 | Breniere et al. 2012 | Author | NA | I | I |
| *Triatoma infestans* | Vector | Hemiptera | DTUI | -16.82860 | -67.70470 | Breniere et al. 2012 | Author | NA | I | I |
| *Triatoma infestans* | Vector | Hemiptera | DTUI | -16.82860 | -67.70470 | Breniere et al. 2012 | Author | NA | I | I |
| *Triatoma infestans* | Vector | Hemiptera | DTUI | -16.82860 | -67.70470 | Breniere et al. 2012 | Author | NA | I | I |
| *Triatoma infestans* | Vector | Hemiptera | DTUI | -16.82860 | -67.70470 | Breniere et al. 2012 | Author | NA | I | I |
| *Triatoma infestans* | Vector | Hemiptera | DTUI | -16.82860 | -67.70470 | Breniere et al. 2012 | Author | NA | I | I |
| *Triatoma infestans* | Vector | Hemiptera | DTUI | -16.82860 | -67.70470 | Breniere et al. 2012 | Author | NA | I | I |
| *Triatoma infestans* | Vector | Hemiptera | DTUI | -16.82860 | -67.70470 | Breniere et al. 2012 | Author | NA | I | I |
| *Triatoma infestans* | Vector | Hemiptera | DTUI | -16.82860 | -67.70470 | Breniere et al. 2012 | Author | NA | I | I |
| *Triatoma infestans* | Vector | Hemiptera | DTUI | -16.82860 | -67.70470 | Breniere et al. 2012 | Author | NA | I | I |
| *Triatoma infestans* | Vector | Hemiptera | DTUI | -16.82860 | -67.70470 | Breniere et al. 2012 | Author | NA | I | I |
| *Triatoma infestans* | Vector | Hemiptera | DTUI | -16.82860 | -67.70470 | Breniere et al. 2012 | Author | NA | I | I |
| *Triatoma infestans* | Vector | Hemiptera | DTUI | -16.82860 | -67.70470 | Breniere et al. 2012 | Author | NA | I | I |
| *Triatoma infestans* | Vector | Hemiptera | DTUI | -16.88670 | -67.71190 | Breniere et al. 2012 | Author | NA | I | I |
| *Triatoma infestans* | Vector | Hemiptera | DTUI | -16.88670 | -67.71190 | Breniere et al. 2012 | Author | NA | I | I |
| *Triatoma infestans* | Vector | Hemiptera | DTUI | -16.88670 | -67.71190 | Breniere et al. 2012 | Author | NA | I | I |
| *Triatoma infestans* | Vector | Hemiptera | DTUI | -16.88670 | -67.71190 | Breniere et al. 2012 | Author | NA | I | I |
| *Triatoma infestans* | Vector | Hemiptera | DTUI | -16.88670 | -67.71190 | Breniere et al. 2012 | Author | NA | I | I |
| *Triatoma infestans* | Vector | Hemiptera | DTUI | -16.88670 | -67.71190 | Breniere et al. 2012 | Author | NA | I | I |
| *Triatoma infestans* | Vector | Hemiptera | DTUI | -16.88670 | -67.71190 | Breniere et al. 2012 | Author | NA | I | I |
| *Triatoma infestans* | Vector | Hemiptera | DTUI | -16.88670 | -67.71190 | Breniere et al. 2012 | Author | NA | I | I |
| *Triatoma infestans* | Vector | Hemiptera | DTUI | -16.88670 | -67.71190 | Breniere et al. 2012 | Author | NA | I | I |
| *Triatoma infestans* | Vector | Hemiptera | DTUI | -16.88670 | -67.71190 | Breniere et al. 2012 | Author | NA | I | I |
| *Triatoma infestans* | Vector | Hemiptera | DTUI | -16.88670 | -67.71190 | Breniere et al. 2012 | Author | NA | I | I |
| *Triatoma infestans* | Vector | Hemiptera | DTUI | -16.88670 | -67.71190 | Breniere et al. 2012 | Author | NA | I | I |
| *Triatoma infestans* | Vector | Hemiptera | DTUI | -16.88670 | -67.71190 | Breniere et al. 2012 | Author | NA | I | I |
| *Triatoma infestans* | Vector | Hemiptera | DTUI | -16.93020 | -67.69240 | Breniere et al. 2012 | Author | NA | I | I |
| *Triatoma infestans* | Vector | Hemiptera | DTUI | -16.93020 | -67.69240 | Breniere et al. 2012 | Author | NA | I | I |
| *Triatoma infestans* | Vector | Hemiptera | DTUI | -16.93020 | -67.69240 | Breniere et al. 2012 | Author | NA | I | I |
| *Triatoma infestans* | Vector | Hemiptera | DTUI | -16.93020 | -67.69240 | Breniere et al. 2012 | Author | NA | I | I |
| *Triatoma infestans* | Vector | Hemiptera | DTUI | -16.93020 | -67.69240 | Breniere et al. 2012 | Author | NA | I | I |
| *Triatoma infestans* | Vector | Hemiptera | DTUI | -16.93020 | -67.69240 | Breniere et al. 2012 | Author | NA | I | I |
| *Triatoma infestans* | Vector | Hemiptera | DTUI | -16.93020 | -67.69240 | Breniere et al. 2012 | Author | NA | I | I |
| *Triatoma infestans* | Vector | Hemiptera | DTUI | -16.93020 | -67.69240 | Breniere et al. 2012 | Author | NA | I | I |
| *Triatoma infestans* | Vector | Hemiptera | DTUI | -16.93020 | -67.69240 | Breniere et al. 2012 | Author | NA | I | I |
| *Triatoma infestans* | Vector | Hemiptera | DTUI | -16.93020 | -67.69240 | Breniere et al. 2012 | Author | NA | I | I |
| *Triatoma infestans* | Vector | Hemiptera | DTUI | -16.93020 | -67.69240 | Breniere et al. 2012 | Author | NA | I | I |
| *Triatoma infestans* | Vector | Hemiptera | DTUI | -16.93020 | -67.69240 | Breniere et al. 2012 | Author | NA | I | I |
| *Triatoma infestans* | Vector | Hemiptera | DTUI | -16.93020 | -67.69240 | Breniere et al. 2012 | Author | NA | I | I |
| *Triatoma infestans* | Vector | Hemiptera | DTUI | -16.93020 | -67.69240 | Breniere et al. 2012 | Author | NA | I | I |
| *Triatoma infestans* | Vector | Hemiptera | DTUI | -16.93020 | -67.69240 | Breniere et al. 2012 | Author | NA | I | I |
| *Triatoma infestans* | Vector | Hemiptera | DTUI | -16.93020 | -67.69240 | Breniere et al. 2012 | Author | NA | I | I |
| *Triatoma infestans* | Vector | Hemiptera | DTUI | -17.00830 | -67.65700 | Breniere et al. 2012 | Author | NA | I | I |
| *Triatoma infestans* | Vector | Hemiptera | DTUI | -17.00830 | -67.65700 | Breniere et al. 2012 | Author | NA | I | I |
| *Triatoma infestans* | Vector | Hemiptera | DTUI | -17.00830 | -67.65700 | Breniere et al. 2012 | Author | NA | I | I |
| *Triatoma infestans* | Vector | Hemiptera | DTUI | -17.00830 | -67.65700 | Breniere et al. 2012 | Author | NA | I | I |
| *Triatoma infestans* | Vector | Hemiptera | DTUI | -17.00830 | -67.65700 | Breniere et al. 2012 | Author | NA | I | I |
| *Triatoma infestans* | Vector | Hemiptera | DTUI | -17.00830 | -67.65700 | Breniere et al. 2012 | Author | NA | I | I |
| *Triatoma infestans* | Vector | Hemiptera | DTUI | -17.00830 | -67.65700 | Breniere et al. 2012 | Author | NA | I | I |
| *Triatoma infestans* | Vector | Hemiptera | DTUI | -17.00830 | -67.65700 | Breniere et al. 2012 | Author | NA | I | I |
| *Triatoma infestans* | Vector | Hemiptera | DTUI | -17.00830 | -67.65700 | Breniere et al. 2012 | Author | NA | I | I |
| *Triatoma infestans* | Vector | Hemiptera | DTUI | -17.00830 | -67.65700 | Breniere et al. 2012 | Author | NA | I | I |
| *Triatoma infestans* | Vector | Hemiptera | DTUI | -17.00830 | -67.65700 | Breniere et al. 2012 | Author | NA | I | I |
| *Triatoma infestans* | Vector | Hemiptera | DTUI | -17.00830 | -67.65700 | Breniere et al. 2012 | Author | NA | I | I |
| *Triatoma infestans* | Vector | Hemiptera | DTUI | -17.00830 | -67.65700 | Breniere et al. 2012 | Author | NA | I | I |
| *Triatoma infestans* | Vector | Hemiptera | DTUI | -17.00830 | -67.65700 | Breniere et al. 2012 | Author | NA | I | I |
| *Triatoma infestans* | Vector | Hemiptera | DTUI | -17.00830 | -67.65700 | Breniere et al. 2012 | Author | NA | I | I |
| *Triatoma infestans* | Vector | Hemiptera | DTUI | -17.00830 | -67.65700 | Breniere et al. 2012 | Author | NA | I | I |
| *Triatoma infestans* | Vector | Hemiptera | DTUI | -17.00830 | -67.65700 | Breniere et al. 2012 | Author | NA | I | I |
| *Triatoma infestans* | Vector | Hemiptera | DTUI | -17.00830 | -67.65700 | Breniere et al. 2012 | Author | NA | I | I |
| *Triatoma infestans* | Vector | Hemiptera | DTUI | -17.00830 | -67.65700 | Breniere et al. 2012 | Author | NA | I | I |
| *Triatoma infestans* | Vector | Hemiptera | DTUI | -17.00830 | -67.65700 | Breniere et al. 2012 | Author | NA | I | I |
| *Triatoma infestans* | Vector | Hemiptera | DTUI | -17.00830 | -67.65700 | Breniere et al. 2012 | Author | NA | I | I |
| *Triatoma infestans* | Vector | Hemiptera | DTUI | -17.00830 | -67.65700 | Breniere et al. 2012 | Author | NA | I | I |
| *Triatoma infestans* | Vector | Hemiptera | DTUI | -17.00830 | -67.65700 | Breniere et al. 2012 | Author | NA | I | I |
| *Triatoma infestans* | Vector | Hemiptera | DTUI | -17.00830 | -67.65700 | Breniere et al. 2012 | Author | NA | I | I |
| *Triatoma infestans* | Vector | Hemiptera | DTUI | -17.00830 | -67.65700 | Breniere et al. 2012 | Author | NA | I | I |
| *Triatoma infestans* | Vector | Hemiptera | DTUI | -17.00830 | -67.65700 | Breniere et al. 2012 | Author | NA | I | I |
| *Triatoma infestans* | Vector | Hemiptera | DTUI | -17.00830 | -67.65700 | Breniere et al. 2012 | Author | NA | I | I |
| *Triatoma infestans* | Vector | Hemiptera | DTUI | -17.00830 | -67.65700 | Breniere et al. 2012 | Author | NA | I | I |
| *Triatoma infestans* | Vector | Hemiptera | DTUI | -17.00830 | -67.65700 | Breniere et al. 2012 | Author | NA | I | I |
| *Triatoma infestans* | Vector | Hemiptera | DTUI | -17.00830 | -67.65700 | Breniere et al. 2012 | Author | NA | I | I |
| *Triatoma infestans* | Vector | Hemiptera | DTUI | -17.00830 | -67.65700 | Breniere et al. 2012 | Author | NA | I | I |
| *Triatoma infestans* | Vector | Hemiptera | DTUI | -17.00830 | -67.65700 | Breniere et al. 2012 | Author | NA | I | I |
| *Triatoma infestans* | Vector | Hemiptera | DTUI | -17.00830 | -67.65700 | Breniere et al. 2012 | Author | NA | I | I |
| *Triatoma infestans* | Vector | Hemiptera | DTUI | -17.00830 | -67.65700 | Breniere et al. 2012 | Author | NA | I | I |
| *Triatoma infestans* | Vector | Hemiptera | DTUI | -17.00830 | -67.65700 | Breniere et al. 2012 | Author | NA | I | I |
| *Triatoma infestans* | Vector | Hemiptera | DTUI | -17.00830 | -67.65700 | Breniere et al. 2012 | Author | NA | I | I |
| *Triatoma infestans* | Vector | Hemiptera | DTUI | -17.00830 | -67.65700 | Breniere et al. 2012 | Author | NA | I | I |
| *Triatoma infestans* | Vector | Hemiptera | DTUI | -17.00830 | -67.65700 | Breniere et al. 2012 | Author | NA | I | I |
| *Triatoma infestans* | Vector | Hemiptera | DTUI | -17.00830 | -67.65700 | Breniere et al. 2012 | Author | NA | I | I |
| *Triatoma infestans* | Vector | Hemiptera | DTUI | -17.00830 | -67.65700 | Breniere et al. 2012 | Author | NA | I | I |
| *Triatoma infestans* | Vector | Hemiptera | DTUI | -17.00830 | -67.65700 | Breniere et al. 2012 | Author | NA | I | I |
| *Triatoma infestans* | Vector | Hemiptera | DTUI | -17.00830 | -67.65700 | Breniere et al. 2012 | Author | NA | I | I |
| *Triatoma infestans* | Vector | Hemiptera | DTUI | -17.00830 | -67.65700 | Breniere et al. 2012 | Author | NA | I | I |
| *Triatoma infestans* | Vector | Hemiptera | DTUI | -17.03180 | -67.67730 | Breniere et al. 2012 | Author | NA | I | I |
| *Triatoma infestans* | Vector | Hemiptera | DTUI | -17.06500 | -67.66420 | Breniere et al. 2012 | Author | NA | I | I |
| *Triatoma infestans* | Vector | Hemiptera | DTUI | -17.06500 | -67.66420 | Breniere et al. 2012 | Author | NA | I | I |
| *Triatoma infestans* | Vector | Hemiptera | DTUI | -17.06500 | -67.66420 | Breniere et al. 2012 | Author | NA | I | I |
| *Triatoma infestans* | Vector | Hemiptera | DTUI | -17.06500 | -67.66420 | Breniere et al. 2012 | Author | NA | I | I |
| *Triatoma infestans* | Vector | Hemiptera | DTUI | -17.06500 | -67.66420 | Breniere et al. 2012 | Author | NA | I | I |
| *Triatoma infestans* | Vector | Hemiptera | DTUI | -17.06800 | -67.65700 | Breniere et al. 2012 | Author | NA | I | I |
| *Triatoma infestans* | Vector | Hemiptera | DTUI | -17.06800 | -67.65700 | Breniere et al. 2012 | Author | NA | I | I |
| *Triatoma infestans* | Vector | Hemiptera | DTUI | -17.06800 | -67.65700 | Breniere et al. 2012 | Author | NA | I | I |
| *Triatoma infestans* | Vector | Hemiptera | DTUI | -17.06800 | -67.65700 | Breniere et al. 2012 | Author | NA | I | I |
| *Triatoma infestans* | Vector | Hemiptera | DTUI | -17.06800 | -67.65700 | Breniere et al. 2012 | Author | NA | I | I |
| *Triatoma infestans* | Vector | Hemiptera | DTUI | -17.07330 | -67.64510 | Breniere et al. 2012 | Author | NA | I | I |
| *Triatoma infestans* | Vector | Hemiptera | DTUI | -17.07360 | -67.63320 | Breniere et al. 2012 | Author | NA | I | I |
| *Triatoma infestans* | Vector | Hemiptera | DTUI | -17.07360 | -67.63320 | Breniere et al. 2012 | Author | NA | I | I |
| *Triatoma infestans* | Vector | Hemiptera | DTUI | -17.07360 | -67.63320 | Breniere et al. 2012 | Author | NA | I | I |
| *Triatoma infestans* | Vector | Hemiptera | DTUI | -17.07360 | -67.63320 | Breniere et al. 2012 | Author | NA | I | I |
| *Triatoma infestans* | Vector | Hemiptera | DTUI | -17.07360 | -67.63320 | Breniere et al. 2012 | Author | NA | I | I |
| *Triatoma infestans* | Vector | Hemiptera | DTUI | -17.07900 | -67.63250 | Breniere et al. 2012 | Author | NA | I | I |
| *Triatoma infestans* | Vector | Hemiptera | DTUI | -17.07900 | -67.63250 | Breniere et al. 2012 | Author | NA | I | I |
| *Triatoma infestans* | Vector | Hemiptera | DTUI | -17.07900 | -67.63250 | Breniere et al. 2012 | Author | NA | I | I |
| *Triatoma infestans* | Vector | Hemiptera | DTUI | -17.07900 | -67.63250 | Breniere et al. 2012 | Author | NA | I | I |
| *Triatoma infestans* | Vector | Hemiptera | DTUI | -17.07900 | -67.63250 | Breniere et al. 2012 | Author | NA | I | I |
| *Triatoma infestans* | Vector | Hemiptera | DTUI | -17.07900 | -67.63250 | Breniere et al. 2012 | Author | NA | I | I |
| *Triatoma infestans* | Vector | Hemiptera | DTUI | -17.07900 | -67.63250 | Breniere et al. 2012 | Author | NA | I | I |
| *Triatoma infestans* | Vector | Hemiptera | DTUI | -17.07900 | -67.63250 | Breniere et al. 2012 | Author | NA | I | I |
| *Triatoma infestans* | Vector | Hemiptera | DTUI | -17.07900 | -67.63250 | Breniere et al. 2012 | Author | NA | I | I |
| *Triatoma infestans* | Vector | Hemiptera | DTUI | -17.07900 | -67.63250 | Breniere et al. 2012 | Author | NA | I | I |
| *Triatoma infestans* | Vector | Hemiptera | DTUI | -17.07900 | -67.63250 | Breniere et al. 2012 | Author | NA | I | I |
| *Triatoma infestans* | Vector | Hemiptera | DTUI | -17.07900 | -67.63250 | Breniere et al. 2012 | Author | NA | I | I |
| *Triatoma infestans* | Vector | Hemiptera | DTUI | -17.07900 | -67.63250 | Breniere et al. 2012 | Author | NA | I | I |
| *Triatoma infestans* | Vector | Hemiptera | DTUI | -17.07900 | -67.63250 | Breniere et al. 2012 | Author | NA | I | I |
| *Triatoma infestans* | Vector | Hemiptera | DTUI | -17.07900 | -67.63250 | Breniere et al. 2012 | Author | NA | I | I |
| *Triatoma infestans* | Vector | Hemiptera | DTUI | -17.07900 | -67.63250 | Breniere et al. 2012 | Author | NA | I | I |
| *Triatoma infestans* | Vector | Hemiptera | DTUI | -17.12550 | -67.59980 | Breniere et al. 2012 | Author | NA | I | I |
| *Triatoma infestans* | Vector | Hemiptera | DTUI | -17.12550 | -67.59980 | Breniere et al. 2012 | Author | NA | I | I |
| *Triatoma infestans* | Vector | Hemiptera | DTUI | -17.12550 | -67.59980 | Breniere et al. 2012 | Author | NA | I | I |
| *Triatoma infestans* | Vector | Hemiptera | DTUI | -17.13630 | -67.58830 | Breniere et al. 2012 | Author | NA | I | I |
| *Triatoma infestans* | Vector | Hemiptera | DTUI | -17.13630 | -67.58830 | Breniere et al. 2012 | Author | NA | I | I |
| *Triatoma infestans* | Vector | Hemiptera | DTUI | -17.13630 | -67.58830 | Breniere et al. 2012 | Author | NA | I | I |
| *Triatoma infestans* | Vector | Hemiptera | DTUI | -17.13630 | -67.58830 | Breniere et al. 2012 | Author | NA | I | I |
| *Triatoma infestans* | Vector | Hemiptera | DTUI | -17.71250 | -66.49410 | Breniere et al. 2012 | Author | NA | I | I |
| *Triatoma infestans* | Vector | Hemiptera | DTUI | -17.71250 | -66.49410 | Breniere et al. 2012 | Author | NA | I | I |
| *Triatoma infestans* | Vector | Hemiptera | DTUI | -17.71250 | -66.49410 | Breniere et al. 2012 | Author | NA | I | I |
| *Triatoma infestans* | Vector | Hemiptera | DTUI | -17.71250 | -66.49410 | Breniere et al. 2012 | Author | NA | I | I |
| *Triatoma infestans* | Vector | Hemiptera | DTUI | -17.71250 | -66.49410 | Breniere et al. 2012 | Author | NA | I | I |
| *Triatoma infestans* | Vector | Hemiptera | DTUI | -17.71250 | -66.49410 | Breniere et al. 2012 | Author | NA | I | I |
| *Triatoma infestans* | Vector | Hemiptera | DTUI | -17.71250 | -66.49410 | Breniere et al. 2012 | Author | NA | I | I |
| *Triatoma infestans* | Vector | Hemiptera | DTUI | -17.71250 | -66.49410 | Breniere et al. 2012 | Author | NA | I | I |
| *Triatoma infestans* | Vector | Hemiptera | DTUI | -17.71250 | -66.49410 | Breniere et al. 2012 | Author | NA | I | I |
| *Triatoma infestans* | Vector | Hemiptera | DTUI | -17.42750 | -66.25890 | Breniere et al. 2012 | Author | NA | I | I |
| *Triatoma infestans* | Vector | Hemiptera | DTUI | -17.42750 | -66.25890 | Breniere et al. 2012 | Author | NA | I | I |
| *Triatoma infestans* | Vector | Hemiptera | DTUI | -17.42750 | -66.25890 | Breniere et al. 2012 | Author | NA | I | I |
| *Triatoma infestans* | Vector | Hemiptera | DTUI | -17.42750 | -66.25890 | Breniere et al. 2012 | Author | NA | I | I |
| *Triatoma infestans* | Vector | Hemiptera | DTUI | -17.42750 | -66.25890 | Breniere et al. 2012 | Author | NA | I | I |
| *Triatoma infestans* | Vector | Hemiptera | DTUI | -17.42750 | -66.25890 | Breniere et al. 2012 | Author | NA | I | I |
| *Triatoma infestans* | Vector | Hemiptera | DTUI | -17.42750 | -66.25890 | Breniere et al. 2012 | Author | NA | I | I |
| *Triatoma infestans* | Vector | Hemiptera | DTUI | -17.42750 | -66.25890 | Breniere et al. 2012 | Author | NA | I | I |
| *Triatoma infestans* | Vector | Hemiptera | DTUI | -17.42750 | -66.25890 | Breniere et al. 2012 | Author | NA | I | I |
| *Triatoma infestans* | Vector | Hemiptera | DTUI | -17.42750 | -66.25890 | Breniere et al. 2012 | Author | NA | I | I |
| *Triatoma infestans* | Vector | Hemiptera | DTUI | -17.42750 | -66.25890 | Breniere et al. 2012 | Author | NA | I | I |
| *Triatoma infestans* | Vector | Hemiptera | DTUI | -17.46260 | -66.31410 | Breniere et al. 2012 | Author | NA | I | I |
| *Triatoma infestans* | Vector | Hemiptera | DTUI | -17.46260 | -66.31410 | Breniere et al. 2012 | Author | NA | I | I |
| *Triatoma infestans* | Vector | Hemiptera | DTUI | -17.46260 | -66.31410 | Breniere et al. 2012 | Author | NA | I | I |
| *Triatoma infestans* | Vector | Hemiptera | DTUI | -17.47700 | -66.13780 | Breniere et al. 2012 | Author | NA | I | I |
| *Triatoma infestans* | Vector | Hemiptera | DTUI | -17.47700 | -66.13780 | Breniere et al. 2012 | Author | NA | I | I |
| *Triatoma infestans* | Vector | Hemiptera | DTUI | -17.47700 | -66.13780 | Breniere et al. 2012 | Author | NA | I | I |
| *Triatoma infestans* | Vector | Hemiptera | DTUI | -17.47700 | -66.13780 | Breniere et al. 2012 | Author | NA | I | I |
| *Triatoma infestans* | Vector | Hemiptera | ND | 17.98780 | -65.08640 | Breniere et al. 2012 | Author | NA | II | ND |
| *Triatoma infestans* | Vector | Hemiptera | DTUI | -18.01230 | -65.80900 | Breniere et al. 2012 | Author | NA | I | I |
| *Triatoma infestans* | Vector | Hemiptera | DTUI | -18.01230 | -65.80900 | Breniere et al. 2012 | Author | NA | I | I |
| *Triatoma infestans* | Vector | Hemiptera | DTUI | -18.01230 | -65.80900 | Breniere et al. 2012 | Author | NA | I | I |
| *Triatoma infestans* | Vector | Hemiptera | ND | -18.03080 | -65.78850 | Breniere et al. 2012 | Author | NA | II | ND |
| *Triatoma infestans* | Vector | Hemiptera | DTUI | -21.62130 | -65.81270 | Breniere et al. 2012 | Author | NA | I | I |
| *Triatoma infestans* | Vector | Hemiptera | DTUI | -21.62130 | -65.81270 | Breniere et al. 2012 | Author | NA | I | I |
| *Triatoma infestans* | Vector | Hemiptera | DTUI | -21.62130 | -65.81270 | Breniere et al. 2012 | Author | NA | I | I |
| *Triatoma infestans* | Vector | Hemiptera | DTUI | -21.62130 | -65.81270 | Breniere et al. 2012 | Author | NA | I | I |
| *Triatoma infestans* | Vector | Hemiptera | DTUI | -21.62130 | -65.81270 | Breniere et al. 2012 | Author | NA | I | I |
| *Triatoma infestans* | Vector | Hemiptera | DTUI | -21.62130 | -65.81270 | Breniere et al. 2012 | Author | NA | I | I |
| *Triatoma infestans* | Vector | Hemiptera | DTUI | -21.62130 | -65.81270 | Breniere et al. 2012 | Author | NA | I | I |
| *Triatoma infestans* | Vector | Hemiptera | ND | -18.59790 | -65.12590 | Breniere et al. 2012 | Author | NA | II | ND |
| *Triatoma infestans* | Vector | Hemiptera | DTUI | -19.92750 | -63.90240 | Breniere et al. 2012 | Author | NA | I | I |
| *Triatoma infestans* | Vector | Hemiptera | ND | -20.18610 | -64.02200 | Breniere et al. 2012 | Author | NA | II | ND |
| *Triatoma infestans* | Vector | Hemiptera | DTUI | -21.37690 | -63.35960 | Breniere et al. 2012 | Author | NA | I | I |
| *Triatoma lecticularia* | Vector | Hemiptera | DTUIV | 31.96833333 | -99.90166667 | Cura et al. 2015 | Google earth | Province | II | III |
| *Triatoma longipennis* | Vector | Hemiptera | DTUI | 19.52666667 | -105.07361111 | Cura et al. 2015 | Google earth | Locality | I | I |
| *Triatoma maculata* | Vector | Hemiptera | DTUI | 8.58300000 | -63.95000000 | Carrasco et al. 2012 | Google earth | State | I | I |
| *Triatoma maculata* | Vector | Hemiptera | DTUI | 8.58300000 | -63.95000000 | Carrasco et al. 2012 | Google earth | State | I | I |
| *Triatoma maculata* | Vector | Hemiptera | DTUI | 8.58300000 | -63.95000000 | Carrasco et al. 2012 | Google earth | State | I | I |
| *Triatoma maculata* | Vector | Hemiptera | DTUI | 8.58300000 | -63.95000000 | Carrasco et al. 2012 | Google earth | State | I | I |
| *Triatoma maculata* | Vector | Hemiptera | DTUI | 8.58300000 | -63.95000000 | Carrasco et al. 2012 | Google earth | State | I | I |
| *Triatoma maculata* | Vector | Hemiptera | DTUI | 8.58300000 | -63.95000000 | Carrasco et al. 2012 | Google earth | State | I | I |
| *Triatoma maculata* | Vector | Hemiptera | DTUI | 8.58300000 | -63.95000000 | Carrasco et al. 2012 | Google earth | State | I | I |
| *Triatoma maculata* | Vector | Hemiptera | DTUI | 8.58300000 | -63.95000000 | Carrasco et al. 2012 | Google earth | State | I | I |
| *Triatoma maculata* | Vector | Hemiptera | DTUI | 8.30000000 | -70.05000000 | Carrasco et al. 2012 | Google earth | State | I | I |
| *Triatoma maculata* | Vector | Hemiptera | DTUI | 8.30000000 | -70.05000000 | Carrasco et al. 2012 | Google earth | State | I | I |
| *Triatoma maculata* | Vector | Hemiptera | DTUI | 8.73400000 | -66.23400000 | Carrasco et al. 2012 | Google earth | State | I | I |
| *Triatoma maculata* | Vector | Hemiptera | DTUI | 10.40000000 | -63.28300000 | Carrasco et al. 2012 | Google earth | State | I | I |
| *Triatoma maculata* | Vector | Hemiptera | DTUI | 10.40000000 | -63.28300000 | Carrasco et al. 2012 | Google earth | State | I | I |
| *Triatoma maculata* | Vector | Hemiptera | DTUI | 8.37000000 | -70.51000000 | Llewellyn et al. 2009b | Author | NA | I | I |
| *Triatoma maculata* | Vector | Hemiptera | DTUI | 10.36000000 | -66.75000000 | Llewellyn et al. 2009b | Author | NA | I | I |
| *Triatoma maculata* | Vector | Hemiptera | DTUI | 10.04000000 | -64.32000000 | Llewellyn et al. 2009b | Author | NA | I | I |
| *Triatoma nigromaculata* | Vector | Hemiptera | DTUI | 10.25000000 | -66.41670000 | Carrasco et al. 2012 | Google earth | State | I | I |
| *Triatoma nitida* | Vector | Hemiptera | DTUI | 14.60000000 | -90.53300000 | Higo et al. 2004 | Google earth | Department/State | I | I |
| *Triatoma Pallidipennis* | Vector | Hemiptera | DTUI | 20.95000000 | -89.61670000 | Higo et al. 2004 | Google earth | Department/State | I | I |
| *Triatoma Pallidipennis* | Vector | Hemiptera | DTUI | 20.95000000 | -89.61670000 | Higo et al. 2004 | Google earth | Department/State | I | I |
| *Triatoma Pallidipennis* | Vector | Hemiptera | DTUI | 20.65000000 | -103.33000000 | Higo et al. 2004 | Google earth | Department/State | I | I |
| *Triatoma Pallidipennis* | Vector | Hemiptera | DTUI | 18.95300000 | -99.22300000 | Zumaya-Estrada et al. 2012 | Author | NA | I | I |
| *Triatoma phyllosoma* | Vector | Hemiptera | DTUI | 16.18333333 | -95.20861111 | Cura et al. 2015 | Google earth | Locality | I | I |
| *Triatoma phyllosoma* | Vector | Hemiptera | DTUI | 16.18333333 | -95.20861111 | Cura et al. 2015 | Google earth | Locality | I | I |
| *Triatoma phyllosoma* | Vector | Hemiptera | DTUI | 16.18333333 | -95.20861111 | Cura et al. 2015 | Google earth | Locality | I | I |
| *Triatoma protracta* | Vector | Hemiptera | DTUI | 36.77805556 | -119.41777778 | Cura et al. 2015 | Google earth | State | I | I |
| *Triatoma sanguisuga* | Vector | Hemiptera | DTUI | 29.65000000 | -82.31670000 | Roellig et al. 2013 | Google earth | Community | I | I |
| *Triatoma sanguisuga* | Vector | Hemiptera | DTUI | 29.65000000 | -82.31670000 | Roellig et al. 2013 | Google earth | Community | I | I |
| *Triatoma sanguisuga* | Vector | Hemiptera | DTUI | 32.51670000 | -81.76700000 | Roellig et al. 2013 | Google earth | Community | I | I |
| *Triatoma sanguisuga* | Vector | Hemiptera | DTUI | 30.50000000 | -81.66000000 | Llewellyn et al. 2009b | Author | NA | I | I |
| *Triatoma sanguisuga* | Vector | Hemiptera | DTUI | 31.96833333 | -99.90166667 | Cura et al. 2015 | Google earth | State | I | I |
| *Triatoma sordida* | Vector | Hemiptera | DTUI | -25.08333333 | -56.96666667 | Maffey et al. 2012 | Google earth | Locality | I | I |
| *Triatoma sordida* | Vector | Hemiptera | DTUI | -25.08333333 | -56.96666667 | Maffey et al. 2012 | Google earth | Locality | I | I |
| *Triatoma sordida* | Vector | Hemiptera | DTUI | -25.08333333 | -56.96666667 | Maffey et al. 2012 | Google earth | Locality | I | I |
| *Triatoma sordida* | Vector | Hemiptera | DTUVI | -25.08333333 | -56.96666667 | Maffey et al. 2012 | Google earth | Locality | II | ND |
| *Triatoma sordida* | Vector | Hemiptera | DTUVI | -25.08333333 | -56.96666667 | Maffey et al. 2012 | Google earth | Locality | II | ND |
| *Triatoma sordida* | Vector | Hemiptera | DTUVI | -25.08333333 | -56.96666667 | Maffey et al. 2012 | Google earth | Locality | II | ND |
| *Triatoma sordida* | Vector | Hemiptera | DTUVI | -25.08333333 | -56.96666667 | Maffey et al. 2012 | Google earth | Locality | II | ND |
| *Triatoma sordida* | Vector | Hemiptera | DTUVI | -25.08333333 | -56.96666667 | Maffey et al. 2012 | Google earth | Locality | II | ND |
| *Triatoma sordida* | Vector | Hemiptera | DTUI | -17.78340000 | -63.01670000 | Barnabe et al. 2011 | Google earth | Province | I | I |
| *Triatoma sordida* | Vector | Hemiptera | DTUI | -17.78340000 | -63.01670000 | Barnabe et al. 2011 | Google earth | Province | I | I |
| *Triatoma sordida* | Vector | Hemiptera | DTUI | -17.78340000 | -63.01670000 | Barnabe et al. 2011 | Google earth | Province | I | I |
| *Triatoma sordida* | Vector | Hemiptera | DTUI | -17.78340000 | -63.01670000 | Barnabe et al. 2011 | Google earth | Province | I | I |
| *Triatoma sordida* | Vector | Hemiptera | DTUI | -17.78340000 | -63.01670000 | Barnabe et al. 2011 | Google earth | Province | I | I |
| *Triatoma sordida* | Vector | Hemiptera | DTUI | -17.78340000 | -63.01670000 | Barnabe et al. 2011 | Google earth | Province | I | I |
| *Triatoma sordida* | Vector | Hemiptera | DTUI | -17.78340000 | -63.01670000 | Barnabe et al. 2011 | Google earth | Province | I | I |
| *Triatoma sordida* | Vector | Hemiptera | DTUI | -17.78340000 | -63.01670000 | Barnabe et al. 2011 | Google earth | Province | I | I |
| *Triatoma sordida* | Vector | Hemiptera | DTUI | -17.78340000 | -63.01670000 | Barnabe et al. 2011 | Google earth | Province | I | I |
| *Triatoma sordida* | Vector | Hemiptera | DTUI | -17.78340000 | -63.01670000 | Barnabe et al. 2011 | Google earth | Province | I | I |
| *Triatoma sordida* | Vector | Hemiptera | DTUI | -17.78340000 | -63.01670000 | Barnabe et al. 2011 | Google earth | Province | I | I |
| *Triatoma sordida* | Vector | Hemiptera | DTUI | -23.45000 | -52.03400 | Zalloum et al. 2005 | Google earth | Community | I | I |
| *Triatoma sordida* | Vector | Hemiptera | DTUI | -23.45000 | -52.03400 | Zalloum et al. 2005 | Google earth | Community | I | I |

*Abbreviations*: ND, not determined

*Some species are not correctly specified in original articles and are georeferenced according to the locality or community
