# Supplementary material for: Geographical, landscape and host associations of Trypanosoma cruzi DTUs and lineages
Source: Parasit Vectors. 2016 Dec 7;9:631. doi: 10.1186/s13071-016-1918-2 (PMC5142175; doi:10.1186/s13071-016-1918-2)
Supplement: Additional file 2: Table S2. — Phylogenetic studies not using analytical methods. (DOCX 30 kb) [file 13071_2016_1918_MOESM2_ESM.docx]

Table S2. Phylogenetic studies non using analytical methods.

| **Article** | **Hypothesis/Aim** | **Parasite population** | **Sample size** | **Geographical scale** | **Temporal scale** | **Population genetic analysis** | **Outgroup** | **Statistical analytical method** |
| --- | --- | --- | --- | --- | --- | --- | --- | --- |
| Herrera et al. (19) | TcI haplotypes and their relationship to the tranmission cycle | Isolates | 12 | Colombia | md | Mini-exon | none | Analysing of the sequences |
| Falla et al. (20) | Haplotipe identification within TcI isolates | Isolates | 37 | Colombia | md | Mini-exon | TcII | Phylogenetic analysis (ML,MP) |
| Broutin et al. (33) | GPI as a reliable marker to explore phylogenetic relationships within Tc | Isolates | 12 | South America | md | GPI gene | *T.c.marinkellei, T.rangeli* | Phylogenetic analysis (ML) Recombination events /Synonymus-nonsynonymus analysis |
| Cura et al. (40) | Tc SL-IR based genotypes and type TcI culture isolates | Isolates + original | 105 | Argentina/Brasil/Chile/Colombia/Mexico/Panama/USA | md | Mini-exon | none | Phylogenetic inferences (MP and Bayesian Inference) |
| Marcili et al. (73) | Phylogenetic relationships among isolates froma bats and other hosts representative of lineages | Isolates | 15 | Brasil | md | SSU rRNA, Cyb and H2B | *T.c.marinkellei, T.dionisii* | Data analysis of three genes (ML) |
| Barnabe and Breniere (104) | Scarce events of mitochondrial introgression in Tc | Isolates | 49 | Bolivia and Peru | md | Nd1 and Gpi | none | ML |
| Bhattacharyya et al. (105) | Molecular diversity of the Tc trypomastigote small surface antigen | Isolates | 25 | America | md | TSSA gene | none | Phylogenetic analysis /non-synonymous nt substitutions per site ratios |
| Briones et al. (106) | Lineage 2 is indigenous to South America while lineage 1 has been introduced to South America recently | Isolates | 16-37 | Tc stocks | md | LSUrDNA and SSU rDNA | *B.caudatus, .borreli, T.rangeli* | Phylogenetic inferences |
| Brito et al. (107) | Genetic characterisation for identify areas where sylvatic and domestic transmission occur | Original | 50 | Five communities, Ceara State, Brazil | md | LSSP-PCR | none | Phenetic analyses |
| Camara et al. (108) | Genetic diversity and population structure of the parasite in the epidemiological chain of transmission to humans | Isolates | 25 | One state, Brazil | md | Microsatellite loci | none | Phylogenetic inferences using Wagner algorithm |
| Carranza et al. (109) | ND7 constitute a valuable target for PCR assays in the differential diagnosis of the infective Tc strain | Isolates | 16 | Brasil | md | ND4-CR4, COIII, COII and ND7 and microsatellite loci | none | Microsatellite analysis, Phylogenetic inference |
| Cerqueira et al. (110) | Genetic variability among various types of multigene families | Isolates | 6 | Tc stocks | md | 3 genes | none | Sequence distances and phylogenetic trees |
| Cuervo et al. (111) | Genetic diverstity of Colombian sylvatic Tc isolates | Isolates | 14 | Colombia | md | RFLP-rDNA | *T.rangeli, T.dionisii* | Jaccard, UPGMA |
| D'Avila et al. (112) | Population dynamics of Tc during progression of the chronic phase in chagasic patients | Isolates | 102 | Brasil | 10 years | Microsatellite loci, 24s rRNA, COII and SL-IR | none | Phylogenetic trees (UPGMA) |
| Herrera et al. (113) | Genetic variability and phylogenetic relationships within TcI | Isolates | 31 | Colombia | md | Mini-exon | TcII | MP and ML /Bayesian inference |
| Hwang et al. (114) | Genotyping of trypanosome isolates | Original | 34 | Two communities, California | 3 months | 24s rRNA | *T.rangeli* | Phylogenetic approaches (MP, ML and Bayesian inference) |
| Iwagami et al. (115) | Genetic exchange of sexual reproduction would enhance the opportunities for adaptative radiation and might be associated with the variable clinical manifestations | Isolates | 21 | Central and South America | md | DHFR-TS, TR and COII-NDI | *T.c. marinkellei*, *T.vespertilionis* | Phylogenetic analysis/ NJ |
| Kawashita et al. (116) | Maximum-Likelihood Divergence Date Estimates Based on rRNA Gene Sequences | Isolates | 20 | Tc stocks | md | 18S rDNA and 24srDNA | *T.rangeli, T.c.marinkellei* | Phylogenetic analysis (ML) |
| Lewis et al. (117) | DNA content diversity in natural populations of Tc in the context of its genetic subdivision | Isolates | 54 | Americas | md | Microsatellite loci | none | Mantel test (Genalex), Student's t-test and ANOVA |
| Luna-Marin et al. (118) | "not all parasite clones present in insect are found in infected patients"/Genetic variability of TcI | Isolates | 16 | Five communities | 5 years | RAPD, ITS-RFLP | *Leishmania chagasi* | Jaccard |
| Machado and Ayala (119) | Genetic exchange among distanly related lineages | Isolates | 46 | Tc stocks | md | Nuclear and mitochondrial sequences | *T.c.marinkellei,T.vespertilionis* | Phylogenetic analyses/Estimating divergence times |
| Martinez et al. (120) | Characterization of Mexican human TcI strains | Isolates | 20 | Mexico | md | Mini-exon and microsatellite loci | none | Phylogenetic analysis and drug susceptibility (IC50 determination) |
| Martins et al. (121) | Relationship of parasite genetic diversity with its pathogenicity and virulence in host | Isolates | 5 | One state, Brazil | md | SSU rRNA | *T.c. marinkellei* | Sequencing |
| Messenger et al. (122) | Mitochondrial DNA ro reveal diversity hidden at the sub-DTU level | Isolates | 32 | Americas | md | mtMLST, GPI, mini-exon and microsatellite loci | none | Phylogenetic analysis |
| O'Connor et al. (123) | Genetic variability and the phylogenetic relationships within TcI usin sequences of non-transcribed spacer of miniexon genes | Isolates | 20 | Mexico, Bolivia, Argentina, Colombia, Brasil, USA | 31 years | Mini-exon | none | Phylogenetic analysis (ML) |
| Perez et al. (124) | Genetic diversity of Tc DTUs infecting *T.infestans* collected before and after spraying | Original | 54 | Bolivian Gran Chaco | 2 years | Mini-exon and GPI | *T.c. marinkellei* | Phylogenetic tree (ML) |
| Roellig et al. (125) | Molecular diversity of Tc from the US | Isolates | 50 | USA | md | Multilocus Sequence Typing (4targets) | *T.c.marinkellei,* *T.brucei* | Phylogenetic analysis /Phylogenetic trees NJ,ME, MP methods |
| Rozas et al. (126) | Value of antigen gene-unique interfaces for the understanding of the evolution within Tc | Isolates | 20 | Referenced (Rozas et al. 2007) | md | Eleven antigen-encoding genes | *T.c.marinkellei* | Phenetic analysis |
| Salazar et al. (127) | Genetic relationship among stocks from distinct geographical regions and different vector species and host | Isolates | 30 | Colombia | md | LSSP-PCR | *T.rangeli* | UPGMA |
| Santos et al. (128) | Genetic diversity and phylogenetic relationships of isolates | Isolates | 29 | Brazil | md | ITS-RFLP | *T.rangeli* | Phylogenetic analysis (NJ) |
| Spotorno et al. (129) | Identify and characterize chilean samples of Tc and their association with hosts | Isolates | 28 | Chile and American sequences | md | Cyb | *T.c.marinkellei* | Phylogenetic analyses (MP, NJ, ML) |
| Subileau et al. (130) | Basic research of Tc: phylogeny, mode of reproduction | Isolates | 20 | Bolivia, Brasil, Venezuela, USA, Paraguay, Chile | md | 3 nuclear and one maxicircle gene | *T.c.marinkellei* | Phylogenetic analysis (ML)/ Recombination events /Synonymus-nonsynonymus analysis |
| Telleria et al. (131) | Relationships between the parasite's population structure and kDNA polymorphism | Isolates | 19 | South America | md | kDNA minicircle variable regions | *T.c.marinkellei* | Jaccard, UPGMA |
| Tomasini et al. (132) | Interest and limitations of SL-IR sequences for analysing TcI phylogenetic diversity | Isolates | 25 | Chaco Province, Argentina | md | Mini-exon | none | 4 recombination tests (RDP/GENCONV/Bootscan/MaxChi) |
| Triana et al. (133) | Genetic characterisation for epidemiological purpose | Isolates | 19 | Colombia | 32 years | Molecular karyotupe and minicircle Southern blot | *T.rangeli* | CSDI, UPGMA |
| Venegas et al. (134) | Two hybridation events as the origin of Tc lineages | Isolates | 19 | Brasil, Colombia, Chile, Bolivia | md | Microsatellite loci | none | Phylogenetic analysis (ML) |
| Westenberger et al. (135) | Two hybridation events define the population structure of Tc | Isolates | 26 | Americas | md | Amplification of nine loci marker/RFLP analyses | none | Phylogenetic analysis (NJ) |
| Westenberger et al. (136) | 5SrRNA intergenic region is a useful diagnostic marker for determining Tc strains DTU identity and provenance | Isolates | 46 | Americas | md | Amplification of 5srRNA and Southern Blot | none | Phylogenetic analysis (NJ,MEGA) |
| Yeo et al. (137) | MLST gene loci for lineage assignment, inter-DTU relationships and for population genetic analysis of Tc | Isolates | 39 | Americas | md | Nine single copy housekeeping genes | none | Diploid sequence typing/Phylogenetic analysis/ Recombination test |
| Zafra et al. (138) | Correlation between the genetic profile of the Tc group circulating in the biological cycle and that of the group directly responsible for the clinical form and severity of the disease | Isolates + original | 20 | Santander, Colombia | md | Miniexon sequencing, RFLP-PCR, microsatellites | none | Nei and Li algorithm |
| Zalloum et al. (139) | Genetic variability associated with Tc virulence | Isolates | 12 | One state, Brazil | md | RAPD, SSR-PCR | none | Jaccard, UPGMA |
| Zingales et al. (140) | Epidemiology, biochemistry and evolution of Tc Lineages | Isolates | 88 | Latin America countries | md | LSU rDNA | *T.rangeli* | Phylogenetic analysis (UPGMA) |

*Abbreviations*: md, missing data; Tc, *Trypanosoma cruzi*
