# Supplementary material for: Geographical, landscape and host associations of Trypanosoma cruzi DTUs and lineages
Source: Parasit Vectors. 2016 Dec 7;9:631. doi: 10.1186/s13071-016-1918-2 (PMC5142175; doi:10.1186/s13071-016-1918-2)
Supplement: Additional file 3: Table S3. — Phylogeographic studies not using analytical methods. (DOCX 15 kb) [file 13071_2016_1918_MOESM3_ESM.docx]

Table S3. Phylogeographic studies non using analytical methods

| **Article** | **Hypothesis/Aim** | **Parasite population** | **Sample size** | **Geographic scale** | **Temporal scale** | **Population genetic analysis** | **Outgroup** | **Statistical analytical method** |
| --- | --- | --- | --- | --- | --- | --- | --- | --- |
| Marcili et al. (64) | Isolate characterization and intra-lineage genetic diversity | Isolates | 78 | Three states, Brazil | md | SSrRNA, Cyb sequencing and RAPD | none | Sequencing (alignment and dendrograms) |
| Marcili et al. (145) | Association between Tc genotype with ecotopes, region, and hosts | Isolates | 28 | Amazonian and Atlantic rainforest | md | SSU rDNA and cyb sequencing and RFLP | *T.dionisii, T.c. marinkellei* | Sequencing (alignment and dendrograms) |
| Mejia-Jaramillo et al. (146) | TcI populations associations with geographical distribution and different sylvatic vector species | Isolates | 47 | Colombia | md | LSSP-PCR | TcII | Genetic distances (NJ) |

*Abbreviations*: md, missing data; Tc, *Trypanosoma cruzi*
