# Supplementary material for: Geographical, landscape and host associations of Trypanosoma cruzi DTUs and lineages
Source: Parasit Vectors. 2016 Dec 7;9:631. doi: 10.1186/s13071-016-1918-2 (PMC5142175; doi:10.1186/s13071-016-1918-2)
Supplement: Additional file 4: Table S4. — Number of species and records reported in different orders. (DOCX 16 kb) [file 13071_2016_1918_MOESM4_ESM.docx]

Table S4. Number of species and records reported in different orders

|  | Artiodactyla | | Carnivora | | Carnivora without pets | | Cingulata | | Didelphimorphia | | Primates | | Primates without humans | | Rodentia | | Chiroptera | |
| --- | --- | --- | --- | --- | --- | --- | --- | --- | --- | --- | --- | --- | --- | --- | --- | --- | --- | --- |
|  | #species | n | #species | n | #species | n | #species | n | #species | n | #species | n | #species | n | #species | n | #species | n |
| DTUI | 2 | 2 | 3 | 56 | 1 | 1 | 1 | 3 | 5 | 190 | 10 | 294 | 9 | 21 | 13 | 73 | 12 | 15 |
| DTUII | 0 | 0 | 1 | 1 | 0 | 0 | 0 | 0 | 2 | 2 | 4 | 19 | 3 | 8 | 0 | 0 | 4 | 4 |
| DTUIII | 0 | 0 | 1 | 5 | 0 | 0 | 3 | 63 | 3 | 16 | 1 | 1 | 0 | 0 | 2 | 2 | 0 | 0 |
| DTUIV | 0 | 0 | 3 | 26 | 2 | 21 | 1 | 1 | 0 | 0 | 8 | 45 | 7 | 10 | 0 | 0 | 0 | 0 |
| DTUV | 0 | 0 | 2 | 7 | 0 | 0 | 0 | 0 | 0 | 0 | 1 | 34 | 0 | 0 | 0 | 0 | 0 | 0 |
| DTUVI | 2 | 4 | 2 | 65 | 0 | 0 | 0 | 0 | 0 | 0 | 1 | 3 | 0 | 0 | 2 | 2 | 2 | 2 |
| *T. cruzi* | 4 | 6 | 12 | 160 | 3 | 22 | 5 | 67 | 10 | 208 | 25 | 396 | 19 | 39 | 17 | 77 | 18 | 21 |
