# Supplementary figures and images for: Geographical, landscape and host associations of Trypanosoma cruzi DTUs and lineages
Source: Parasit Vectors. 2016 Dec 7;9:631. doi: 10.1186/s13071-016-1918-2 (PMC5142175; doi:10.1186/s13071-016-1918-2)

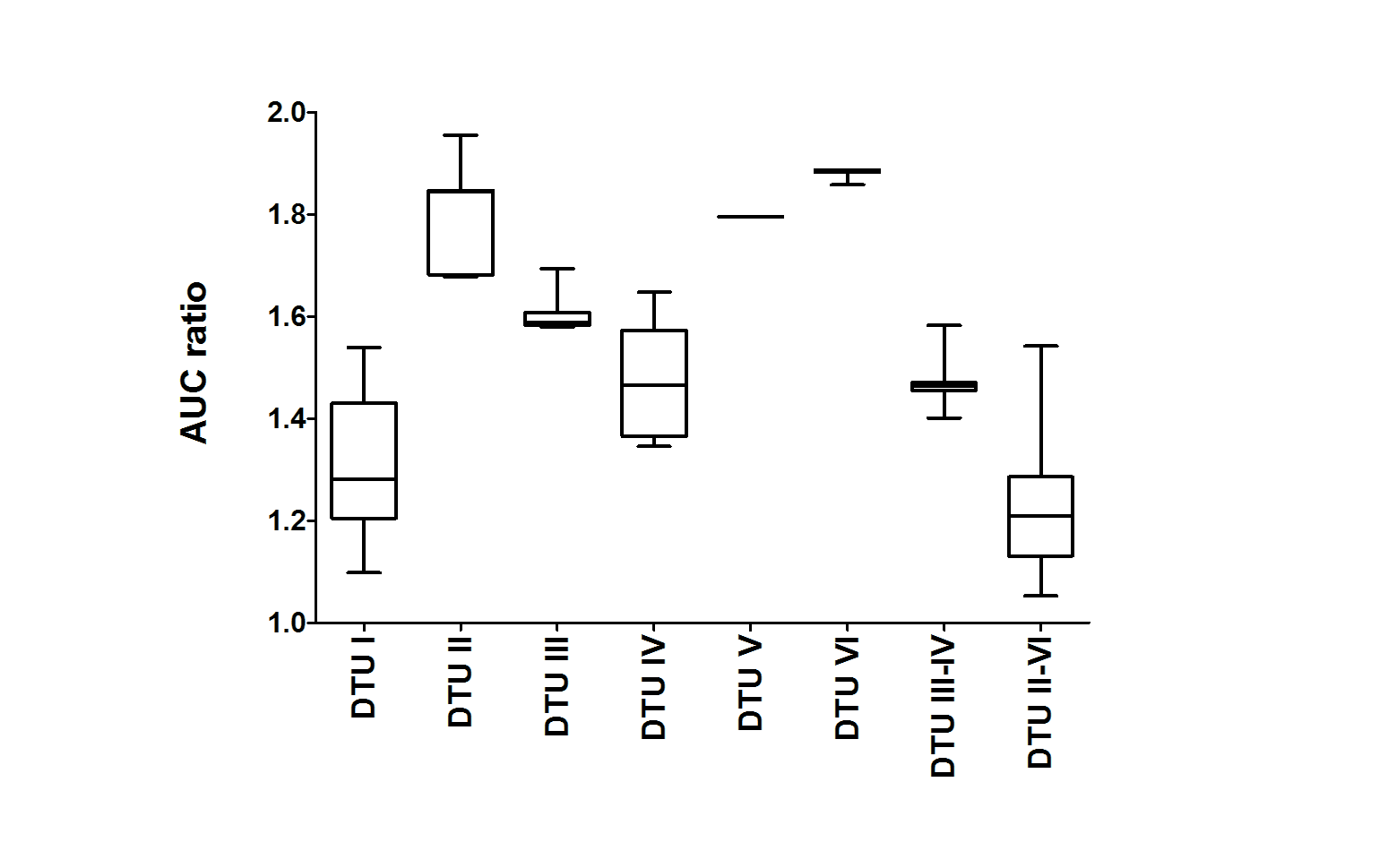

Supplement: Additional file 5: Figure S1. — Ecological niche significance values. (TIF 40 kb) [file 13071_2016_1918_MOESM5_ESM.tif]
